# Au(iii)-aryl intermediates in oxidant-free C–N and C–O cross-coupling catalysis†
†Electronic supplementary information (ESI) available: Detailed synthesis and full characterization of substrates, products and complexes. Crystallographic data for compounds **1ah**, **3a** and **3b**. CCDC 1489676, 1489681 and 1489682, respectively. For ESI and crystallographic data in CIF or other electronic format see DOI: 10.1039/c6sc03699f



**DOI:** 10.1039/c6sc03699f

**Published:** 2016-09-23

**Authors:** Jordi Serra, Teodor Parella, Xavi Ribas

**Affiliations:** a QBIS-CAT Group , Institut de Química Computacional i Catàlisi (IQCC) , Departament de Química , Universitat de Girona , Campus Montilivi , Girona , E-17003 , Catalonia , Spain . Email: xavi.ribas@udg.edu; b Servei de RMN , Facultat de Cieǹcies , Universitat Autoǹoma de Barcelona , Campus UAB , Bellaterra E-08193 , Catalonia , Spain

## Abstract

Au(iii)-aryl species have been unequivocally identified as reactive intermediates in oxidant-free C–O and C–N cross coupling catalysis. The crystal structures of cyclometalated neutral and cationic Au(iii) species are described and their key role in 2 electron-redox Au(i)/Au(iii) catalysis in C–O and C–N cross couplings is shown. Nucleophiles compatible with Au-catalyzed cross couplings include aromatic and aliphatic alcohols and amines, as well as water and amides.

## Abstract 2

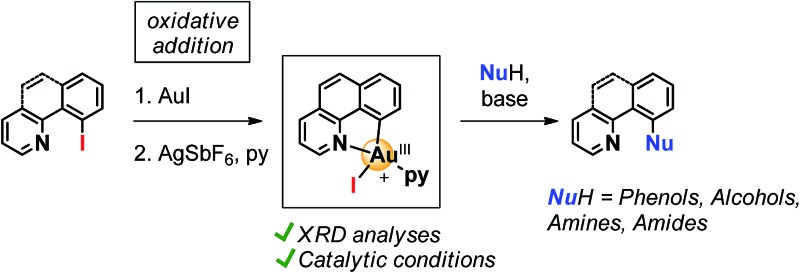
Au(iii)-aryl species have been crystallographically isolated as reactive intermediates in oxidant-free C–O and C–N cross coupling processes, using aromatic and aliphatic alcohols and amines, as well as water and amides, as nucleophiles.

## Introduction

**Scheme 1:**
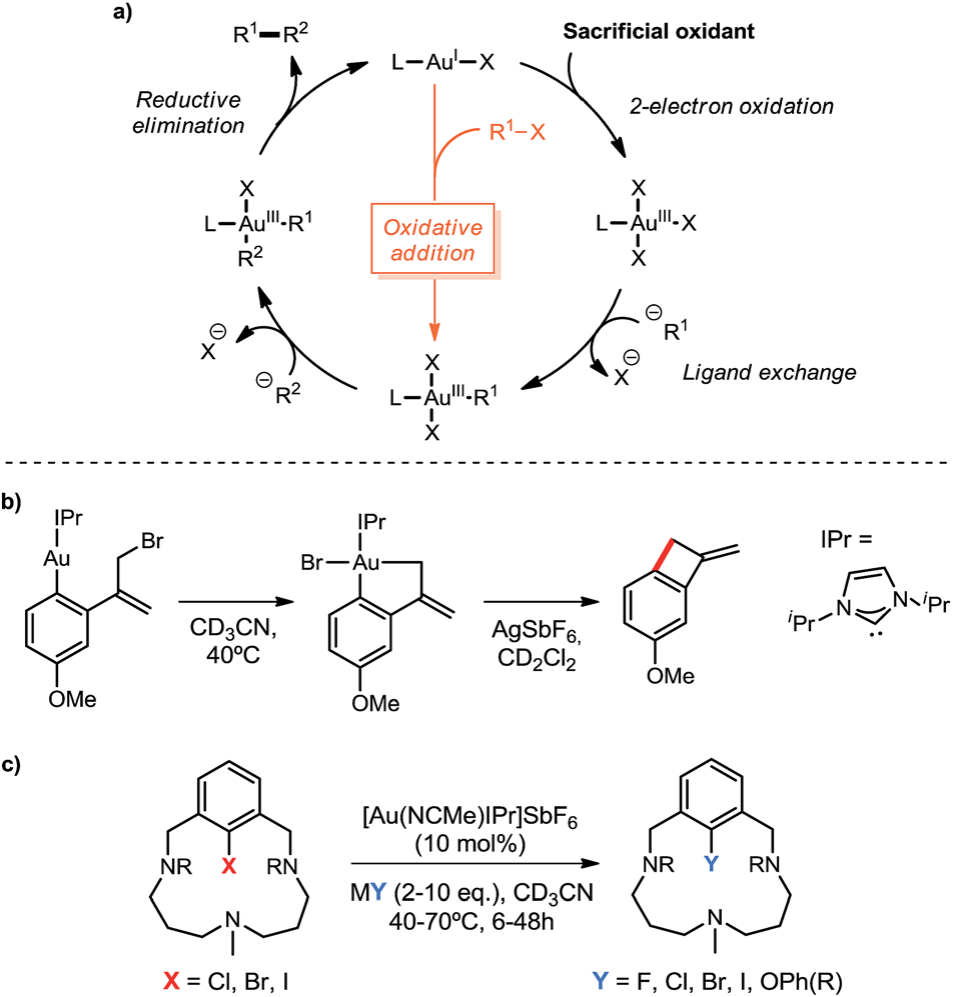
(a) Common 2-electron redox mechanistic cycle in Au(i)/Au(iii) oxidative catalysis, featuring in orange the alternative oxidative addition pathway to Au(iii) intermediates; (b) oxidant-free Toste's allylation of arenes and (c) Ribas' halogen exchange and C–O coupling catalysis.

In the last few years, the use of gold in homogeneous catalysis has experienced increasing attention and progress. Although typically regarded as superior Lewis acids for the activation of multiple C–C bonds towards nucleophiles,^1^ gold complexes have recently found new patterns of reactivity, namely 2-electron redox processes applied to cross-coupling transformations.^2^ In spite of the growing number of examples in this field, the access to key Au(iii) intermediates has been limited to harsh sacrificial oxidants, such as I^3+^ derivatives or F^+^ sources (Scheme 1a),^3^ as well as using highly electrophilic aryldiazonium salts under photoredox conditions or *via* light-driven radical chain reactions.^4^ On the other hand, the straightforward pathway through the oxidative addition of C_sp^2^_–X and C_sp^3^_–X bonds (X = halide) to Au(i) has generally been regarded as highly reluctant,^5^ markedly differing from other transition-metal chemistry.^6^ However, recent in-depth organometallic investigations have dismissed this conception with remarkable achievements. The first evidence for the intramolecular oxidative addition of aryl halides to gold(i) complexes was disclosed by the Bourissou group in 2014,^7^ showing that phosphine-chelation assistance is key to delivering the C_sp^2^_–X bond in close proximity to the gold center, thus promoting the oxidative addition even at room temperature for X = I. The same group later took advantage of the ability of carborane diphosphines to chelate gold(i) with small P–Au–P bite angles, which render a preorganized architecture closer in energy to the ensuing square-planar geometry of the oxidative addition product. By means of this strategy, intermolecular oxidative addition of aryl iodides and strained C–C bonds under mild conditions was accomplished.^8^ Also in 2014, the Toste group substantiated the ability of gold to perform the elementary steps of organometallic cross-coupling chemistry, including oxidative addition, with the first example of a Au(i)-catalyzed C–C bond formation without the requirement of external oxidants.^9^ A tethered Au(i) aryl complex featuring an allyl bromide moiety allowed to support this mechanism *via* oxidative addition under intramolecular conditions (Scheme 1b).

In our group we envisioned that a coordinating environment could favor the oxidative addition of Au(i) salts to aryl halides and the following reactivity towards nucleophiles. This strategy, based on attaching a macrocyclic appendage to aryl halide substrates bearing three available nitrogen coordination sites, had actually proved extremely beneficial in the isolation and exhaustive characterization of square pyramidal aryl-Cu(iii) and aryl-Ag(iii) complexes in the context of 2*e* ^–^ redox M(i)/M(iii) cross coupling catalysis.^10^ Hence, using this approach we were able to describe the first oxidant-free gold(i)-catalyzed halide exchange and C_sp^2^_–O bond forming reactions (Scheme 1c), additionally transferring this novel chemistry to more easily available substrates such as 2-(2-halophenyl)pyridines.^11^

In this work we present the expansion of the nucleophile scope from halides and phenols to amines and amides, which stands as the first example of a gold(i)-catalyzed C–N bond formation, resembling the well-known Cu-based Ullmann-type^12^ or Pd-based Buchwald–Hartwig cross-coupling catalysis.^13^ Furthermore, the straightforward synthesis and crystallographic characterization of the hitherto new (N,C)-cyclometalated Au(iii) complexes **3a** and **3b** *via* the oxidative addition of a C_sp^2^_–I bond is also herein described. Their competency as intermediate species in catalytic C–O and C–N couplings is also demonstrated, therefore confirming the redox Au(i)/Au(iii) mechanistic cycle previously postulated.

## Results and discussion

In the course of our investigations into the oxidant-free gold(i)-catalyzed halogen exchange we found that the applicability of the system could be extended to C–O coupling reactions using sodium *p*-chlorophenolate and, interestingly, sodium methoxide. The latter required its conjugate acid (MeOH) as solvent to proceed, presumably due to the low solubility of the salt in CH_3_CN. This prompted us to study the related transformations with other alkoxides and protic solvents. In a similar manner, EtONa was reacted with 2-(2-bromophenyl)pyridine **1a-I** in ethanol (110 °C, [Au(NCMe)IPr]SbF_6_ as catalyst) and after 24 h a moderate 56% yield of the desired coupling product **1ac** was obtained, which could be increased up to 78% after 48 h (Table 1, entry 6). This can be attributed both to the lower acidity of EtOH compared to MeOH and the larger steric hindrance of the ethoxide. The preference for smaller and more acidic alkoxides was confirmed by carrying out the reaction with MeONa in EtOH, whereby **1ab** was the major product. This trend could be further extrapolated to more sterically demanding alkoxides such as 2-propoxide (**1ad**) and *tert*-butoxide (**1ae**) giving yields of 21% and 4%, respectively (Table 1, entries 7 and 8).

**Table 1:** Au(i)-catalyzed C–O bond formation with different NaO–R^*a*^

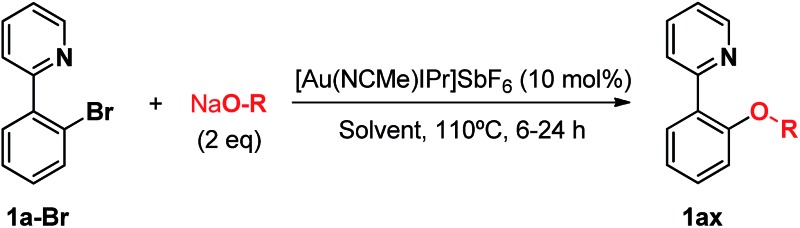					
Entry	R–ONa	Solvent	Time (h)	Product	Yield^*b*^ (%)
1	*p*Cl-PhONa	CH_3_CN	6	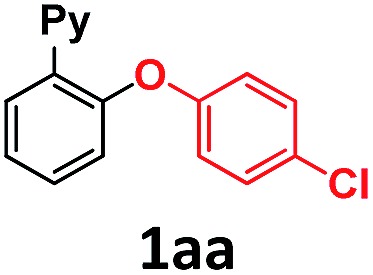	>99^*c*^
2	HONa	MeOH	8	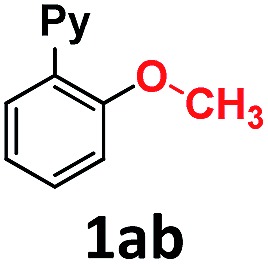	>99
3	MeONa	MeOH	8		>99^*c*^
4	EtONa	MeOH	24		>99
5	MeONa	EtOH	24		86 (9)^*d*^
6	EtONa	EtOH	24	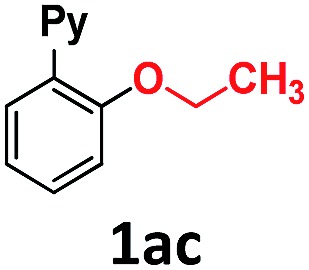	56 (78)^*e*^
7	(2-Propoxide)Na^*f*^	2-Propanol	48	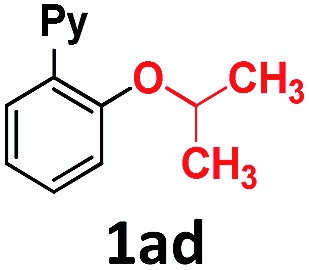	21
8	*t*-BuONa	*tert*-Butanol	48	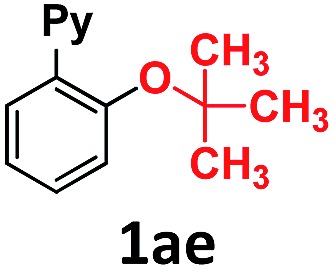	4
9	HONa	H_2_O	24	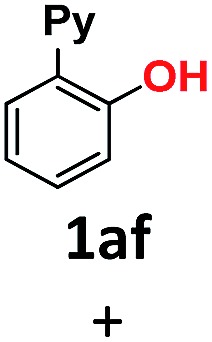	34 (**1af**), 26 (**1ag**)
10	MeONa	H_2_O	24	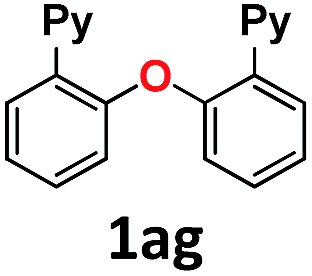	52 (**1af**), 21 (**1ag**)

^*a*^General conditions: [2-(2-halophenyl)pyridine] = 20 mM, [alkoxide] = 40 mM, 0.5 mL solvent, 110 °C.

^*b*^Calculated with ^1^H-NMR spectroscopy using 1,3,5-trimethoxybenzene as the internal standard.

^*c*^ Ref. 11.

^*d*^In parentheses, the yield of the ethoxide insertion product.

^*e*^In parentheses, the yield after 48 h.

^*f*^The alkoxide was generated *in situ* adding 2 equivalents of sodium *tert*-butoxide as a base.

The use of sodium hydroxide as a nucleophile (in H_2_O solvent) deserves a special mention. Although in terms of reactivity one might anticipate a similar behavior to sodium methoxide in MeOH (Table 1, entry 3) to readily provide 2-(pyridin-2-yl)phenol **1af**, this was only formed in a 34% yield (Table 1, entry 9). On the contrary, the diaryl ether homocoupling product **1ag** was the major product (26% yield). A simple explanation for this outcome involves the deprotonation in basic media of the early-stage generated phenol, which subsequently acts as the preferred nucleophile in the coupling reaction with **1a-Br** starting material. It should be noted that this transformation using NaOH as a base was performed in aqueous media, in the absence of a phase-transfer reagent and without the addition of a co-solvent. In an effort to gain a better understanding of this reactivity, we carried out the same reaction using NaOMe instead of NaOH, and the yield of phenol **1af** increased up to 52% (Table 1, entry 10). These results suggest that control over the selectivity and yield of each product might be achieved by the appropriate modification of the reaction conditions. Notably, the combination of sodium hydroxide and methanol led to the complete formation of the methoxy insertion product **1ab** (Table 1, entry 2).

Overall, we have shown that steric and pH effects play a crucial role in the Au(i)-catalyzed ether and phenol formation, with smaller and more basic alkoxides leading to better results. This methodology allows for the synthesis of 2-(pyridin-2-yl)phenol **1af** in water and 2-(2-methoxyphenyl)pyridine **1ab** and 2-(2-ethoxyphenyl)pyridine **1ac** in moderate to excellent yields, and represents the first example of the Au-catalyzed cross-coupling of aliphatic alcohols to aryl halides. Moreover, gold promotes the coupling of water to form phenols, and the coupling of linear aliphatic alcohols to form ethers, thus presenting a complementary methodology to the Cu-based C–O cross-couplings for these challenging nucleophiles.^14^

In light of these promising results, we then sought to expand the Au-catalyzed C-heteroatom reaction scope towards C_sp^2^_–N bond formation through the combination of aryl halides and N-nucleophiles, typically relying on two different approaches: (a) Cu-catalyzed Ullmann-type and (b) Pd-catalyzed Buchwald–Hartwig coupling reactions. As far as we know, this reactivity has only been successfully transferred to nickel.^15^ We initially selected *p*-nitroaniline, given its major acidity compared to other amines (p*K*_a_ = 20.9 in DMSO), as the nucleophile to be reacted with **1a-Br** in the presence of 10 mol% [Au(NCMe)IPr](SbF_6_) at 110 °C (Table S1†). Regardless of the base used, in all cases the starting material was recovered. Thus, afterwards, optimization of the solvent was investigated, whereby we obtained a significant and encouraging 39% yield of the desired product in DMSO and with KO*t*-Bu as a base. The same conditions employing the more reactive **1a-I** substrate resulted in almost quantitative yields. The final optimization set the preferred experimental conditions at 110 °C, 24 h and an excess of the nucleophile and 3 equivalents of base for further substrate scoping. It is worth pointing out the high-performance of KO*t*-Bu as a base without competing with *p*-nitroaniline as the nucleophile in the coupling reaction, as foreseen from the C–O bond forming results (Table 1, entry 8). Detailed NMR and X-ray crystallography analysis of the isolated product confirmed the expected structure. Remarkably, a gold-free blank experiment was performed and the reaction did not proceed (0% yield). The sluggishness of the reaction when **1a-Br** is used instead of **1a-I** is in good agreement with a rate-limiting oxidative addition step.

**Scheme 2:**
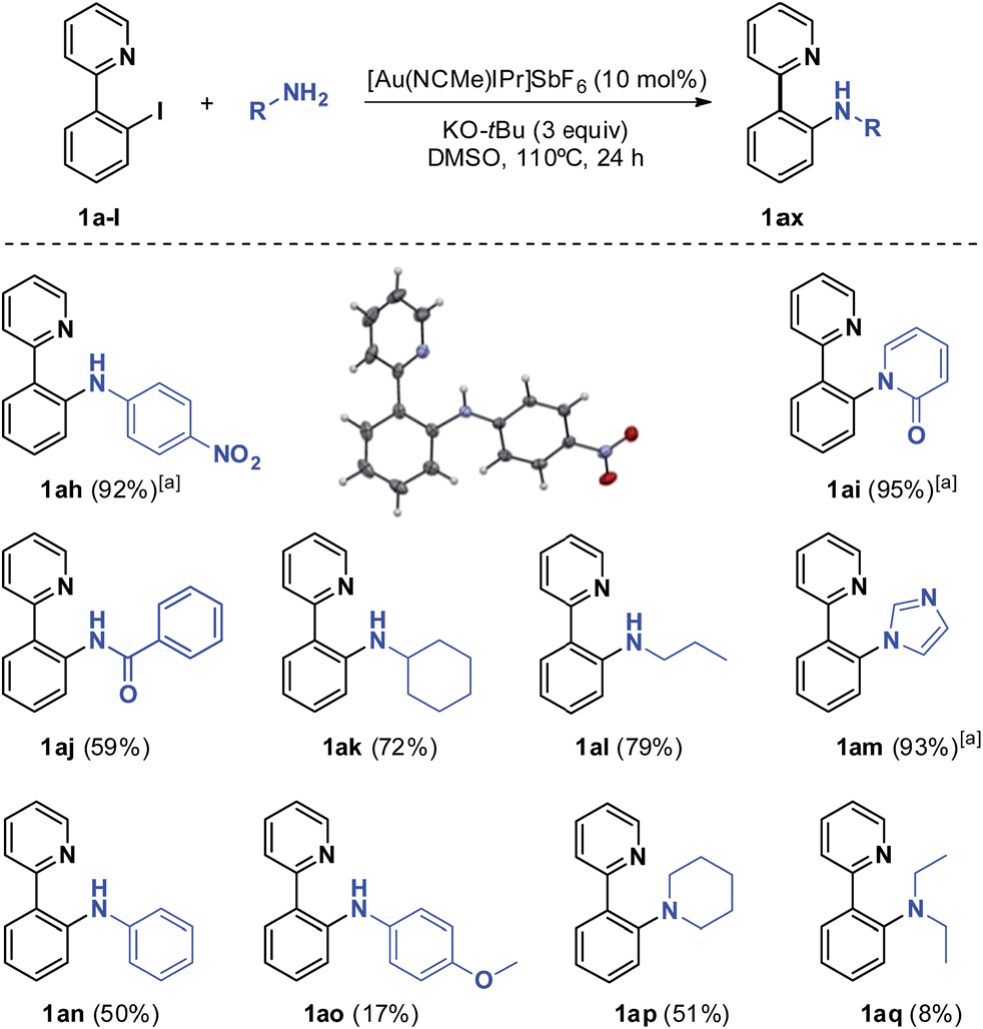
Substrate scope of the C–N bond forming reactions. ^[a]^100 °C.

Then the scope of the optimized protocol was examined using different amines and amides. Cyclic aromatic and aliphatic amines and amides (imidazole and 2-hydroxypyridine, Scheme 2, products **1am** and **1ai**) afforded the best results, most likely owing to their superior acidity (p*K*_a_ = 18.6 and 17.0 in DMSO, respectively).^16^ Good outcomes were obtained for primary aliphatic amines (Scheme 2, products **1ak** and **1al**), while secondary amines were less prone to arylation, with moderate and low yields for piperidine (**1ap**) and diethylamine (**1aq**), respectively. Likewise, benzamide was arylated in a moderate yield (59%, **1aj**). Essentially, acidity and steric hindrance seem to be the basis of the observed reactivity trend for the aryl iodide **1a-I**. We then turned our attention to the electronic properties of the *para*-substituents on the aniline ring. In contrast to the excellent results provided by *p*-nitroaniline, aniline did not exceed 50% yield, while *p*-methoxyaniline was poorly arylated (17%, **1ao**). This observation, together with the data collected for the other N-nucleophiles, indicates sensitivity to less acidic and sterically hindered substrates, which is translated into a decrease in yields. In this system, the Au-based methodology for the arylation of aliphatic amines is superior to Cu-based Ullmann-type couplings.^14^

C–N cyclometalated gold(iii) complexes have been the subject of deep investigation, ever since the first report of neutral AuCl_2_(ppy) containing a 2-phenylpyridine-type ligand by Constable and co-workers.^17^ This family of complexes has shown potential anticancer activity^18^ and photophysical properties,^19^ and their use has benefited from their tolerance to both air and water. The methods to prepare them require either transmetallation from toxic organomercury derivatives or formal C–H auration with gold(iii) tri or tetrahalide salts.^20^ Furthermore, an alternative approach, employing aryldiazonium salts and Au(i) complexes under visible light photoredox conditions, has been developed very recently.^4*c*^ Nevertheless, since their synthesis had not been previously realized *via* the oxidative addition of C_sp^2^_–X bonds, we deemed it worthy to investigate this possibility using the 2-(2-halophenyl)pyridine substrates.

**Scheme 3:**
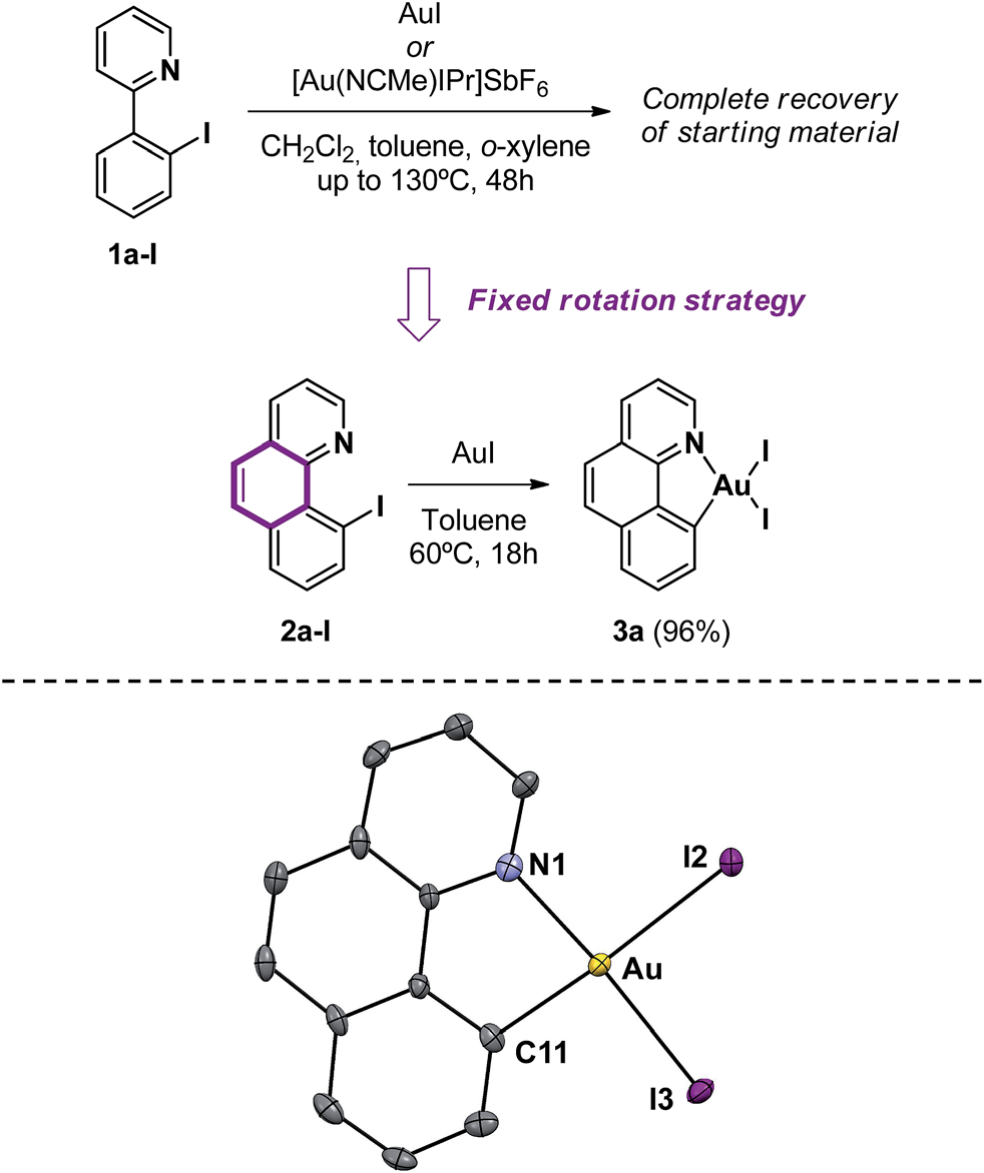
Synthetic approach to Au(iii) complex **3a** and the molecular structure in the solid state. Selected bond lengths [Å] and angles [°]: Au–C(11) = 2.055(7), Au–N(1) = 2.113(8), Au–I(2) = 2.6402(6) and Au–I(3) = 2.5755(7); C(11)–Au–I(3) = 94.5(2), C(11)–Au–N(1) = 81.5(3), I(2)–Au–I(3) = 89.3(2) and N(1)–Au–I(2) = 94.8(2). (Ellipsoids are set at 50% probability; hydrogen atoms have been omitted for clarity).

Inspired by the work of Bourissou and co-workers,^7^ we first reacted 2-(2-iodophenyl)pyridine **1a-I** and AuI in dichloromethane at room temperature. However, the complete recovery of the starting materials was observed, even after changing the solvent to toluene and *o*-xylene and heating up to 130 °C. A cationic [Au(NCCH_3_)IPr]SbF_6_ gold(i) source gave identical results, regardless of the reaction conditions. This observation suggests the equilibrium displacement towards more stable reagents (see the ESI,† Section 1.6, for details). To tackle the aforementioned difficulties, we envisaged to block the rotation of the pyridine chelating group by incorporating an extra ring into the substrate, aiming at enhancing the stability of the desired product. Consequently, 10-iodobenzo[*h*]quinolone **2a-I** was prepared following a two-step procedure starting from benzo[*h*]quinolone (see the ESI†). Gratifyingly, oxidative addition of the C_Ar_–I bond proceeded readily with AuI at 60 °C for 18 h to give the cyclometalated Au(iii) complex **3a** as a red powder (Scheme 3). Complex **3a** withstands air and water indefinitely and is only soluble in CH_2_Cl_2_, CHCl_3_ and DMSO. X-ray-quality crystals were obtained by gently stirring **2a-I** and AuI in CH_2_Cl_2_ at room temperature for 4 days, followed by slow evaporation of the solvent. The solid-state structure of the complex displays a Au–C bond length of 2.055(7) Å, indicative of a Au(iii) center, and a distorted square-planar environment owing to the constraints of the five-membered (N,C) chelate ligand, with a C11–Au–N1 angle of 81.5(3) and an associated N1–Au–I2 angle of 94.8(2).

The bond lengths in **3a** are similar to those reported for the related cyclometalated [Au(ppy)Cl_2_]^18*a*^ and [Au(tpy)Br_2_]^21^ complexes. Specifically, the Au–I distances are longer than the analogous Au–Cl and Au–Br ones, consistent with bigger and more labile iodine atoms. The Au–I3 distance (2.5755(7) Å) *trans* to the pyridyl nitrogen is shorter than the Au–I2 distance (2.6402(6) Å) as a result of the greater structural *trans* influence of the aryl group. Most diagnostic of the formation of a C–Au(iii) bond is the low-field shift of the ^1^H-NMR signal corresponding to the α-proton in the pyridine moiety from *δ* 9.11 in **2a** to 10.35 ppm, as well as the disappearance of the ^13^C-NMR signal of the C–I atom at *δ* 88.9 ppm and the appearance of a new signal at *δ* 153.6 ppm, corresponding to the quaternary carbon ligated to the Au(iii) center.

**Scheme 4:**
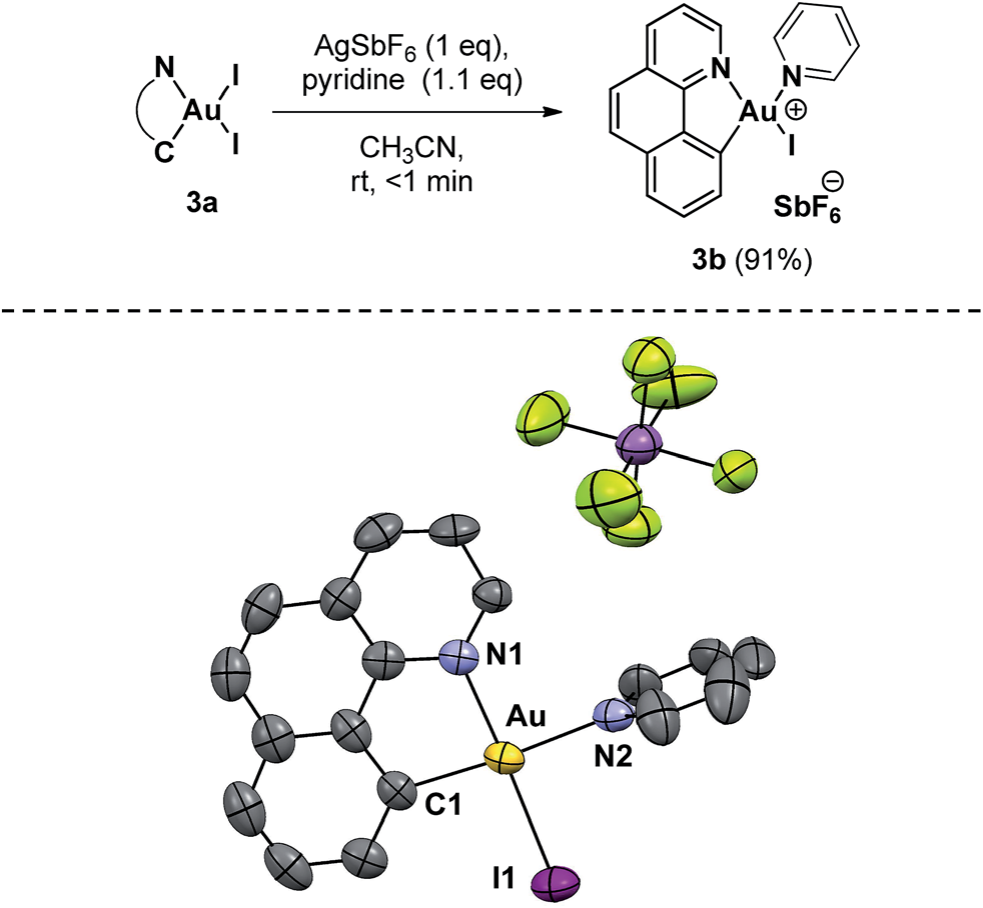
Synthetic approach to Au(iii) complex **3b** and the molecular structure in the solid state. Selected bond lengths [Å] and angles [°]: Au–C(1) = 2.023, Au–N(1) = 2.068(7), Au–N(2) = 2.119(8) and Au–I(1) = 2.556(2); C(1)–Au–I(1) = 95.1(3), C(1)–Au–N(1) = 82.8(3), N(2)–Au–I(1) = 89.2(2) and N(1)–Au–N(2) = 93.0(3). (Ellipsoids are set at 50% probability; hydrogen atoms have been omitted for clarity).

With neutral **3a** in hand, we next investigated the abstraction of iodide using 1 equivalent of AgSbF_6_ in order to generate a more soluble and reactive cationic complex (Scheme 4). The reaction was carried out in acetonitrile due to its coordinating properties, but no changes were detected after vigorously stirring for 3 hours at room temperature. At this point we hypothesized that the medium-strength Lewis base character of acetonitrile cannot efficiently stabilize a three-coordinate gold(iii) species, and consequently a stronger Lewis base was employed. Upon the addition of pyridine (1.1 equiv.), **3a** was immediately consumed and the solution turned bright yellow with the formation of an abundant grey precipitate (AgI). Complex **3b** was isolated as an orange powder after filtration and solvent removal (91% yield). **3b** is also air- and moisture-stable and can be stored on the benchtop without noticeable decomposition. The ESI-HRMS mass spectrum of this complex shows one major peak at *m*/*z* 580.9782 corresponding to the mass of [(**2a**)AuI(Py)]^+^, the cationic fragment of **3b**. Low-temperature ^1^H-NMR (248 K) allowed the structural characterization of **3b** in solution (see the ESI†). Crystals of this complex, grown from the slow diffusion of diethyl ether in a CH_3_CN solution of the compound, confirmed the removal of the iodide *trans* to the Au–C bond, as expected from the larger *trans* effect (Scheme 4).

**Scheme 5:**
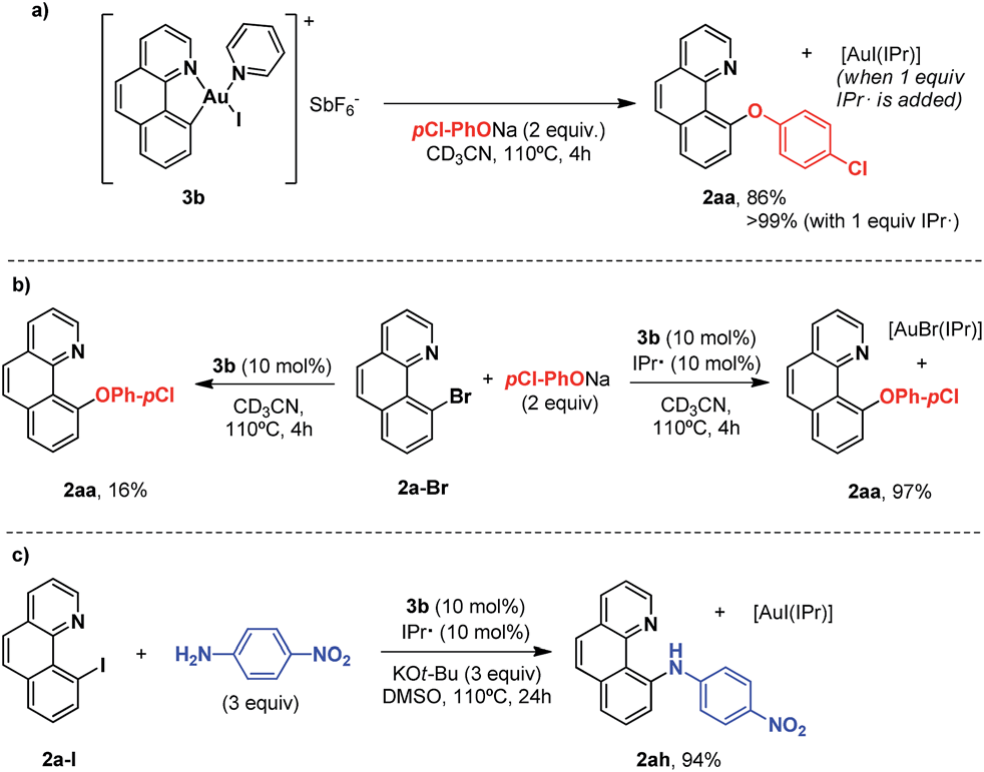
(a) Stoichiometric reactions of complex **3b** with sodium *p*-chlorophenolate in the absence and in the presence of the NHC ligand IPr. (b) Catalytic version, with and without IPr, starting from **2a-Br**, and (c) analogous coupling of *p*-nitroaniline to **2a-I** with IPr.

**Scheme 6:**
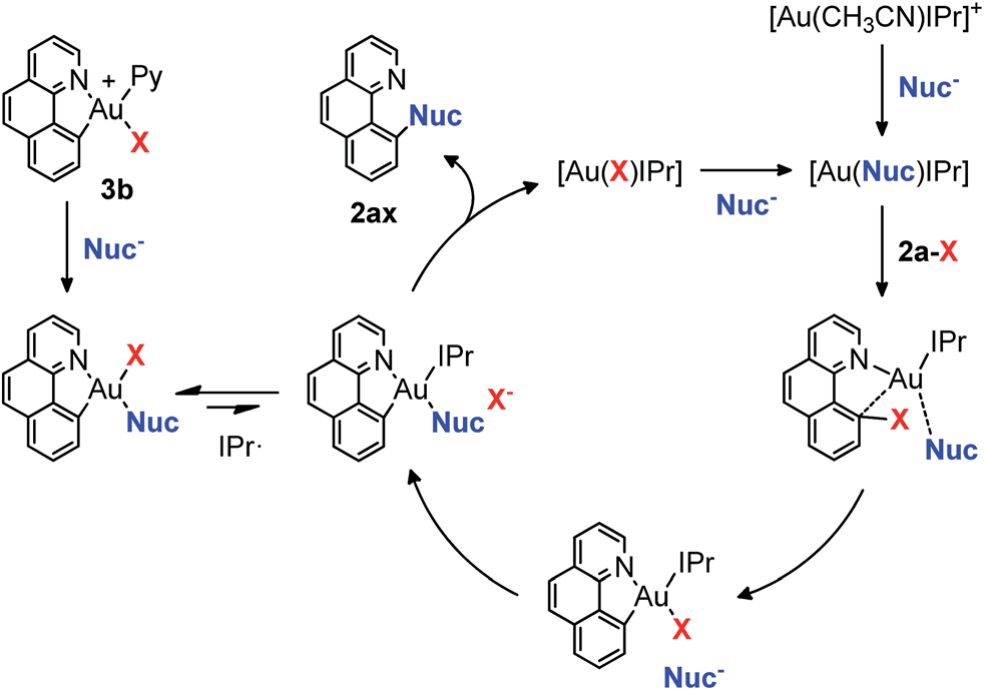
Proposed mechanism of the Au(i)-catalyzed cross-coupling reactions operating *via* aryl-Au(iii) intermediates. X = Br, I.

In our previous studies on gold(i)-catalyzed halogen exchange and C–O bond formation we proposed a mechanism operating through the general two-electron-based Au(i)/Au(iii) cycle (Scheme 6),^11^ albeit the high-valent [Au(**2a**)(IPr)X] (X = halide or phenolate) species could not be detected. In this regard we reasoned that the cationic Au(iii) complex **3b** might be helpful in trying to unveil the involvement of aryl-Au(iii) species in this oxidant-free transformation. First, 1 equivalent of complex **3b** was subjected to the same conditions as used for the *p*-chlorophenolate insertion with 2-(2-bromophenyl)pyridine **1a-I** and the desired 10-(4-chlorophenoxy)benzo[*h*]quinoline **2aa** was obtained in 86% yield (Scheme 5a), validating the feasibility of aryl-Au(iii) species in Au(i)-catalyzed cross couplings. Nonetheless, an analogous experiment starting from 10-bromobenzo[*h*]quinolone **2a-Br** and catalytic amounts of **3b** (10 mol%) provided almost 2 catalytic turnovers (16% yield of product **2aa**, Scheme 5b). Therefore, 10 mol% of the N-heterocyclic carbene donor ligand IPr· (IPr = 1,3-bis(diisopropylphenyl)imidazol-2-ylidene) employed in the catalytic transformations was also added, and we were pleased to find that virtually quantitative yields of **2aa** were achieved (Scheme 5b). Equal behavior was found with the *p*-nitroaniline insertion to **2a-I** (Scheme 5c), overall unequivocally substantiating the implication of aryl-Au(iii) species as competent catalytic intermediates. **3b** rapidly generates the neutral complex [Au(**2a**)(Nuc)X] (X = Br, I) in the presence of a nucleophile (Scheme 6). Then, the IPr· carbene exchanges one of the anionic ligands to form the less favored [Au(**2a**)(IPr)Nuc] intermediate, which rapidly reductively eliminates the cross-coupling product (**2ax**) and gold(i) as [Au(X)IPr].^11^ Strikingly, ^1^H-NMR inspection of the crude product proved that all the Au(i) remained in solution in this resting state form. On the other hand, attempts to isolate the [Au(**2a**)(IPr)Nuc] species generated in solution when IPr· is added were fruitless, supporting their short-lived nature in the catalytic cycle.

## Conclusions

In conclusion, we have developed the first examples of gold(i)-catalyzed C–N cross-coupling reactions in the absence of sacrificial oxidants, in parallel to the well-established Cu- and Pd-catalyzed methodologies, and extended the previously described C–O coupling catalysis with phenols to aliphatic alcohols and water. This system allows entry to different arylamine, arylamide, phenol and aryl-ether products under practical synthetic laboratory conditions, with absolute tolerance for both air and water. In either case, the acidity of the nucleophile is at the basis of the reactivity observed. Moreover, we have synthesized novel neutral and cationic C–N cyclometalated Au(iii) complexes through mild oxidative addition of a C_sp^2^_–I bond to gold(i) iodide, and presented conclusive evidence of their competence in the C–O and C–N coupling transformations. To the best of our knowledge, this represents the first example in which the intermediacy of Au(iii) species in an oxidant-free 2-electron coupling processes is demonstrated, clarifying the proposed mechanism operating *via* oxidative addition and reductive elimination steps. Future work is directed towards investigating other suitable chelating groups for a more versatile system, with special interest in removable directing groups.
