# Supplementary material for: Au(iii)-aryl intermediates in oxidant-free C–N and C–O cross-coupling catalysis
Source: Chem Sci. 2016 Sep 23;8(2):946–52. doi: 10.1039/c6sc03699f (PMC5452266; doi:10.1039/c6sc03699f)
Supplement: Supplementary file 1 [file SC-008-C6SC03699F-s001.pdf]

Electronic Supplementary Material (ESI) for Chemical Science.
This journal is © The Royal Society of Chemistry 2016

Supplementary Information

Au(III)-Aryl Intermediates in Oxidant-Free C-N and C-O Cross-Coupling Catalysis

Jordi Serra,[a] Teodor Parella,[b] Xavi Ribas[a,*]

[a] *QBIS Group, Institut de Química Computacional i Catàlisi (IQCC) and Departament de Química, Universitat de Girona, Campus Montilivi, Girona, E-17071, Catalonia, Spain.*
[b] *Servei de RMN, Facultat de Ciències, Universitat Autònoma de Barcelona, Campus UAB, Bellaterra E08193, Catalonia, Spain.*

Email: xavi.ribas@udg.edu

CONTENTS

1. Materials and Methods S2

1.1. Materials, methods and instrumentation S2

1.2. Synthesis of substrates and catalysts S2

1.3. Synthesis of gold(III) complexes **3a** and **3b** S3

1.4. General procedure for the coupling of *p*-chlorophenolate and sodium alkoxides to substrates **1a-Br** and **2a-Br** S4

1.5. General procedure for the C-N bond formation with aryl iodides **1a-I** and **2a-I**. S6

1.6. Reactivity of **1a-I** and AuI in the presence of bases and *p*-chlorophenolate S10

2. Supplementary Figures S12

3. X-Ray Crystallography Data S113

4. References S117

1. Materials and methods

1.1. Materials and Instrumentation

Reagents and solvents used were commercially available reagent quality unless indicated otherwise. Solvents were purchased from SDS-Carlo Erba and Scharlab and were purified and dried by passing through an activated alumina purification system (MBraun SPS-800). Preparation and handling of air-sensitive materials was carried out in a N_2 drybox (JACOMEX) with O_2 and H_2O concentrations < 1.0 ppm. NMR data were collected on a BRUKER 400 or 300 AVANCE spectrometer in the corresponding deuterated solvent, and ^1H and $^{13}C\{^1H\}$ experiments were recorded under routine conditions. ESI-MS experiments were collected and analyzed on a Bruker Daltonics Esquire 6000 spectrometer with acetonitrile or acetonitrile/water (80:20) as solvent. High resolution mass spectra (HRMS) were recorded on a Bruker MicrOTOF-Q II TM instrument using ESI or Cryospray ionization sources with acetonitrile as solvent by Dr. Laura Gómez of Serveis Tècnics of the University of Girona.

1.2. Synthesis of substrates and catalysts

The cationic NHC-Au(I) catalyst $[Au(NCMe)IPr]SbF_6$ was prepared following a literature procedure.[1]

2-(2-bromophenyl)-pyridine (**1a-Br**) was synthesized as previously described by Pd-catalyzed cross-coupling of 2-bromopyridine and 2-bromophenylboronic acid.[2] Derivative 2-(2-iodophenyl)-pyridine (**1a-I**) was prepared following a procedure found in the literature.[3]

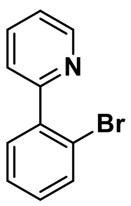

1a-Br: Spectral data are in accordance with those reported in the literature. **^{1}H-NMR** ($CDCl_3$, 400 MHz) δ, ppm: 8.71 (dq, *J* = 4.8, 2.0, 0.8 Hz, 1H, H^a), 7.76 (td, *J* = 7.6, 1.6 Hz, 1H, H^c), 7.67 (dd, *J* = 8.0, 1.2 Hz, 1H, H^h), 7.60 (dt, *J* = 8.0, 1.2 Hz, 1H, H^d), 7.54 (dd, *J* = 8.0, 1.6 Hz, 1H, H^e), 7.41 (td, *J* = 7.6, 1.2 Hz, 1H, H^g), 7.27 (m, 2H, H^b, H^f); **HRMS (ESI)** (CH_3CN, m/z): calcd for $C_{11}H_8BrN$ [M + H^+] 233.9913, found: 233.9918.

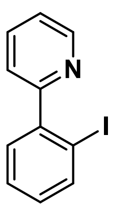

1a-I: Spectral data are in accordance with those reported in the literature.[4] **^{1}H-NMR** ($CDCl_3$, 400 MHz) δ, ppm: 8.71 (dq, *J* = 4.8, 2.0, 1.2 Hz, 1H, H^a), 7.97 (ddd, *J* = 8.0, 0.8, 0.4 Hz, 1H, H^e), 7.77 (td, *J* = 7.6, 1.6 Hz, 1H, H^c), 7.51 (dt, *J* = 8.0, 0.8 Hz, 1H, H^d), 7.44 (m, 2H, H^g and H^h), 7.31 (ddd, *J* = 4.8, 2.8, 1.2 Hz, 1H, H^b), 7.09 (ddd, *J* = 8.0, 2.4, 1.2 Hz, 1H, H^f); **HRMS (ESI)** (CH_3CN, m/z): calcd for $C_{11}H_8NI$ [M + H^+] 281.9774, found: 281.9776.

10-Bromobenzo[*h*]quinolone (**2a-Br**) was prepared according to the literature procedure starting from benzo[*h*]quinolone.[5] Derivative 10-Iodobenzo[*h*]quinolone (**2a-I**) was synthesized from **2a-Br** following Buchwald's copper-catalyzed iodination of aryl bromides.[2]

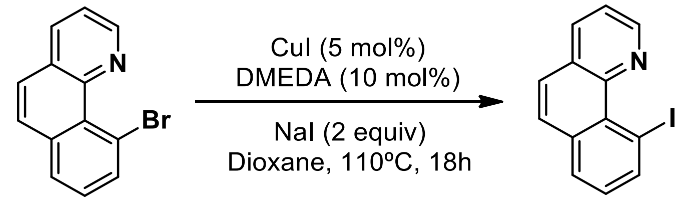

2a-Br **2a-I**

A vial was charged with **2a-Br** (516 mg, 2 mmol) and NaI (600 mg, 4 mmol) and entered in an inert-atmosphere glovebox. There CuI (19.0 mg, 0.1 mmol, 5 mol%), *N,N'*-Dimethylethylenediamine (22 µL, 0.2 mmol) and dry dioxane (1 mL) were added. The vial was capped and the reaction mixture was stirred at 110 °C for 18h. The resulting mixture was purified by column chromatography on silica using a solvent mixture of hexane-ethyl acetate 9:1. The desired product was obtained as a white solid (407 mg, 67% isolated yield).

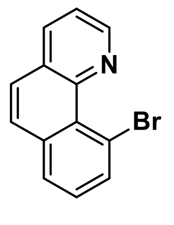

2a-Br: Spectral data are in accordance with those reported in the literature. **^{1}H-NMR** ($CDCl_3$, 400 MHz) δ, ppm: 9.11 (dd, *J* = 4.4, 2.0 Hz, 1H, H^a), 8.18 (dd, *J* = 8.0, 2.0 Hz, 1H, H^c), 8.10 (dd, *J* = 7.6, 1.2 Hz, 1H, H^h), 7.88 (dd, *J* = 8.0, 0.8 Hz, 1H, H^f), 7.75 (dd, *J* = 8.8 Hz, 2H, H^d+H^e), 7.57 (dd, *J* = 8.0, 4.4 Hz, 1H, H^b), 7.47 (t, *J* = 8.0 Hz, 1H, H^g); **HRMS (ESI)** (CH_3CN, m/z): calcd for $C_{13}H_8BrN$ [M + H^+] 257.9913, found: 257.9910.

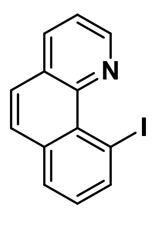

2a-I: **^{1}H-NMR** ($CDCl_3$, 400 MHz) δ, ppm: 9.11 (dd, *J* = 4.4, 2.0 Hz, 1H, H^a), 8.55 (dd, *J* = 7.6, 1.2 Hz, 1H, H^h), 8.17 (dd, *J* = 8.0, 2.0 Hz, 1H, H^c), 7.92 (dd, *J* = 8.0, 1.2 Hz, 1H, H^f), 7.74 (dd, *J* = 8.8 Hz, 2H, H^d+H^e), 7.58 (dd, *J* = 8.0, 4.4 Hz, 1H, H^b), 7.28 (t, *J* = 8.0 Hz, 1H, H^g); **^{13}C{1H}-NMR** ($CDCl_3$, 100 MHz) δ, ppm: 145.85 (C1), 144.52 (C13), 143.41 (C10), 135.58 (C3), 135.20 (C7), 129.22 (C8), 129.14 (C12), 128.45 (C9), 128.15 (C6), 126.80 (C4), 126.18 (C5), 122.17 (C2), 88.85 (C11); **HRMS (ESI)** (CH_3CN, m/z): calcd for $C_{13}H_8IN$ [M + H^+] 305.9774, found: 305.9771.

1.3. Synthesis of the Gold(III) complexes 3a and 3b

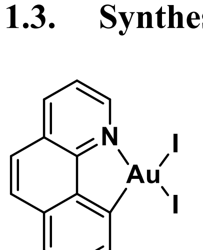

To a mixture of gold iodide (65.2 mg, 0.2 mmol) and 10-Iodobenzo[*h*]quinolone (**2a-I**) was added toluene (1 mL) at room temperature and stirred at 60ºC for 18h. The resulting suspension was then dried under vacuum and washed with cold DCM to yield **3a** as a red powder (120.8 mg, 96%). **^{1}H-NMR** ($CDCl_3$, 400 MHz, 323K) δ, ppm: 10.36 (dd, *J* = 5.6, 1.2 Hz, 1H, H^a), 8.93 (dd, *J* = 8.0, 0.8 Hz, 1H, H^h), 8.55 (dd, *J* = 8.0, 0.4 Hz, 1H, H^c), 7.88 (d, *J* = 8.8 Hz, 1H, H^e), 7.87 (d, *J* = 8.0 Hz, 1H, H^f), 7.79 (dd, *J* = 8.0, 5.6 Hz, 1H, H^b), 7.71 (d, *J* = 8.7 Hz, 1H, H^d), 7.59 (t, *J* = 8.0 Hz, 1H, H^g); **^{13}C{1H}-NMR** ($CDCl_3$, 100 MHz, 323K) δ, ppm: 154.81 (C13), 153.63 (C11), 150.00 (C1), 140.64 (C12), 140.26 (C3), 135.88 (C7), 134.52 (C10), 131.44 (C9), 130.38 (C6), 129.22 (C4), 126.33 (C8), 124.06 (C5), 122.60 (C2).

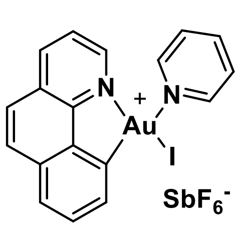

3b: Complex **3a** (25.2 mg, 0.04 mmol) and $AgSbF_6$ (13.9 mg, 0.04 mmol) were suspended in acetonitrile (1 mL) and pyridine (3.5 mg, 0.044 mmol) was added at room temperature. After stirring for <1 min at room temperature, the resulting orange mixture was filtered through *Celite*®, dried under vacuum and washed with hexanes (3 x 2 mL) to give the desired complex as a yellow solid (29.7 mg, 91%). **^{1}H-NMR** (CD_3CN, 400 MHz, 248K) δ, ppm: 8.89 (m, 3H, H^h+H^i), 8.82 (m, 1H, H^c), 8.33 (tt, *J* = 8.0, 1.6 Hz, 1H, H^k), 7.98 (m, 4H, H^f+H^e+H^j), 7.91 (d, *J* = 8.8 Hz, 1H, H^d), 7.75 (m, 2H, H^a+H^b), 7.49 (t, *J* = 8.0 Hz, 1H, H^g); **^{13}C{1H}-NMR** (CD_3CN, 100 MHz, 248K) δ, ppm: 152.23 (C13), 150.55 (C14), 145.66 (C1), 142.74 (C3), 142.62 (C16), 139.50 (C11), 139.33 (C12), 138.56 (C10), 136.21 (C7), 131.88 (C9), 130.32 (C6), 129.32 (C4), 128.80 (C15), 127.74 (C8), 125.29 (C5), 123.59 (C2); **HRMS (ESI)** (CH_3CN, m/z): calcd for $C_{18}H_{13}AuIN_2$ [M^+] 580.9783, found: 580.9782.

1.4. General procedure for the coupling of *p*-chlorophenolate and sodium alkoxides to substrates 1a-Br and 2a-Br

Substrates **1a-Br** or **2a-Br** (0.02 mmol), [Au(NCMe)IPr]SbF_6 (2 μmol, 10 mol%) and *p*-chlorophenolate or sodium alkoxide (2 equivalents, 0.04 mmol) were weighed using a precision balance (accuracy: +/-0.01 mg) into a glass vial, and dissolved in CD_3CN for *p*-chlorophenolate or the protic solvent of choice for the other alkoxides. The vial was sealed and the mixture stirred at 110ºC. After the required reaction time, the crude was allowed to reach room temperature and, if needed, solvent evaporated under vacuum. The residue was re-dissolved in $CDCl_3$ and a standard solution of 1,3,5-trimethoxybenzene (20mM, 40 μL) was added. The products in the crude reaction mixture were identified by ^{1}H-NMR and ESI-HRMS.

<u>*Product isolation*</u>: The same synthetic protocol was followed, in this case employing 0.2 mmol substrate **1a-Br** or **2a-Br**, 0.02 mmol [Au(NCMe)IPr]SbF_6 and 0.4 mmol alkoxide in 0.8 mL of solvent. After solvent evaporation the residue was subjected to column chromatography on silica gel (eluent: hexanes/EtOAc).

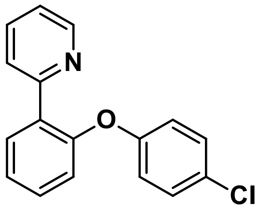

1aa: Column chromatography on silica gel (hexane-ethyl acetate 7:3) provided a colorless oil (41.0 mg, 73% isolated yield). Spectroscopic data are consistent with literature reports.[4] **^{1}H-NMR** ($CDCl_3$, 400 MHz) δ, ppm: 8.67 (dq, *J* = 4.8, 2.0, 0.8 Hz, 1H, H^a), 7.90 (dd, *J* = 7.6, 1.6 Hz, 1H, H^e), 7.77 (dt, *J* = 8.0, 1.2 Hz, 1H, H^d), 7.65 (ddd, *J* = 8.0, 7.6, 2.0 Hz, 1H, H^c), 7.38 (ddd, *J* = 7.6, 7.4, 1.6 Hz, 1H, H^g), 7.29 (td, *J* = 8., 7.6, 1.2 Hz, 1H, H^f), 7.20 (m, 2H, H^b+H^i), 7.01 (dd, *J* = 7.4, 1.2 Hz, 1H, H^h), 6.86 (d, *J* = 8.8 Hz, 2H, H^j); **HRMS (ESI)** (CH_3CN, m/z): calcd for $C_{17}H_{12}NClO$ [M + H+] 282.0680, found: 282.0682.

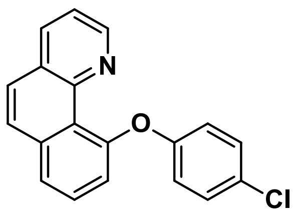

2aa: Column chromatography on silica gel (hexane-ethyl acetate 4:1) provided a pale yellow solid (47.6 mg, 78% isolated yield). **^{1}H-NMR** ($CDCl_3$, 400 MHz) δ, ppm: 8.80 (dd, *J* = 4.4, 2.0 Hz, 1H, H^a), 8.11 (dd, *J* = 8.0, 2.0 Hz, 1H, H^c), 7.84 (d, *J* = 8.8 Hz, 1H, H^e), 7.83 (dd, *J* = 8.0, 1.2 Hz, 1H, H^h), 7.70 (d, *J* = 8.8 Hz, 1H, H^d), 7.69 (t, *J* = 8.0 Hz, 1H, H^g), 7.43 (dd, *J* = 8.0, 1.6 Hz, 1H, H^f), 7.41 (dd, *J* = 8.0, 4.4 Hz, 1H, H^b), 7.15 (d, *J* = 9.2, 1.6 Hz, 2H, H^j), 6.82 (d, *J* = 9.2 Hz, 2H, H^i); **^{13}C{1H}-NMR** ($CDCl_3$, 100 MHz) δ, ppm: 158.49 (C14), 153.21 (C11), 148.84 (C1), 145.79 (C13), 136.60 (C7), 135.38 (C3), 129.09 (C16), 128.51 (C5), 127.94 (C6), 127.12 (C4), 126.67 (C9), 126.03 (C17), 125.80 (C10), 124.40 (C12), 122.31 (C8), 121.43 (C2), 117.65 (C15); **HRMS (ESI)** (CH_3CN, m/z): calcd for $C_{19}H_{12}NOCl$ [M + H^+] 306.0680, found: 306.0677.

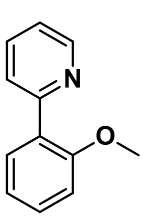

1ab: Column chromatography on silica gel (hexane-ethyl acetate 1:1) afforded a colorless oil (30.7 mg, 83% isolated yield). Spectral data match literature reports.[6] **^{1}H-NMR** ($CDCl_3$, 400 MHz) δ, ppm: 8.70 (ddd, *J* = 4.8, 2.0, 1.2 Hz, 1H, H^a), 7.81 (dt, *J* = 8.0, 1.2 Hz, 1H, H^d), 7.76 (dd, *J* = 7.6, 2.0 Hz, 1H, H^e), 7.70 (td, *J* = 7.6, 2.0 Hz, 1H, H^c), 7.37 (ddd, *J* = 8.8, 7.6, 2.0 Hz, 1H, H^g), 7.20 (ddd, *J* = 7.6, 4.8, 1.2 Hz, 1H, H^b), 7.08 (td, *J* = 7.6, 1.2 Hz, 1H, H^f), 7.01 (dd, *J* = 8.4, 1.2 Hz, 1H, H^h), 3.86 (s, 3H, H^i); **HRMS (ESI)** (CH_3CN, m/z): calcd for $C_{12}H_{11}NO$ [M + H^+] 186.0913, found: 186.0914.

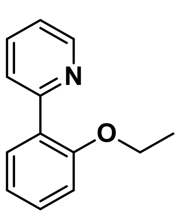

1ac: Column chromatography on silica gel (hexane-ethyl acetate 3:1) gave a yellow oil (24.3 mg, 61% isolated yield). Spectral data match previously reported data.[7] **^{1}H-NMR** ($CDCl_3$, 400 MHz) δ, ppm: 8.70 (ddd, *J* = 4.8, 2.0, 1.2 Hz, 1H, H^a), 7.90 (dt, *J* = 8.0, 1.2 Hz, 1H, H^c), 7.81 (dd, *J* = 7.6, 2.0 Hz, 1H, H^e), 7.69 (td, *J* = 8.0, 2.0 Hz, 1H, H^c), 7.34 (ddd, *J* = 8.4, 7.2, 1.6 Hz, 1H, H^g), 7.19 (ddd, *J* = 7.6, 4.8, 1.2 Hz, 1H, H^b), 7.07 (td, *J* = 7.6, 1.2 Hz, 1H, H^f), 7.01 (dd, *J* = 8.4, 1.2 Hz, 1H, H^h), 4.09 (q, *J* = 7.2 Hz, 2H, H^i), 1.39 (t, *J* = 7.2 Hz, 3H, H^j); **HRMS (ESI)** (CH_3CN, m/z): calcd for $C_{13}H_{13}NO$ [M + H^+] 200.1064, found: 200.1070.

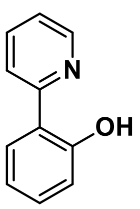

1af: Column chromatography on silica gel (hexane-ethyl acetate 9:1) afforded a yellow oil (14.4 mg, 42% isolated yield). Spectral data match literature reports.[8] **^{1}H-NMR** ($CDCl_3$, 400 MHz) δ, ppm: 14.36 (s, br, 1H, O-*H*), 8.52 (ddd, *J* = 4.8, 1.6, 0.8 Hz, 1H, H^a), 7.92 (d, *J* = 8.4 Hz, 1H, H^d), 7.84 (ddd, *J* = 8.4, 7.6, 1.6 Hz, 1H, H^c), 7.81 (dd, *J* = 8.0, 1.6 Hz, 1H, H^e), 7.31 (ddd, *J* = 8.4, 7.2, 1.6 Hz, 1H, H^g), 7.25 (ddd, *J* = 7.6, 4.8, 0.8 Hz, 1H, H^b), 7.03 (dd, *J* = 8.4, 1.2 Hz, 1H, H^h), 6.91 (ddd, *J* = 8.0, 7.2, 1.2 Hz, 1H, H^f); **HRMS (ESI)** (CH_3CN, m/z): calcd for $C_{11}H_9NO$ [M + H^+] 172.0757, found: 172.0763.

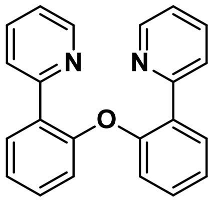

1ag: Column chromatography on silica gel (hexane-ethyl acetate 3:2) provided a white solid (13.0 mg, 20% isolated yield). **^{1}H-NMR** ($CDCl_3$, 400 MHz) δ, ppm: 8.67 (ddd, J = 4.8, 2.0, 0.8 Hz, 2H, H^a), 7.86 (dd, J = 7.6, 2.0 Hz, 2H, H^e), 7.72 (dt, J = 8.0, 2.0 Hz, 2H, H^d), 7.58 (td, J = 7.6, 2.0 Hz, 2H, H^c), 7.30 (ddd, J = 8.0, 7.2, 2.0 Hz, 2H, H^g), 7.21 (td, J = 7.6, 1.2 Hz, 2H, H^f), 7.17 (ddd, J = 7.2, 4.8, 1.2 Hz, 2H, H^b), 6.95 (dd, J = 8.0, 1.2 Hz, 2H, H^h); **^{13}C{1H}-NMR** ($CDCl_3$, 100 MHz) δ, ppm: 155.15 (C5), 154.22 (C11), 149.52 (C1), 135.84 (C3), 131.56 (C6), 131.42 (C7), 130.03 (C9), 124.74 (C4), 123.89 (C8), 122.02 (C2), 119.05 (10); **HRMS (ESI)** (CH_3CN, m/z): calcd for $C_{22}H_{16}N_2O$ [M + H^+] 325.1335, found: 325.1318.

1.5. General procedure for the C-N bond formation with 1a-I and 2a-I

For the optimization of the reaction conditions with *p*-nitroaniline, a vial was charged with **1a-I** (0.02 mmol), [Au(NCMe)IPr]SbF_6 (1.8mg, 0.002 mmol), *p*-nitroaniline and base, and 0.5 mL of deuterated solvent were added. The vial was capped and the mixture stirred at 110ºC for 24h. The reaction crude was then allowed to reach room temperature and a standard solution of 1,3,5-trimethoxybenzene (20mM, 40 µL) was added. The resulting suspension was analyzed by ^{1}H-NMR. The presence of the final C-N coupling product **1ah** was also confirmed by ESI-MS.

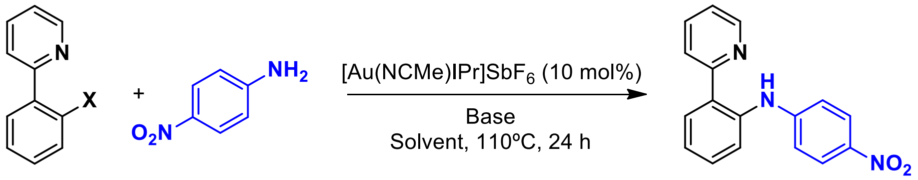

Table S1. Optimization of reaction conditions for the *p*-nitroaniline insertion reaction using 2-(2-bromophenyl)-pyridine (**1a-Br**) or 2-(2-iodophenyl)-pyridine (**1a-I**)

Entry	X	Solvent	Base	Yield 1a (%)^a
1	Br	CH_3CN	K_3PO_4 (3)	**0**
2	Br	CH_3CN	CsF (3)	**0**
3	Br	CH_3CN	KO*t*-Bu (3)	**0**
4	Br	CH_3CN	CsF (5)	**0**^{b}
5	Br	Toluene	CsF (5)	**0**^{b}
6	Br	Dioxane	CsF (5)	**4**^{b,c}
7	Br	DMSO	CsF (5)	**24**^{b}
8	Br	DMSO	KO*t*-Bu (5)	**39**^{b}
9	I	DMSO	CsF (5)	**44**^{b}
10	I	DMSO	KO*t*-Bu (5)	**93**^{b}
11	I	DMSO	KO*t*-Bu (3)	**95 (92)**^{d}

^{a}Yields as determined by ^{1}H-NMR using 1,3,5-trimethoxybenzene as an internal standard. ^{b}[*p*-NO_2aniline] = 100 mM; ^{c}After the required reaction time, solvent was removed under vacuum and $CDCl_3$ (0.6 mL) was added for ^{1}H-NMR analysis. ^{d}T = 100ºC.

Following the optimized procedure described above (Table S1), different amines and amides were screened. General conditions: [**2-(2-iodophenyl)pyridine**] = 20 mM, [Au(I)] = 2 mM, [nucleophile] = 60 mM, [KO*t*-Bu] = 60 mM, 0.5 mL DMSO-d_6, 110ºC, 24h.

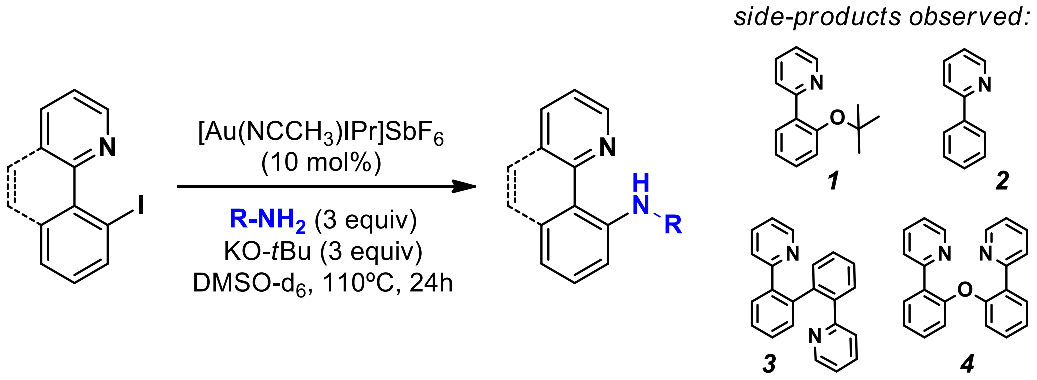

Table S2. Outcomes of the C-N cross-coupling reactions using **1a-I** and **2a-I**.

Entry	Nucleophile	T (ºC)	Yield [%][a]	Conversion [%][a]	Side-products (% yield)[a]
1	*p*-Nitroaniline	100	92	100	***1*** (trace)
2[b]	*p*-Nitroaniline	100	93	100	-
3	2-Hydroxypyridine	100	95	100	-
4	Imidazole	100	93	100	-
5	Benzamide	110	59	66	***4*** (trace)
6[c]	Cyclohexylamine	110	72	81	***3*** (5)
7[c]	Propylamine	110	79	84	***4*** (trace)
8[c]	Piperidine	110	51	67	***2*** (4); ***3*** (6)
9	Aniline	110	50	100	***2*** (8); ***3*** (15); ***4*** (9)
10	*p*-Anisidine	110	17	44	***1*** (12); ***3*** (7)
11	Diethylamine	110	8	19	-

[a]Average yields and conversions of at least two experiments as determined by ^{1}H-NMR using 1,3,5-trimethoxybenzene as internal standard. [b]10-Iodobenzo[*h*]quinolone (**2a-I**) as substrate. [c]Average GC and ^{1}H-NMR yields and conversions using 1,3,5-trimethoxybenzene as an internal standard.

Product isolation: A larger scale reaction was also performed: (0.2 mmol substrate **1a-I** or **2a-I**; 0.02 mmol [Au(NCMe)IPr]SbF$_6$; 0.6 mmol nucleophile; 0.6 mmol KO-*t*Bu) in 0.8 mL of DMSO. After cooling down to room temperature the residue was subjected to column chromatography on silica gel (eluent: hexanes/EtOAc).

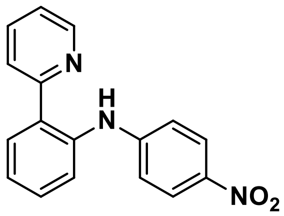

1ah: Column chromatography on silica gel (hexanes-ethyl acetate 3:2) provided a yellow solid (43.8 mg, 79% isolated yield). ^{1}**H-NMR** ($CDCl_3$, 400 MHz) δ, ppm: 10.89 (s, 1H, H^i), 8.66 (dq, J = 4.8, 2.0, 0.8

Hz, 1H, H^a), 8.11 (d, J = 8.8 Hz, 2H, H^k), 7.83 (ddd, J = 8.0, 7.6, 2.0 Hz, 1H, H^c), 7.72 (dt, J = 8.0, 1.2 Hz, 1H, H^d), 7.67 (dd, J = 7.6, 1.2 Hz, 1H, H^h), 7.60 (dd, J = 8.0, 0.8 Hz, 1H, H^e), 7.39 (td, J = 7.6, 1.6 Hz, 1H, H^g), 7.28 (ddd, J = 7.6, 4.8, 1.2 Hz, 1H, H^b), 7.15 (td, J = 7.6, 1.2 Hz, 1H, H^f), 7.11 (d, J = 8.8 Hz, 2H, H^j); **^{13}C{1H}-NMR** ($CDCl_3$, 100 MHz) δ, ppm: 158.21 (C5), 149.41 (C12), 147.66 (C1), 139.76 (C15), 139.42 (C11), 137.56 (C3), 130.21 (C10), 129.69 (C9), 127.85 (C6), 126.10 (C14), 123.03 (C4), 122.82 (C8), 121.95 (C2), 119.97 (C17), 115.10 (C13); **HRMS (ESI)** (CH_3CN, m/z): calcd for $C_{17}H_{13}N_3O_2$ [M + H^+] 291.1075, found: 291.1081.

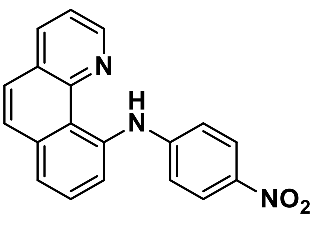

2ah: Column chromatography on silica gel (hexane-ethyl acetate 3:2) afforded an orange solid (52.9 mg, 84% isolated yield). **^{1}H-NMR** ($CDCl_3$, 400 MHz) δ, ppm: 13.65 (s, br, 1H, H^i), 8.95 (dd, J = 4.4, 2.0 Hz, 1H, H^a), 8.26 (dd, J = 8.0, 2.0 Hz, 1H, H^c), 8.21 (d, J = 9.2 Hz, 2H, H^k), 7.84 (dd, J = 8.0, 1.2 Hz, 1H, H^h), 7.83 (d, J = 8.8 Hz, 1H, H^e), 7.69 (d, J = 8.8 Hz, 1H, H^d), 7.65 (t, J = 8.0 Hz, 1H, H^g), 7.57 (dd, J = 8.0, 4.4 Hz, 1H, H^b), 7.54 (dd, J = 8.0, 0.8 Hz, 1H, H^f), 7.45 (d, J = 9.2 Hz, 2H, H^j); **^{13}C{1H}-NMR** ($CDCl_3$, 100 MHz) δ, ppm: 149.44 (C14), 148.18 (C13), 146.11 (C1), 141.88 (C11), 140.41 (C17), 136.35 (C3), 136.02 (C7), 129.29 (C6), 128.33 (C9), 127.47 (C4), 126.05 (C16), 125.64 (C5), 121.13 (C8), 120.79 (C2), 118.00 (C12), 116.72 (C15), 114.35 (C10); **HRMS (ESI)** (CH_3CN, m/z): calcd for $C_{19}H_{13}N_3O_2$ [M + H^+] 316.1081, found: 316.1074

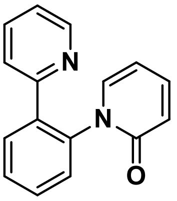

1ai: Column chromatography on silica gel (hexane-ethyl acetate 2:3) provided a colorless oil (37.2 mg, 75% isolated yield). **^{1}H-NMR** ($CDCl_3$, 400 MHz) δ, ppm: 8.63 (dq, J = 4.8, 2.0, 1.2 Hz, 1H, H^a), 8.14 (dq, J = 5.2, 2.0, 0.8 Hz, 1H, H^l), 7.89 (dd, J = 8.0, 1.6 Hz, 1H, H^e), 7.72 (dt, J = 8.0, 1.2 Hz, 1H, H^d), 7.59 (m, 2H, H^c+H^j), 7.44 (ddd, J = 8.0, 7.6, 1.6 Hz, 1H, H^g), 7.35 (td, J = 7.6, 1.2 Hz, 1H, H^f), 7.16 (dd, J = 8.0, 1.2 Hz, 1H, H^h), 7.13 (ddd, J = 7.6, 4.8, 1.2 Hz, 1H, H^b), 6.91 (ddd, J = 7.2, 5.2, 1.2 Hz, 1H, H^k), 6.77 (dt, J = 8.4, 1.2 Hz, 1H, H^i); **^{13}C{1H}-NMR** ($CDCl_3$, 100 MHz) δ, ppm: 163.68 (C12), 155.35 (C5), 153.56 (C6), 149.54 (C1), 147.87 (C16), 139.33 (C3), 135.89 (C14), 133.19 (C11), 131.39 (C7), 129.96 (C9), 125.49 (C8), 124.48 (C4), 122.50 (C10), 121.95 (C2), 118.31 (C15), 111.10 (C13); **HRMS (ESI)** (CH_3CN, m/z): calcd for $C_{16}H_{12}N_2O$ [M + H^+] 249.1022, found: 249.1022.

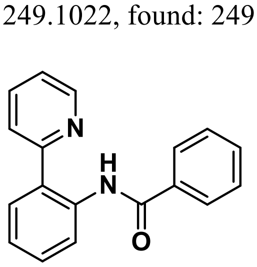

1aj: Column chromatography on silica gel (hexane-diethyl ether 50:50) afforded a white solid (24.7 mg, 45% isolated yield). **^{1}H-NMR** ($CDCl_3$, 400 MHz) δ, ppm: 8.79 (dd, J = 8.4, 1.2 Hz, 1H, H^h), 8.69 (dq, J = 4.8, 1.6, 0.8 Hz, 1H, H^a), 8.04 (m, 2H, H^j), 7.87 (ddd, J = 8.0, 7.2, 2.0 Hz, 1H, H^c), 7.81 (dt, J = 8.0, 1.2 Hz, 1H, H^d), 7.74 (dd, J = 8.0, 1.6 Hz, 1H, H^e), 7.50 (m, 4H, H^k+H^l+H^g), 7.30 (ddd, J = 7.2, 4.8, 1.2 Hz, 1H, H^b), 7.21 (ddd, J = 8.0, 7.2, 1.2 Hz, 1H, H^f); **^{13}C{1H}-NMR** ($CDCl_3$, 100 MHz) δ, ppm: 165.58 (C12), 158.40 (C5), 147.33 (C1), 138.13

(C11), 137.87 (C3), 135.79 (C13), 131.50 (C16), 130.28 (C7), 128.77 (C9), 128.60 (C15), 127.39 (C14), 125.64 (C6), 123.56 (C8), 123.02 (C4), 121.98 (C10), 121.96 (C2); **HRMS (ESI)** (CH_3CN, m/z): calcd for $C_{18}H_{14}N_2O$ [M + Na^+] 297.0998, found: 297.1008.

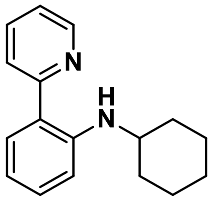

1ak: Column chromatography on silica gel (hexane-ethyl acetate 3:2) afforded a yellow oil (34.4 mg, 70% isolated yield). **^{1}H-NMR** ($CDCl_3$, 400 MHz) δ, ppm: 8.57 (dq, *J* = 4.8, 2.0, 0.8 Hz, 1H, H^a), 8.18 (s, 1H, H^i), 7.73 (ddd, *J* = 8.0, 7.2, 2.0 Hz, 1H, H^c), 7.66 (dt, *J* = 8.0, 1.2 Hz, 1H, H^d), 7.52 (dd, *J* = 7.6, 1.6 Hz, 1H, H^e), 7.23 (ddd, *J* = 8.4, 7.2, 1.6 Hz, 1H, H^g), 7.14 (ddd, *J* = 7.2, 4.8, 1.2 Hz, 1H, H^b), 6.77 (d, *J* = 8.4 Hz, 1H, H^h), 6.66 (ddd, *J* = 7.6, 7.2, 0.4 Hz, 1H, H^f), 3.41 (m, 1H, H^j), 2.04 (m, 2H, H^k), 1.76 (m, 2H, H^m), 1.60 (m, 1H, H^o), 1.36 (m, 5H, H^l+H^n+H^p); **^{13}C{1H}-NMR** ($CDCl_3$, 100 MHz) δ, ppm: 159.91 (C5), 147.32 (C1), 146.90 (C11), 136.73 (C3), 130.20 (C9), 129.69 (C7), 122.35 (C4), 121.04 (C6), 120.54 (C2), 115.03 (C8), 111.94 (C10), 50.80 (C12), 32.95 (C13), 26.11 (C15), 24.70 (C14); **HRMS (ESI)** (CH_3CN, m/z): calcd for $C_{17}H_{20}N_2$ [M + H^+] 253.1699, found: 253.1696.

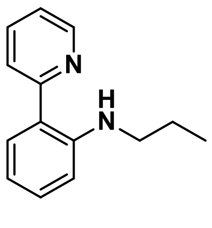

1al: Column chromatography on silica gel (hexane-ethyl acetate 3:2) gave a pale yellow oil (30.1 mg, 71% isolated yield). **^{1}H-NMR** ($CDCl_3$, 400 MHz) δ, ppm: 8.58 (dq, *J* = 4.8, 2.0, 1.2 Hz, 1H, H^a), 8.12 (s, br, 1H, H^i), 7.74 (ddd, *J* = 8.4, 7.2, 2.0 Hz, 1H, H^c), 7.67 (dt, *J* = 8.4, 1.2 Hz, 1H, H^d), 7.53 (dd, *J* = 8.0, 1.6 Hz, 1H, H^e), 7.26 (ddd, *J* = 8.4, 7.6, 1.6 Hz, 1H, H^g), 7.15 (ddd, *J* = 7.2, 4.8, 1.2 Hz, 1H, H^b), 6.75 (dd, *J* = 8.4, 1.2 Hz, 1H, H^h), 6.70 (ddd, *J* = 8.0, 7.6, 1.2 Hz, 1H, H^f), 3.17 (t, *J* = 7.2 Hz, 2H, H^j), 1.71 (sext., *J* = 7.2 Hz, 2H, H^k), 1.03 (t, *J* = 7.2 Hz, 3H, H^l); **^{13}C{1H}-NMR** ($CDCl_3$, 100 MHz) δ, ppm: 159.86 (C5), 147.90 (C11), 147.38 (C1), 136.81 (C3), 130.32 (C9), 129.44 (C7), 122.34 (C4), 121.15 (C6), 120.65 (C2), 115.39 (C8), 111.35 (C10), 45.19 (C12), 22.53 (C13), 11.86 (C14); **HRMS (ESI)** (CH_3CN, m/z): calcd for $C_{14}H_{16}N_2$ [M + H^+] 213.1386, found: 213.1381

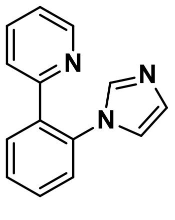

1am: Column chromatography on silica gel (hexane-ethyl acetate 1:4) provided a colorless liquid, which was subsequently washed with brine (5 x 3mL) in order to remove residual DMSO. A white solid was obtained (33.6 mg, 76% isolated yield). **^{1}H-NMR** ($CDCl_3$, 400 MHz) δ, ppm: 8.65 (dq, *J* = 4.8, 2.0, 0.8 Hz, 1H, H^a), 7.79 (dd, *J* = 7.2, 2.0 Hz, 1H, H^e), 7.54 (m, 3H, H^c+H^g+H^f), 7.44 (s, br, 1H, H^k), 7.39 (dd, *J* = 7.2, 2.0 Hz, 1H, H^h), 7.20 (ddd, *J* = 7.6, 4.8, 1.2 Hz, 1H, H^b), 7.06 (s, br, 1H, H^j), 6.89 (s, br, 1H, H^i), 6.83 (dt, *J* = 8.0, 0.8 Hz, 1H, H^d); **^{13}C{1H}-NMR** ($CDCl_3$, 100 MHz) δ, ppm: 155.69 (C5), 149.93 (C1), 137.53 (C14), 136.52 (C6), 136.42 (C3), 135.20 (C11), 131.45 (C7), 129.65 (C13), 129.59 (C8), 128.92 (C9), 126.36 (C10), 123.13 (C4), 122.51 (C2), 120.61 (C12); **HRMS (ESI)** (CH_3CN, m/z): calcd for $C_{14}H_{11}N_3$ [M + H^+] 222.1026, found: 222.1020.

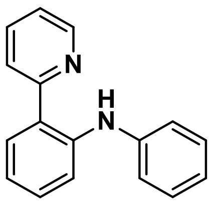

1an: Column chromatography on silica gel (hexane-ethyl acetate 9:1) provided a yellow oil (20.2 mg, 41% isolated yield). Spectroscopic data are consistent with literature reports.[9] **^{1}H-NMR** ($CDCl_3$, 400 MHz) δ, ppm: 10.22 (s, 1H, H^i), 8.63 (dq, *J* = 4.8, 1.6, 0.8 Hz, 1H, H^a), 7.78 (ddd, *J* = 8.0, 7.2, 2.0 Hz, 1H, H^c), 7.70 (dt, *J* = 8.0, 1.2 Hz, 1H, H^d), 7.61 (dd, *J* = 8.0, 1.6 Hz, 1H, H^e or H^h), 7.47 (dd, *J* = 8.4, 1.2 Hz, 1H, H^e or H^h), 7.23 (m, 6H, H^j+H^k+H^g+H^b or H^f), 6.92 (m, 2H, H^l+H^b or H^f); **HRMS (ESI)** (CH_3CN, m/z): calcd for $C_{17}H_{14}N_2$ [M + H^+] 247.1230, found: 247.1231.

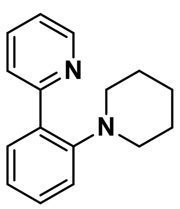

1ap: Column chromatography on silica gel (hexane-ethyl acetate 2:3) gave a colorless oil (19.0 mg, 40% isolated yield). Spectral data match literature reports.[10] **^{1}H-NMR** ($CDCl_3$, 400 MHz) δ, ppm: 8.69 (dq, *J* = 4.8, 2.0, 0.8 Hz, 1H, H^a), 8.08 (dt, *J* = 8.0, 1.2 Hz, 1H, H^d), 7.65 (ddd, *J* = 8.0, 7.6, 2.0 Hz, 1H, H^g), 7.58 (dd, *J* = 7.6, 2.0 Hz, 1H, H^e), 7.31 (ddd, *J* = 8.0, 7.6, 2.0 Hz, 1H, H^c), 7.17 (ddd, *J* = 7.6, 4.8, 1.2 Hz, 1H, H^b), 7.09 (td, *J* = 7.6, 1.2 Hz, 1H, H^f), 7.05 (dd, *J* = 8.0, 1.2 Hz, 1H, H^h), 2.79 (m, 4H, CH_2), 1.49 (m, 6H, CH_2); **HRMS (ESI)** (CH_3CN, m/z): calcd for $C_{16}H_{18}N_2$ [M + H^+] 239.1543, found: 239.1541. .

1.6. Reactivity of 1a-I and AuI in the presence of bases and *p*-chlorophenolate

Attempts to isolate a C-N cyclometalated gold(III) complex by reacting **1a-I** and AuI led in all cases to quantitative recovery of the starting substrate. We rationalize that, although the oxidative addition has an accessible barrier, as expected from the C-O and C-N coupling reactions, the ensuing complex [Au(**1a**)I_2] is thermodynamically disfavored, pulling the equilibrium back towards the reagents. Aiming at supporting this proposal, we performed the reaction in the presence of a base or sodium *p*-chlorophenolate. Thus, **1a-I** (5.6 mg, 0.02 mmol), AuI (6.5, 0.02 mmol) and KO-*t*Bu (4.5 mg, 0.04 mmol) or K_3PO_4 (8.5 mg, 0.04 mmol) or sodium *p*-chlorophenolate (6.0 mg, 0.04 mmol) were weighed into a glass vial, and dissolved in toluene. The vial was capped and the mixture stirred at 110ºC for 24h. ^{1}H-NMR analyses of the crude revealed presence of **1a-I** (90-95%) for potassium *tert*-butoxide and potassium phosphate, while in the presence of sodium *p*-chlorophenolate 60% of the **1aa** coupling product was observed. This yield could be increased up to 90% upon toluene replacement for acetonitrile (see Scheme S1).

KO-*t*Bu and K_3PO_4 cannot displace the iodides in a putative [Au(**1a**)I_2] complex, which would immediately lead to the reductive elimination and consumption of **1a-I**. On the other hand, however, the nucleophilic character of sodium *p*-chlorophenolate allows for this process occur. A catalytic performance can be likewise attained by adding IPr· carbene in the reaction mixture. Overall, this strongly suggests C_{sp2}-I bond activation by Au(I).

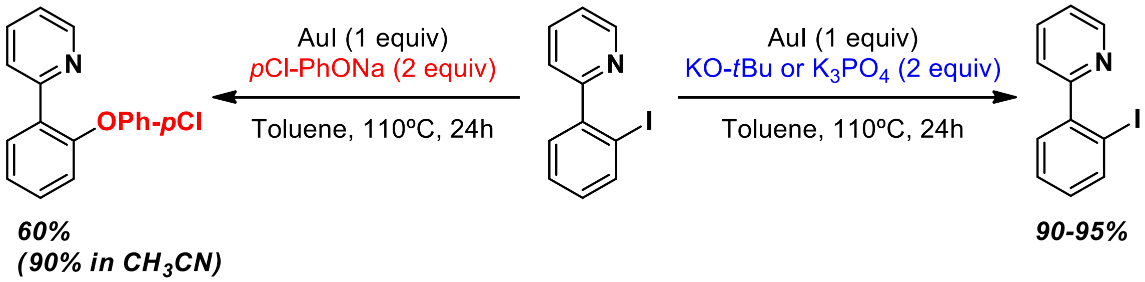

Scheme S1: Outcomes observed in the reaction between **1a-I** and AuI, when potassium *tert*-butoxide, potassium phosphate or sodium *p*-chlorophenolate are added.

2. Supplementary figures

Figure S1. a) ^{1}H-NMR spectrum of compound **1a-Br** in $CDCl_3$, 400 MHz, at 298 K; b) HRMS (ESI-MS) spectrum (*m/z*). Observed HRMS (left) with the theoretical isotope prediction (right).

a)

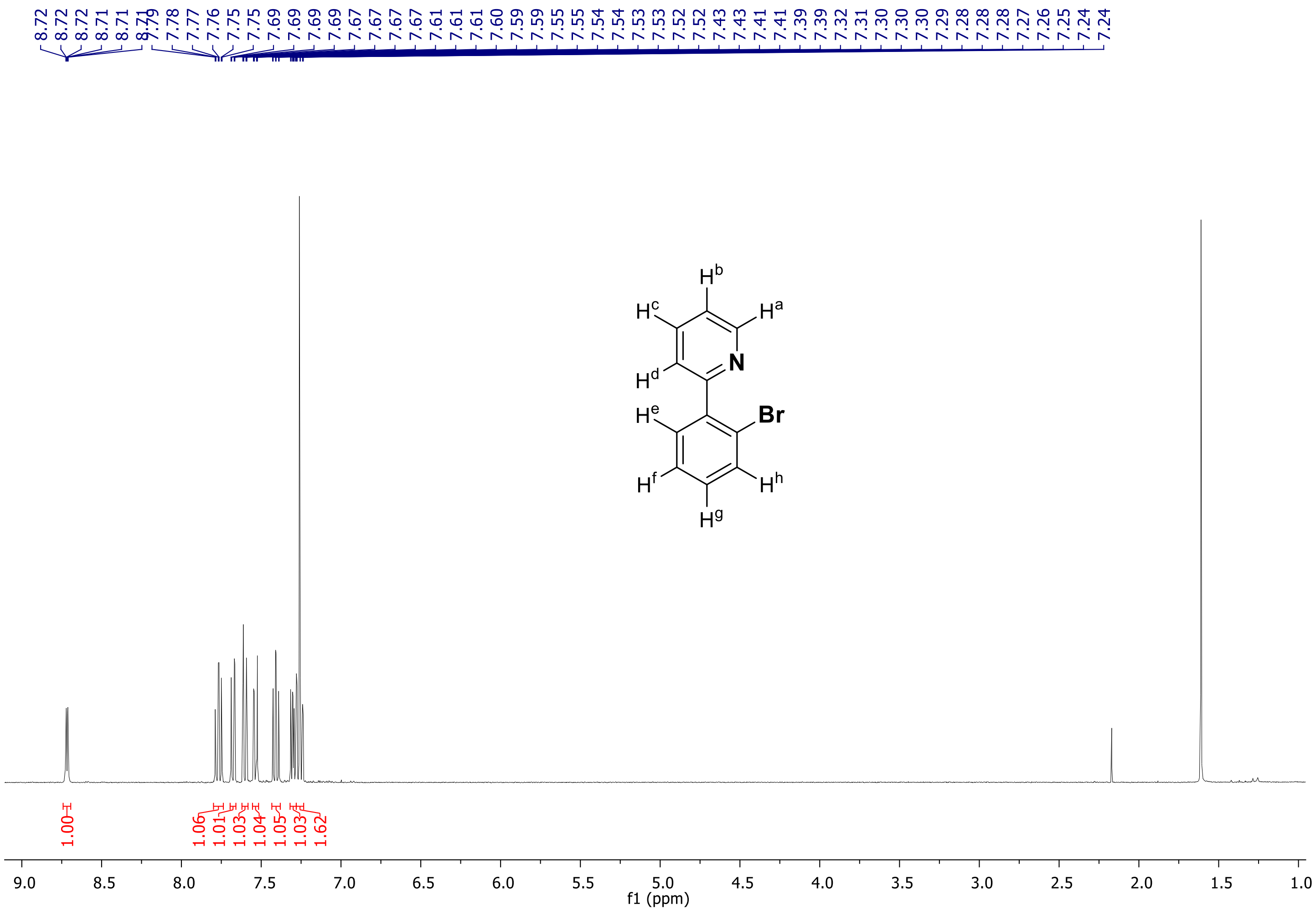

b)

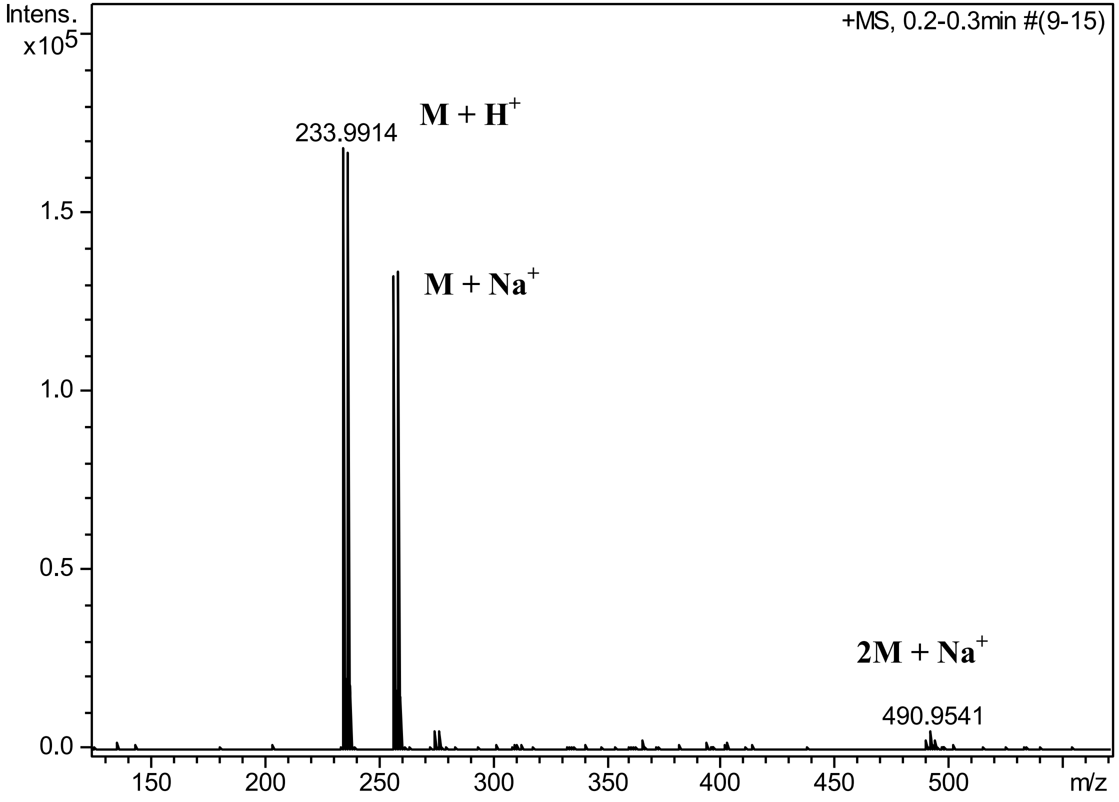

Intens.
x10^5
+MS, 0.2-0.3min #(9-15)
233.9914
M + H+
M + Na+
2M + Na+
490.9541
1.5
1.0
0.5
0.0
150
200
250
300
350
400
450
500
m/z

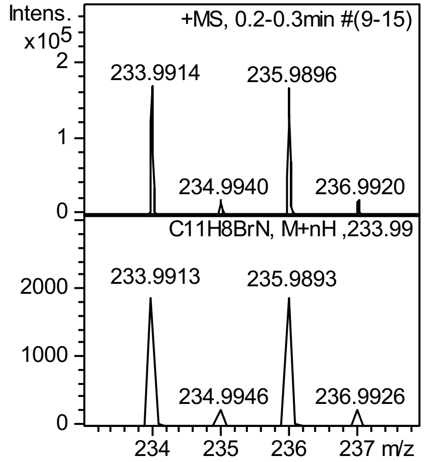

Intens.
x10^5
+MS, 0.2-0.3min #(9-15)
233.9914
235.9896
234.9940
236.9920
C11H8BrN, M+nH ,233.99
233.9913
235.9893
234.9946
236.9926
234
235
236
237 m/z

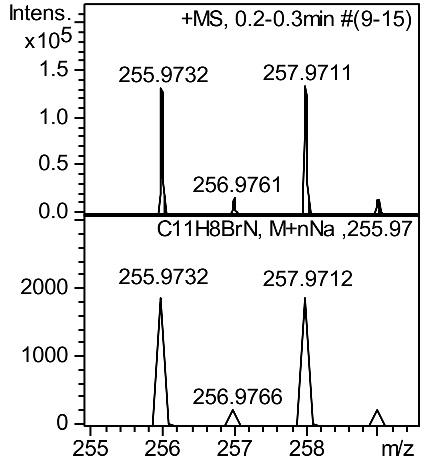

Intens.
x10^5
+MS, 0.2-0.3min #(9-15)
255.9732
257.9711
256.9761
C11H8BrN, M+nNa ,255.97
255.9732
257.9712
256.9766
255
256
257
258
m/z

Figure S2. a) ^{1}H-NMR spectrum of compound **1a-I** in $CDCl_3$, 400 MHz, at 298 K; b) HRMS (ESI-MS) spectrum (*m/z*). Observed HRMS (left) with the theoretical isotope prediction (right).

a)

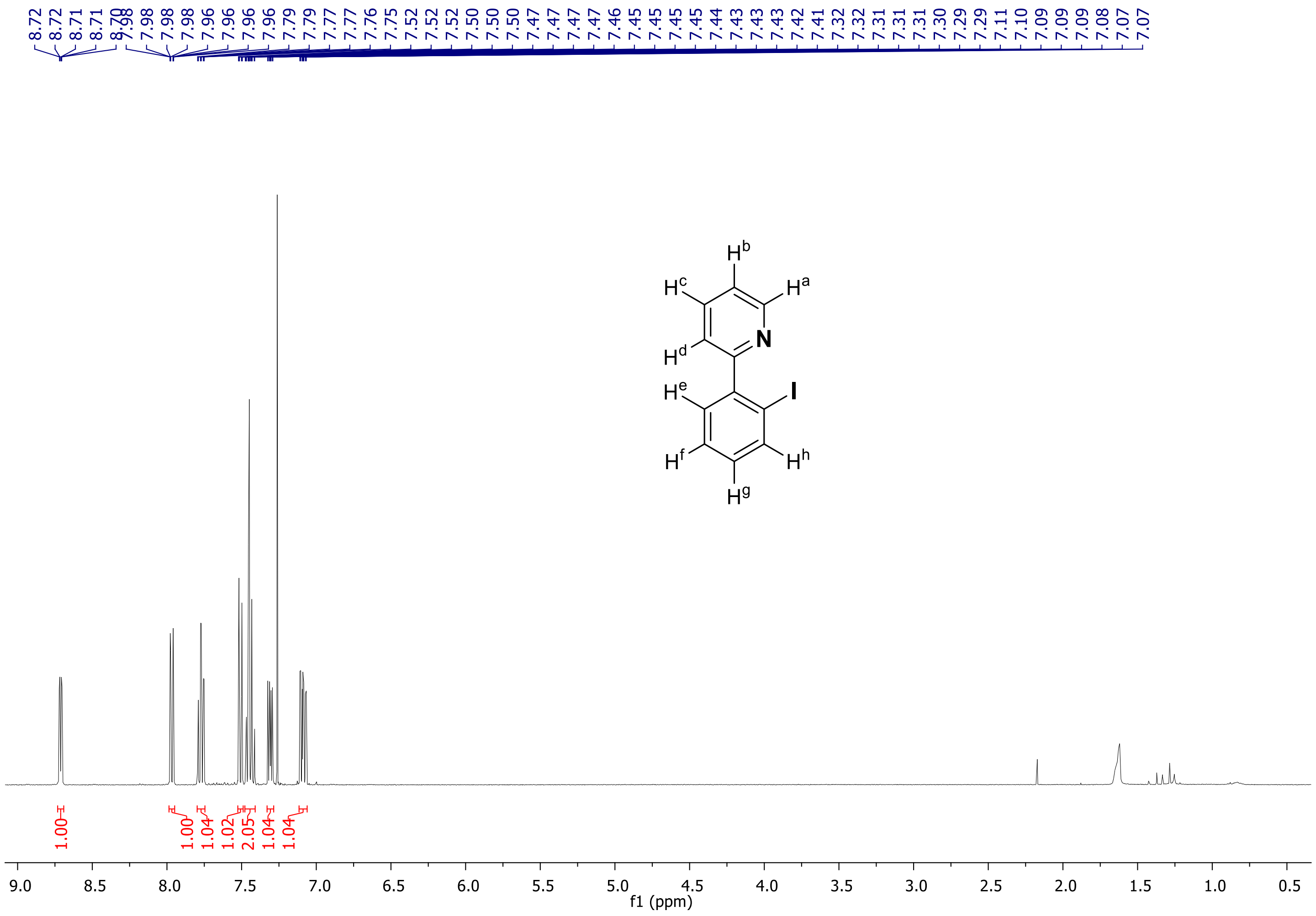

b)

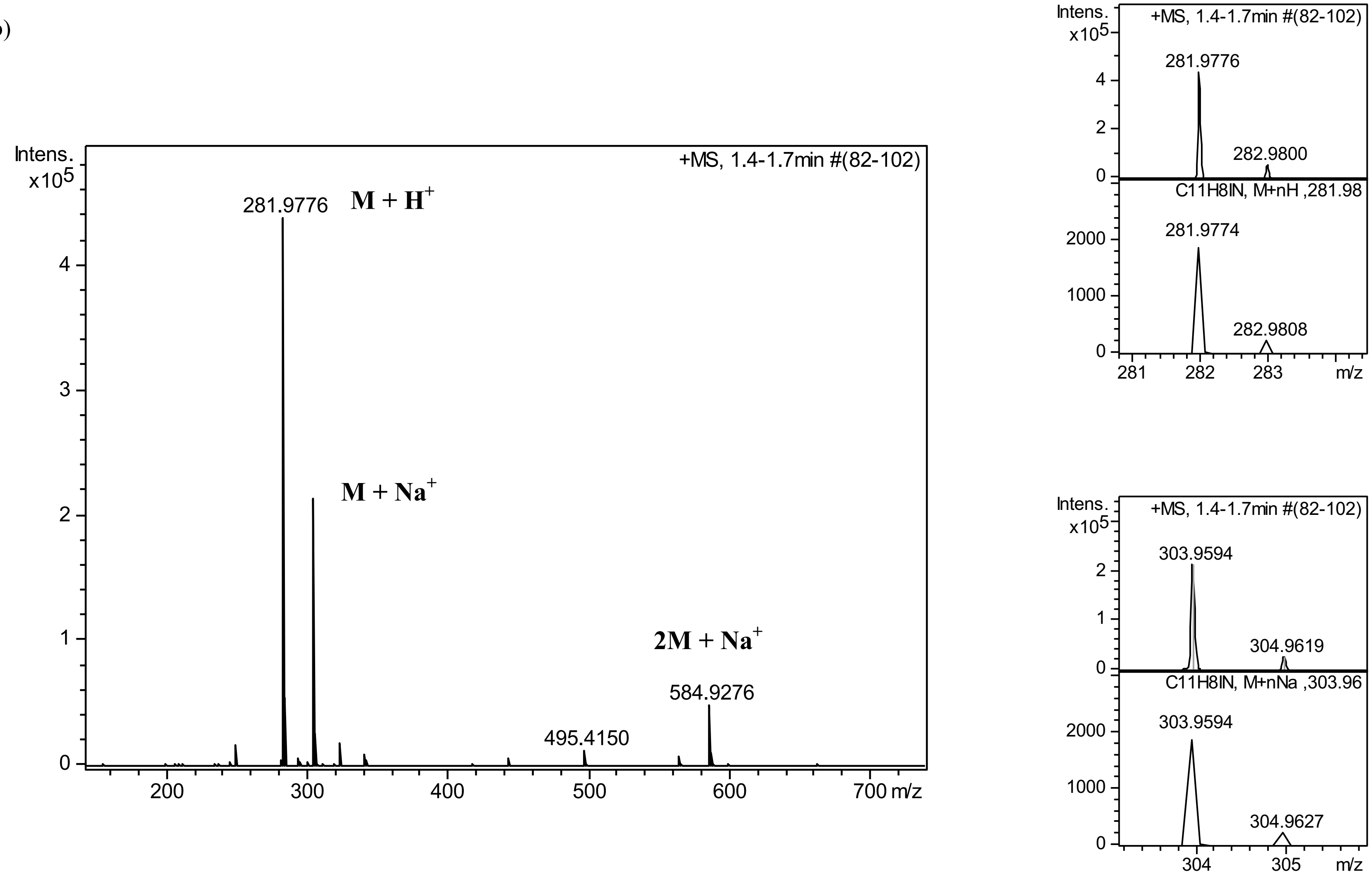
Intens.
x10^5
+MS, 1.4-1.7min #(82-102)
281.9776
M + H+
M + Na+
2M + Na+
584.9276
495.4150
m/z
+MS, 1.4-1.7min #(82-102)
281.9776
282.9800
C11H8IN, M+nH ,281.98
281.9774
282.9808
m/z
+MS, 1.4-1.7min #(82-102)
303.9594
304.9619
C11H8IN, M+nNa ,303.96
303.9594
304.9627
m/z

Figure S3. a) ^{1}H-NMR spectrum of compound **2a-Br** in $CDCl_3$, 400 MHz, at 298 K; b) HRMS (ESI-MS) spectrum (*m/z*). Observed HRMS (left) with the theoretical isotope prediction (right).

a)

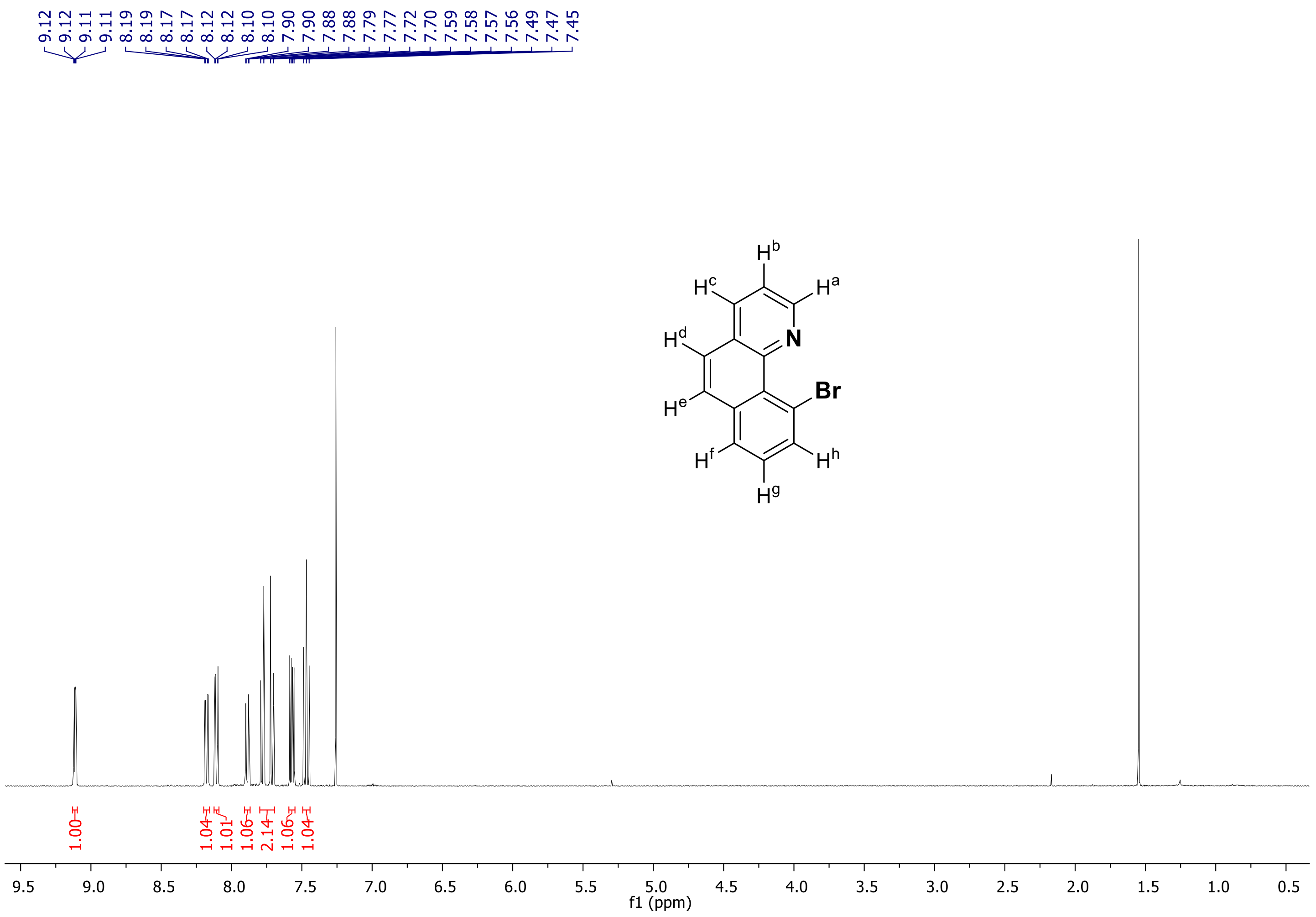

b)

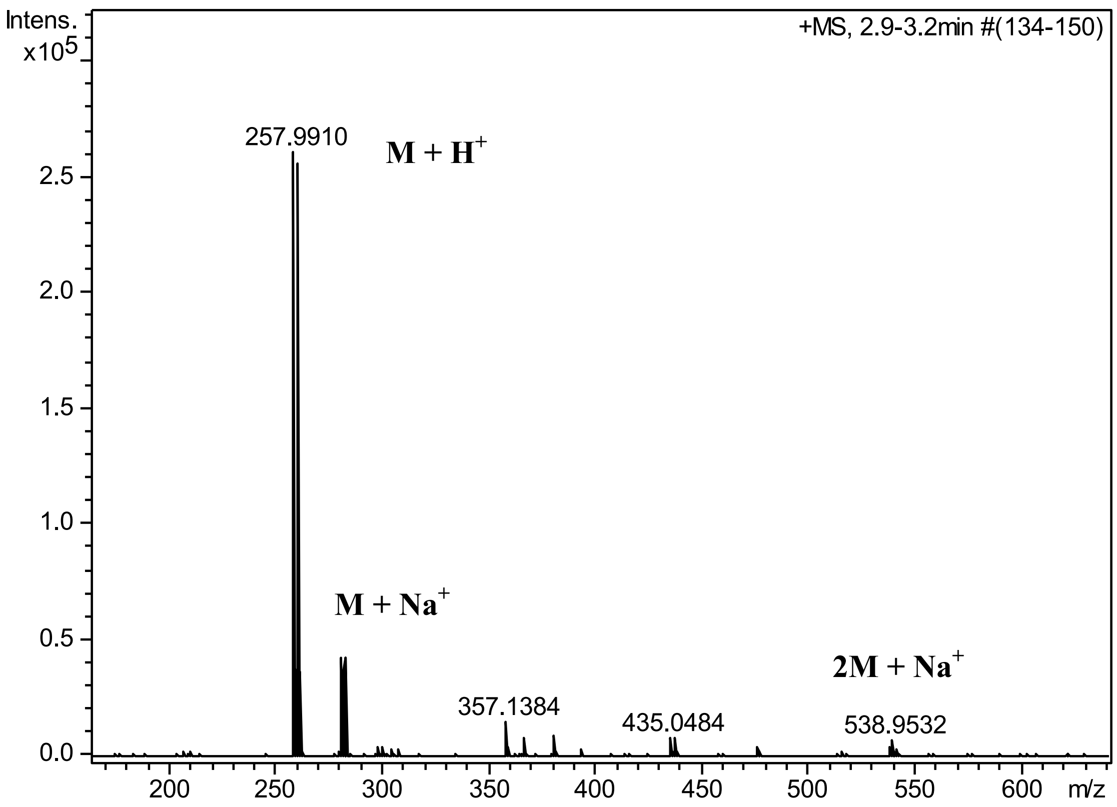
Intens.
x10^5
+MS, 2.9-3.2min #(134-150)
257.9910
M + H+
M + Na+
2M + Na+
357.1384
435.0484
538.9532
2.5
2.0
1.5
1.0
0.5
0.0
200
250
300
350
400
450
500
550
600
m/z

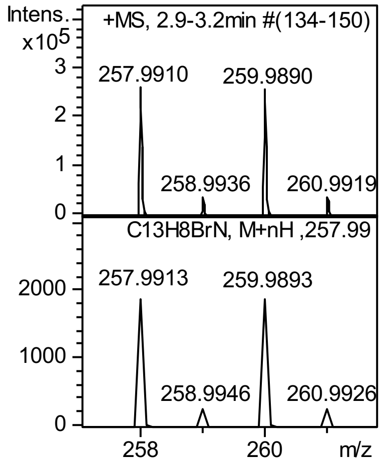
Intens.
x10^5
+MS, 2.9-3.2min #(134-150)
257.9910
259.9890
258.9936
260.9919
C13H8BrN, M+nH ,257.99
257.9913
259.9893
258.9946
260.9926
258
260
m/z

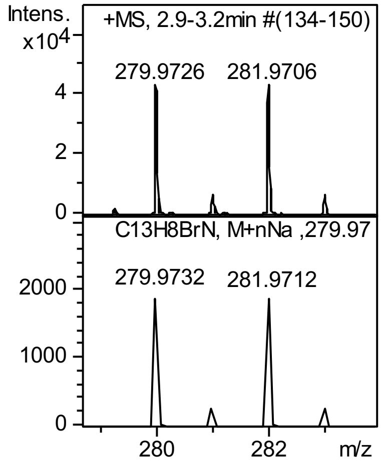
Intens.
x10^4
+MS, 2.9-3.2min #(134-150)
279.9726
281.9706
C13H8BrN, M+nNa ,279.97
279.9732
281.9712
280
282
m/z

Figure S4. a) ^{1}H-NMR spectrum of compound **2a-I** in $CDCl_3$, 400 MHz, at 298 K; b) ^{13}C{^{1}H}-NMR spectrum ($CDCl_3$, 100 MHz, 298 K); c) ^{1}H-^{1}H COSY spectrum ($CDCl_3$, 100 MHz, 298 K); d) ^{1}H-^{13}C HSQCed spectrum ($CDCl_3$, 400 MHz, 298 K); e) ^{1}H-^{13}C HMBC spectrum ($CDCl_3$, 400 MHz, 298 K); f) HRMS (ESI-MS) spectrum (*m/z*). Observed HRMS (left) with the theoretical isotope prediction (right).

a)

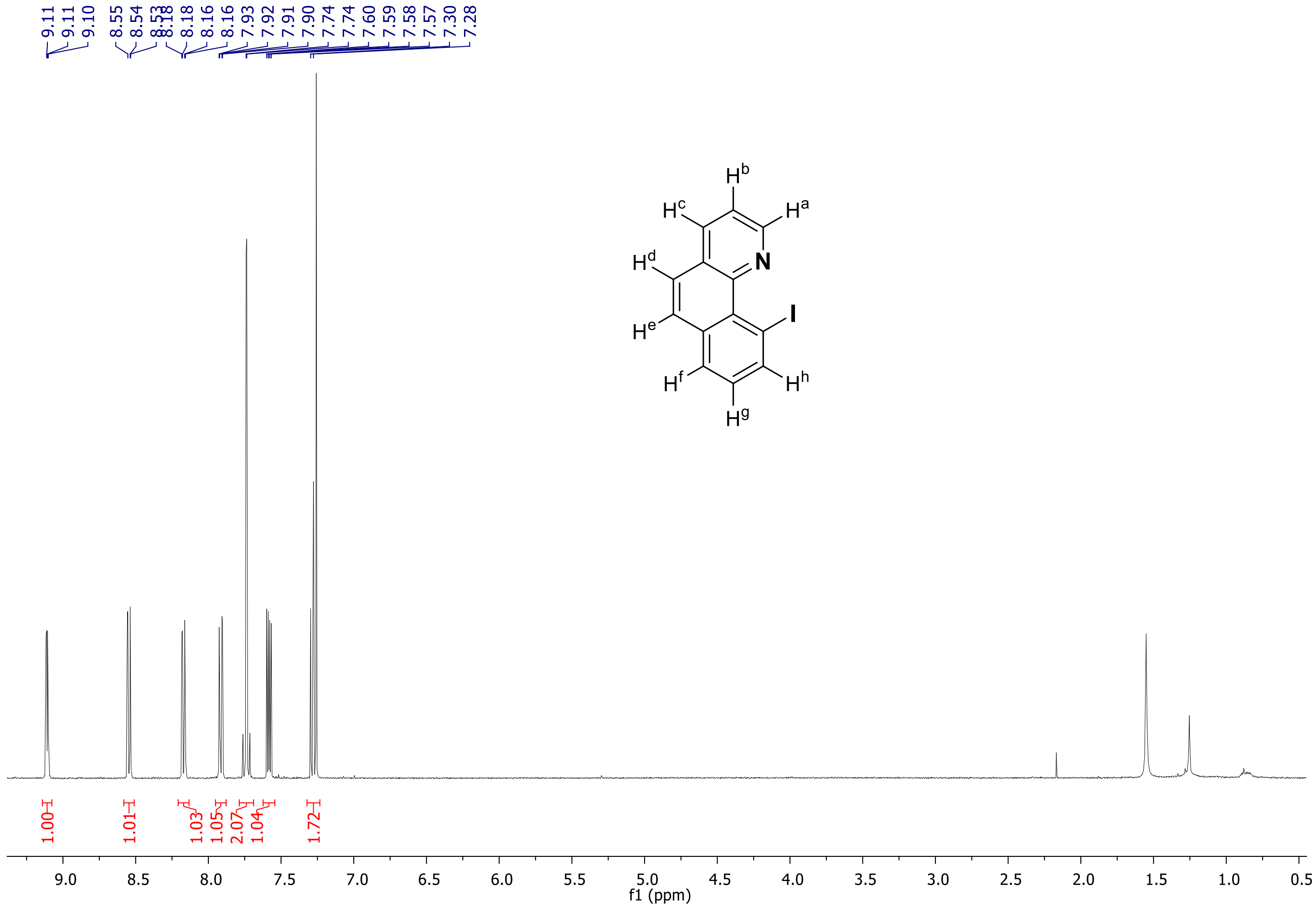

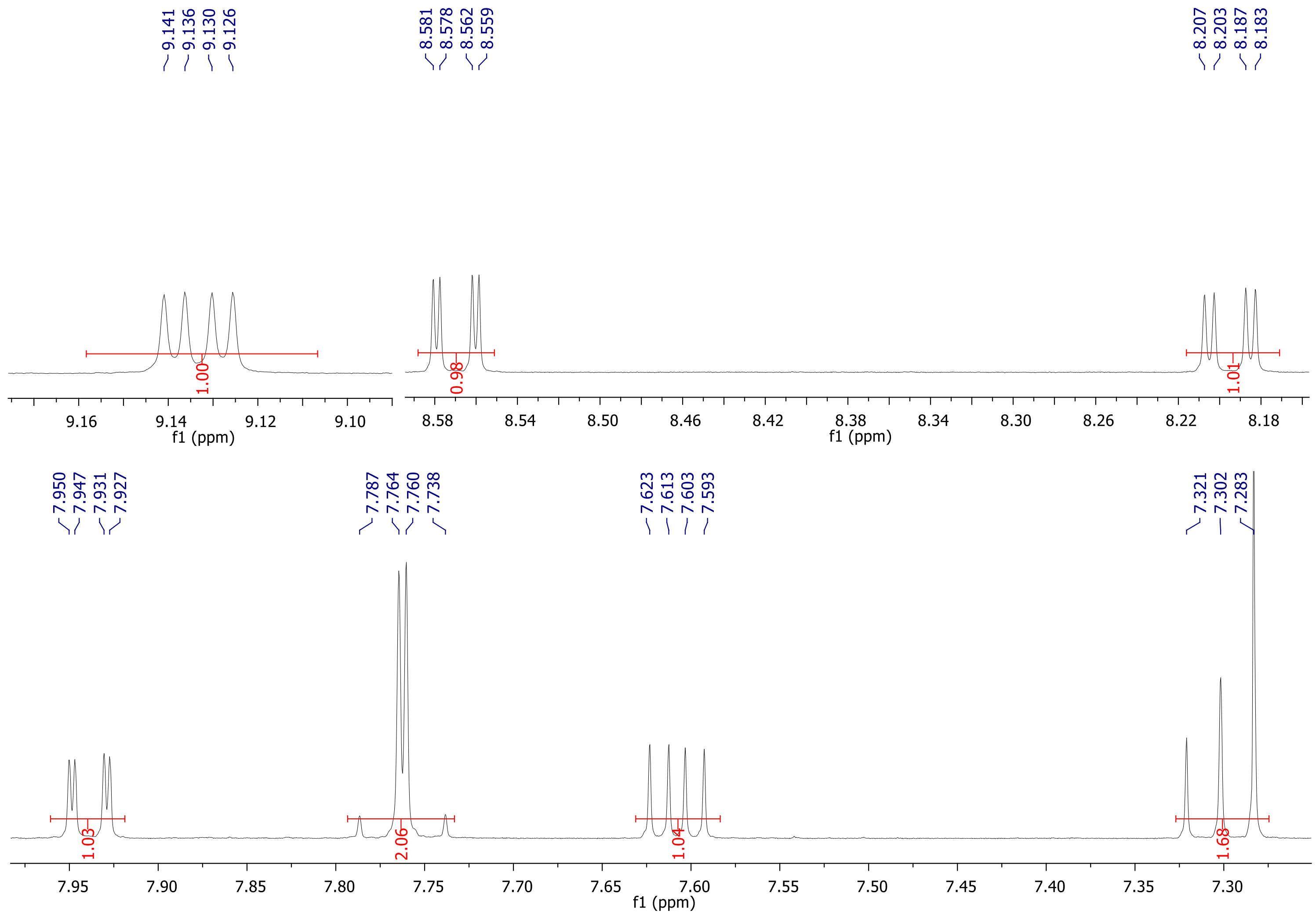

Selected aromatic region

b)

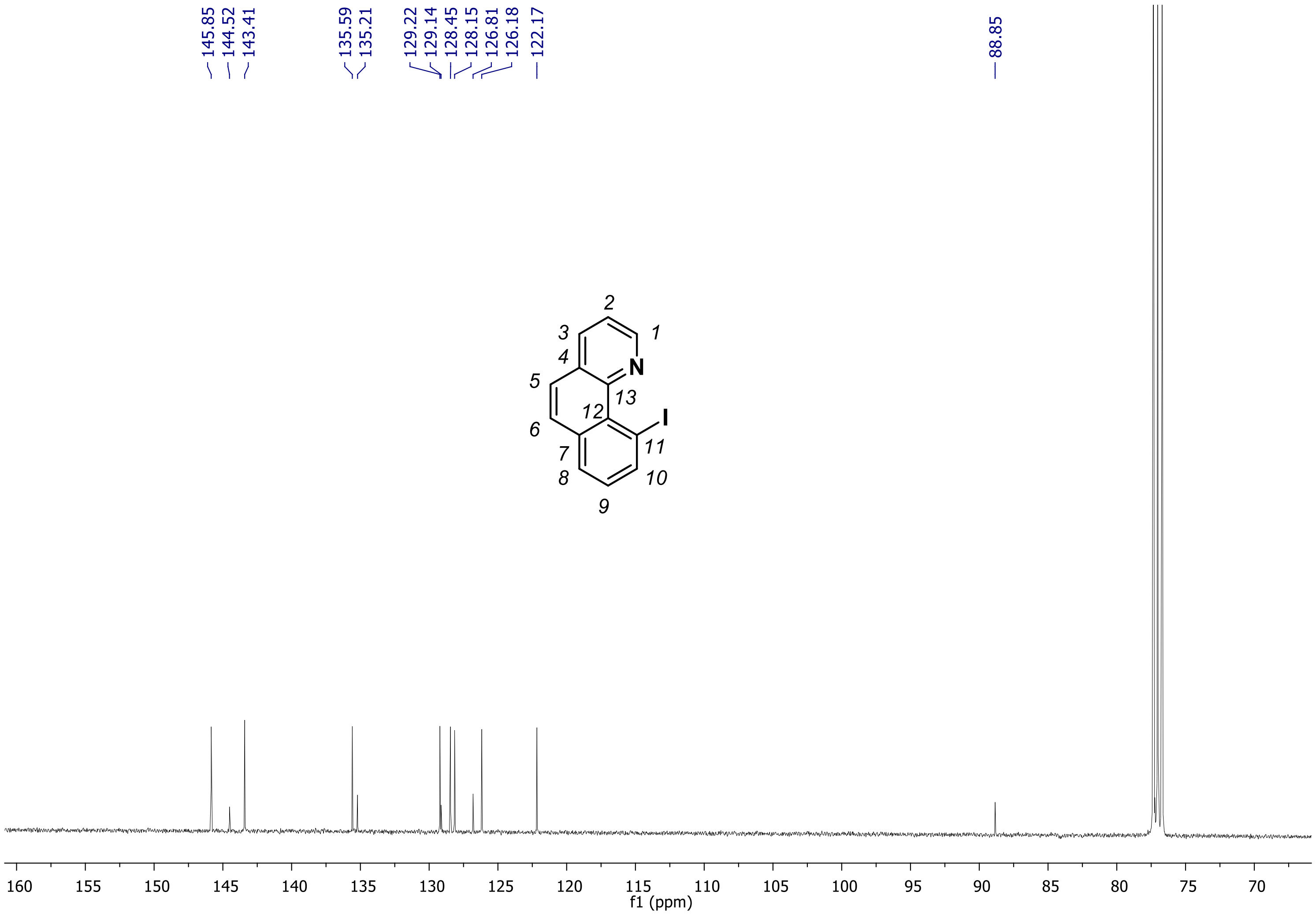
145.85
144.52
143.41
135.59
135.21
129.22
129.14
128.45
128.15
126.81
126.18
122.17
88.85
1
2
3
4
5
6
7
8
9
10
11
12
13
N
I
160 155 150 145 140 135 130 125 120 115 110 105 100 95 90 85 80 75 70
f1 (ppm)

c)

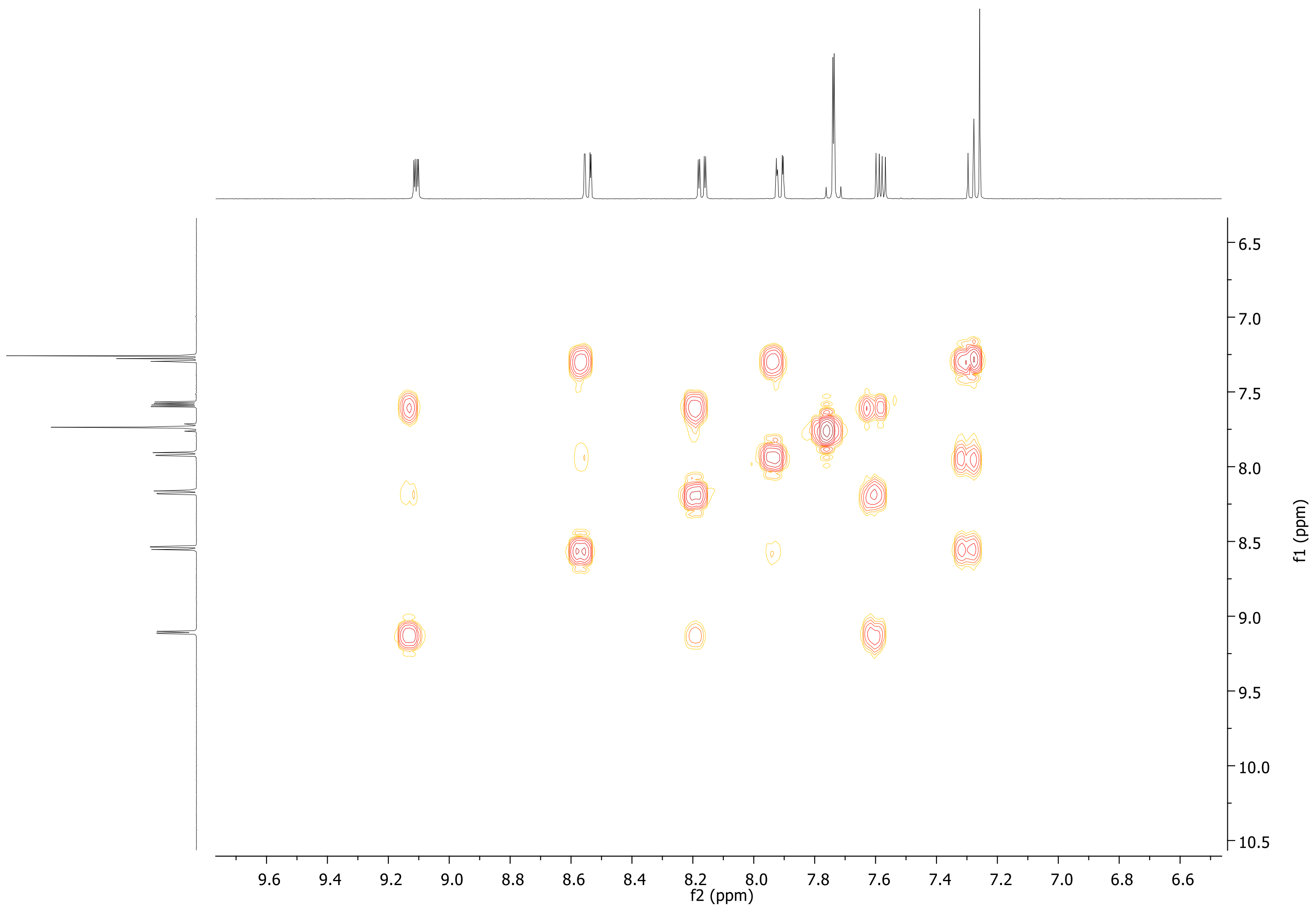

d)

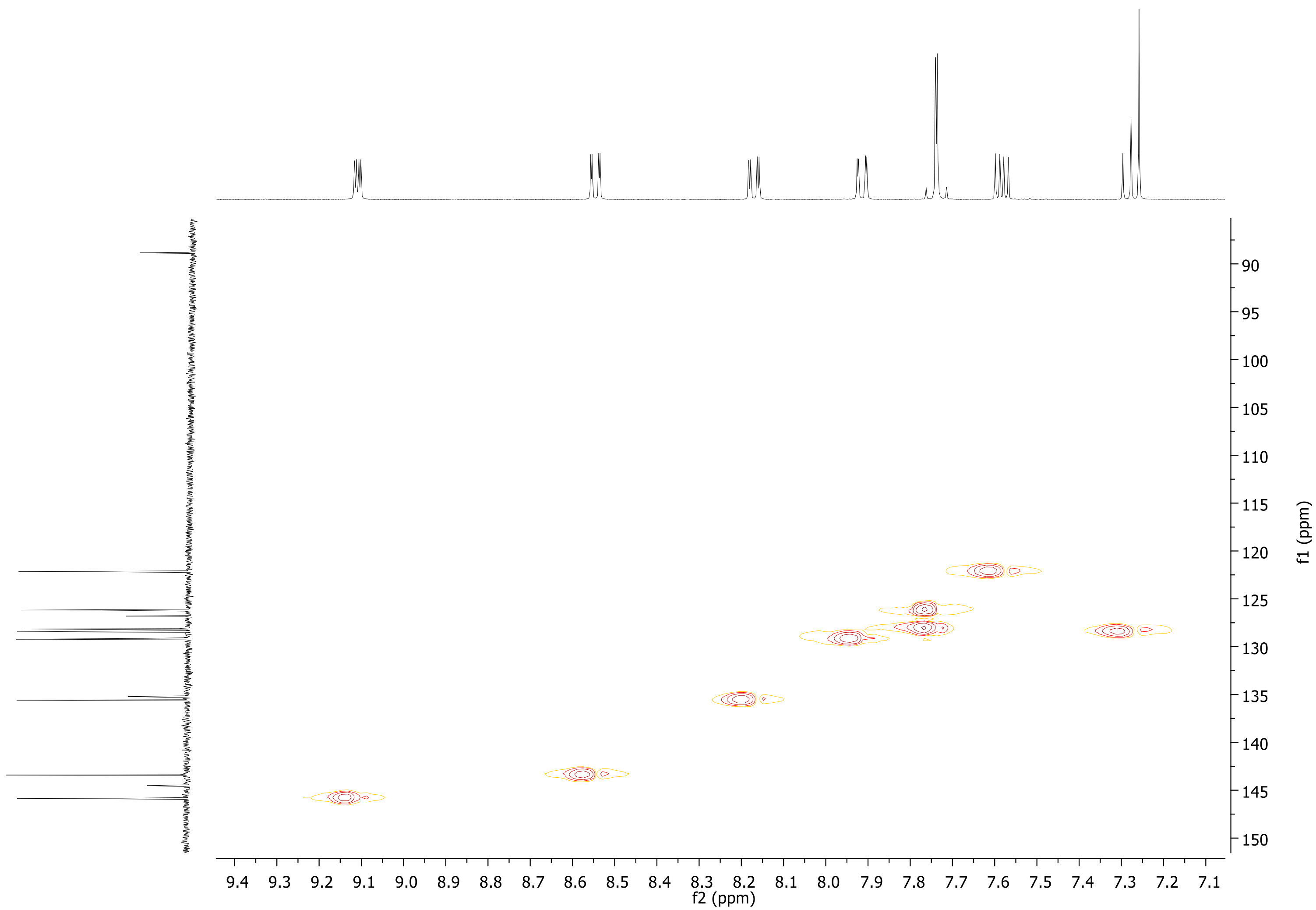

90
95
100
105
110
115
120
125
130
135
140
145
150
f1 (ppm)
9.4
9.3
9.2
9.1
9.0
8.9
8.8
8.7
8.6
8.5
8.4
8.3
8.2
8.1
8.0
7.9
7.8
7.7
7.6
7.5
7.4
7.3
7.2
7.1
f2 (ppm)

e)

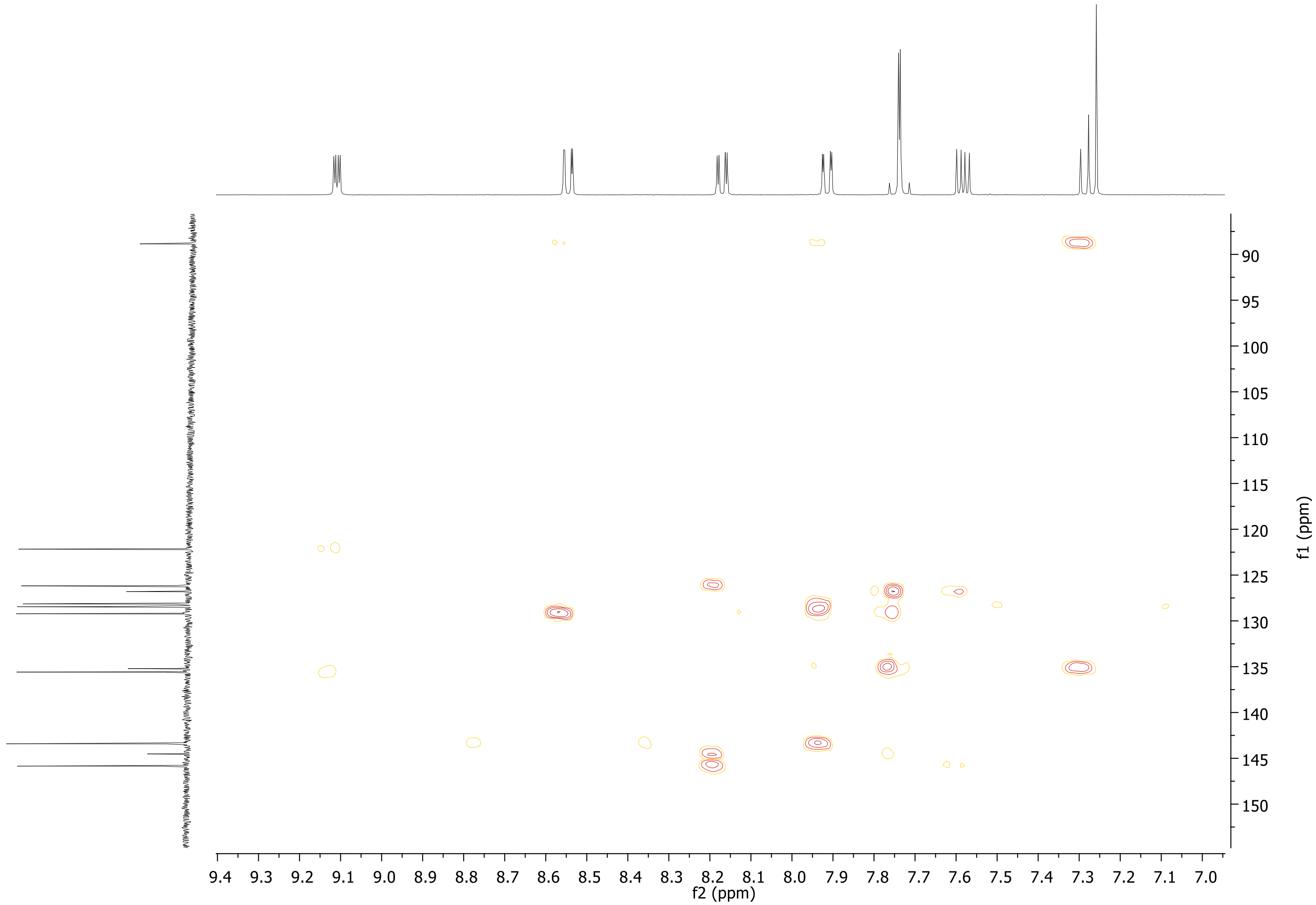

90
95
100
105
110
115
120
125
130
135
140
145
150
f1 (ppm)
9.4
9.3
9.2
9.1
9.0
8.9
8.8
8.7
8.6
8.5
8.4
8.3
8.2
8.1
8.0
7.9
7.8
7.7
7.6
7.5
7.4
7.3
7.2
7.1
7.0
f2 (ppm)

f)

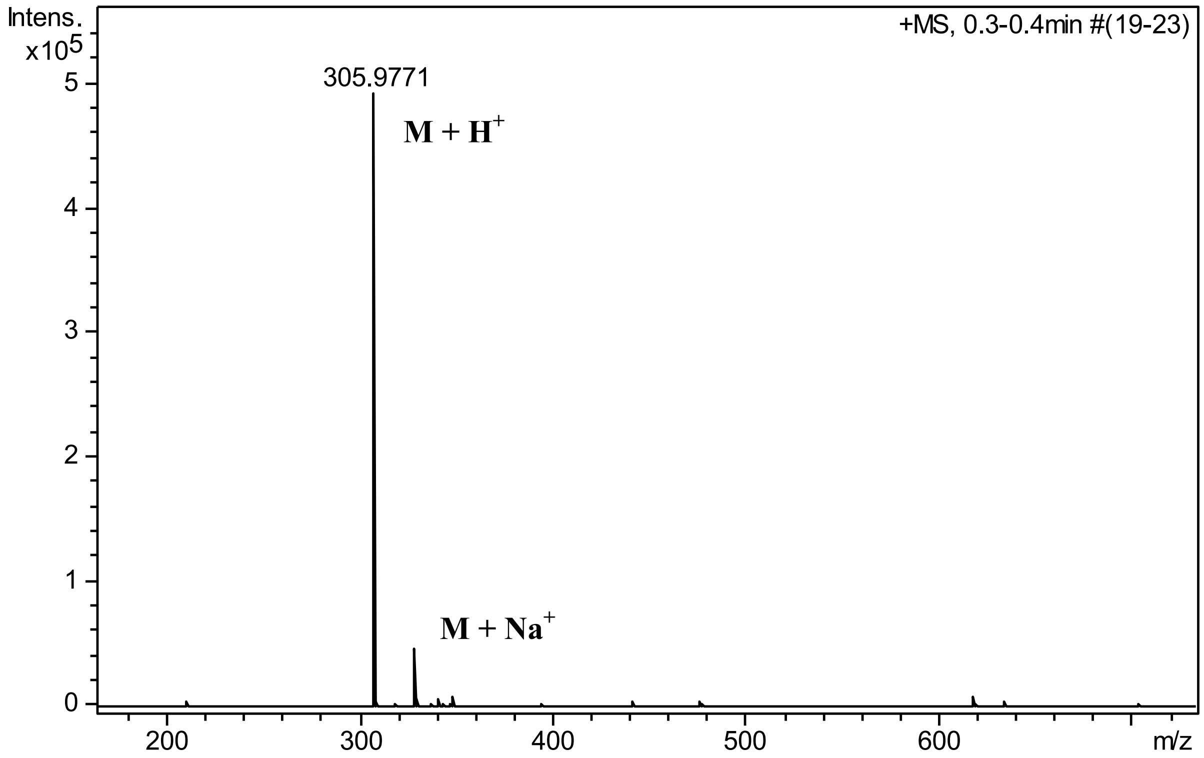

Intens.
x10^5
+MS, 0.3-0.4min #(19-23)
305.9771
M + H+
M + Na+
m/z

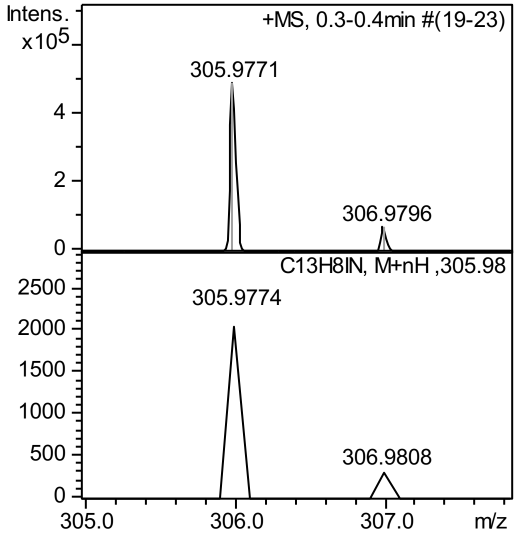

Intens.
x10^5
+MS, 0.3-0.4min #(19-23)
305.9771
306.9796
C13H8IN, M+nH ,305.98
305.9774
306.9808
m/z

Figure S5. a) ^{1}H-NMR spectrum of complex **3a** in $CDCl_3$, 400 MHz, at 323 K; b) $^{13}C\{^{1}H\}$-NMR spectrum ($CDCl_3$, 100 MHz, 323 K); c) ^{1}H-^{1}H COSY spectrum ($CDCl_3$, 100 MHz, 323 K); d) ^{1}H-^{13}C HSQCed spectrum ($CDCl_3$, 400 MHz, 323 K).

a)

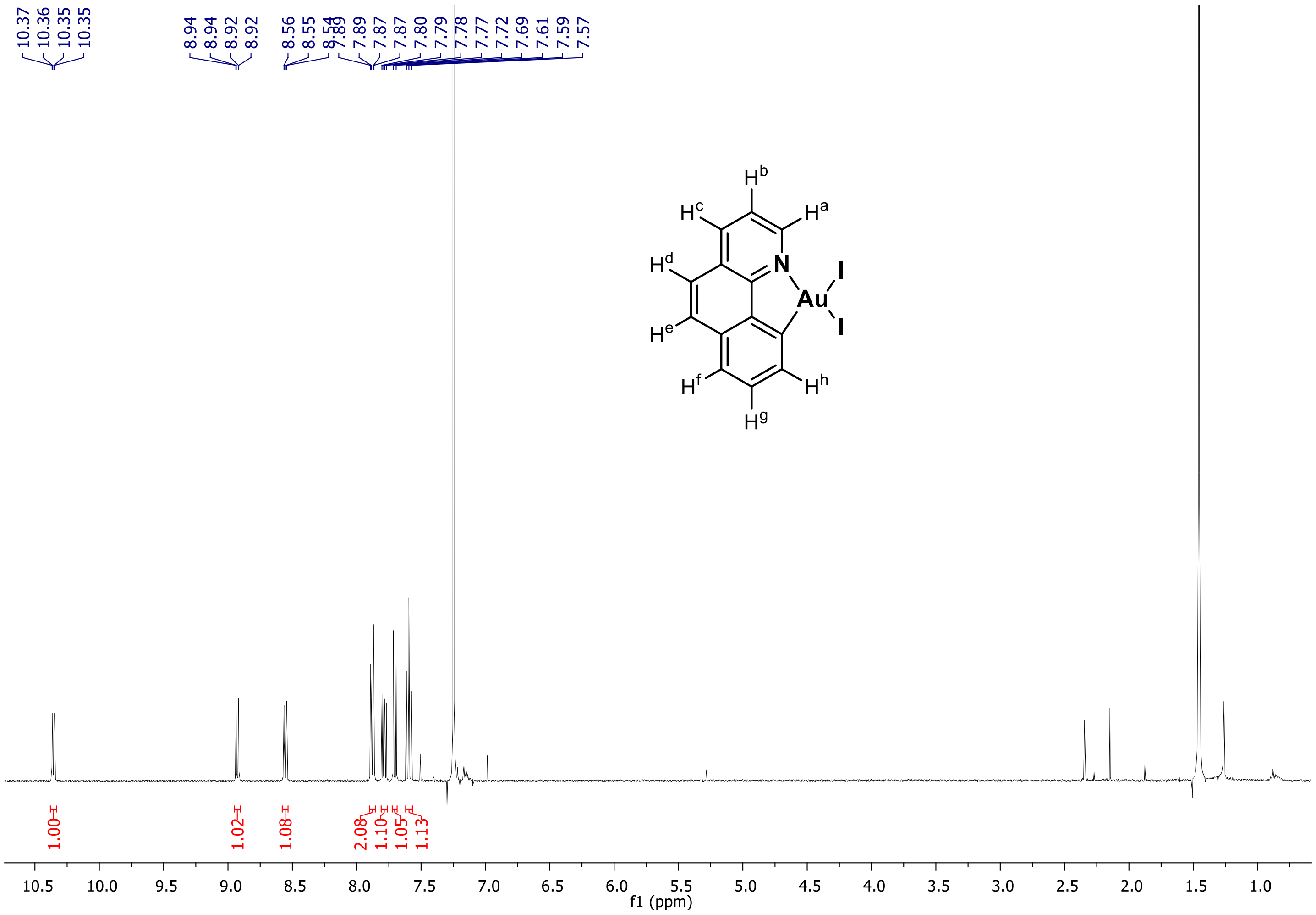

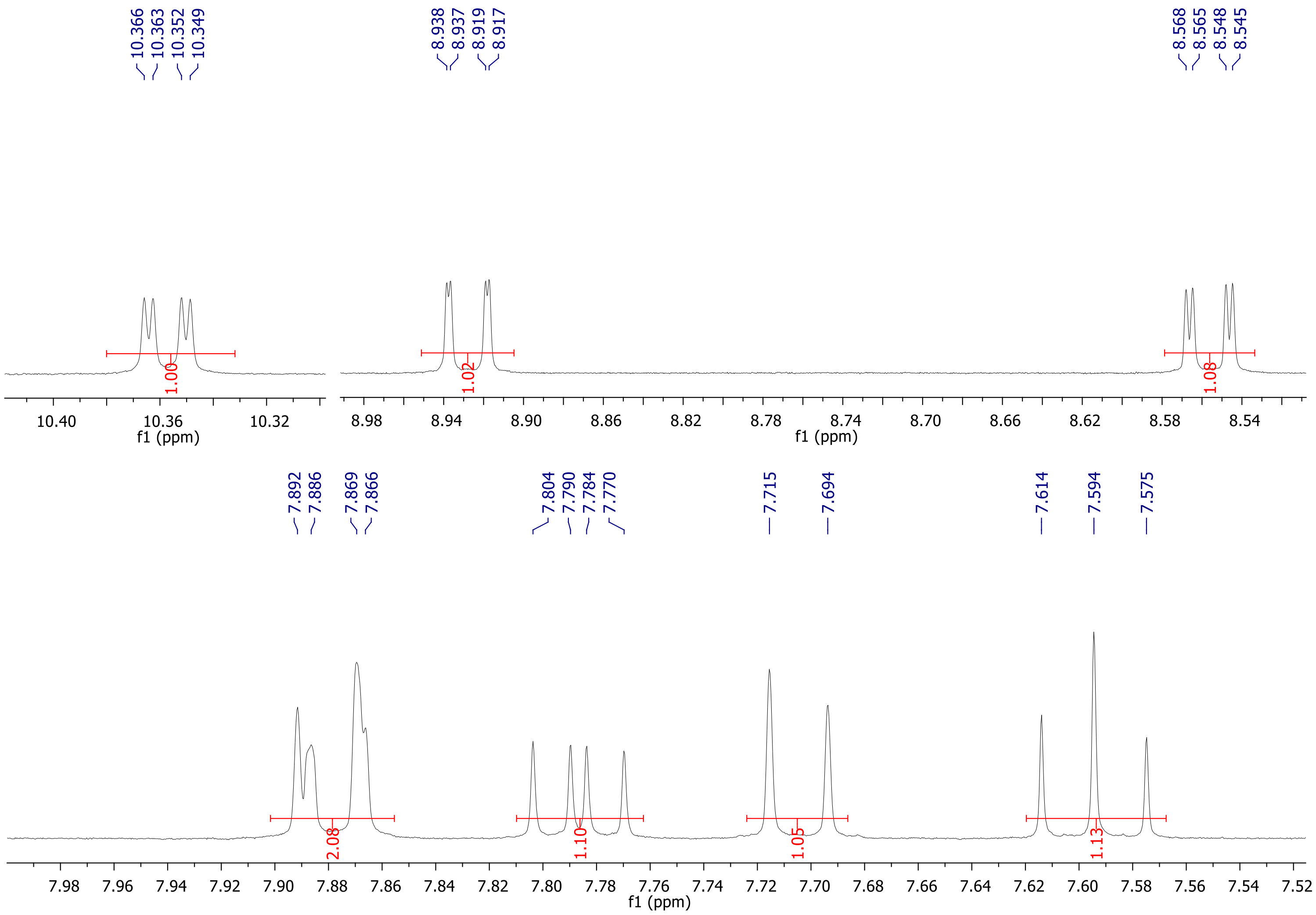

Selected aromatic region

b)

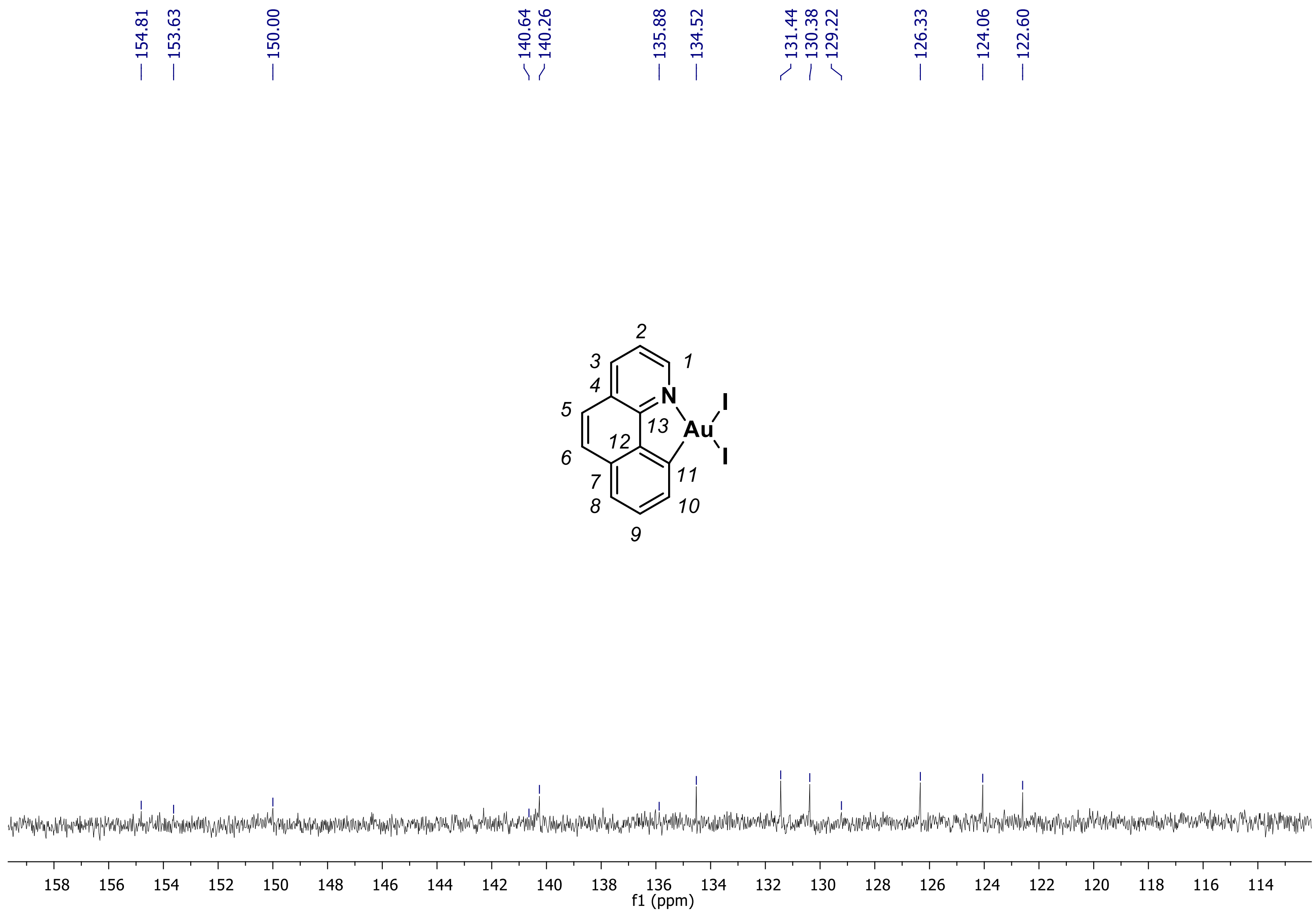

154.81
153.63
150.00
140.64
140.26
135.88
134.52
131.44
130.38
129.22
126.33
124.06
122.60
1
2
3
4
5
6
7
8
9
10
11
12
13
N
Au
I
I
158 156 154 152 150 148 146 144 142 140 138 136 134 132 130 128 126 124 122 120 118 116 114
f1 (ppm)

c)

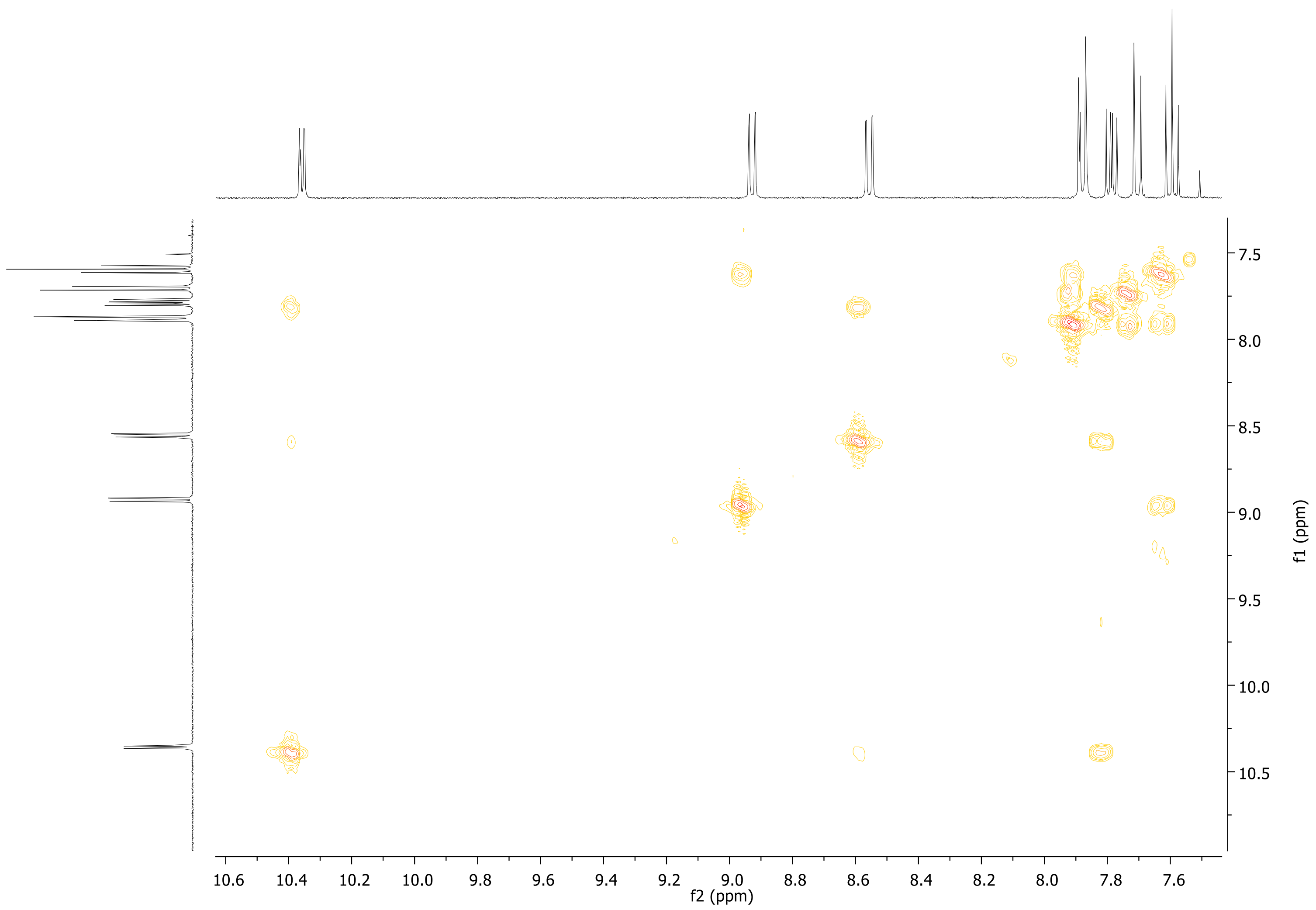

7.5
8.0
8.5
9.0
9.5
10.0
10.5
f1 (ppm)
10.6
10.4
10.2
10.0
9.8
9.6
9.4
9.2
9.0
8.8
8.6
8.4
8.2
8.0
7.8
7.6
f2 (ppm)

d)

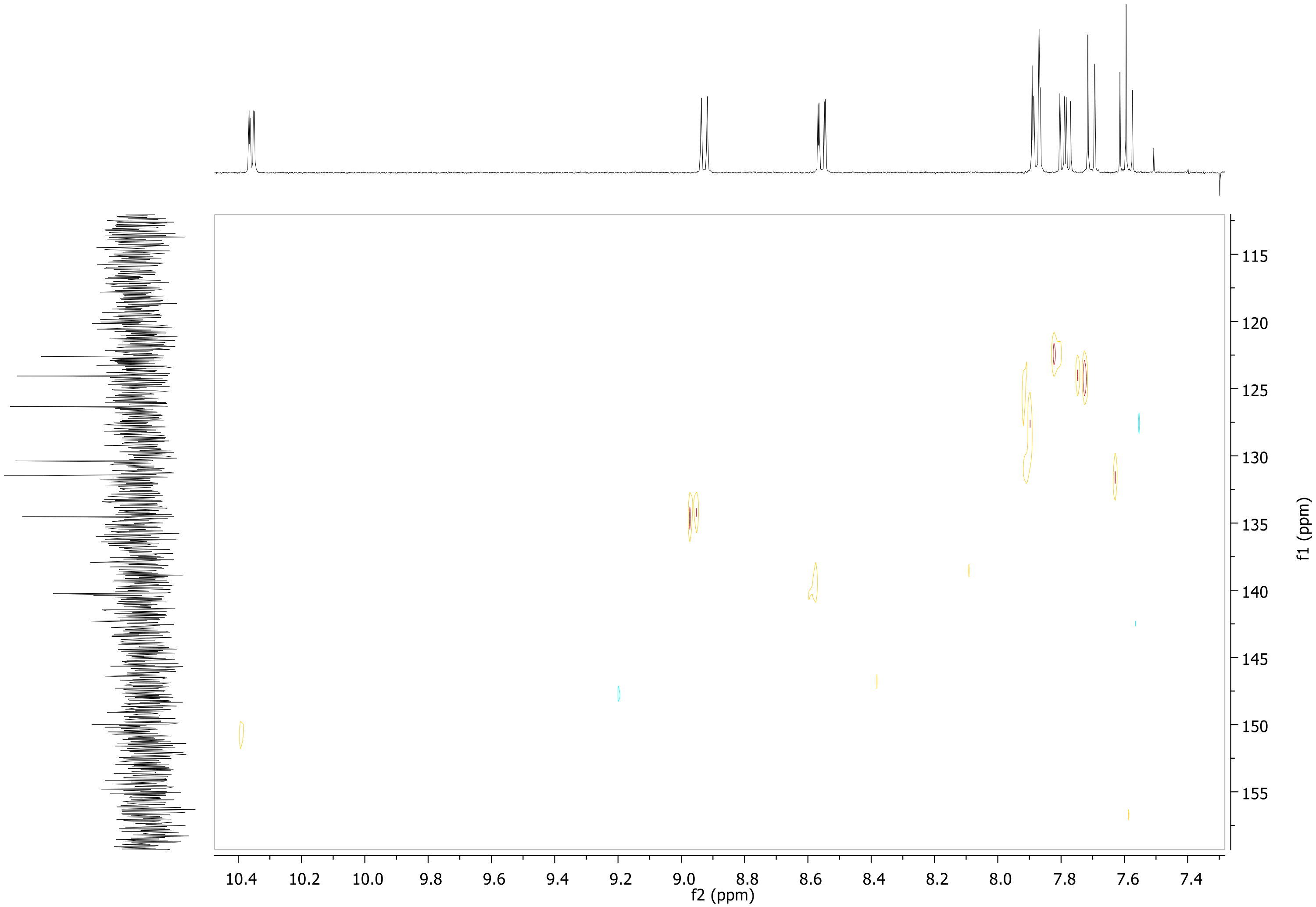

10.4
10.2
10.0
9.8
9.6
9.4
9.2
9.0
8.8
8.6
8.4
8.2
8.0
7.8
7.6
7.4
f2 (ppm)
115
120
125
130
135
140
145
150
155
f1 (ppm)

Figure S6. a) ^{1}H-NMR spectrum of complex **3b** in $CDCl_3$, 400 MHz, at 248 K; b) $^{13}C\{^{1}H\}$-NMR spectrum ($CDCl_3$, 100 MHz, 248 K); c) ^{1}H-^{1}H COSY spectrum ($CDCl_3$, 100 MHz, 248 K); d) ^{1}H-^{13}C HSQCed spectrum ($CDCl_3$, 400 MHz, 248 K); e) ^{1}H-^{13}C HMBC spectrum ($CDCl_3$, 400 MHz, 248 K); f) HRMS (ESI-MS) spectrum (*m/z*). Observed HRMS (top) with the theoretical isotope prediction (bottom).

a)

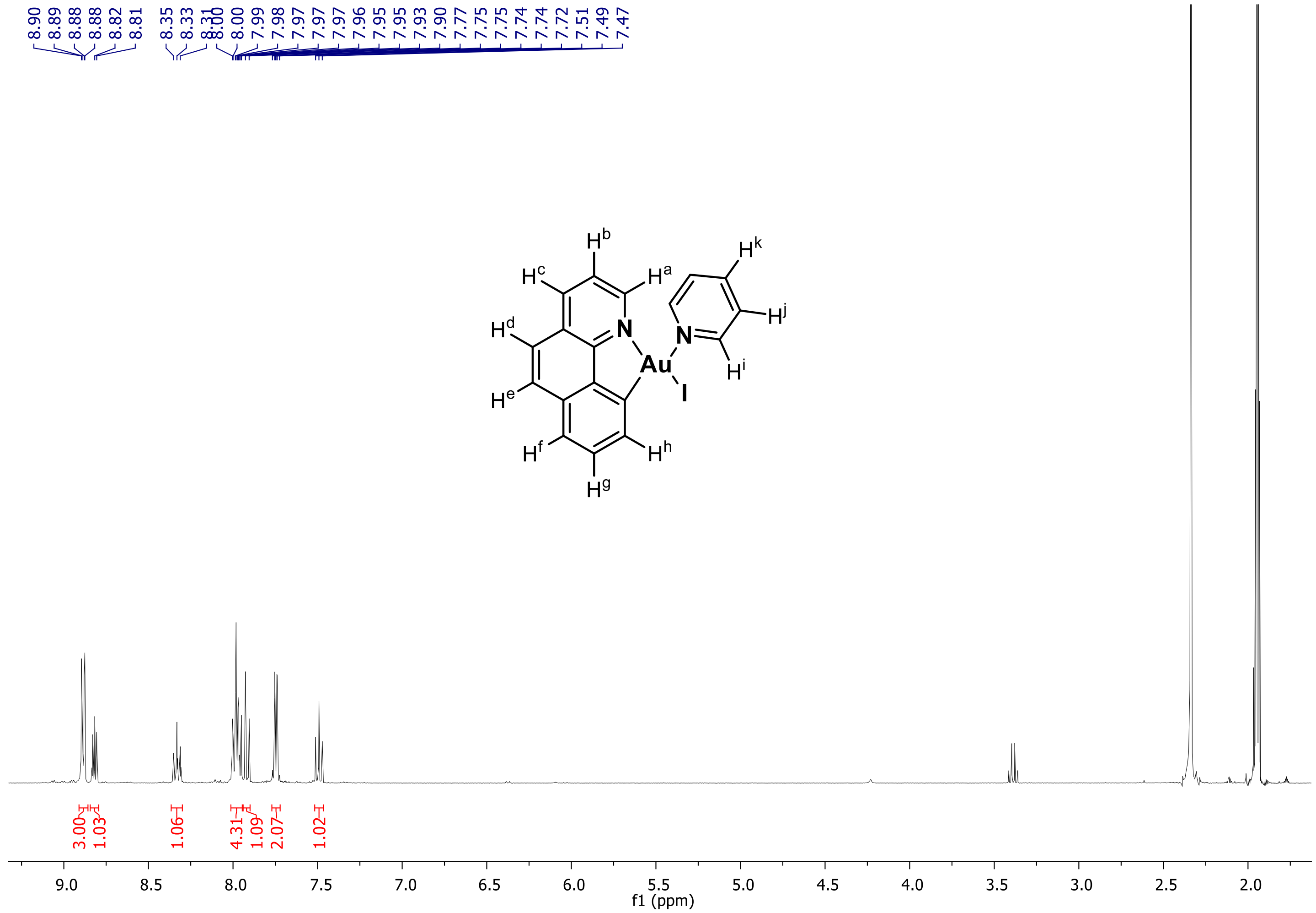

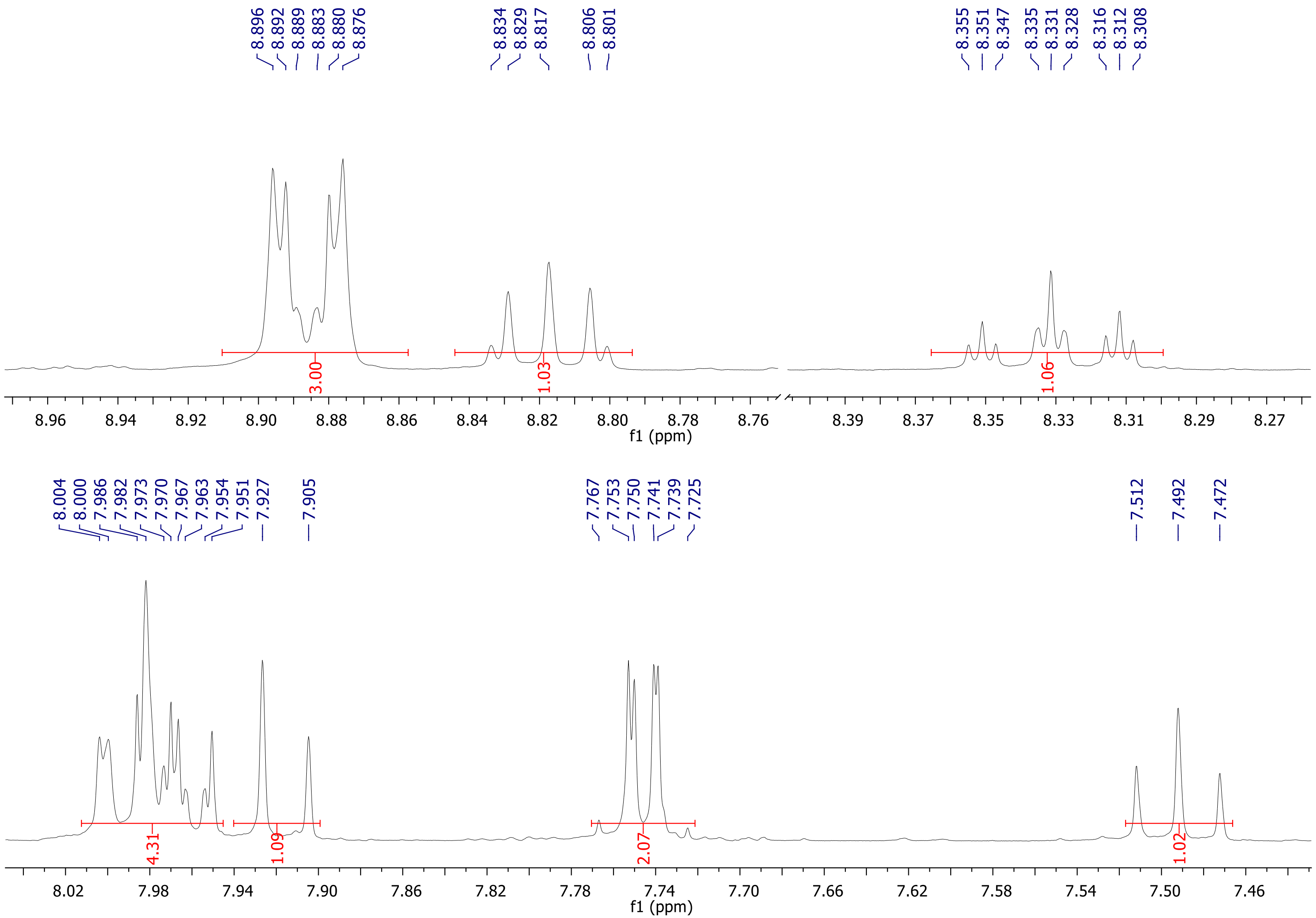

Selected aromatic region

b)

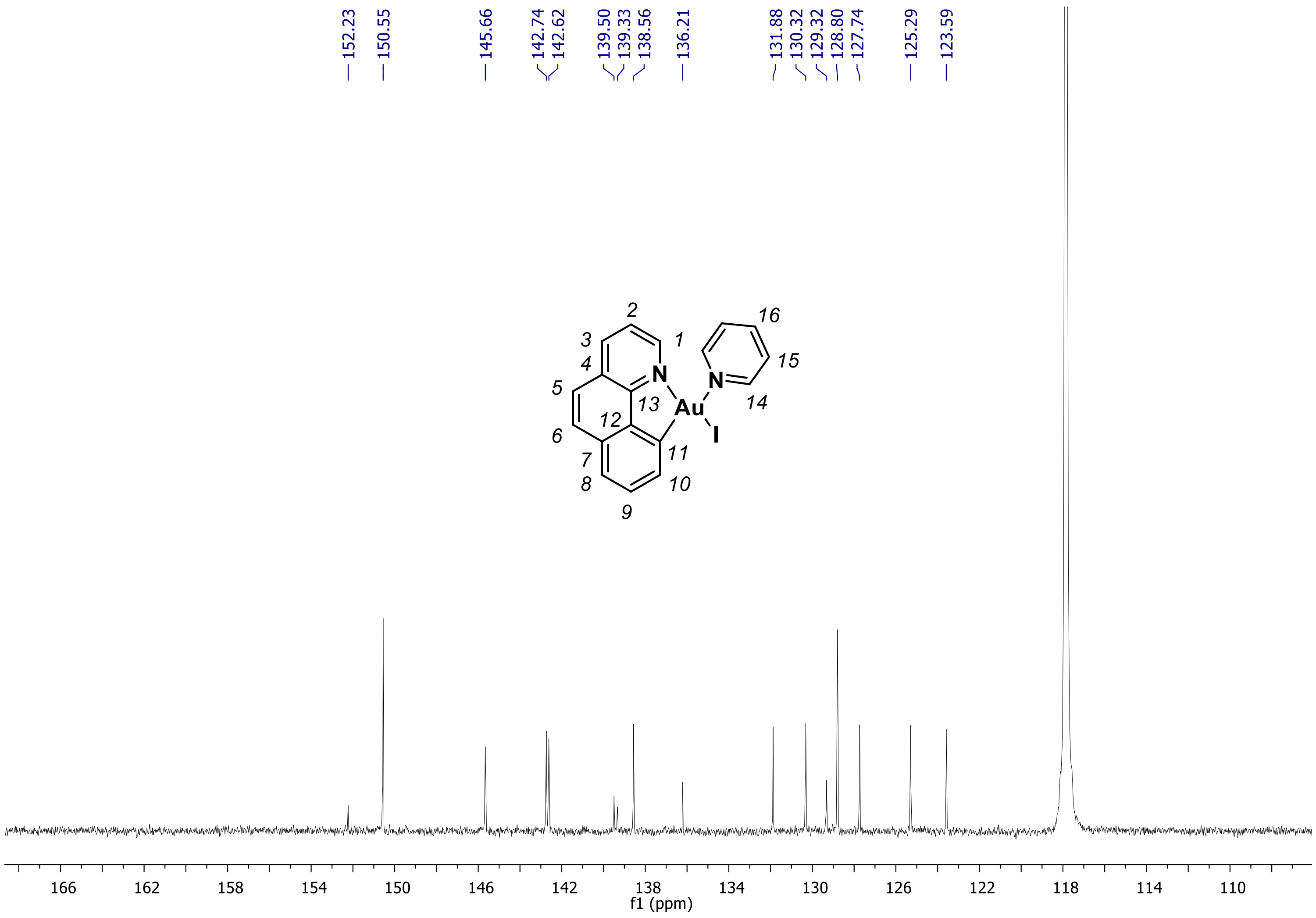
152.23
150.55
145.66
142.74
142.62
139.50
139.33
138.56
136.21
131.88
130.32
129.32
128.80
127.74
125.29
123.59
N
Au
I
N
1
2
3
4
5
6
7
8
9
10
11
12
13
14
15
16
166
162
158
154
150
146
142
138
134
130
126
122
118
114
110
f1 (ppm)

c)

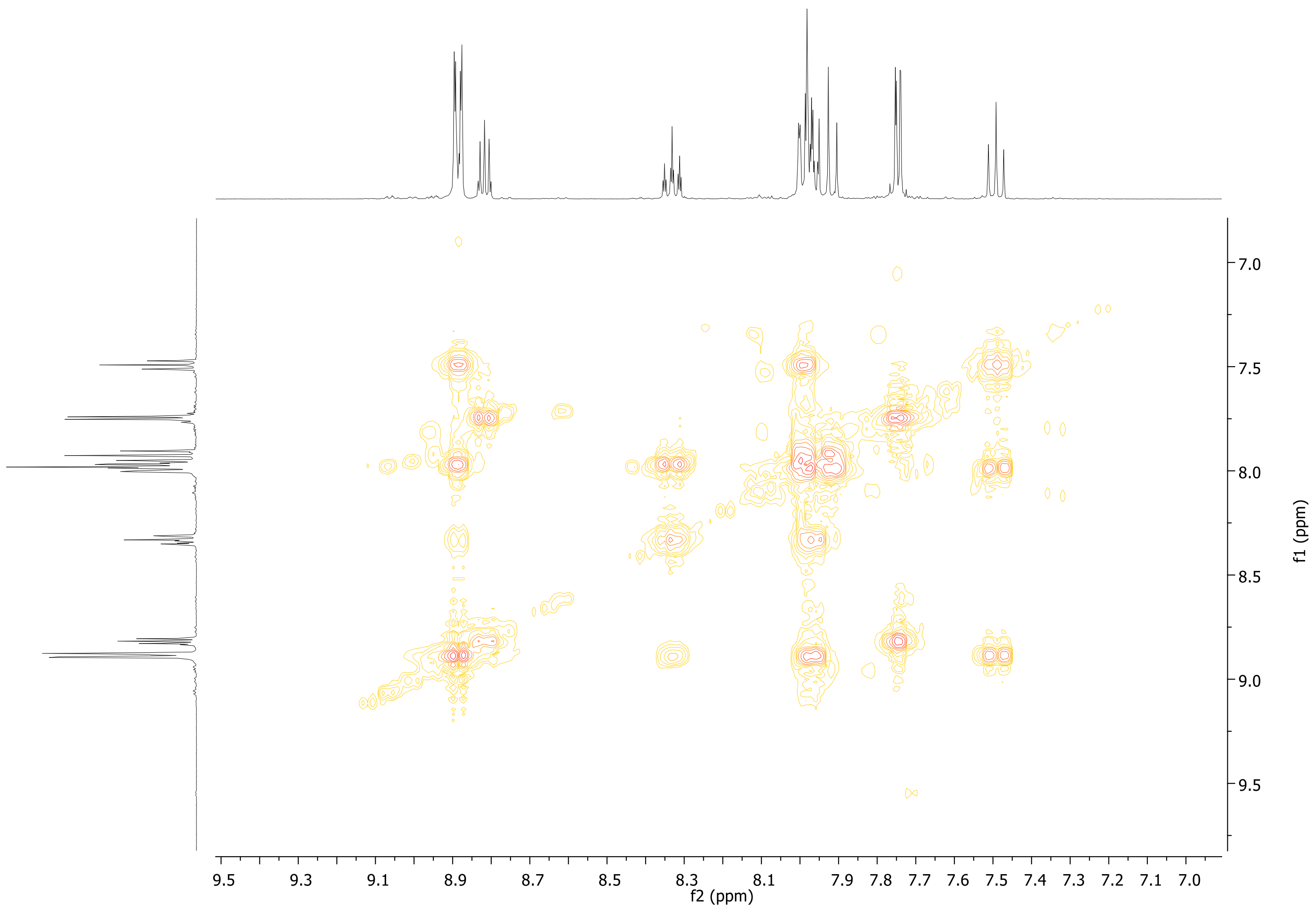

9.5
9.3
9.1
8.9
8.7
8.5
8.3
8.1
7.9
7.8
7.7
7.6
7.5
7.4
7.3
7.2
7.1
7.0
f2 (ppm)
7.0
7.5
8.0
8.5
9.0
9.5
f1 (ppm)

d)

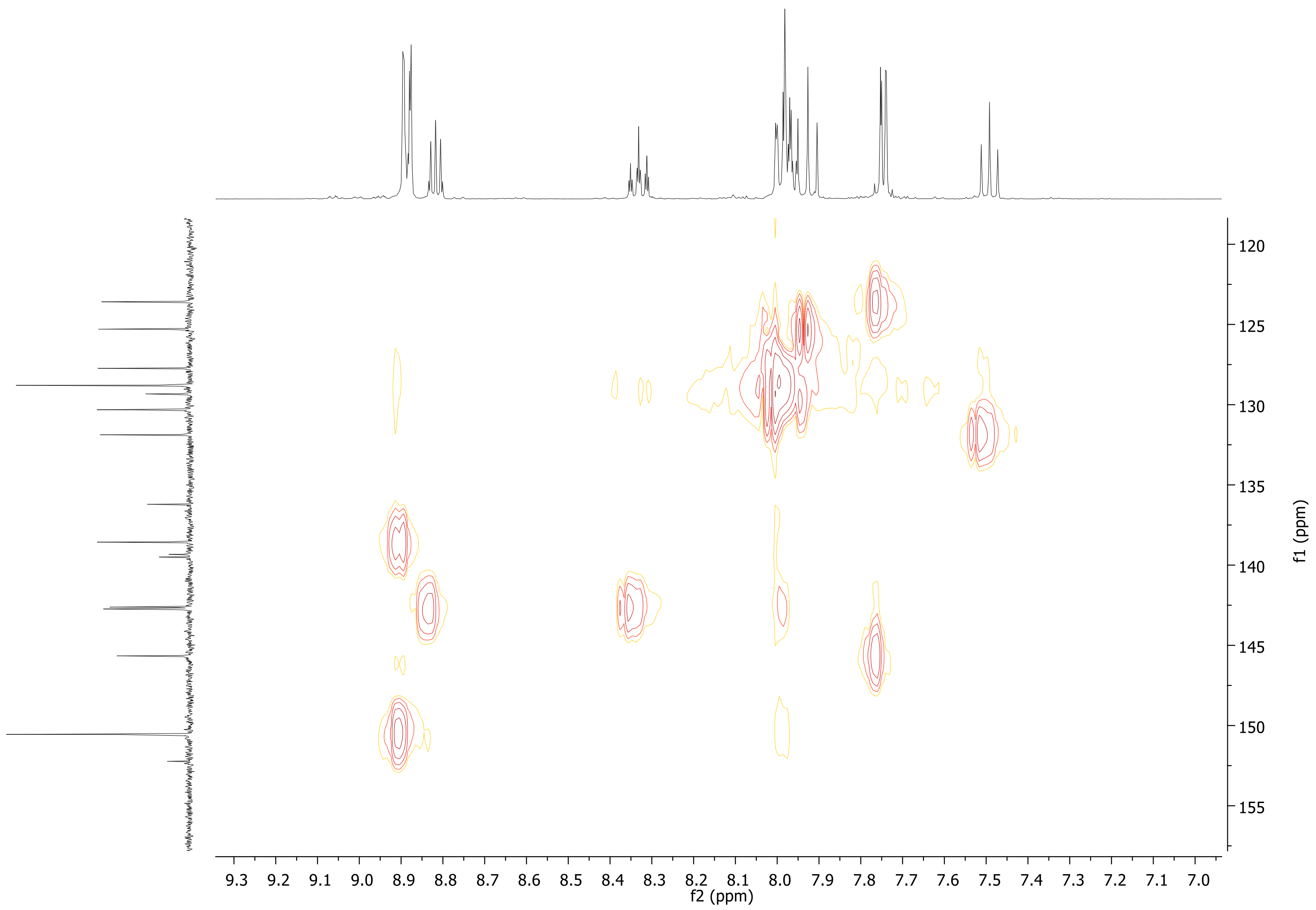

f2 (ppm)
f1 (ppm)
9.3 9.2 9.1 9.0 8.9 8.8 8.7 8.6 8.5 8.4 8.3 8.2 8.1 8.0 7.9 7.8 7.7 7.6 7.5 7.4 7.3 7.2 7.1 7.0
120 125 130 135 140 145 150 155

e)

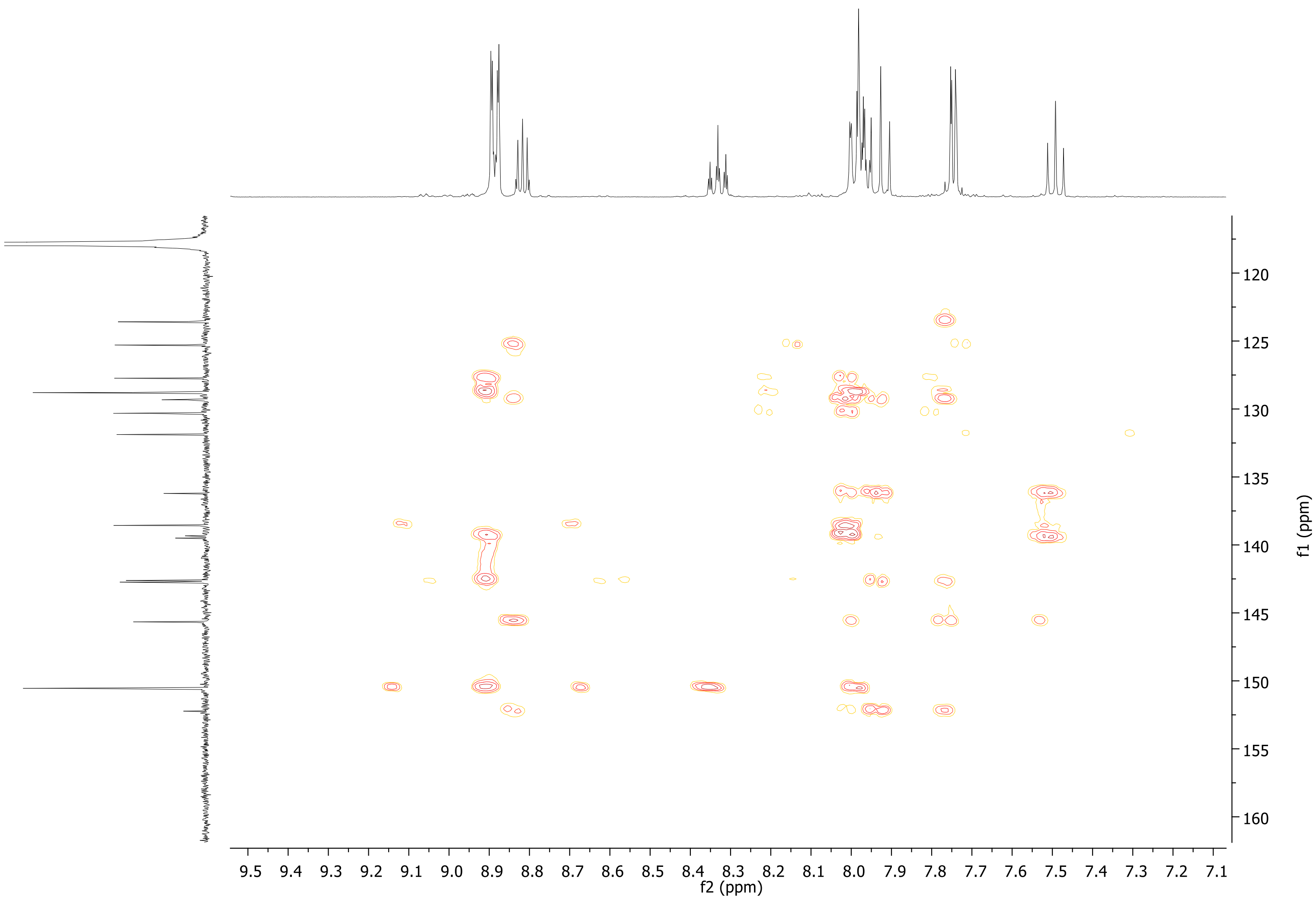

9.5 9.4 9.3 9.2 9.1 9.0 8.9 8.8 8.7 8.6 8.5 8.4 8.3 8.2 8.1 8.0 7.9 7.8 7.7 7.6 7.5 7.4 7.3 7.2 7.1
f2 (ppm)
120 125 130 135 140 145 150 155 160
f1 (ppm)

f)

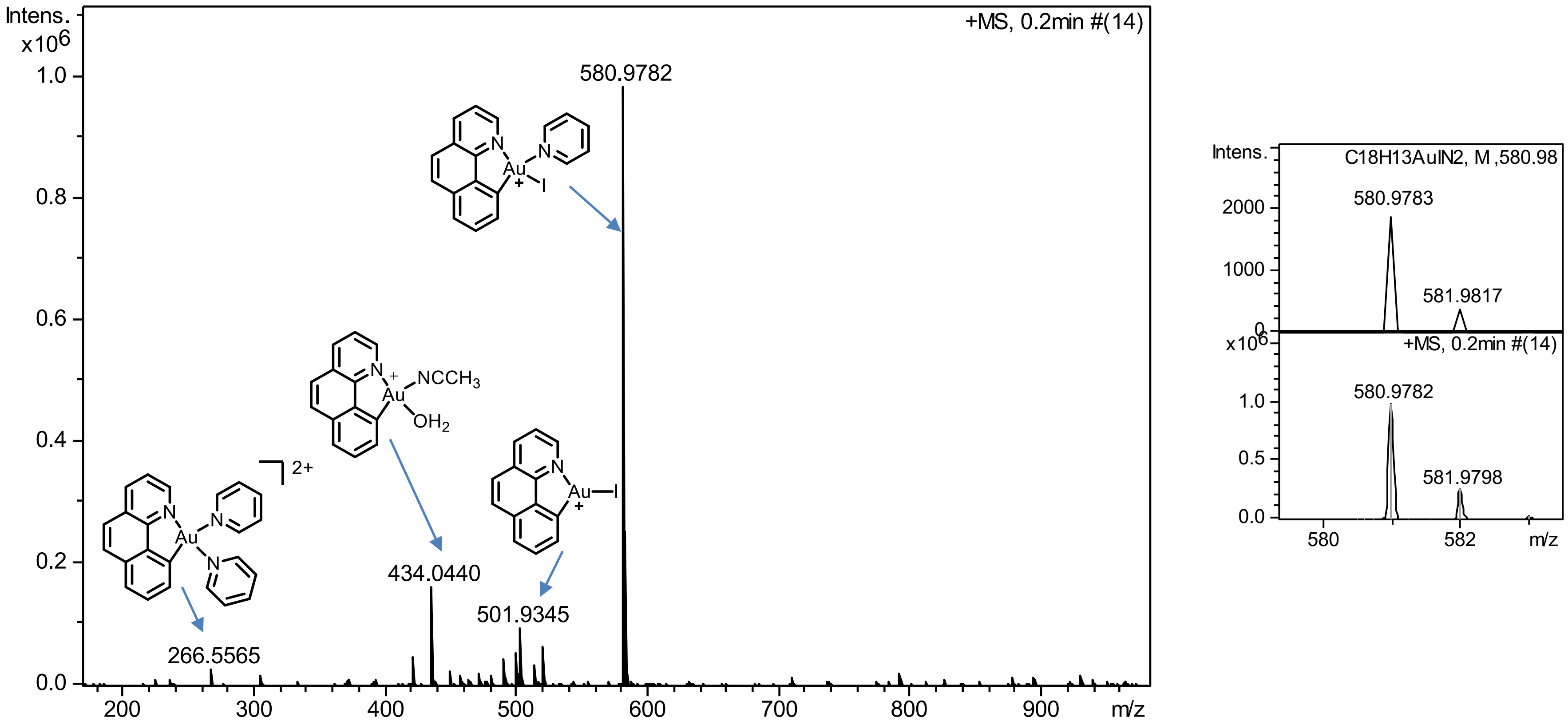

Intens.
x10^6
+MS, 0.2min #(14)
580.9782
434.0440
501.9345
266.5565
2+
NCCH3
OH2
Au
N
I
m/z
C18H13AuIN2, M ,580.98
580.9783
581.9817
580.9782
581.9798

Figure S7. a) ^{1}H-NMR spectrum of compound **1aa** in $CDCl_3$, 400 MHz, at 298 K; b) HRMS (ESI-MS) spectrum (*m/z*). Observed HRMS (left) with the theoretical isotope prediction (right).

a)

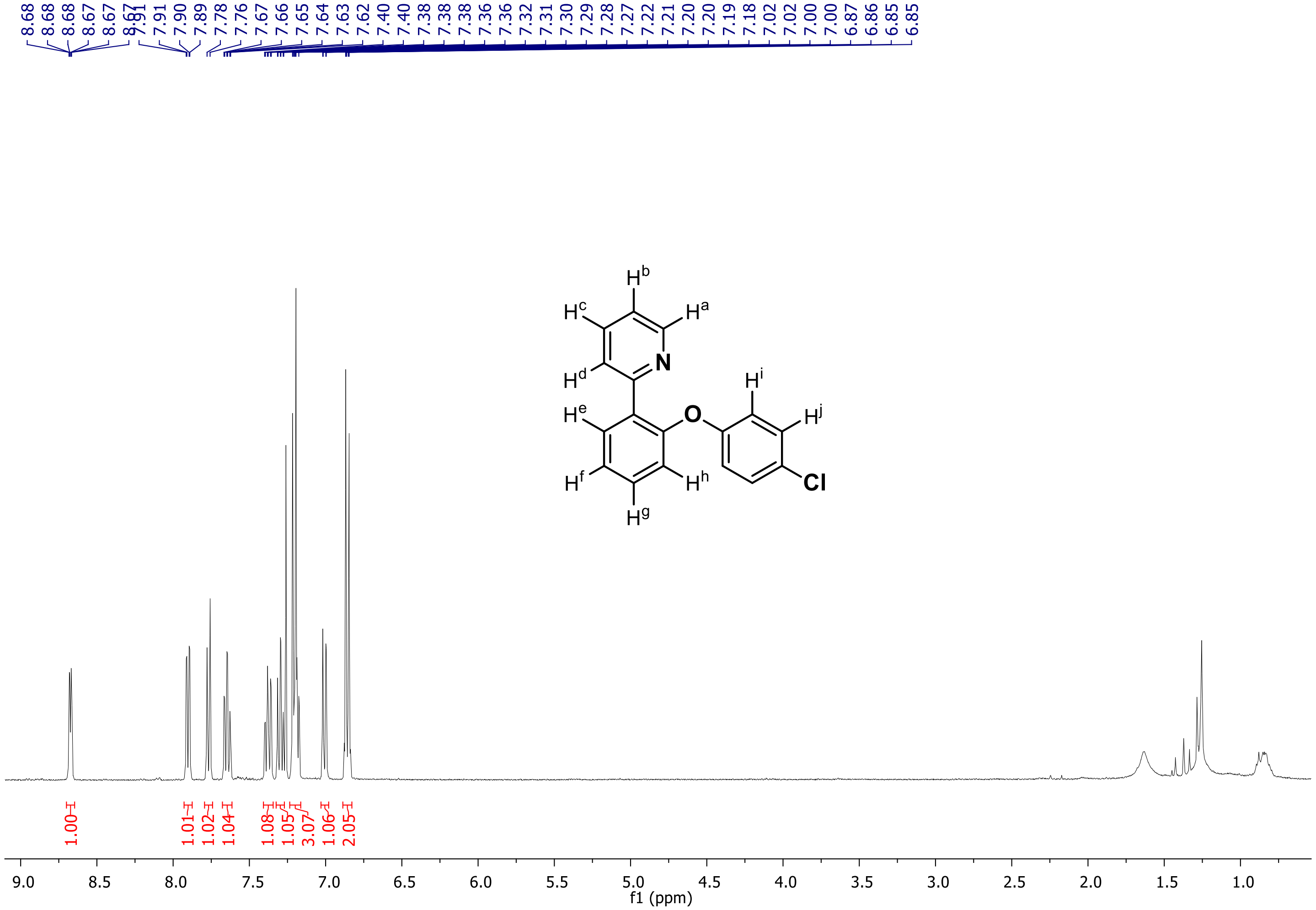

b)

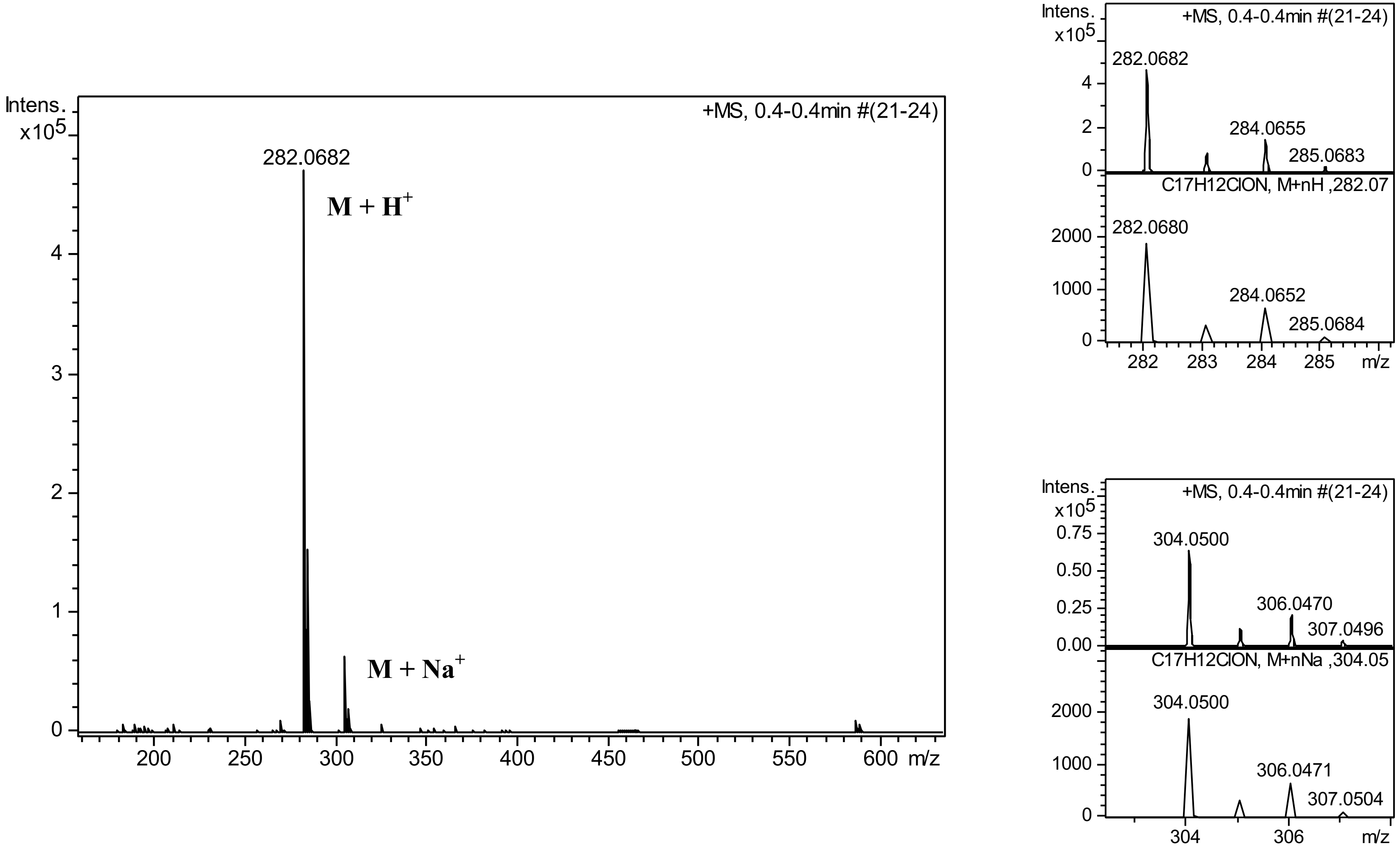
Intens.
x10^5
+MS, 0.4-0.4min #(21-24)
282.0682
M + H+
M + Na+
m/z
284.0655
285.0683
C17H12ClON, M+nH ,282.07
282.0680
284.0652
285.0684
304.0500
306.0470
307.0496
C17H12ClON, M+nNa ,304.05
306.0471
307.0504

Figure S8. a) ^{1}H-NMR spectrum of compound **2aa** in $CDCl_3$, 400 MHz, at 298 K; b) $^{13}C\{^{1}H\}$-NMR spectrum ($CDCl_3$, 100 MHz, 298 K); c) ^{1}H-^{1}H COSY spectrum ($CDCl_3$, 100 MHz, 298 K); d) ^{1}H-^{13}C HSQCed spectrum ($CDCl_3$, 400 MHz, 298 K); e) ^{1}H-^{13}C HMBC spectrum ($CDCl_3$, 400 MHz, 298 K); f) HRMS (ESI-MS) spectrum (*m/z*). Observed HRMS (left) with the theoretical isotope prediction (right).

a)

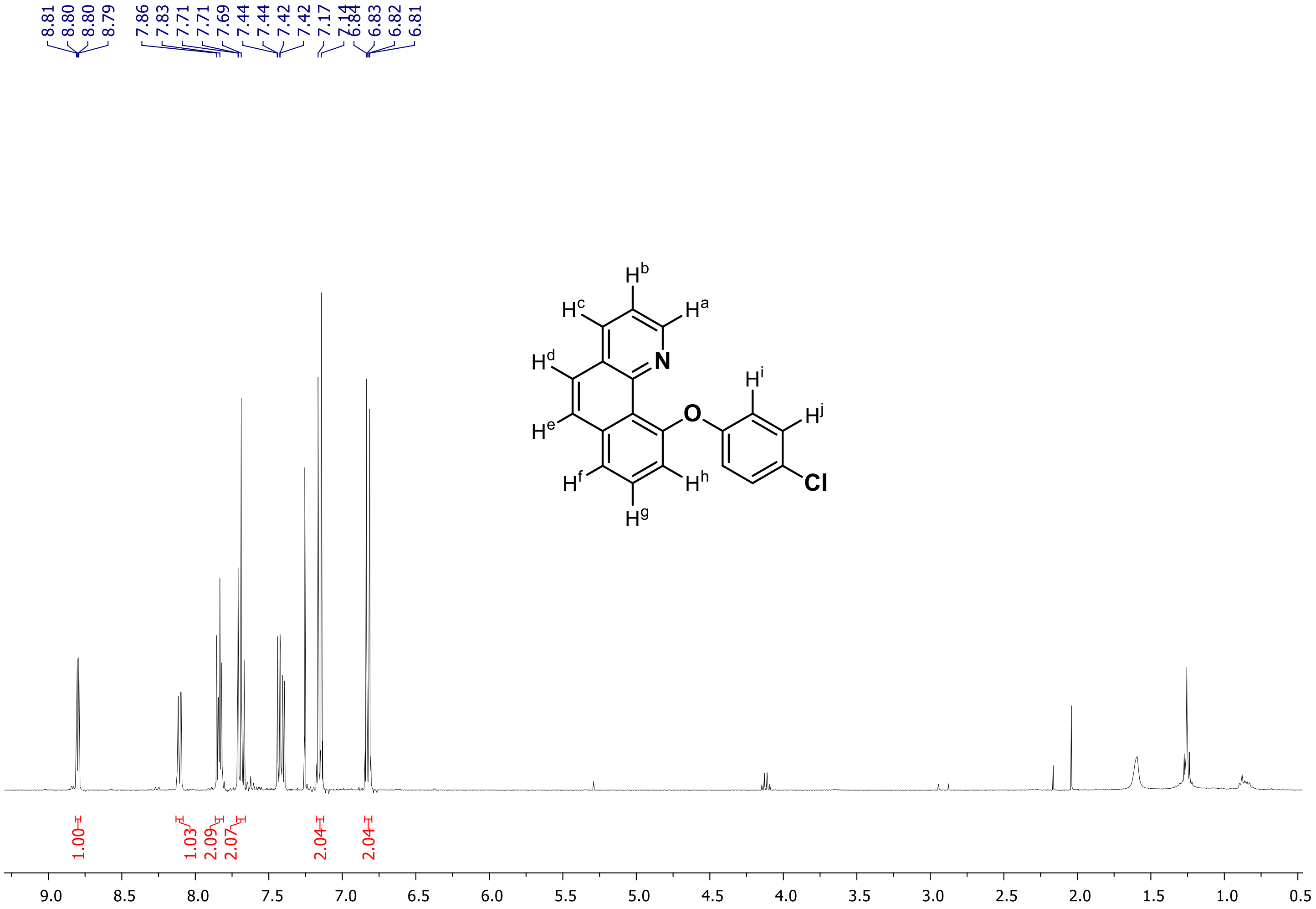

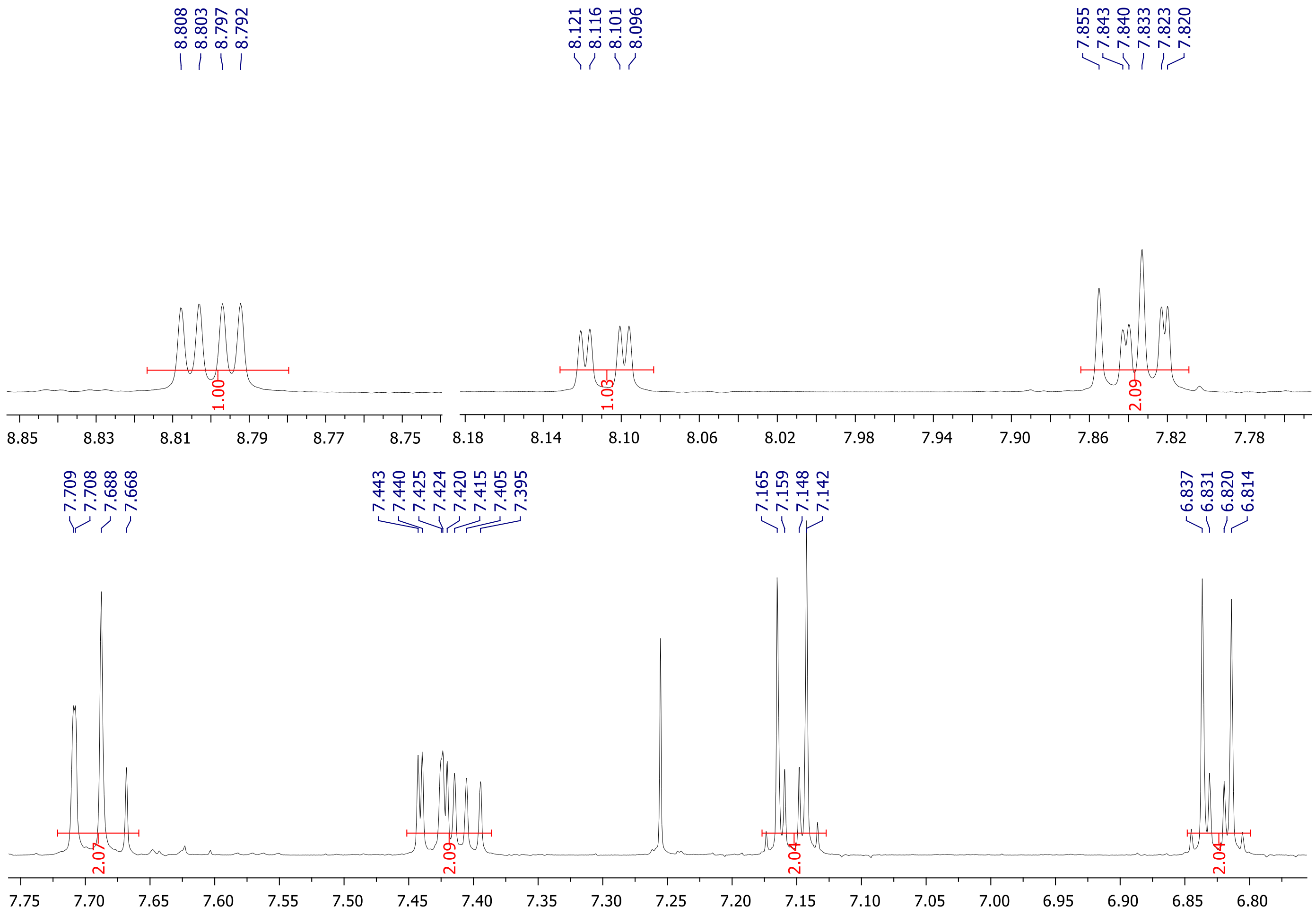

Selected aromatic region

b)

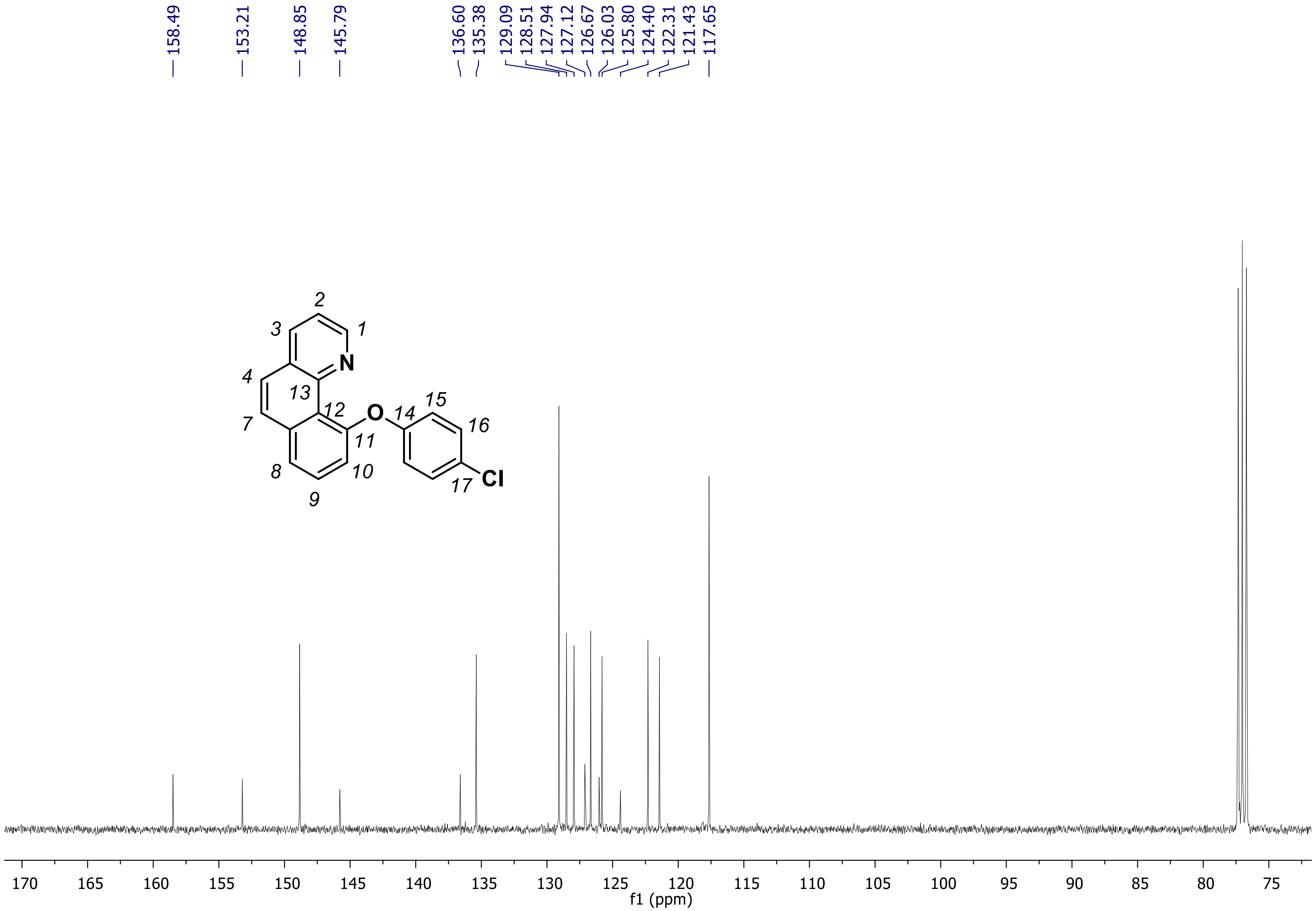

158.49
153.21
148.85
145.79
136.60
135.38
129.09
128.51
127.94
127.12
126.67
126.03
125.80
124.40
122.31
121.43
117.65
2
1
3
N
4
13
12
O
14
15
16
7
11
8
10
9
17
Cl
170
165
160
155
150
145
140
135
130
125
120
115
110
105
100
95
90
85
80
75
f1 (ppm)

c)

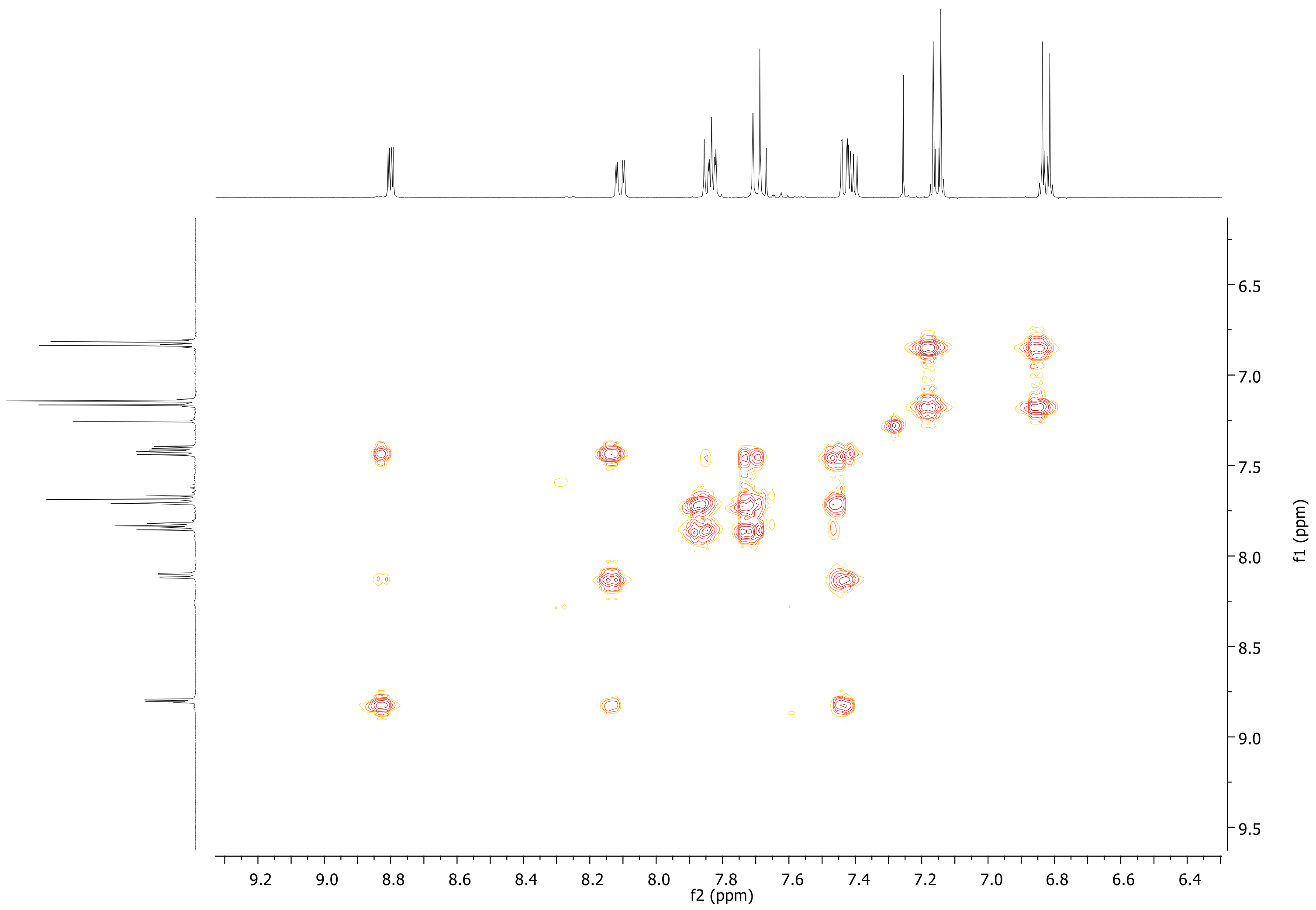

9.2
9.0
8.8
8.6
8.4
8.2
8.0
7.8
7.6
7.4
7.2
7.0
6.8
6.6
6.4
f2 (ppm)
6.5
7.0
7.5
8.0
8.5
9.0
9.5
f1 (ppm)

d)

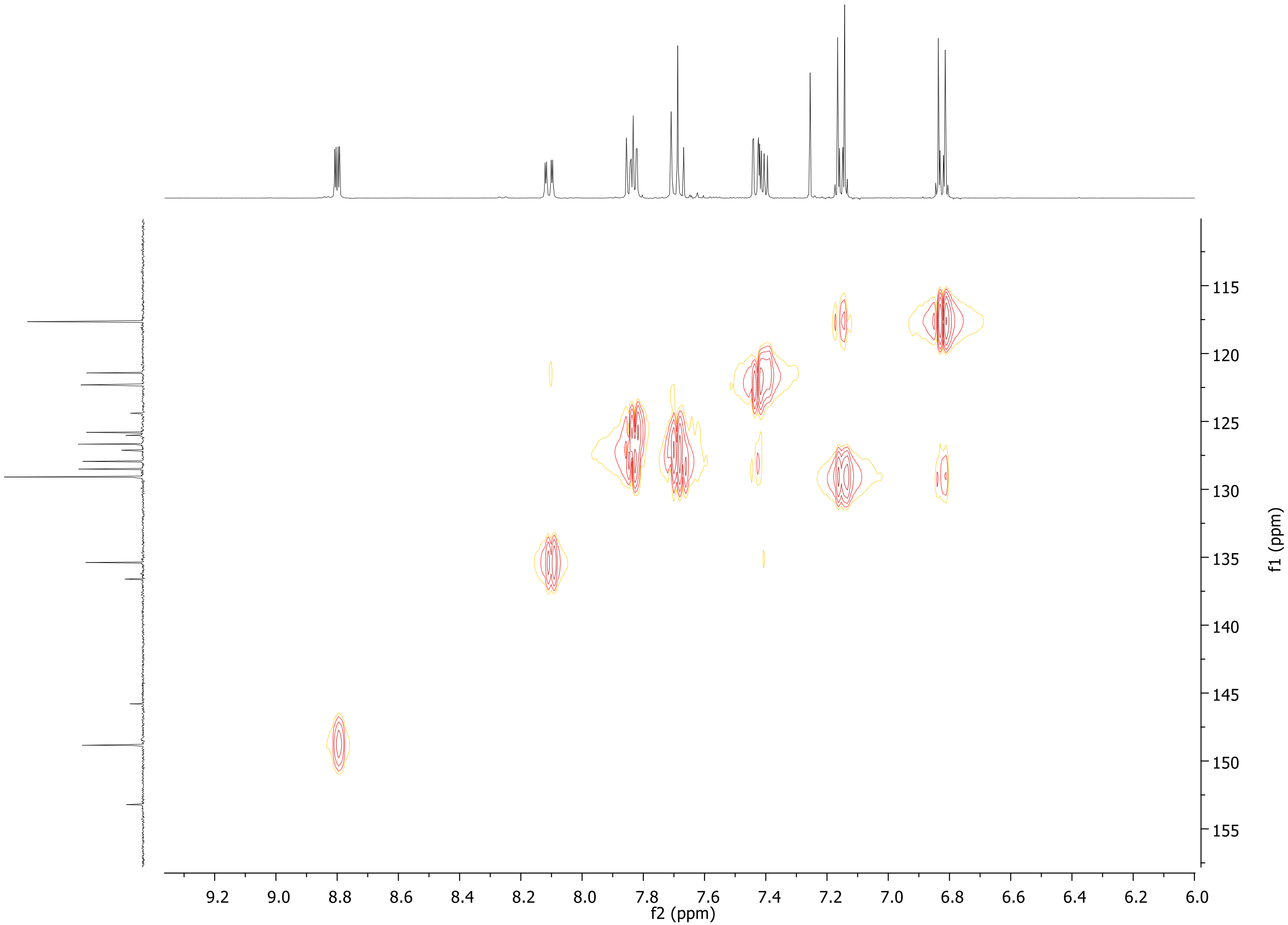

115
120
125
130
135
140
145
150
155
f1 (ppm)
9.2
9.0
8.8
8.6
8.4
8.2
8.0
7.8
7.6
7.4
7.2
7.0
6.8
6.6
6.4
6.2
6.0
f2 (ppm)

e)

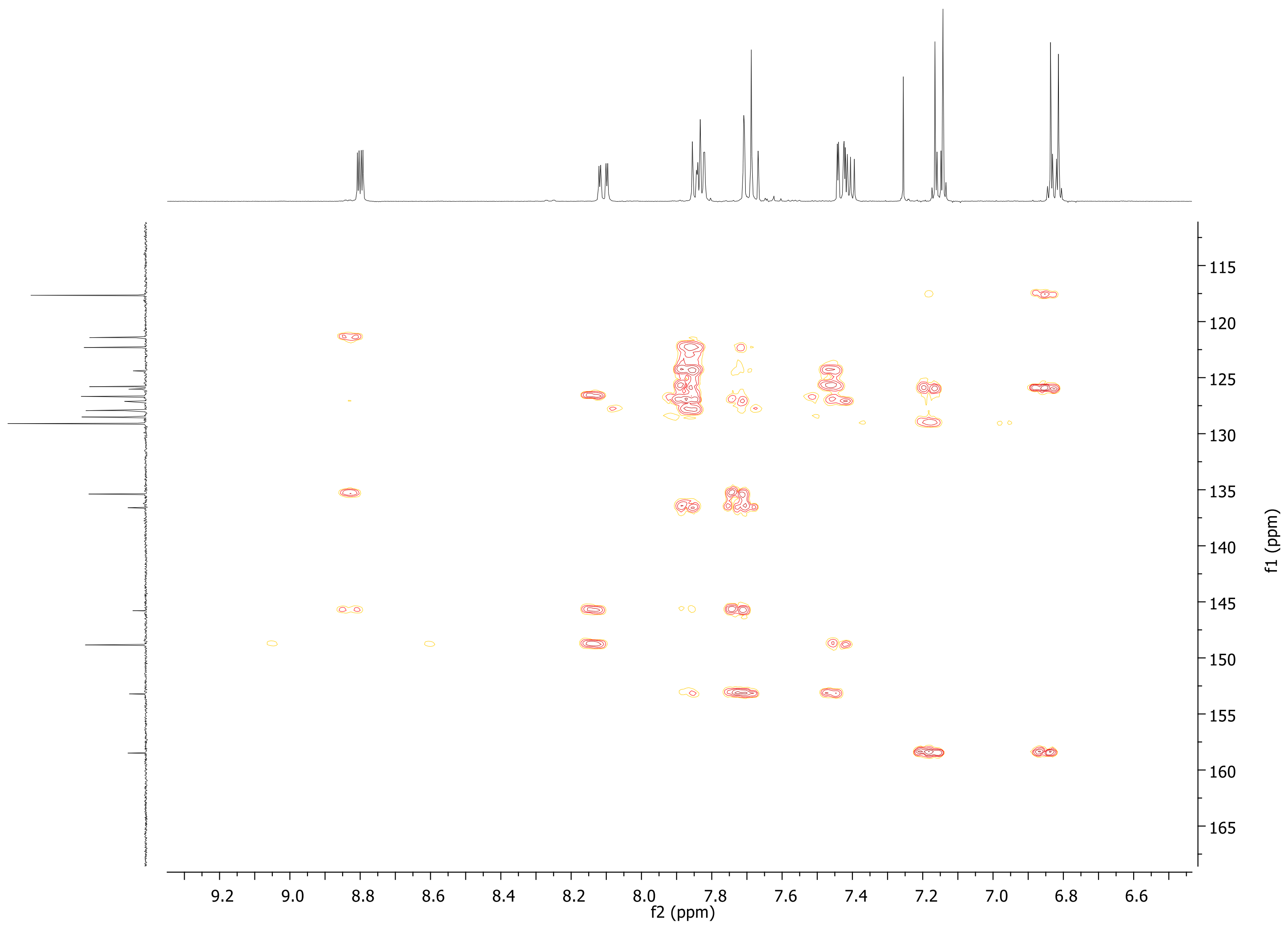

f1 (ppm)
115
120
125
130
135
140
145
150
155
160
165
9.2
9.0
8.8
8.6
8.4
8.2
8.0
7.8
7.6
7.4
7.2
7.0
6.8
6.6
f2 (ppm)

e)

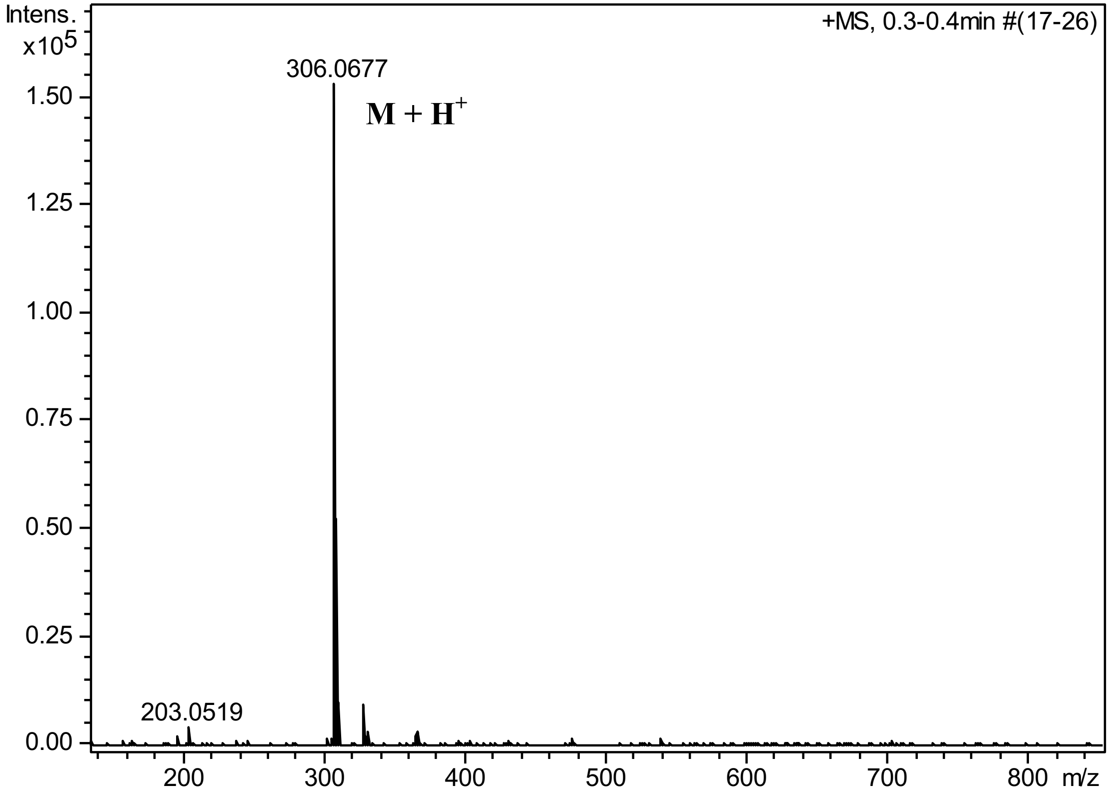

Intens.
x10^5
+MS, 0.3-0.4min #(17-26)
306.0677
M + H+
203.0519
1.50
1.25
1.00
0.75
0.50
0.25
0.00
200
300
400
500
600
700
800
m/z

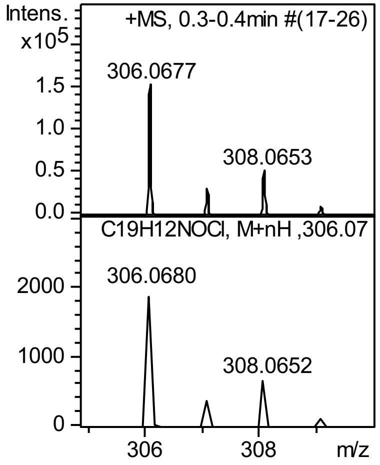

Intens.
x10^5
+MS, 0.3-0.4min #(17-26)
306.0677
308.0653
1.5
1.0
0.5
0.0
C19H12NOCl, M+nH ,306.07
306.0680
308.0652
2000
1000
0
306
308
m/z

Figure S9. a) ^{1}H-NMR spectrum of compound **1ab** in $CDCl_3$, 400 MHz, at 298 K; b) HRMS (ESI-MS) spectrum (*m/z*). Observed HRMS (left) with the theoretical isotope prediction (right).

a)

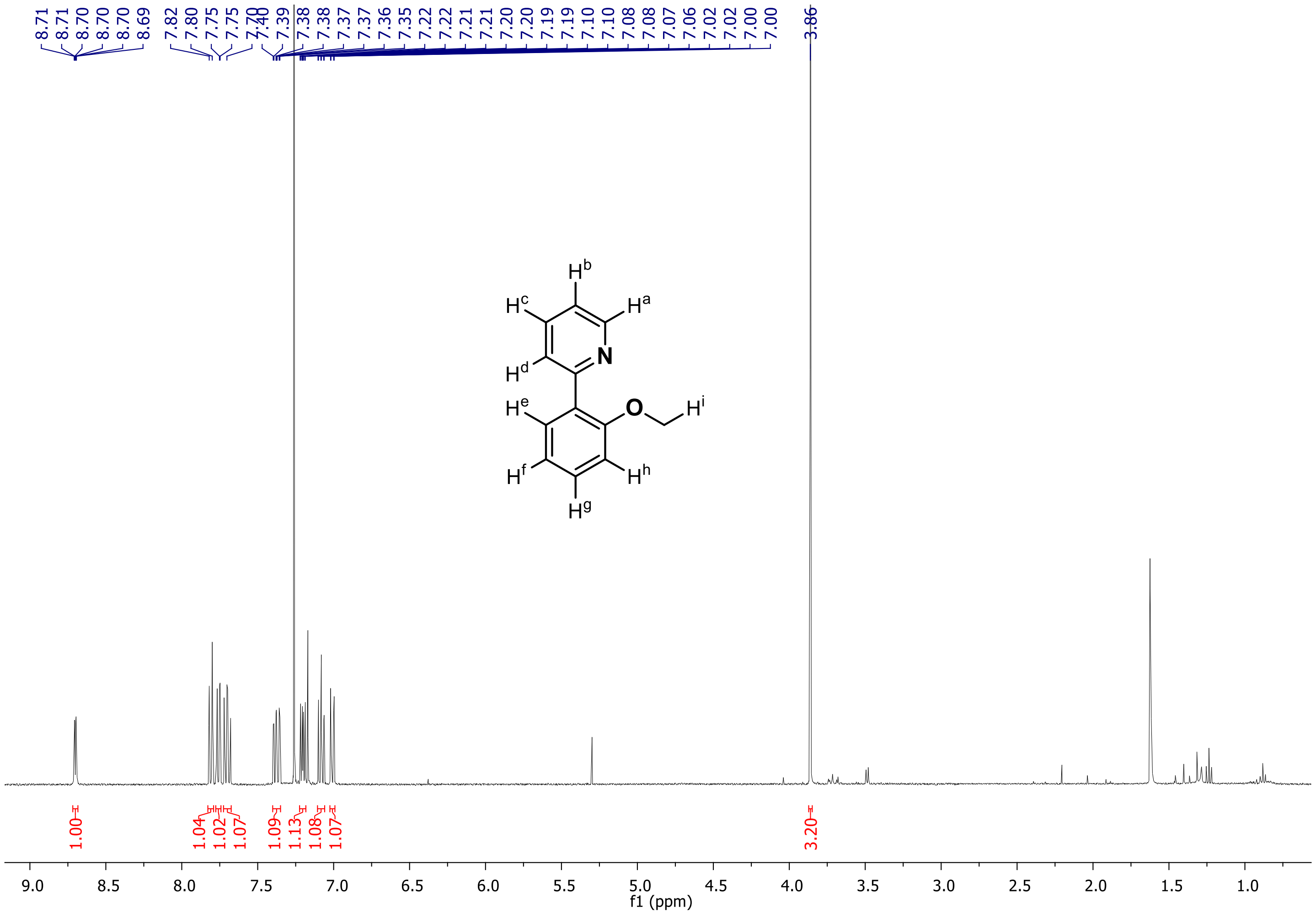

b)

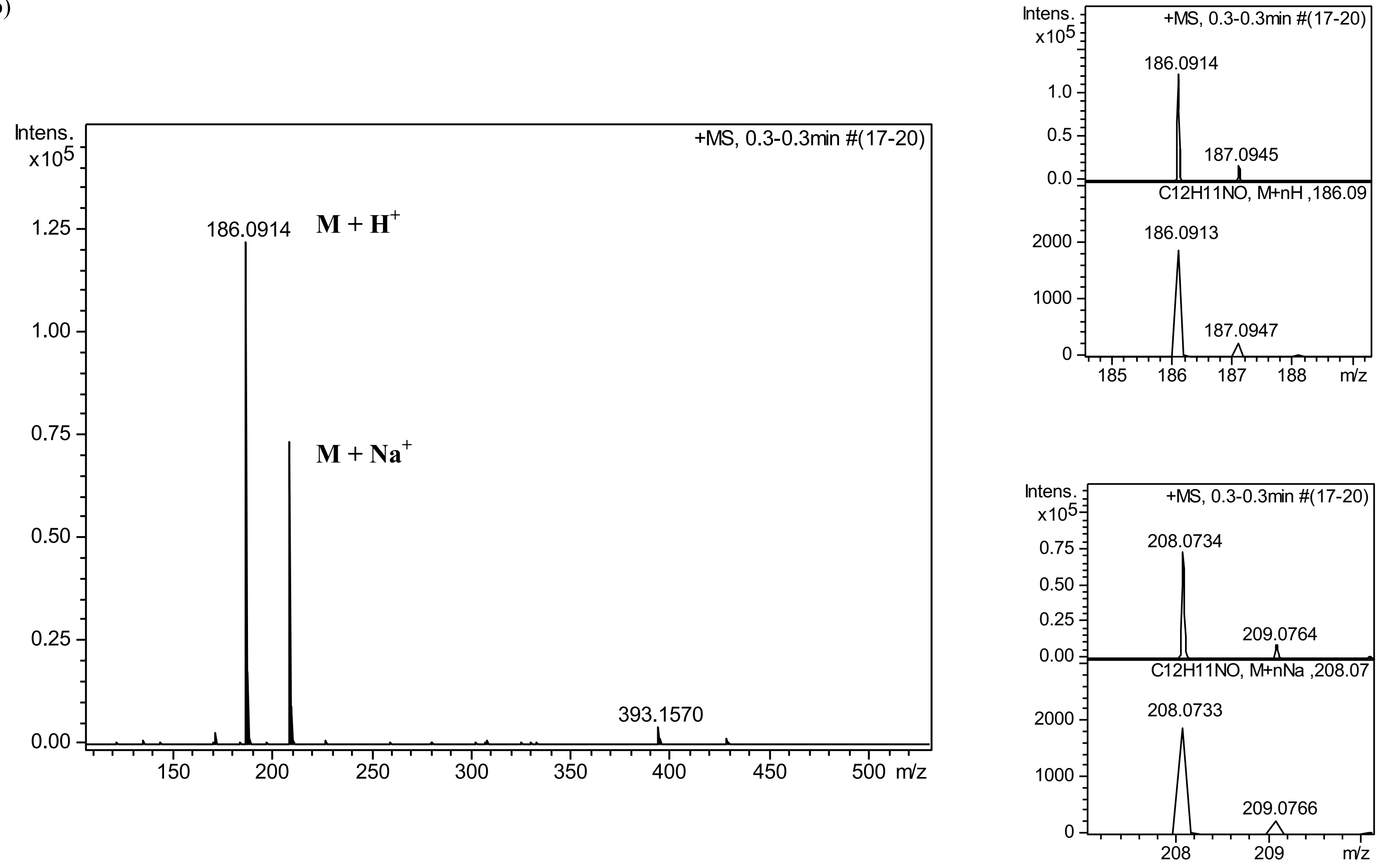
Intens.
x10^5
+MS, 0.3-0.3min #(17-20)
186.0914
M + H+
M + Na+
393.1570
m/z
186.0914
187.0945
C12H11NO, M+nH ,186.09
186.0913
187.0947
208.0734
209.0764
C12H11NO, M+nNa ,208.07
208.0733
209.0766

Figure S10. a) ^{1}H-NMR spectrum of compound **1ac** in $CDCl_3$, 400 MHz, at 298 K; b) HRMS (ESI-MS) spectrum (*m/z*). Observed HRMS (left) with the theoretical isotope prediction (right).

a)

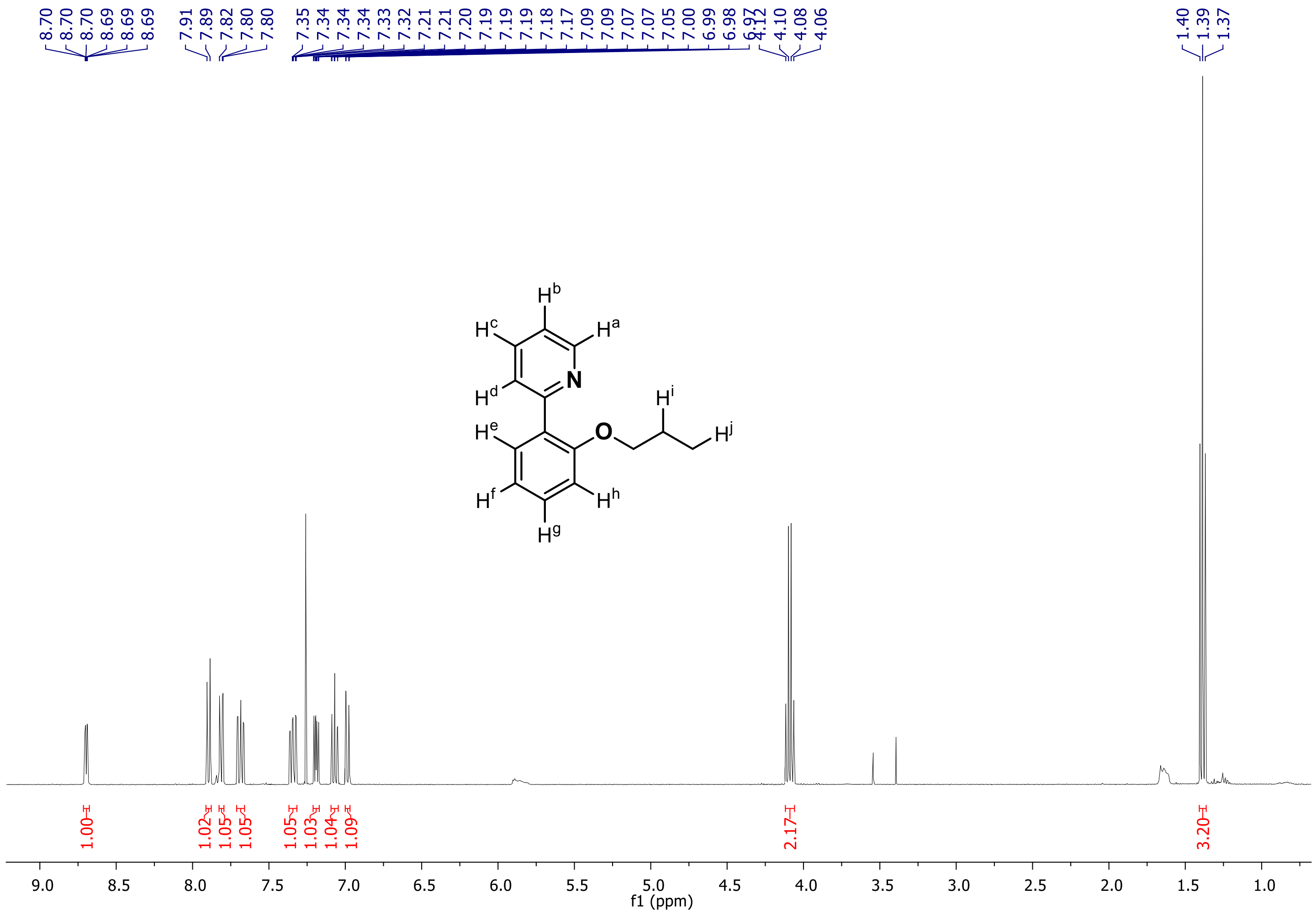

b)

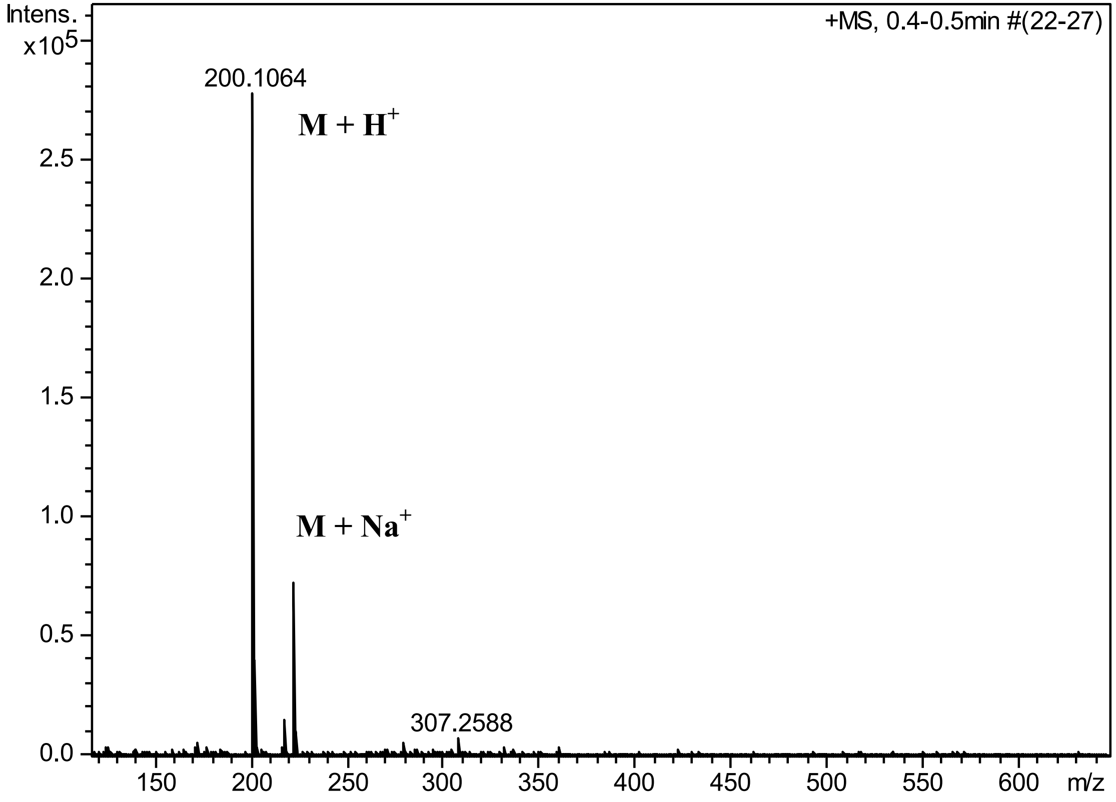

Intens.
x10^5
+MS, 0.4-0.5min #(22-27)
200.1064
M + H+
M + Na+
307.2588
0.0
0.5
1.0
1.5
2.0
2.5
150
200
250
300
350
400
450
500
550
600
m/z

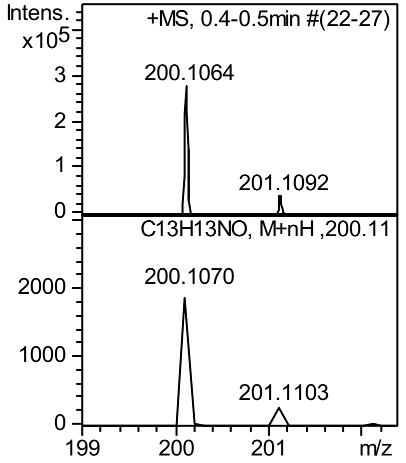

Intens.
x10^5
+MS, 0.4-0.5min #(22-27)
200.1064
201.1092
C13H13NO, M+nH ,200.11
200.1070
201.1103
199
200
201
m/z

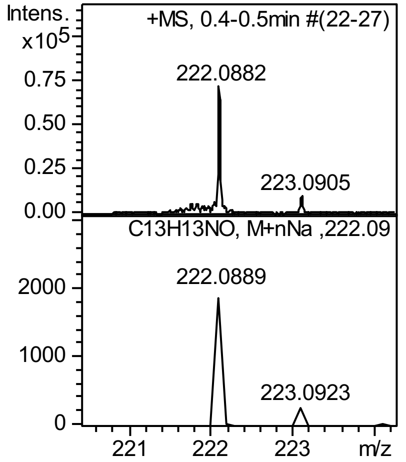

Intens.
x10^5
+MS, 0.4-0.5min #(22-27)
222.0882
223.0905
C13H13NO, M+nNa ,222.09
222.0889
223.0923
221
222
223
m/z

Figure S11. a) ^{1}H-NMR spectrum of compound **1af** in $CDCl_3$, 400 MHz, at 298 K; b) HRMS (ESI-MS) spectrum (*m/z*). Observed HRMS (left) with the theoretical isotope prediction (right).

a)

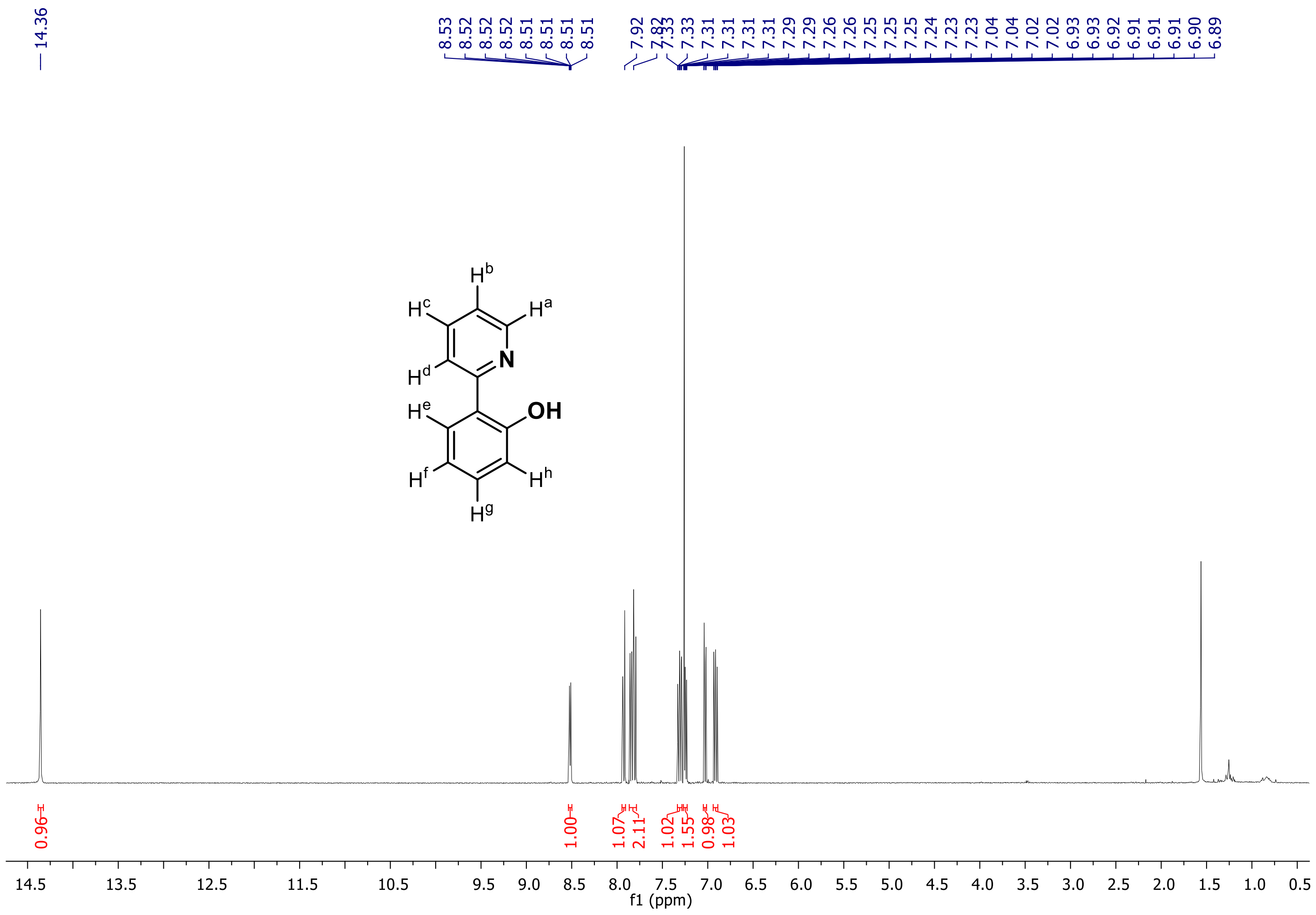

b)

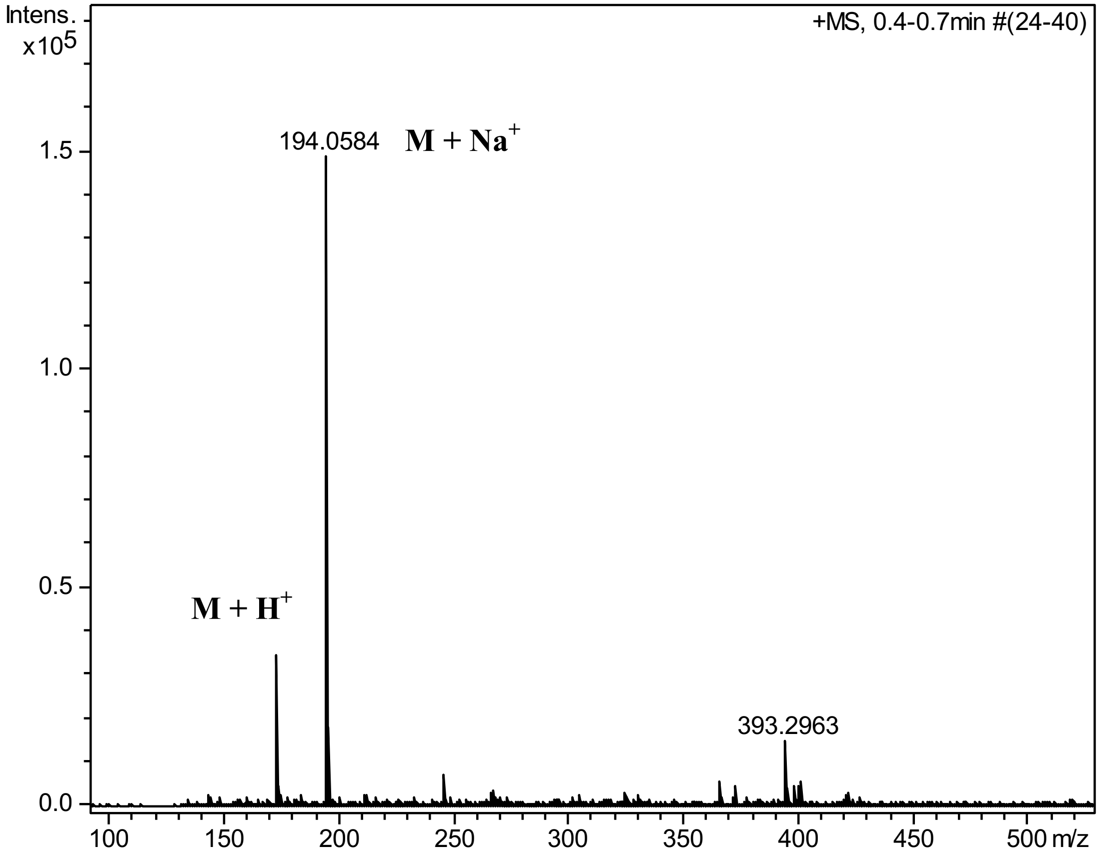

Intens.
x10^5
+MS, 0.4-0.7min #(24-40)
194.0584
M + Na+
M + H+
393.2963
0.0
0.5
1.0
1.5
100
150
200
250
300
350
400
450
500 m/z

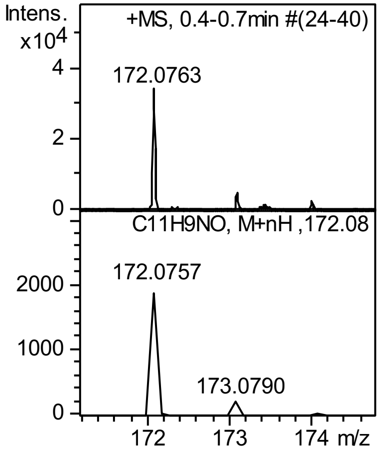

Intens.
x10^4
+MS, 0.4-0.7min #(24-40)
172.0763
C11H9NO, M+nH ,172.08
172.0757
173.0790
172
173
174 m/z

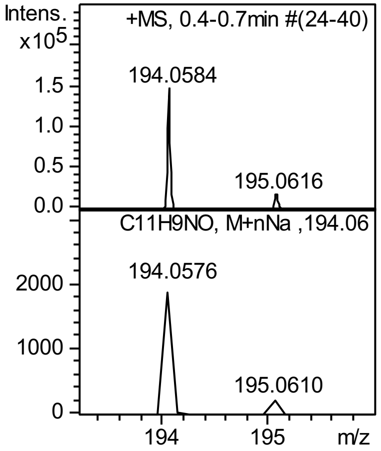

Intens.
x10^5
+MS, 0.4-0.7min #(24-40)
194.0584
195.0616
C11H9NO, M+nNa ,194.06
194.0576
195.0610
194
195
m/z

Figure S12. a) ^{1}H-NMR spectrum of compound **1ag** in $CDCl_3$, 400 MHz, at 298 K; b) $^{13}C\{^1H\}$-NMR spectrum ($CDCl_3$, 100 MHz, 298 K); c) ^1H-^1H COSY spectrum ($CDCl_3$, 100 MHz, 298 K); d) ^1H-^{13}C HSQCed spectrum ($CDCl_3$, 400 MHz, 298 K); e) ^1H-^{13}C HMBC spectrum ($CDCl_3$, 400 MHz, 298 K); f) HRMS (ESI-MS) spectrum (*m/z*). Observed HRMS (left) with the theoretical isotope prediction (right).

a)

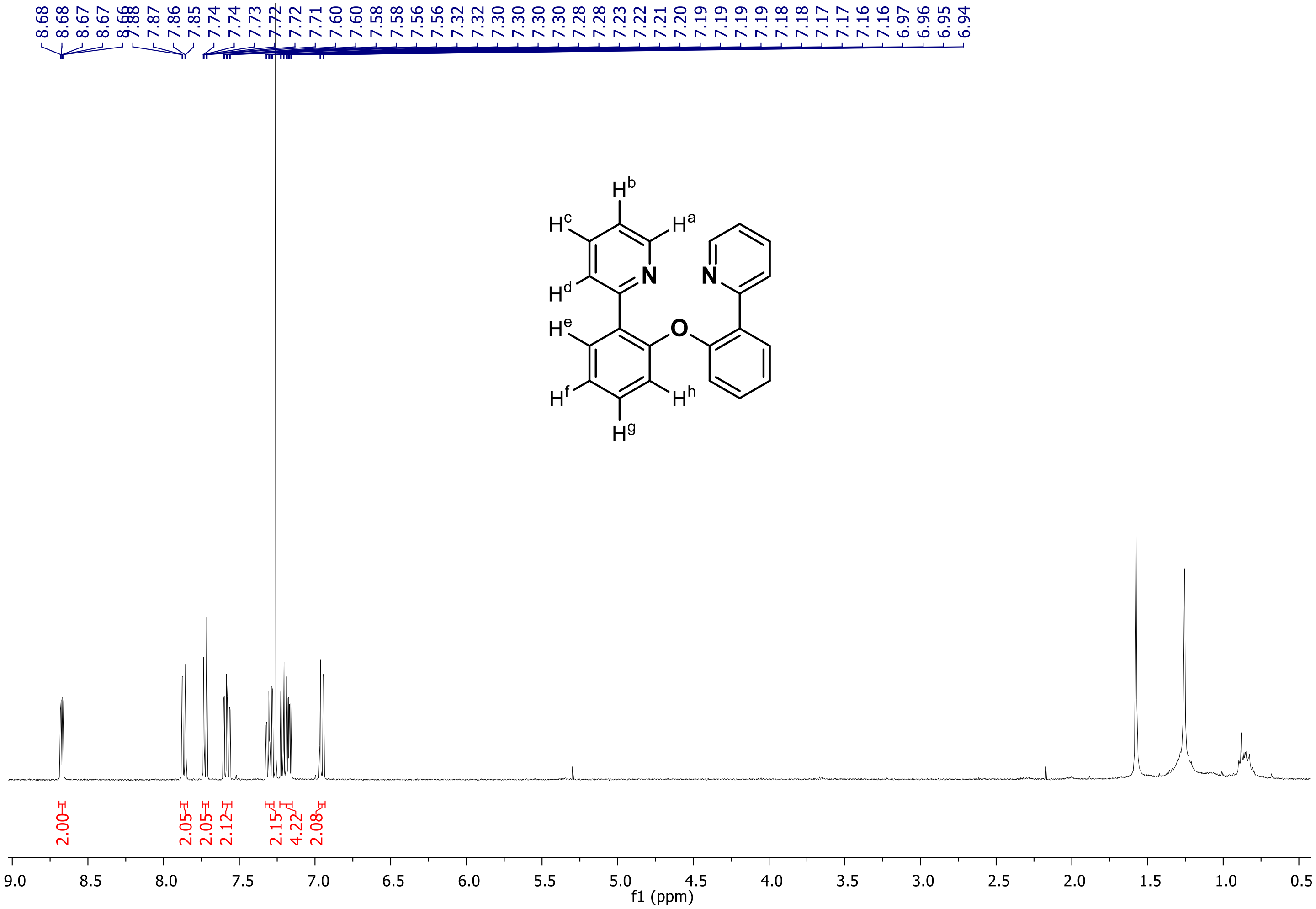

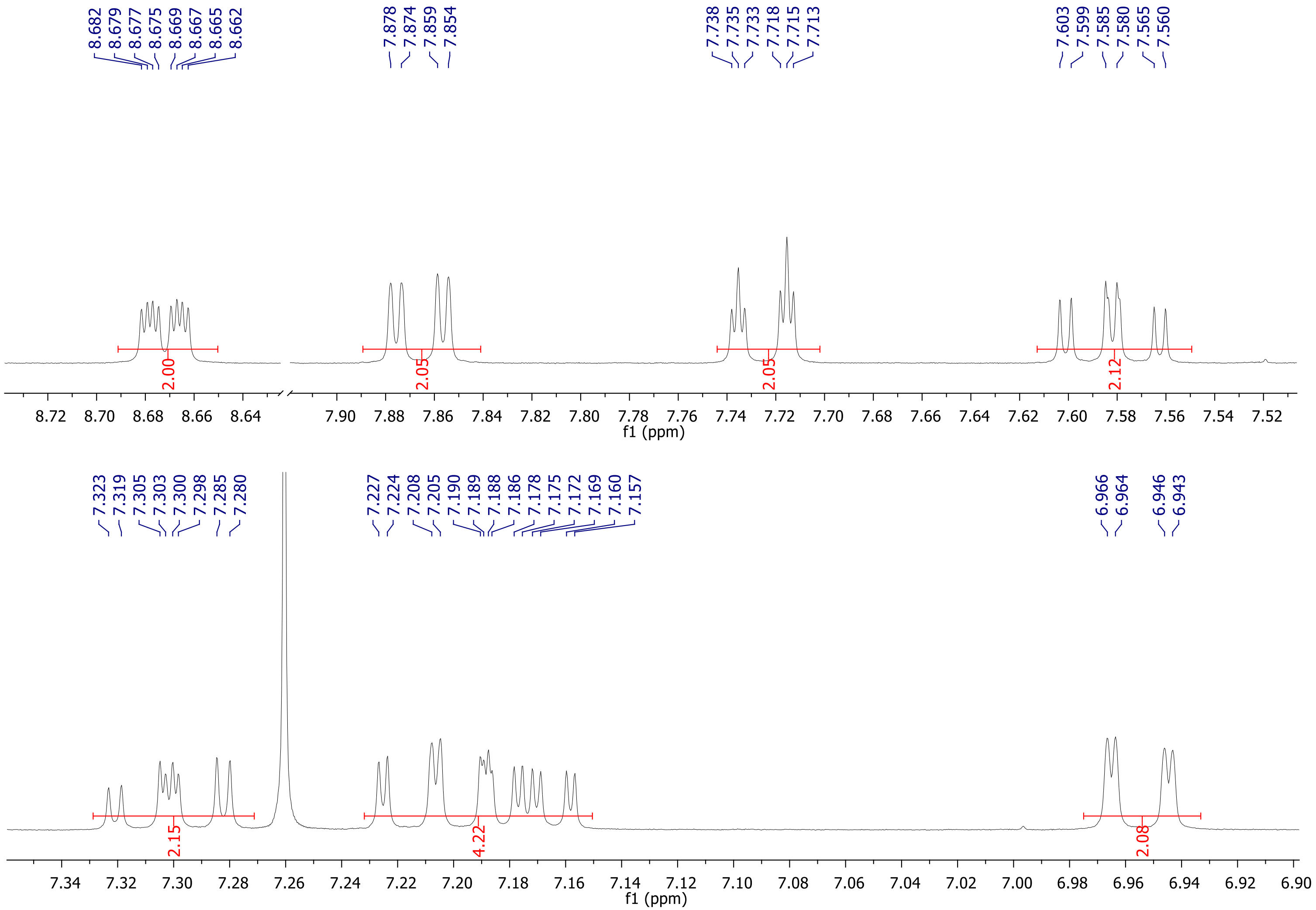

Selected aromatic region

b)

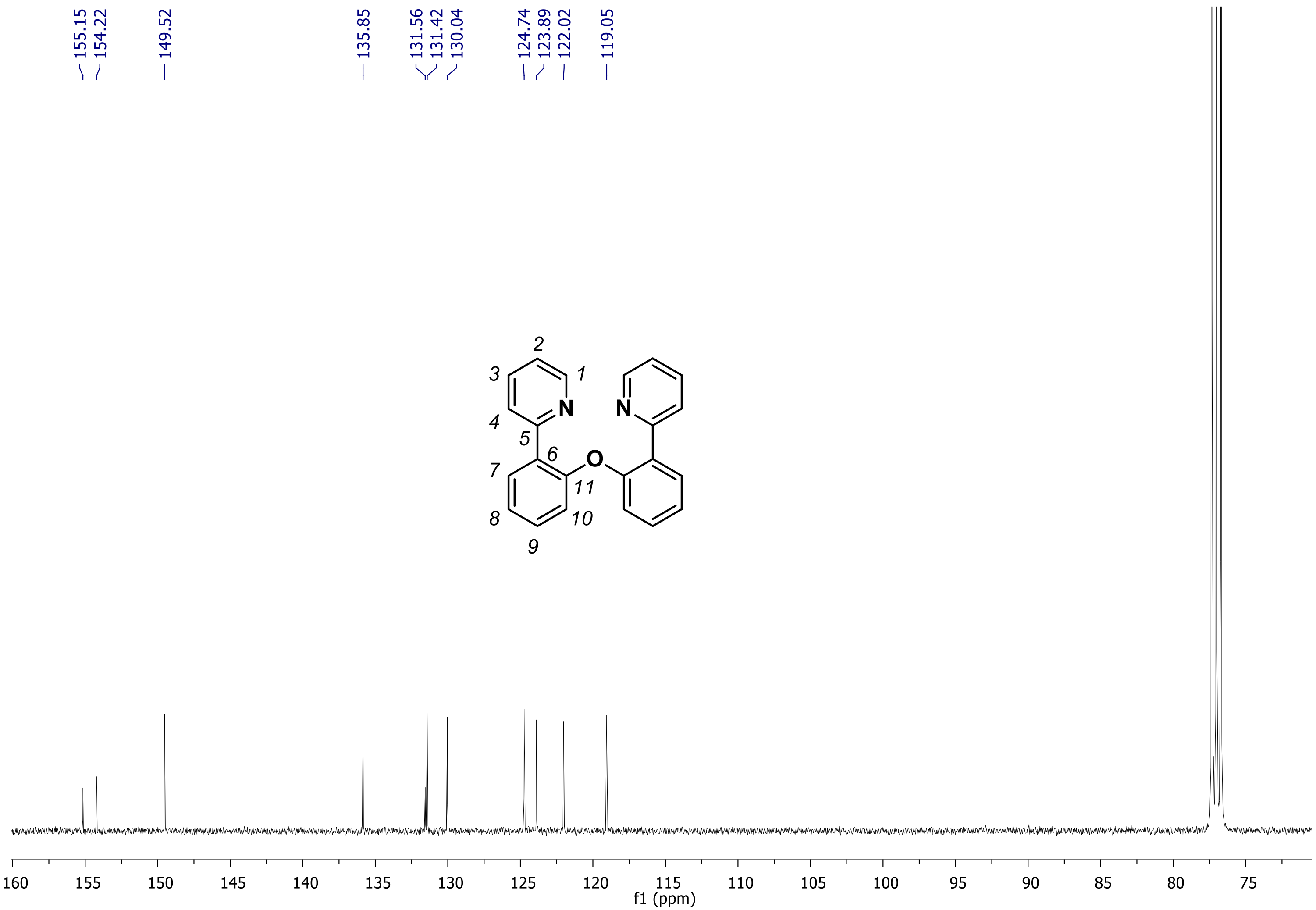
155.15
154.22
149.52
135.85
131.56
131.42
130.04
124.74
123.89
122.02
119.05
2
3
1
4
N
N
5
6
O
7
11
8
10
9
160
155
150
145
140
135
130
125
120
115
110
105
100
95
90
85
80
75
f1 (ppm)

c)

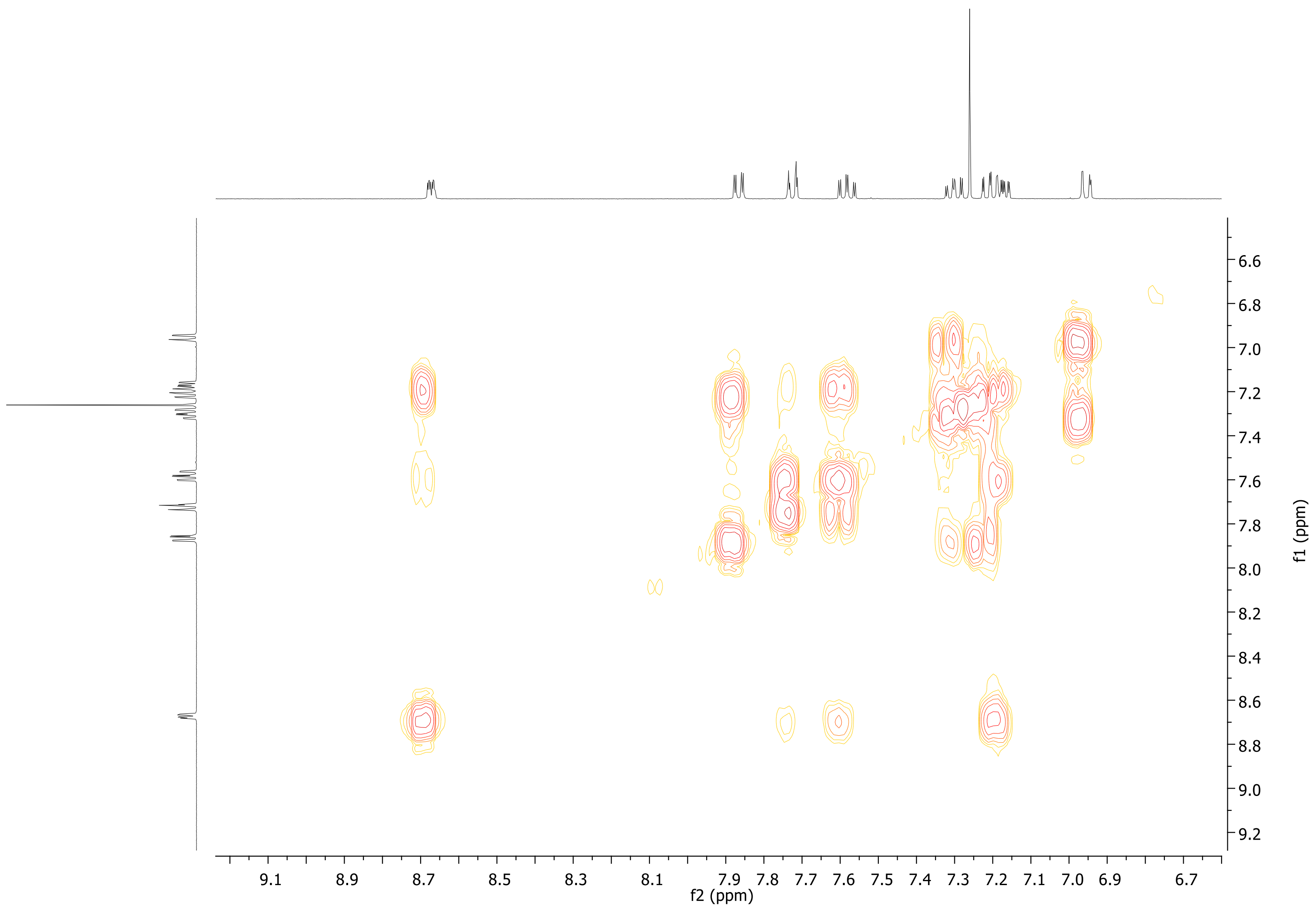

9.1
8.9
8.7
8.5
8.3
8.1
7.9
7.8
7.7
7.6
7.5
7.4
7.3
7.2
7.1
7.0
6.9
6.7
f2 (ppm)
6.6
6.8
7.0
7.2
7.4
7.6
7.8
8.0
8.2
8.4
8.6
8.8
9.0
9.2
f1 (ppm)

d)

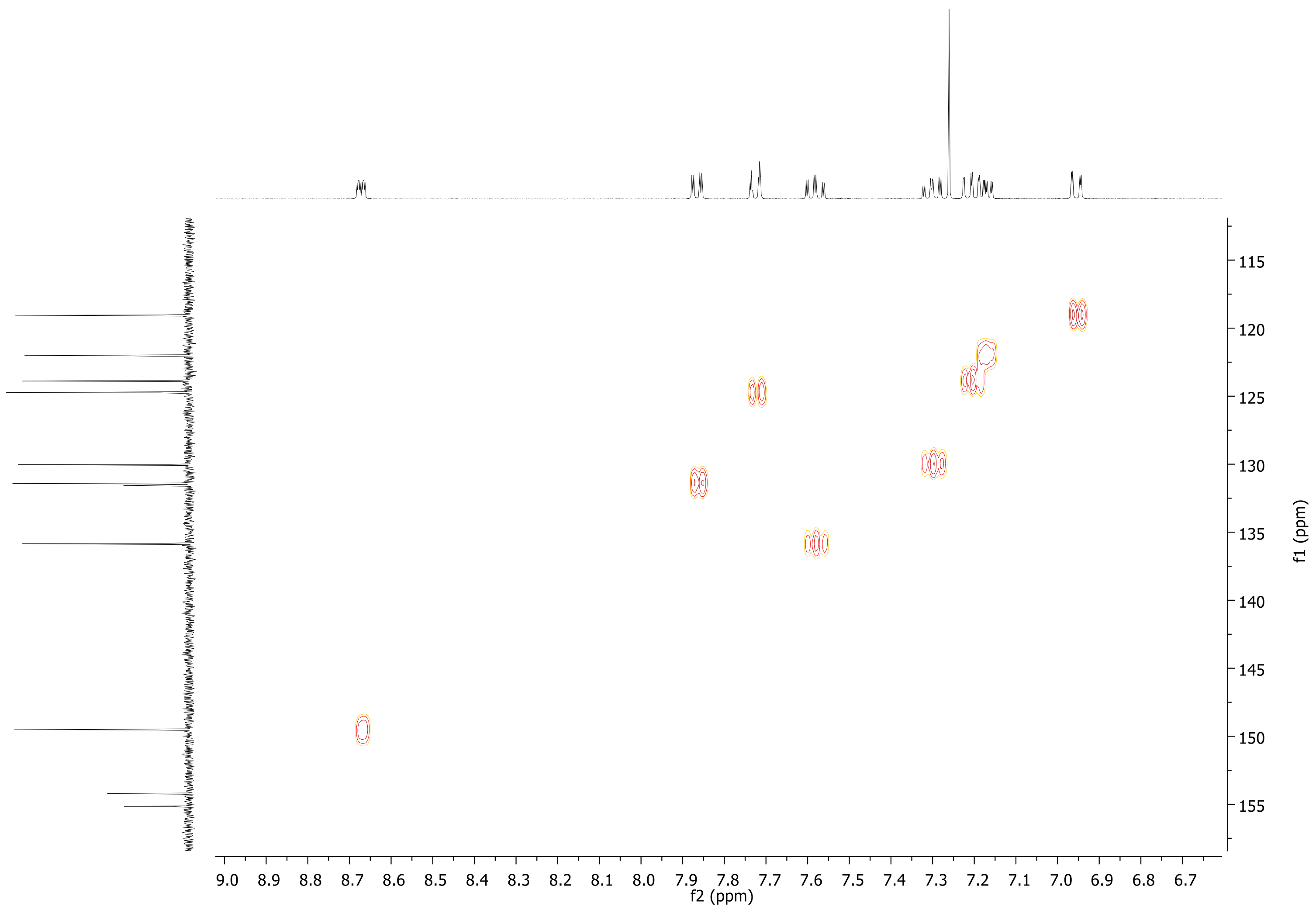

9.0
8.9
8.8
8.7
8.6
8.5
8.4
8.3
8.2
8.1
8.0
7.9
7.8
7.7
7.6
7.5
7.4
7.3
7.2
7.1
7.0
6.9
6.8
6.7
f2 (ppm)
115
120
125
130
135
140
145
150
155
f1 (ppm)

e)

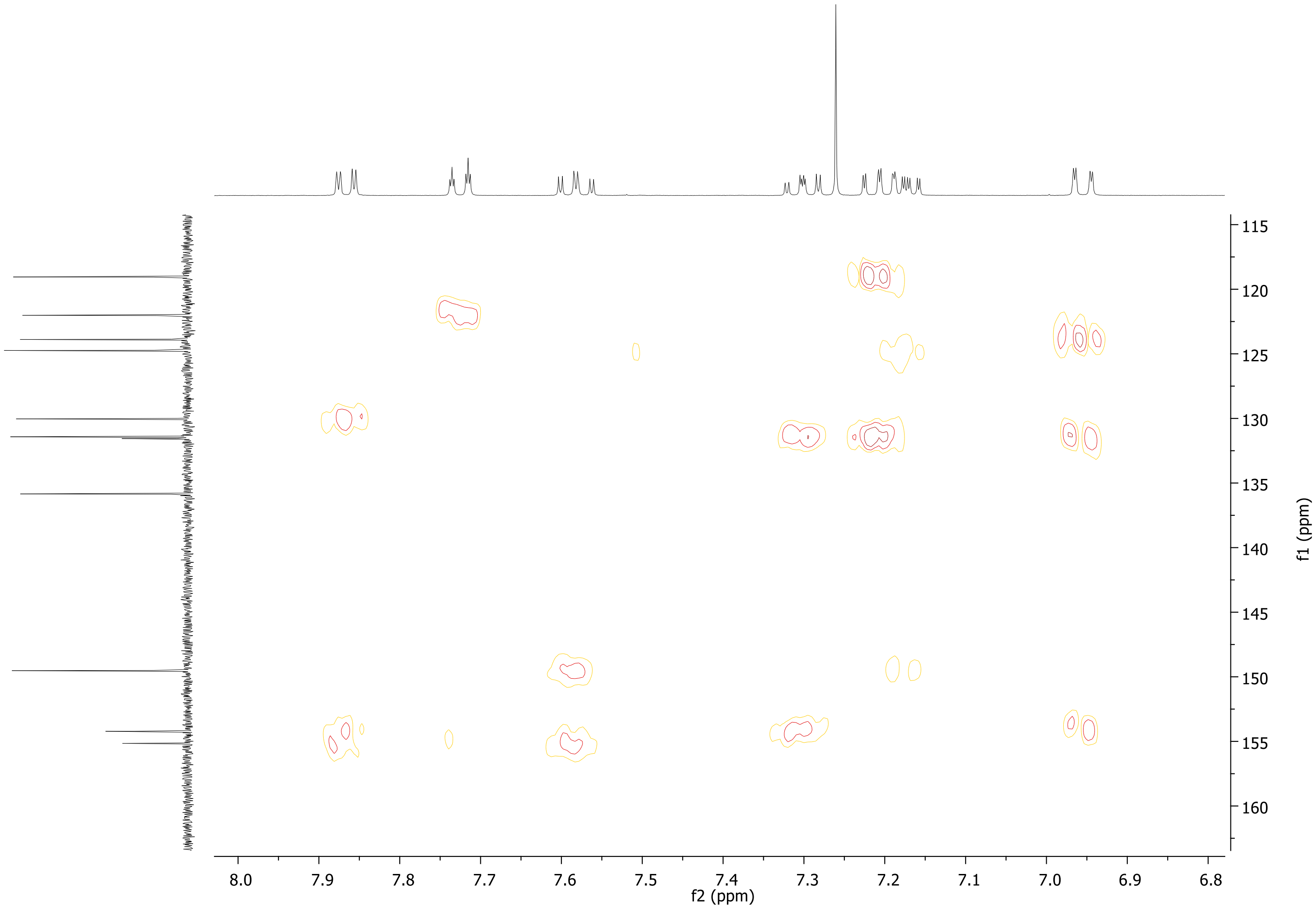

115
120
125
130
135
140
145
150
155
160
f1 (ppm)
8.0
7.9
7.8
7.7
7.6
7.5
7.4
7.3
7.2
7.1
7.0
6.9
6.8
f2 (ppm)

f)

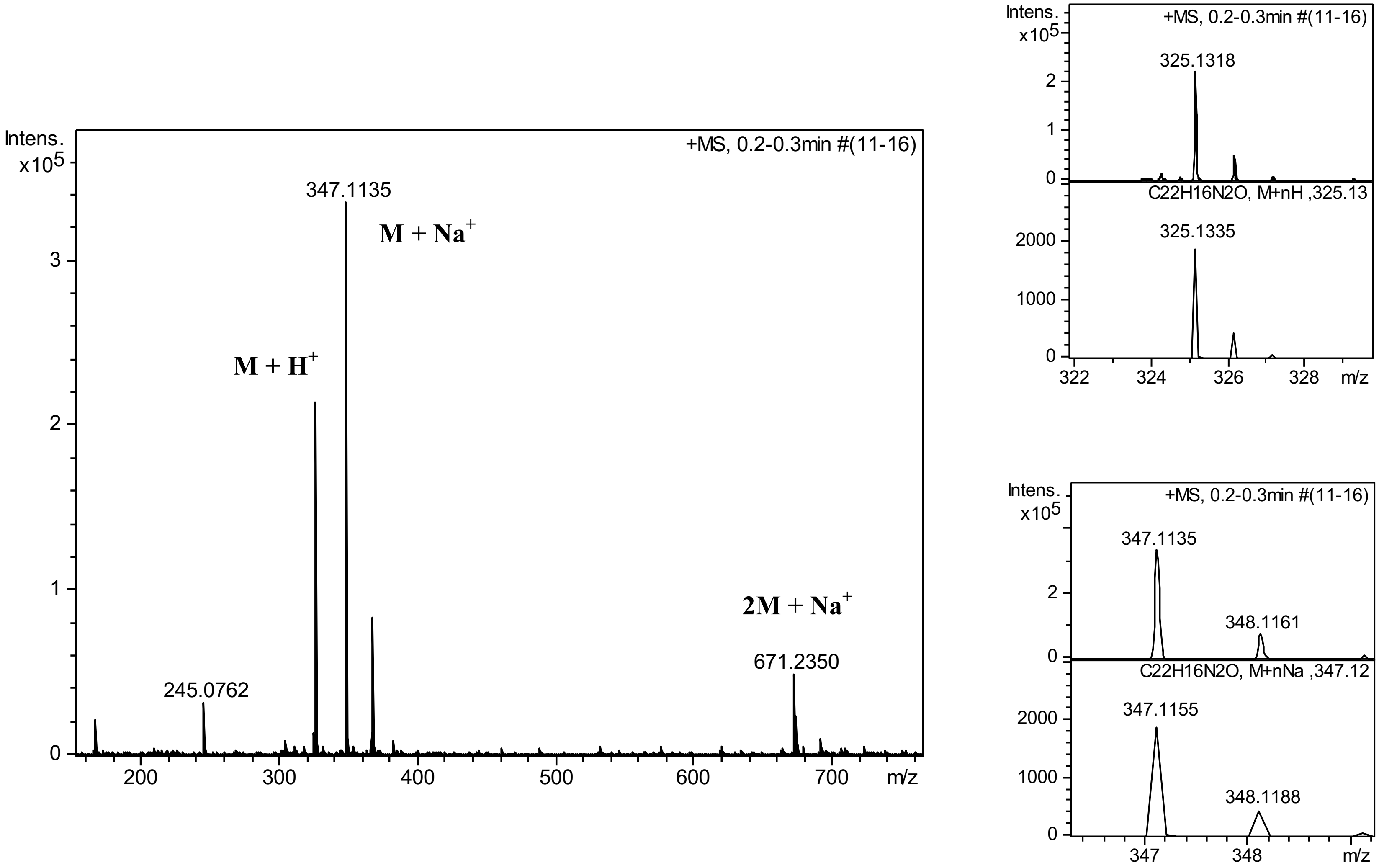
Intens.
x10^5
+MS, 0.2-0.3min #(11-16)
347.1135
M + Na+
M + H+
2M + Na+
671.2350
245.0762
m/z
+MS, 0.2-0.3min #(11-16)
325.1318
C22H16N2O, M+nH ,325.13
325.1335
+MS, 0.2-0.3min #(11-16)
347.1135
348.1161
C22H16N2O, M+nNa ,347.12
347.1155
348.1188

Figure S13. a) ^{1}H-NMR spectrum of compound **1ah** in $CDCl_3$, 400 MHz, at 298 K; b) $^{13}C\{^{1}H\}$-NMR spectrum ($CDCl_3$, 100 MHz, 298 K); c) ^{1}H-^{1}H COSY spectrum ($CDCl_3$, 100 MHz, 298 K); d) ^{1}H-^{13}C HSQCed spectrum ($CDCl_3$, 400 MHz, 298 K); e) ^{1}H-^{13}C HMBC spectrum ($CDCl_3$, 400 MHz, 298 K); f) HRMS (ESI-MS) spectrum (*m/z*). Observed HRMS (left) with the theoretical isotope prediction (right).

a)

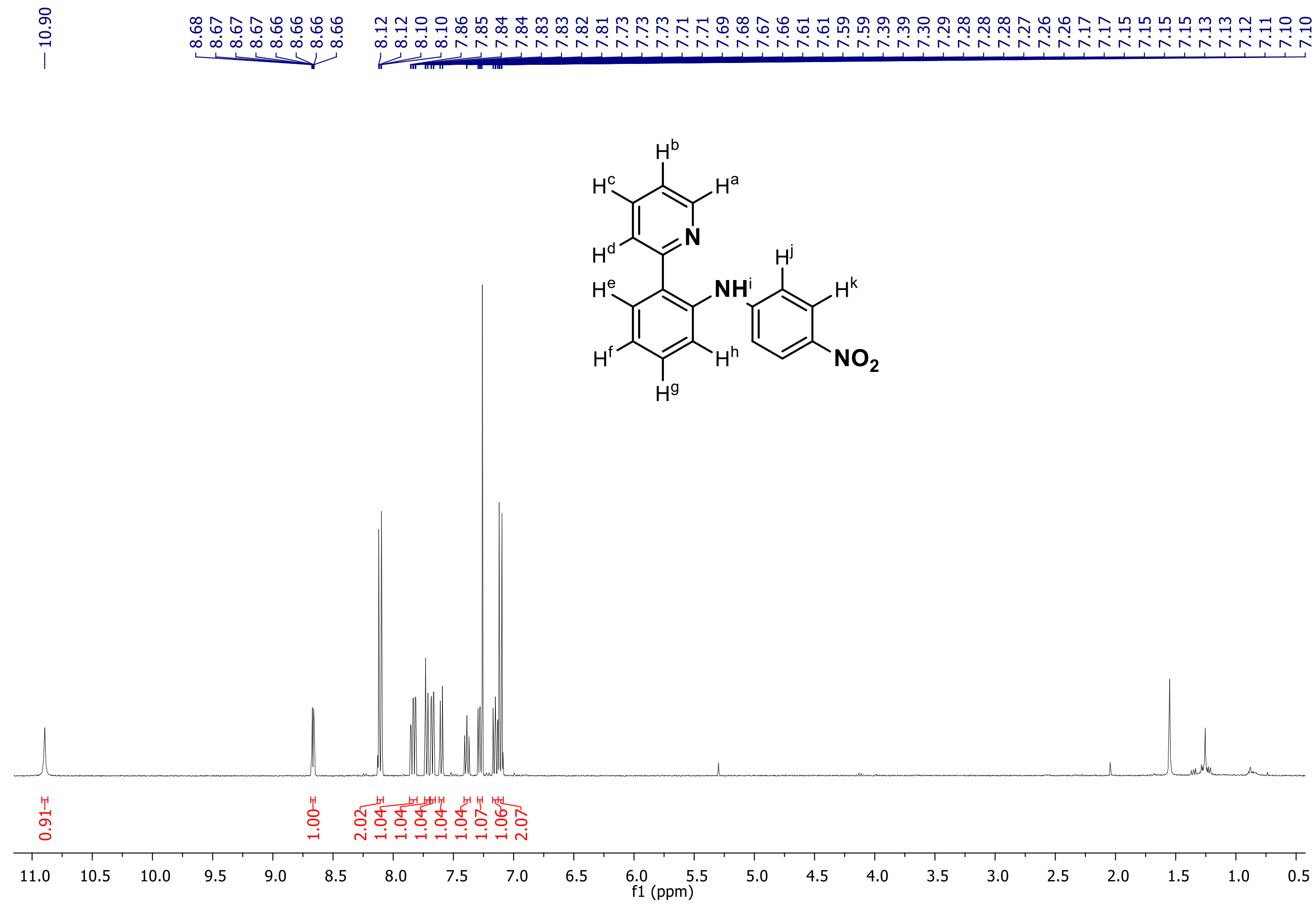

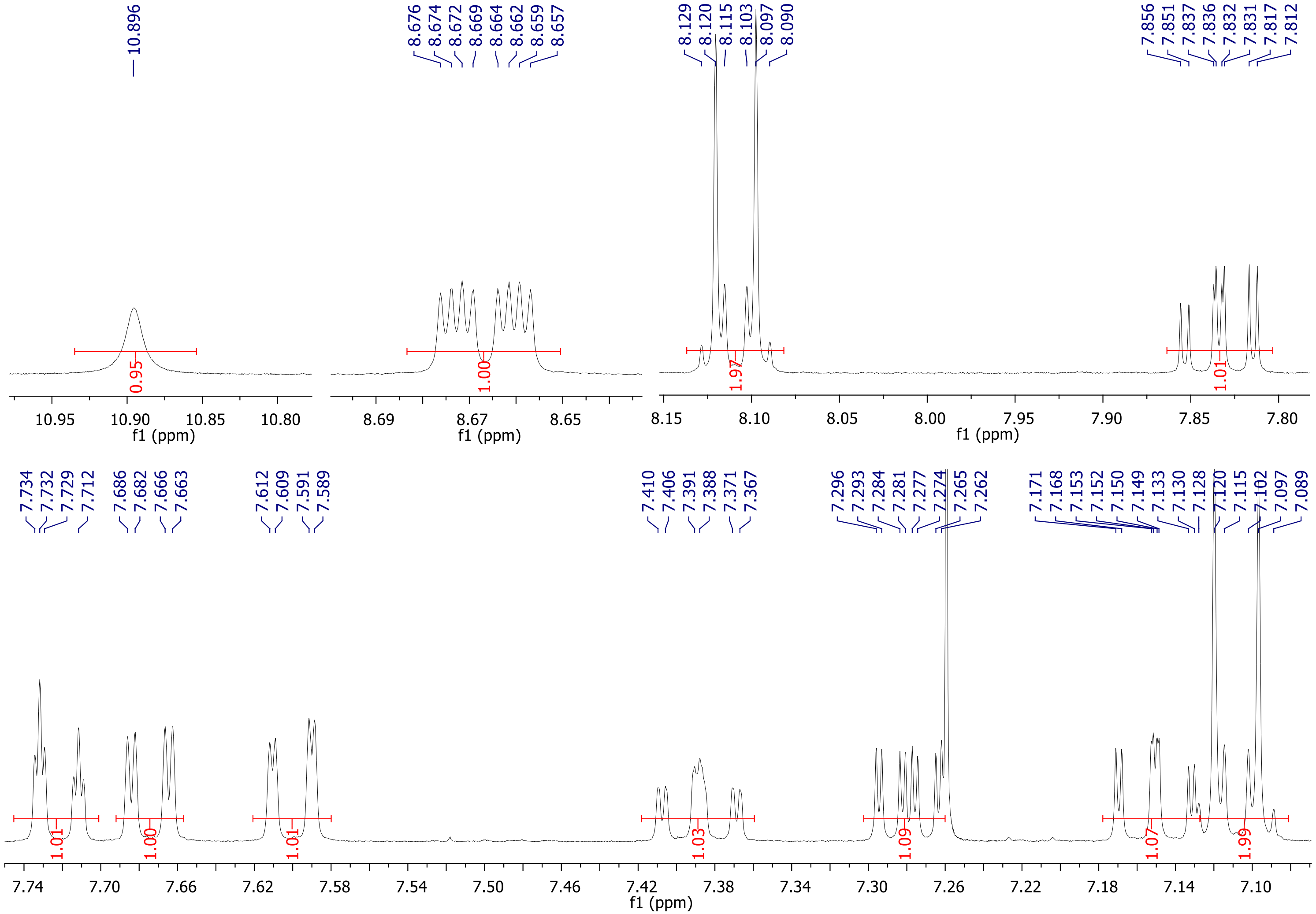

Selected aromatic region

b)

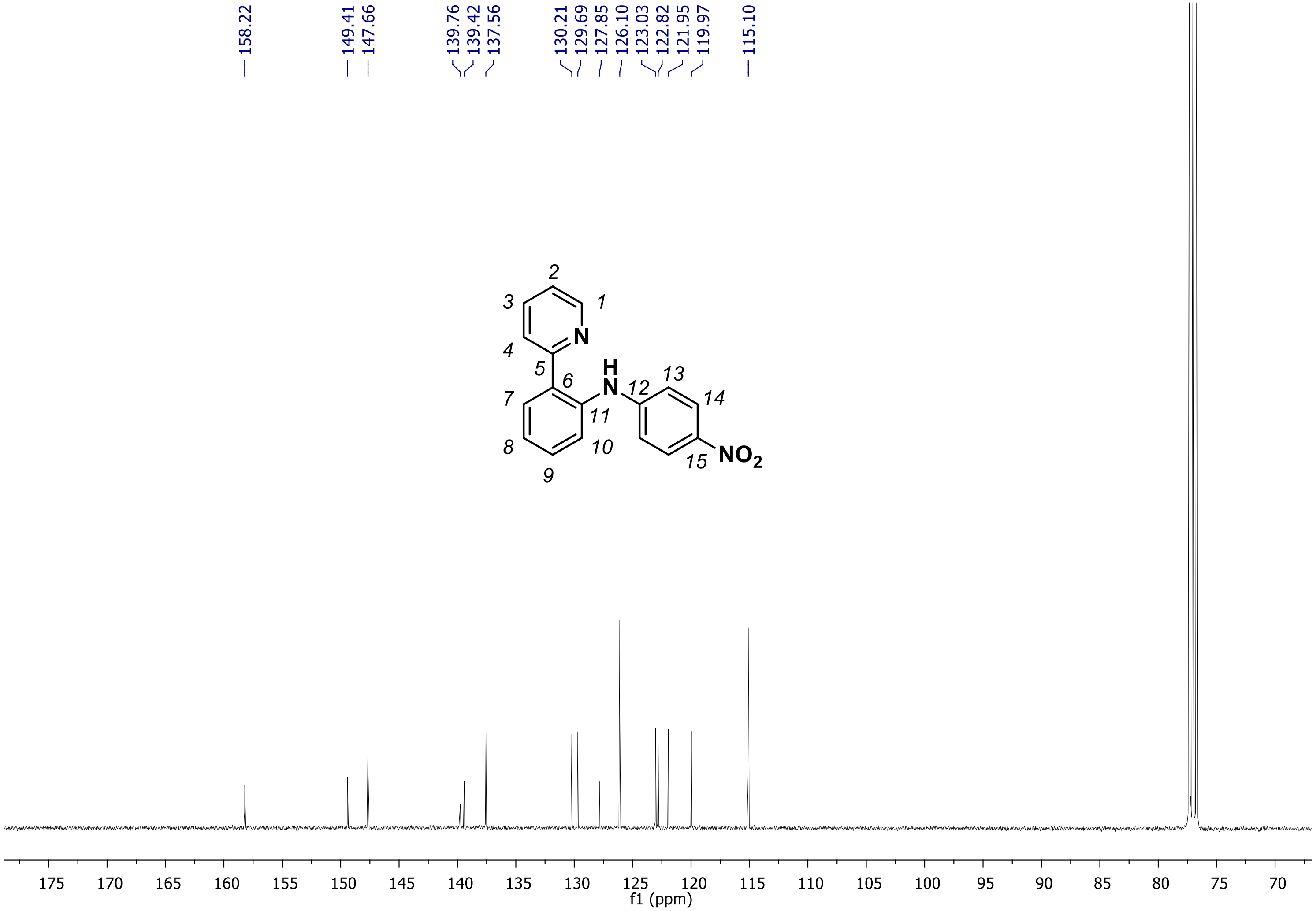
158.22
149.41
147.66
139.76
139.42
137.56
130.21
129.69
127.85
126.10
123.03
122.82
121.95
119.97
115.10
N
H
N
NO2
1
2
3
4
5
6
7
8
9
10
11
12
13
14
15
175
170
165
160
155
150
145
140
135
130
125
120
115
110
105
100
95
90
85
80
75
70
f1 (ppm)

c)

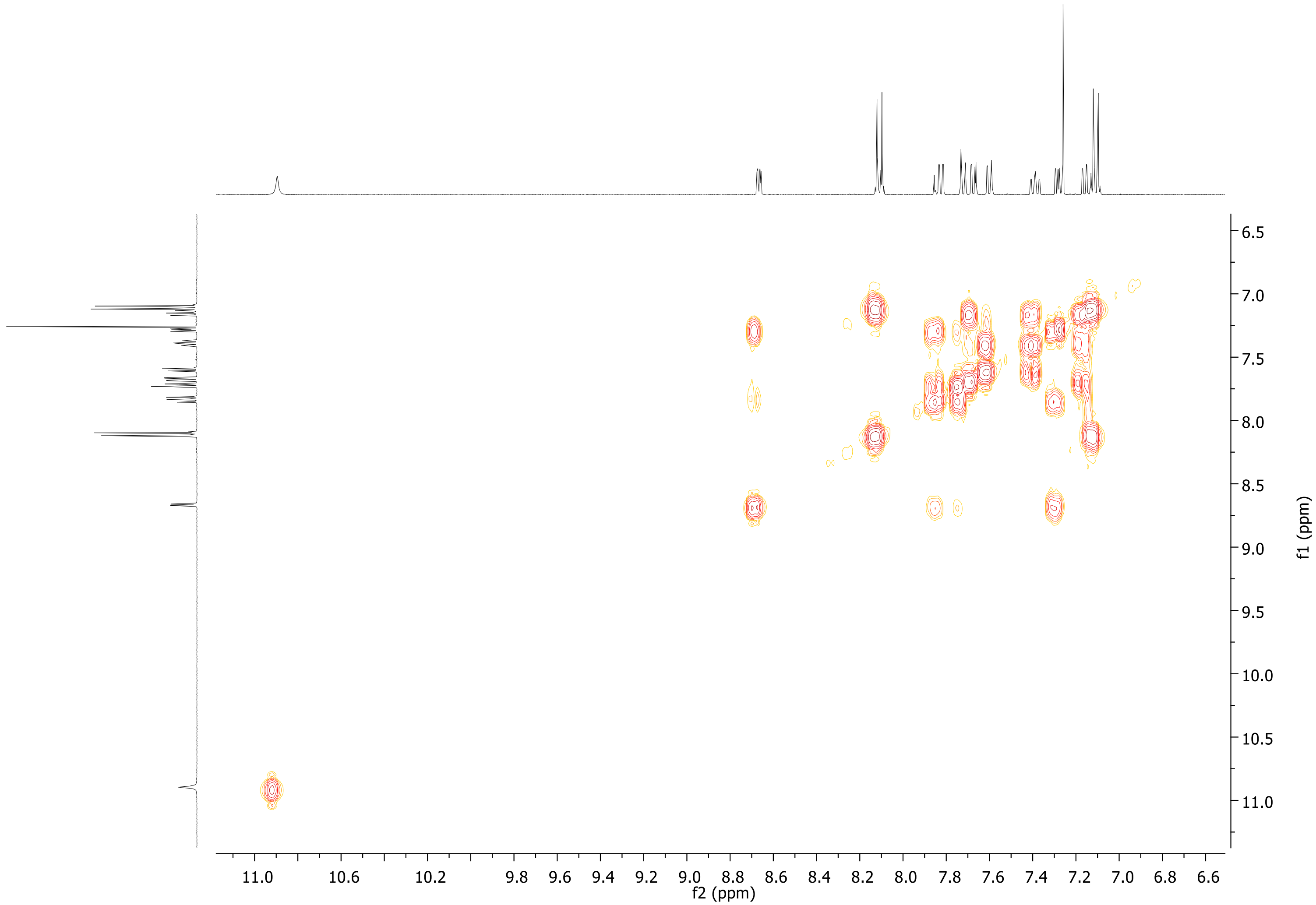

11.0
10.6
10.2
9.8
9.6
9.4
9.2
9.0
8.8
8.6
8.4
8.2
8.0
7.8
7.6
7.4
7.2
7.0
6.8
6.6
f2 (ppm)
6.5
7.0
7.5
8.0
8.5
9.0
9.5
10.0
10.5
11.0
f1 (ppm)

d)

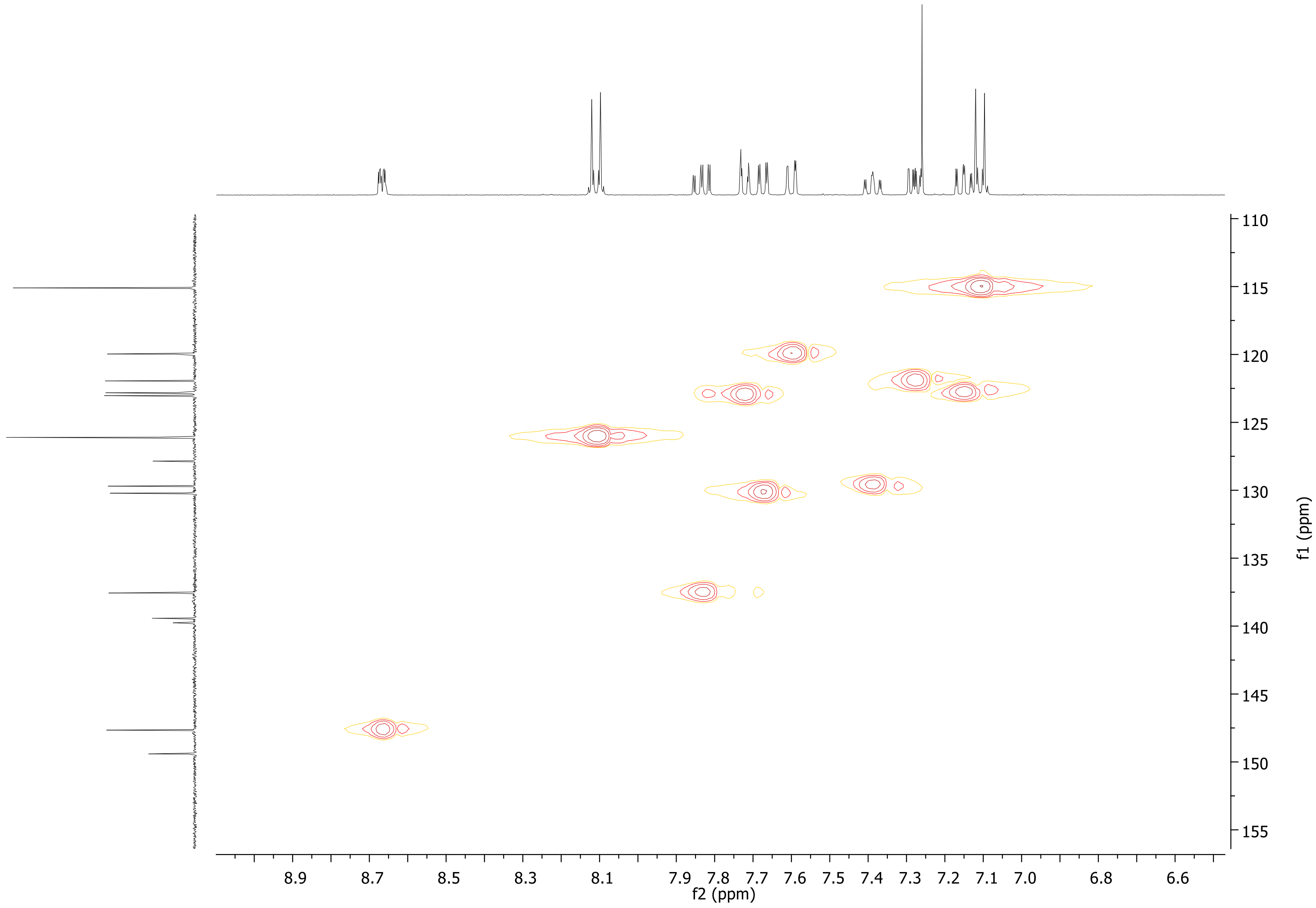
110
115
120
125
130
135
140
145
150
155
f1 (ppm)
8.9 8.7 8.5 8.3 8.1 7.9 7.8 7.7 7.6 7.5 7.4 7.3 7.2 7.1 7.0 6.8 6.6
f2 (ppm)

e)

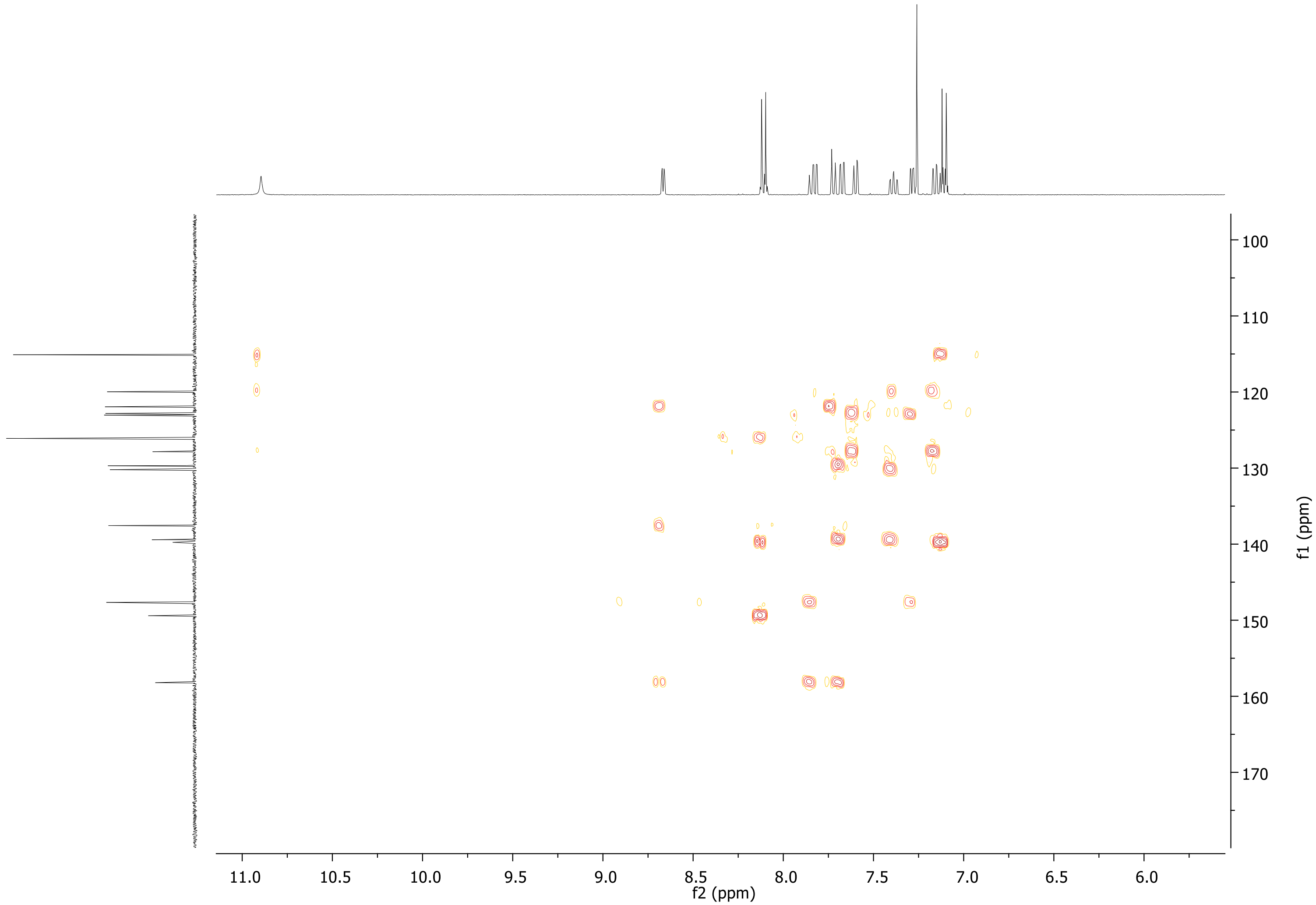

100
110
120
130
140
150
160
170
f1 (ppm)
11.0
10.5
10.0
9.5
9.0
8.5
8.0
7.5
7.0
6.5
6.0
f2 (ppm)

f)

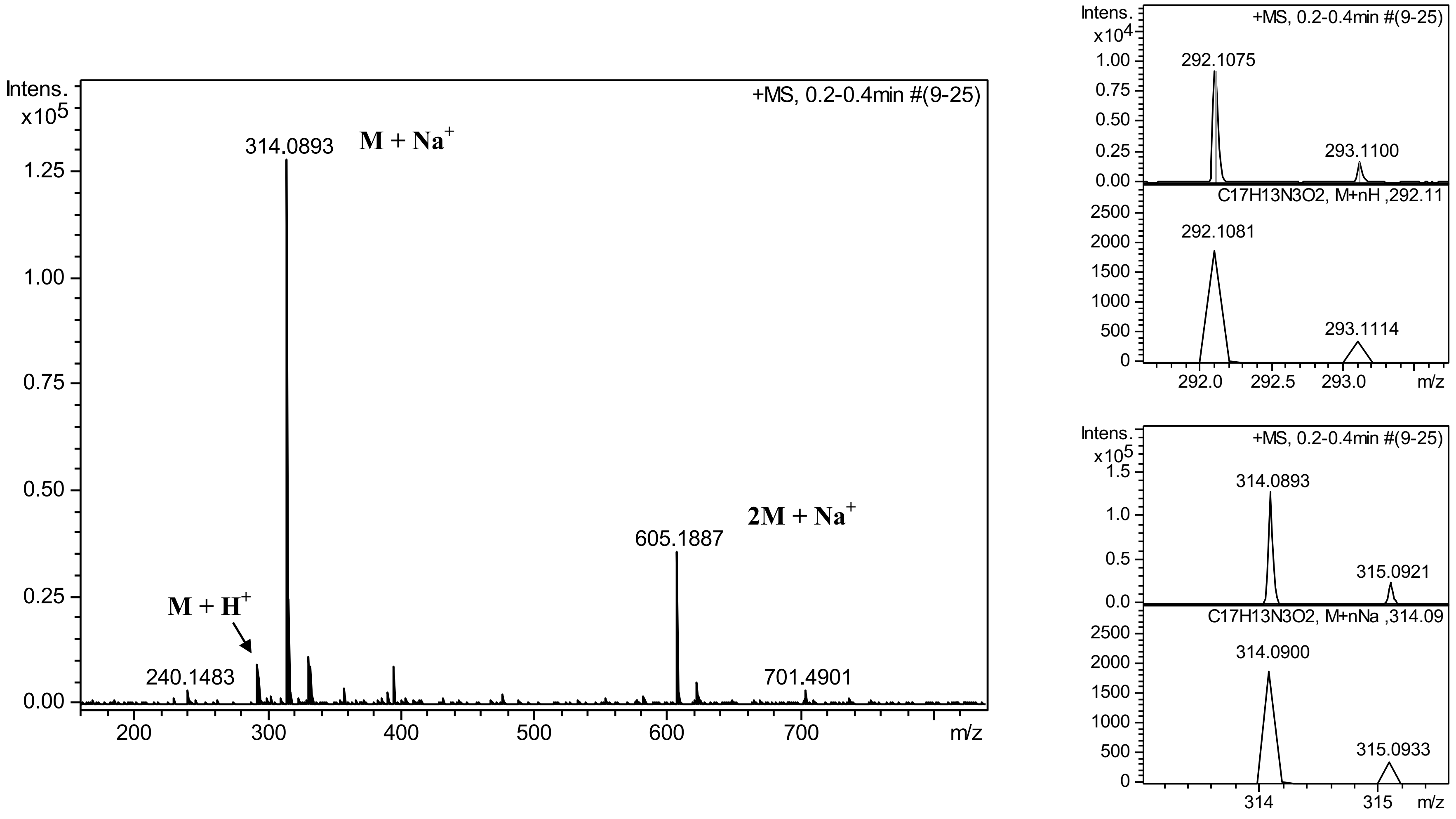
Intens. x10^5
+MS, 0.2-0.4min #(9-25)
314.0893
M + Na+
605.1887
2M + Na+
M + H+
240.1483
701.4901
m/z
Intens. x10^4
292.1075
293.1100
C17H13N3O2, M+nH ,292.11
292.1081
293.1114
314.0893
315.0921
C17H13N3O2, M+nNa ,314.09
314.0900
315.0933

Figure S14. a) ^{1}H-NMR spectrum of compound **2ah** in $CDCl_3$, 400 MHz, at 298 K; b) $^{13}C\{^{1}H\}$-NMR spectrum ($CDCl_3$, 100 MHz, 298 K); c) ^{1}H-^{1}H COSY spectrum ($CDCl_3$, 100 MHz, 298 K); d) ^{1}H-^{13}C HSQCed spectrum ($CDCl_3$, 400 MHz, 298 K); e) ^{1}H-^{13}C HMBC spectrum ($CDCl_3$, 400 MHz, 298 K); f) HRMS (ESI-MS) spectrum (*m/z*). Observed HRMS (left) with the theoretical isotope prediction (right).

a)

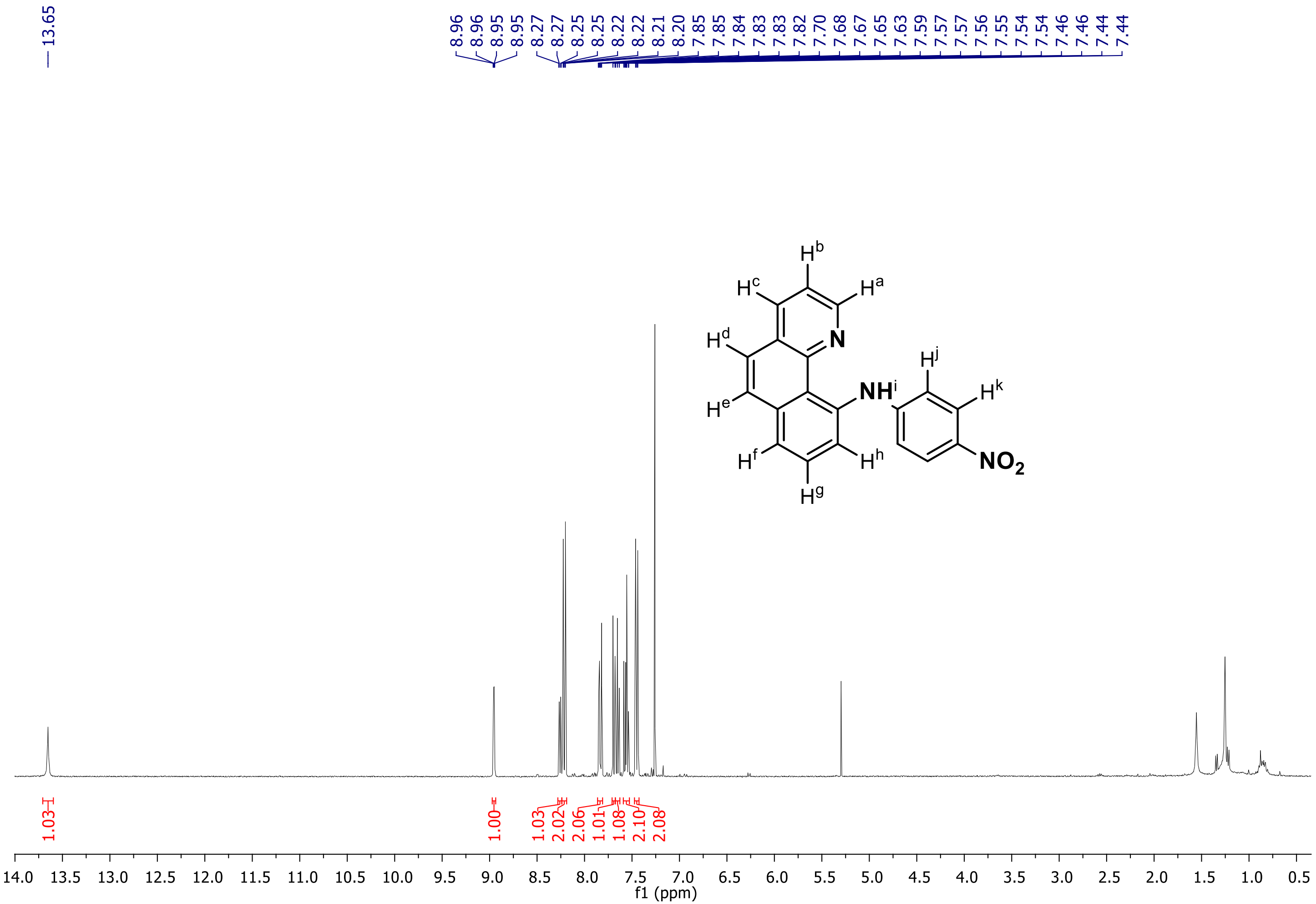

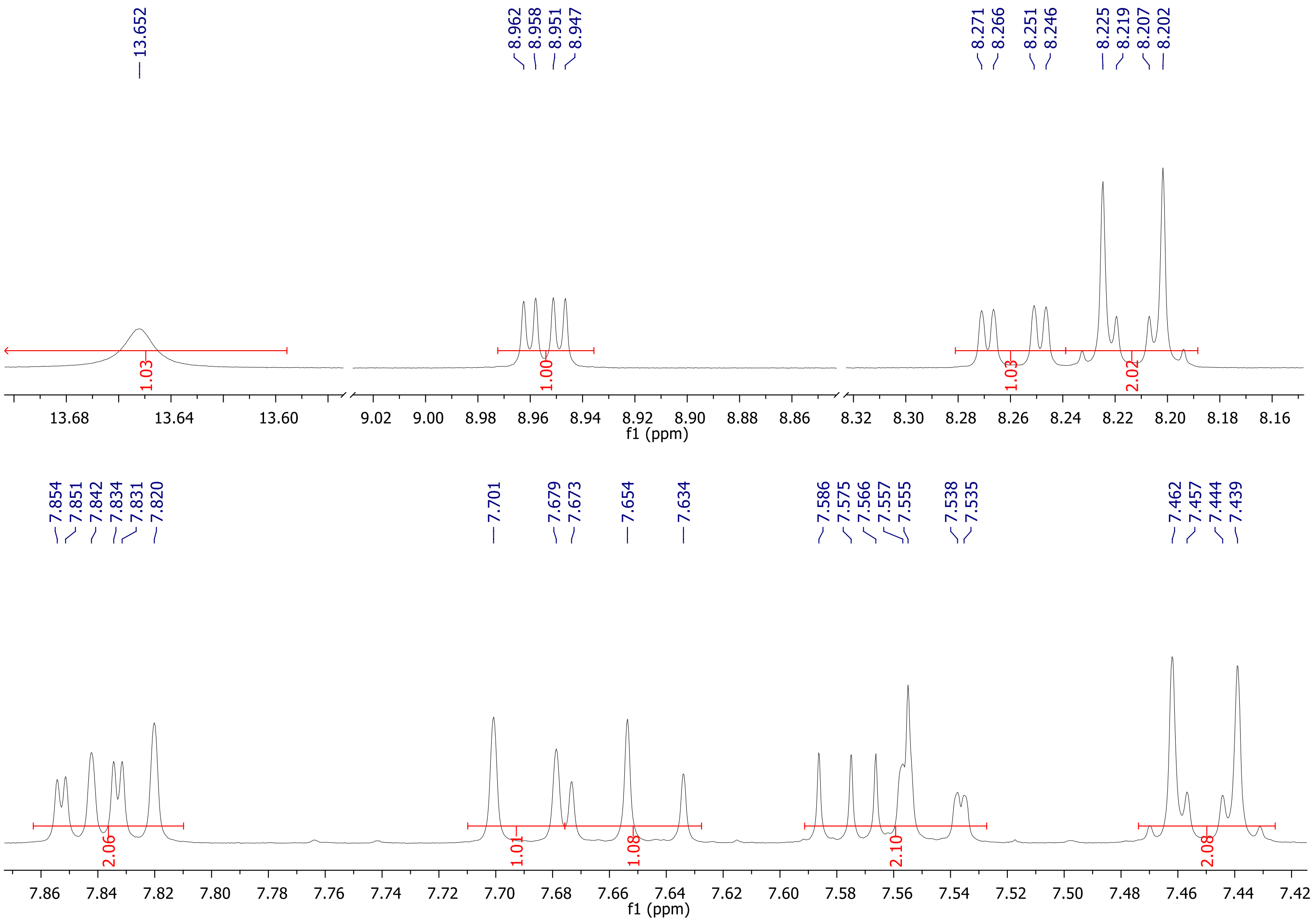

Selected aromatic region

b)

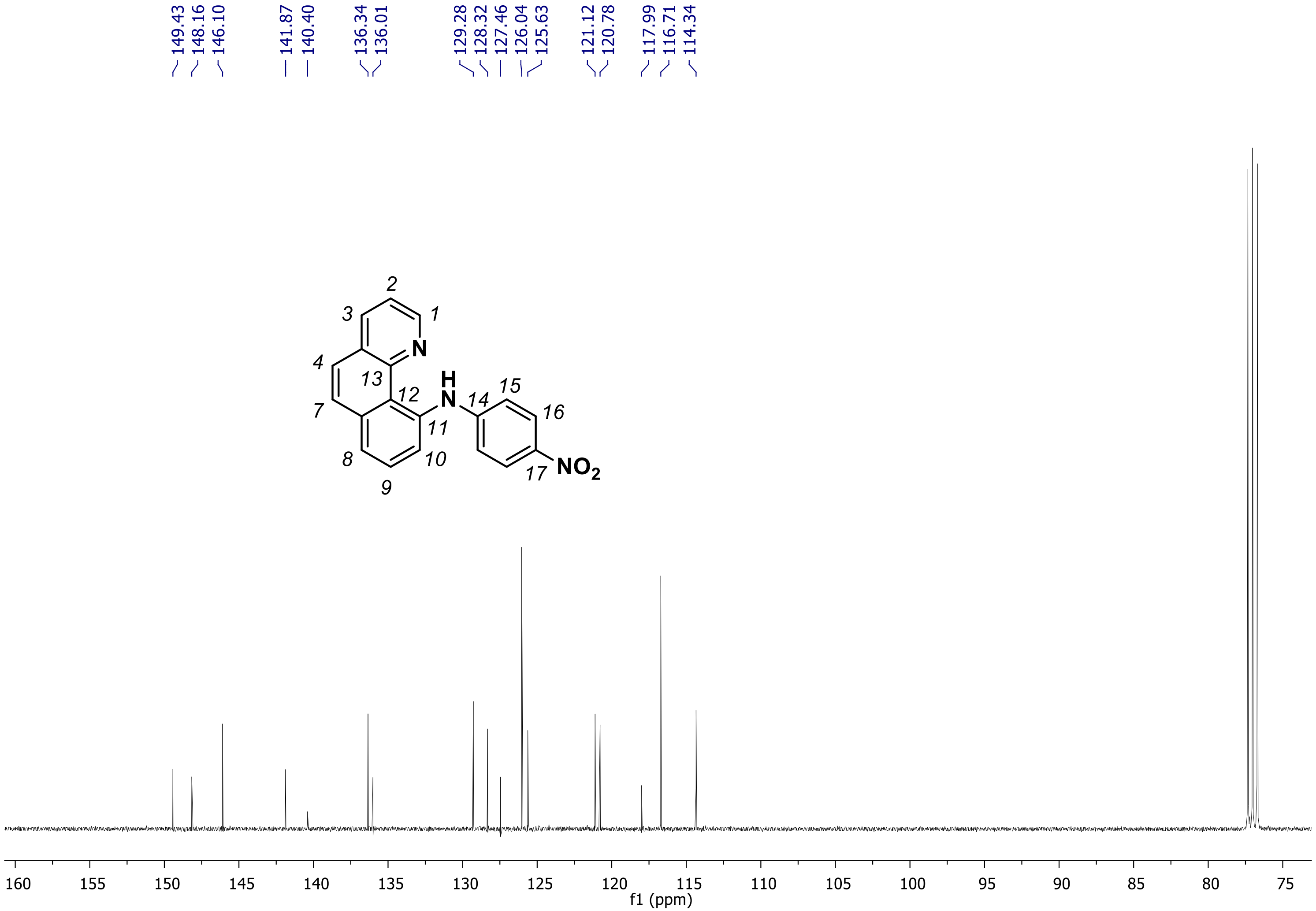
149.43
148.16
146.10
141.87
140.40
136.34
136.01
129.28
128.32
127.46
126.04
125.63
121.12
120.78
117.99
116.71
114.34
N
H
N
NO2
1
2
3
4
7
8
9
10
11
12
13
14
15
16
17
160
155
150
145
140
135
130
125
120
115
110
105
100
95
90
85
80
75
f1 (ppm)

c)

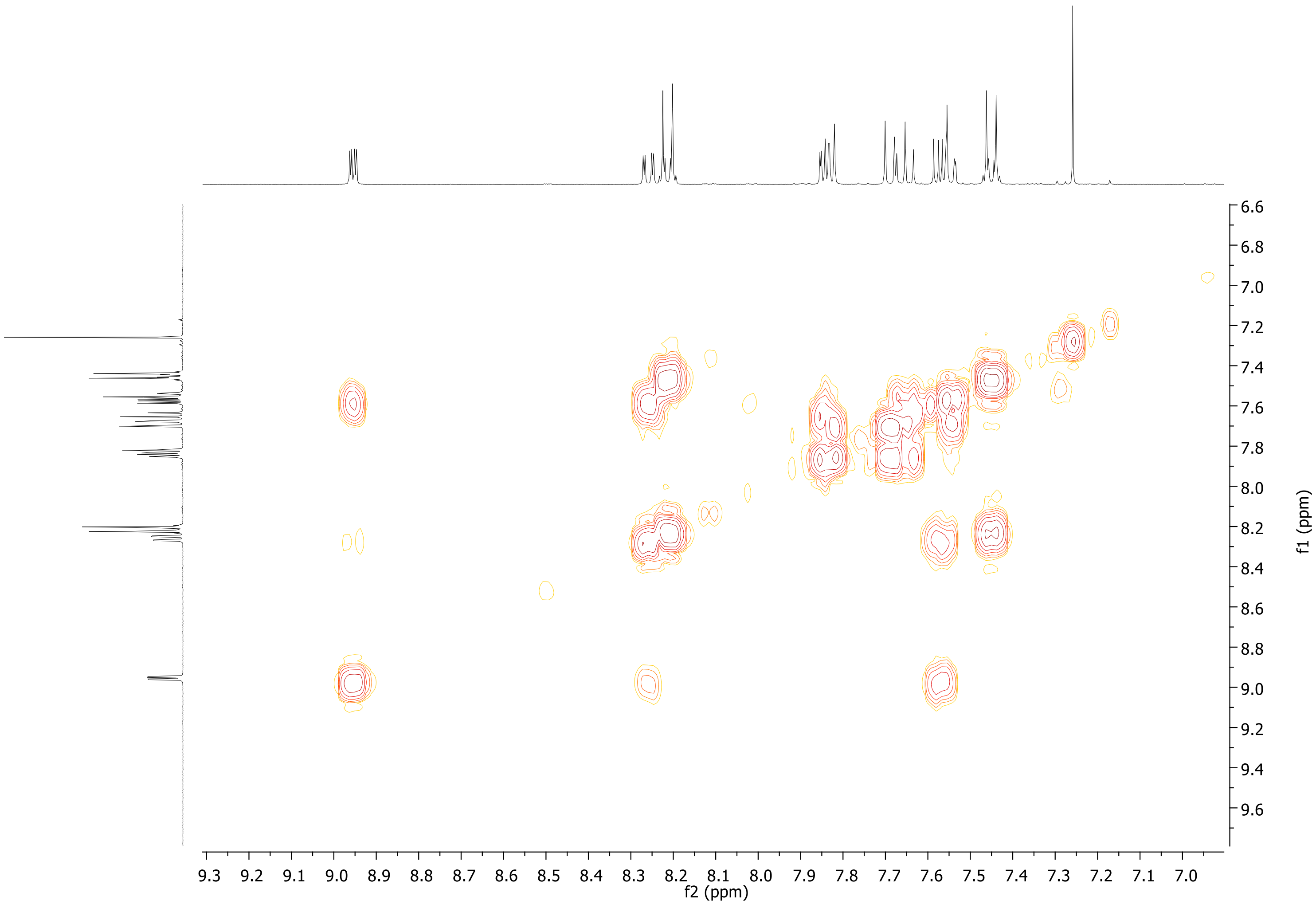

f2 (ppm)
f1 (ppm)
9.3
9.2
9.1
9.0
8.9
8.8
8.7
8.6
8.5
8.4
8.3
8.2
8.1
8.0
7.9
7.8
7.7
7.6
7.5
7.4
7.3
7.2
7.1
7.0
6.6
6.8
7.0
7.2
7.4
7.6
7.8
8.0
8.2
8.4
8.6
8.8
9.0
9.2
9.4
9.6

d)

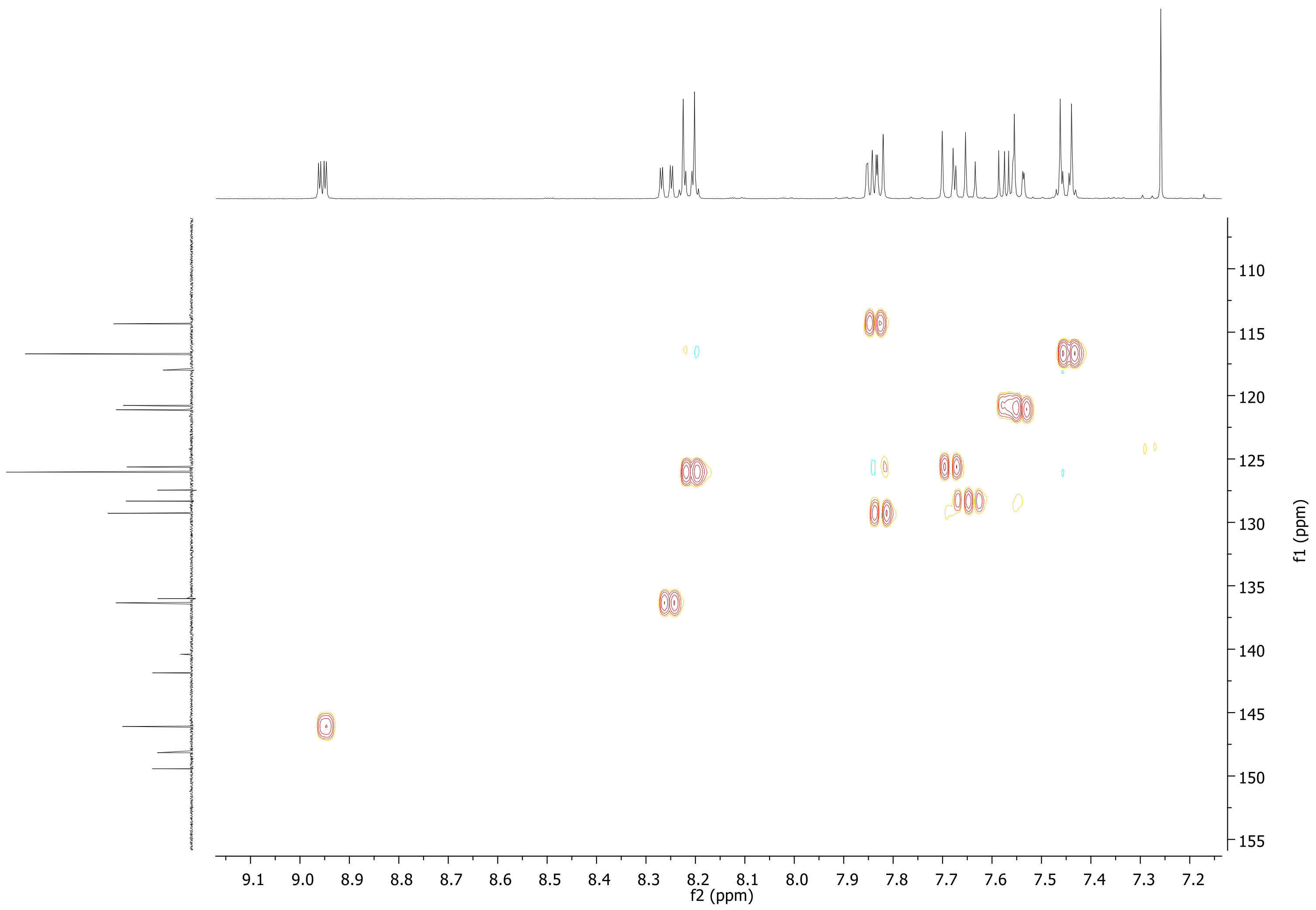

110
115
120
125
130
135
140
145
150
155
f1 (ppm)
9.1
9.0
8.9
8.8
8.7
8.6
8.5
8.4
8.3
8.2
8.1
8.0
7.9
7.8
7.7
7.6
7.5
7.4
7.3
7.2
f2 (ppm)

e)

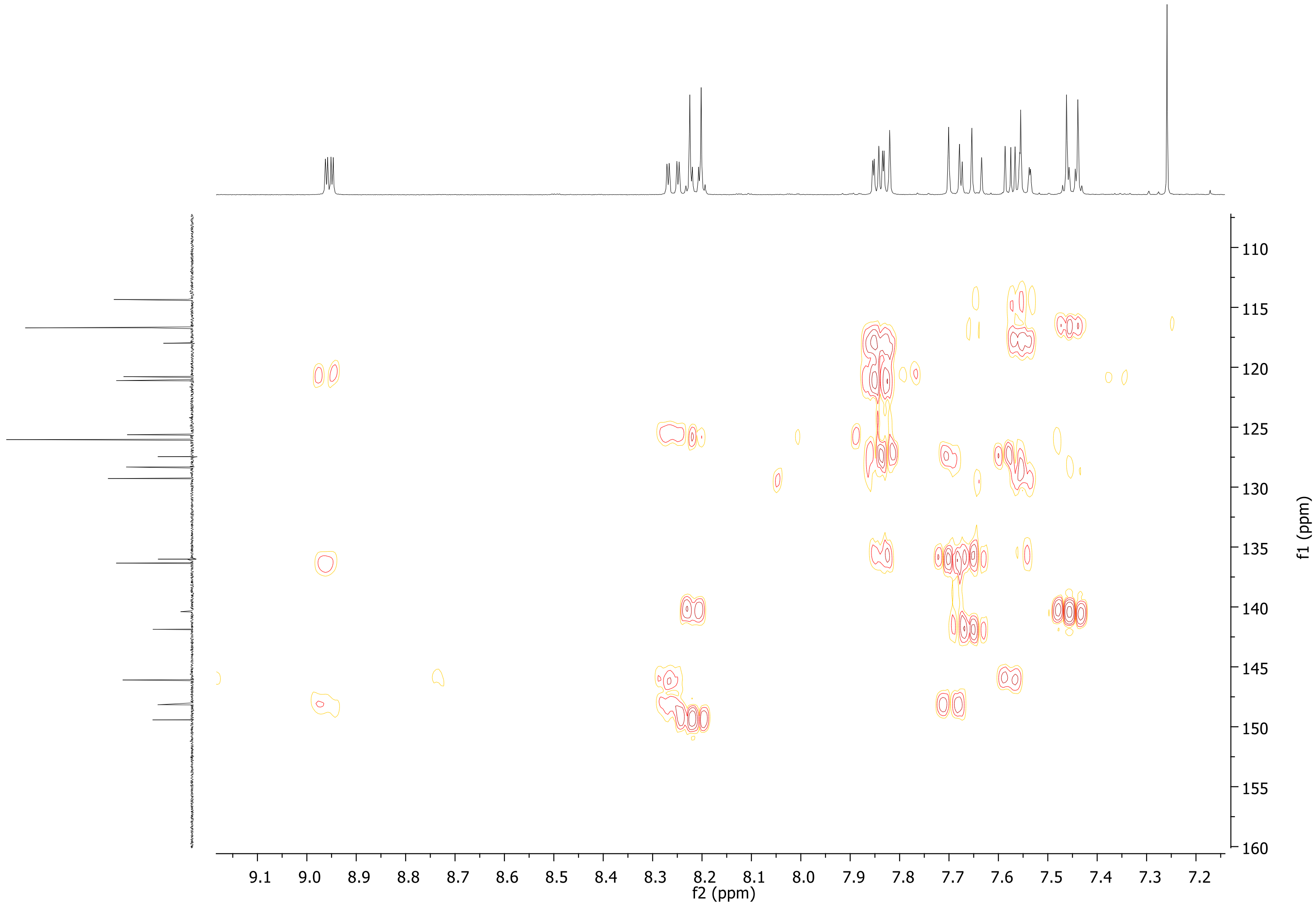

f)

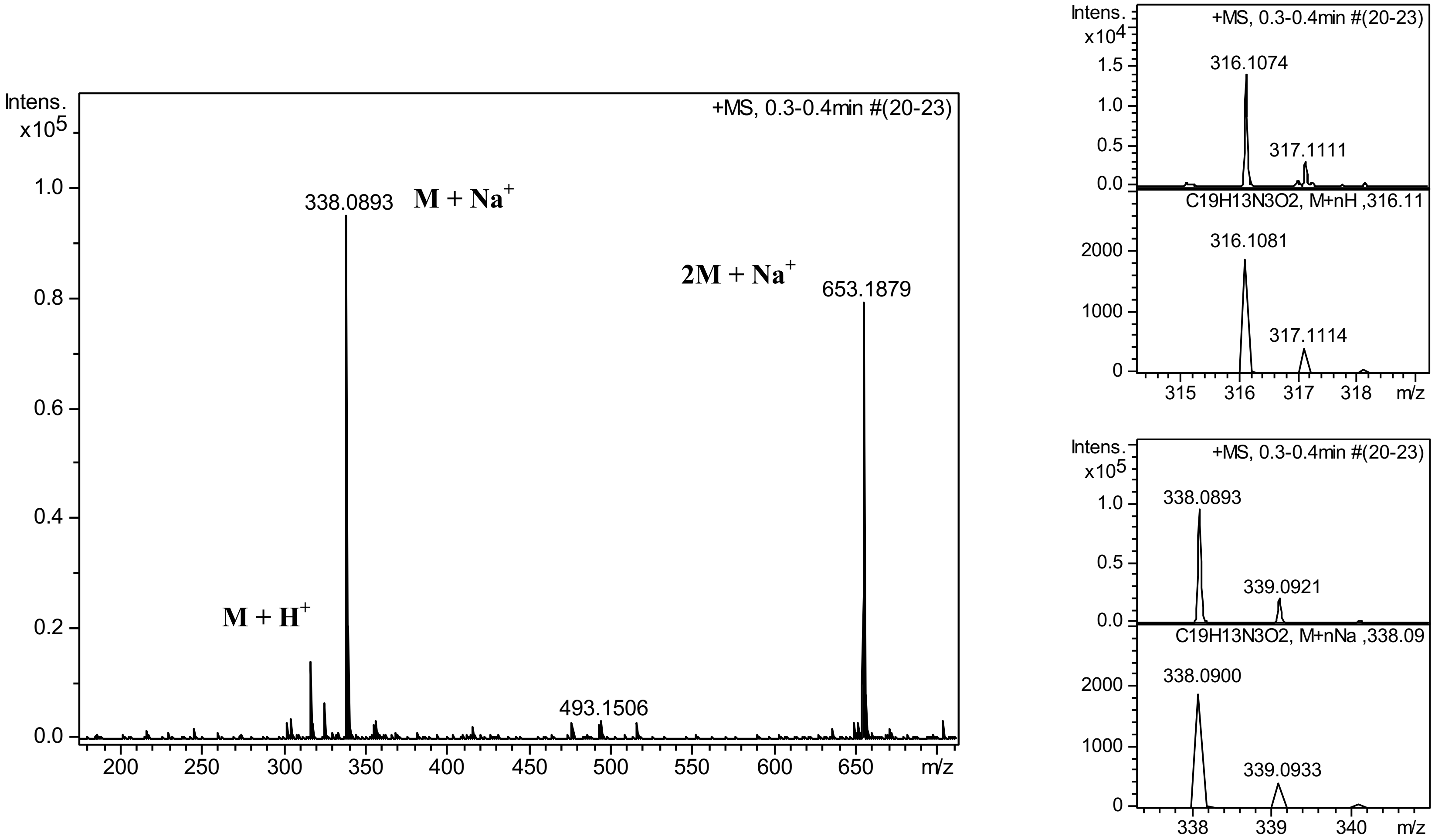
Intens.
x10^5
+MS, 0.3-0.4min #(20-23)
338.0893
M + Na+
2M + Na+
653.1879
M + H+
493.1506
1.0
0.8
0.6
0.4
0.2
0.0
200
250
300
350
400
450
500
550
600
650
m/z
Intens.
x10^4
+MS, 0.3-0.4min #(20-23)
316.1074
317.1111
C19H13N3O2, M+nH ,316.11
316.1081
317.1114
315
316
317
318
m/z
Intens.
x10^5
+MS, 0.3-0.4min #(20-23)
338.0893
339.0921
C19H13N3O2, M+nNa ,338.09
338.0900
339.0933
338
339
340
m/z

Figure S15. a) ^{1}H-NMR spectrum of compound **1ai** in $CDCl_3$, 400 MHz, at 298 K; b) $^{13}C\{^{1}H\}$-NMR spectrum ($CDCl_3$, 100 MHz, 298 K); c) ^{1}H-^{1}H COSY spectrum ($CDCl_3$, 100 MHz, 298 K); d) ^{1}H-^{13}C HSQCed spectrum ($CDCl_3$, 400 MHz, 298 K); e) ^{1}H-^{13}C HMBC spectrum ($CDCl_3$, 400 MHz, 298 K); f) HRMS (ESI-MS) spectrum (*m/z*). Observed HRMS (left) with the theoretical isotope prediction (right).

a)

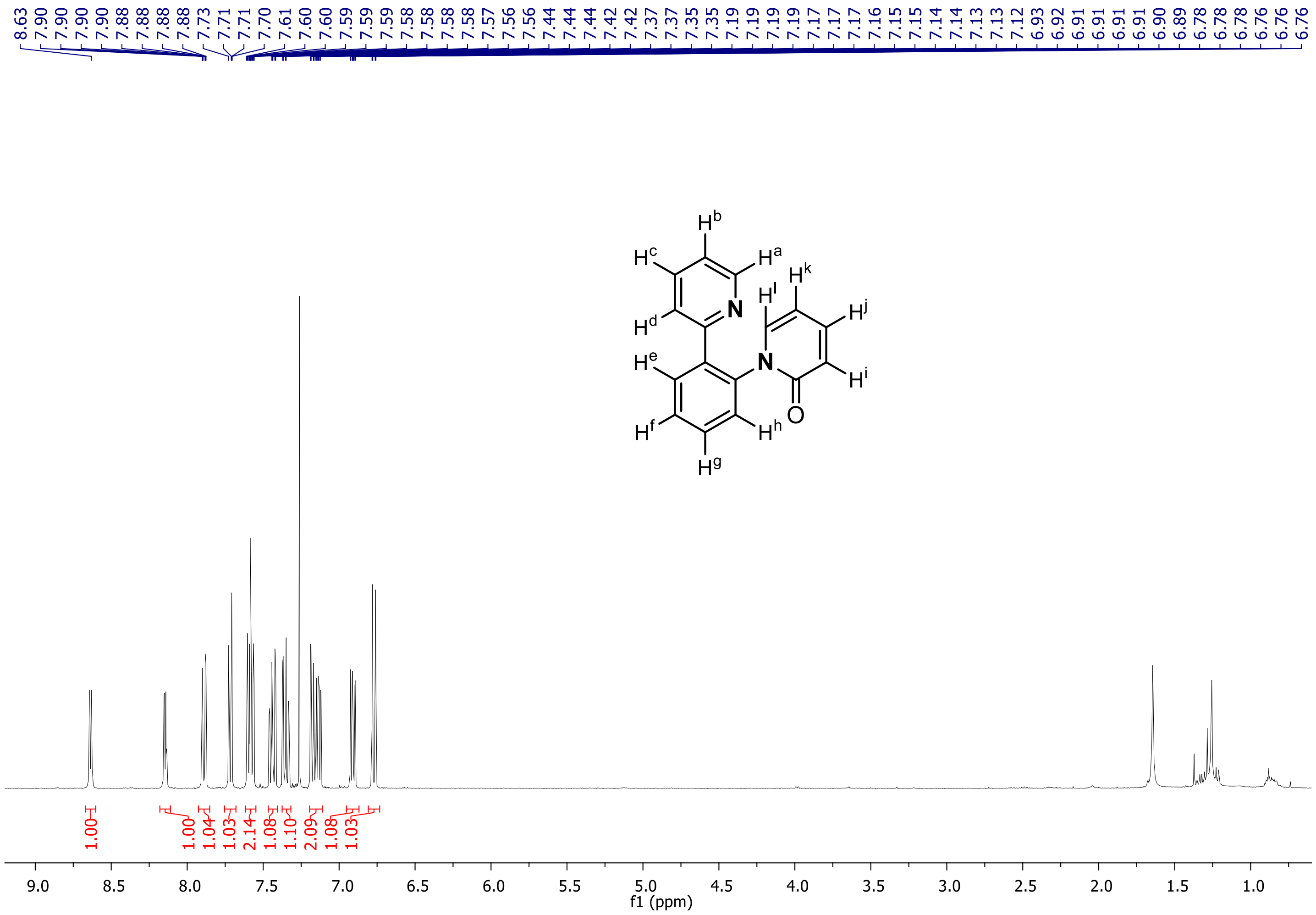

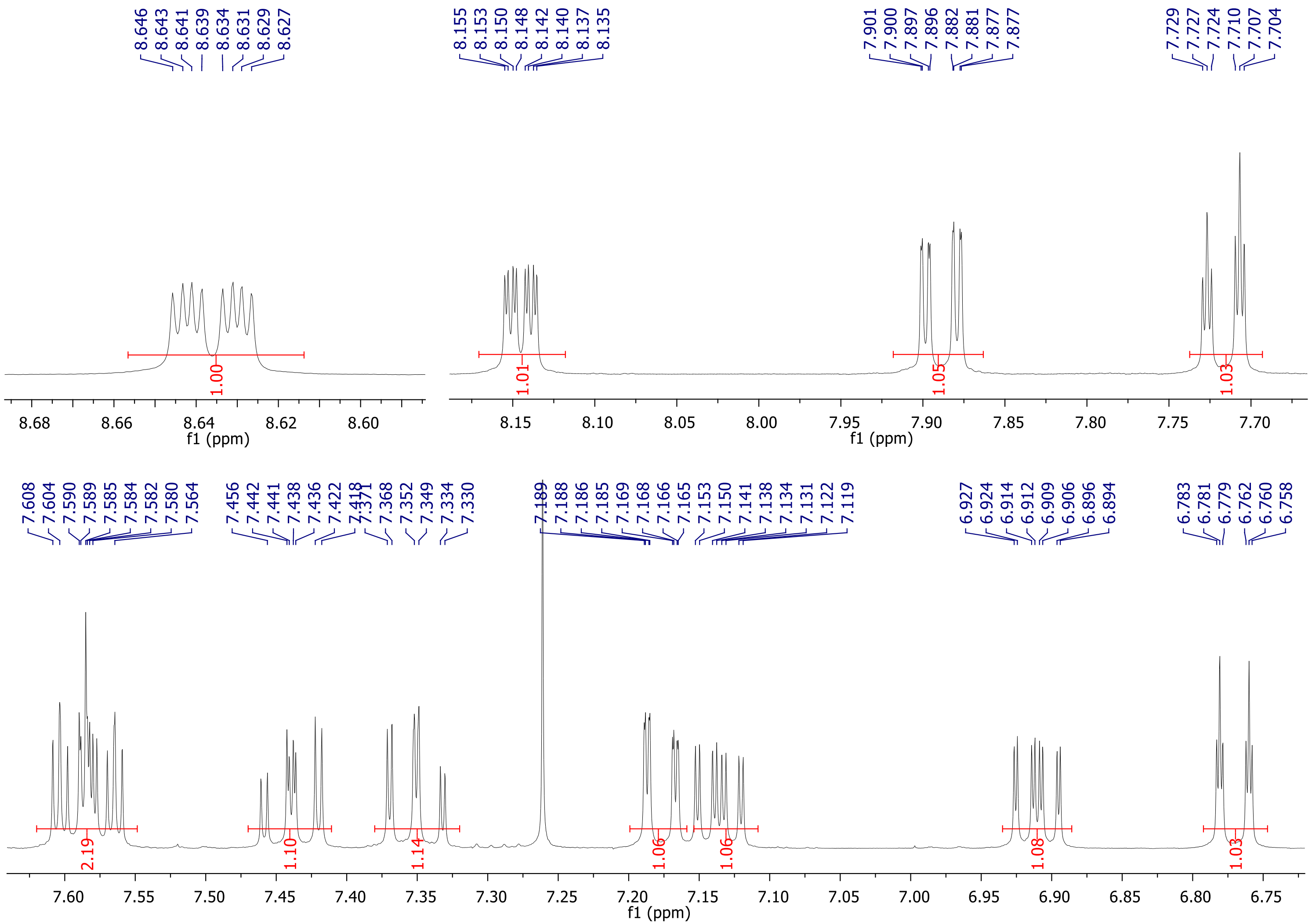

Selected aromatic region

b)

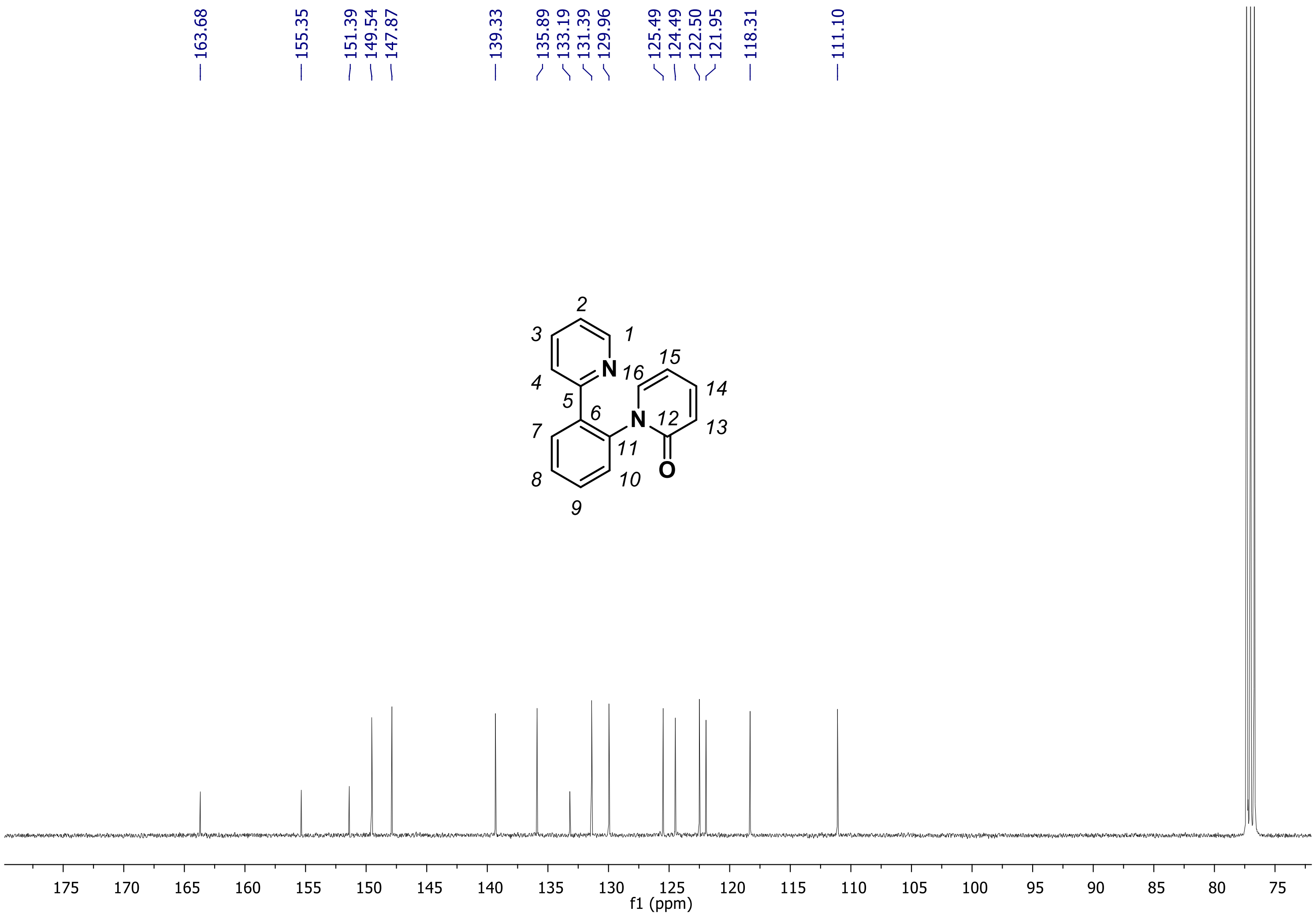
163.68
155.35
151.39
149.54
147.87
139.33
135.89
133.19
131.39
129.96
125.49
124.49
122.50
121.95
118.31
111.10
N
N
O
1
2
3
4
5
6
7
8
9
10
11
12
13
14
15
16
175
170
165
160
155
150
145
140
135
130
125
120
115
110
105
100
95
90
85
80
75
f1 (ppm)

c)

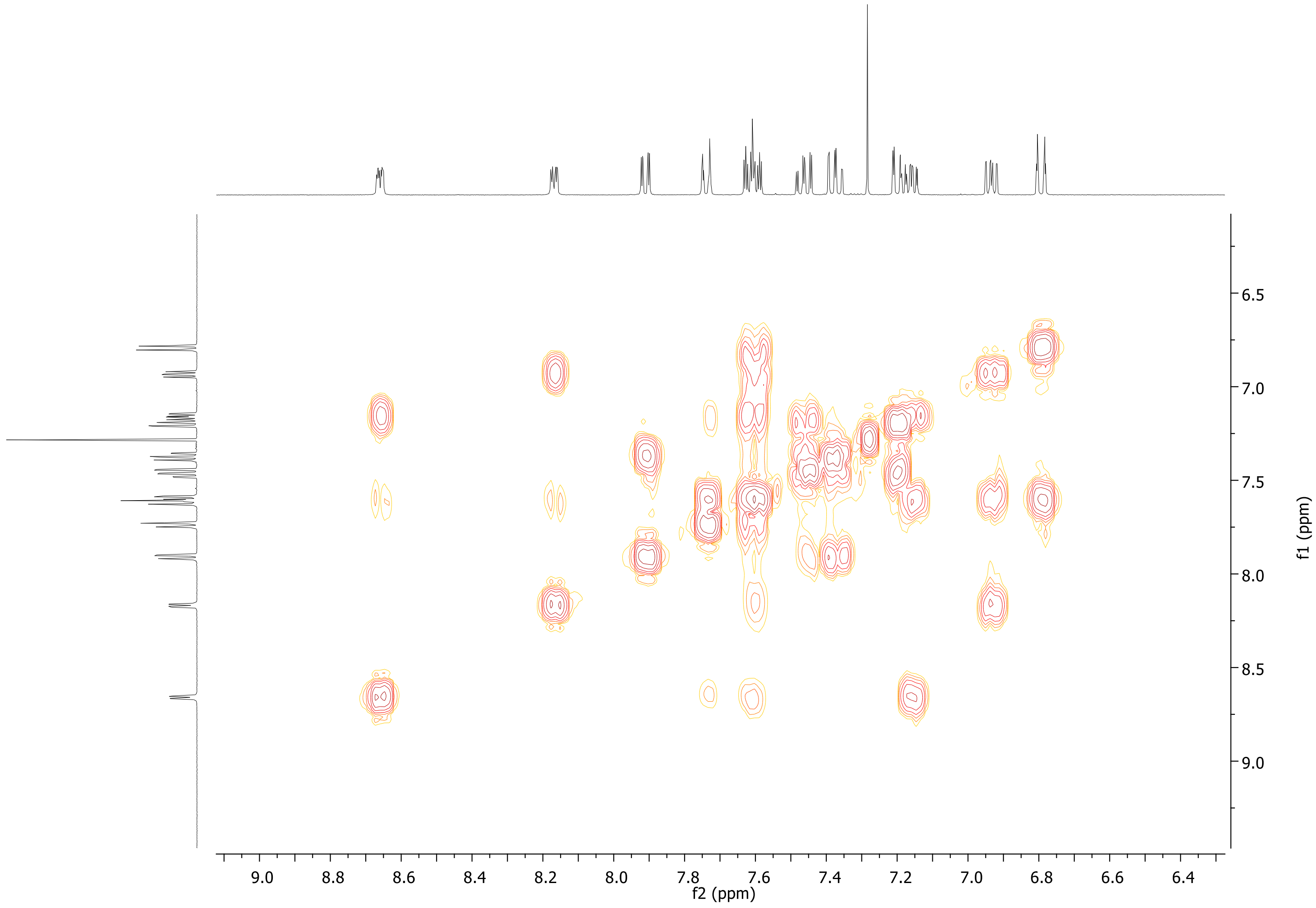
9.0
8.8
8.6
8.4
8.2
8.0
7.8
7.6
7.4
7.2
7.0
6.8
6.6
6.4
f2 (ppm)
6.5
7.0
7.5
8.0
8.5
9.0
f1 (ppm)

d)

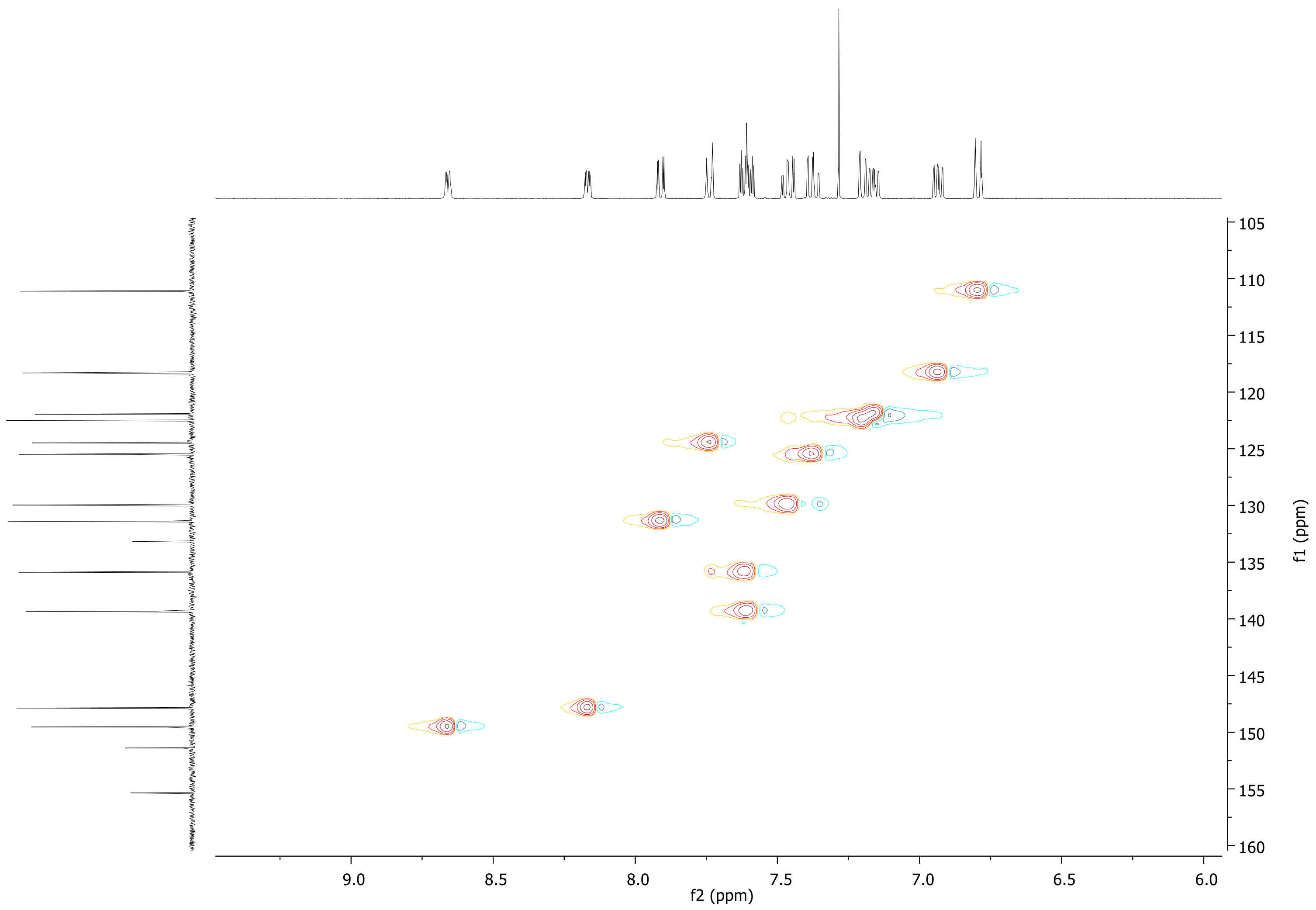

105
110
115
120
125
130
135
140
145
150
155
160
f1 (ppm)
9.0
8.5
8.0
7.5
7.0
6.5
6.0
f2 (ppm)

e)

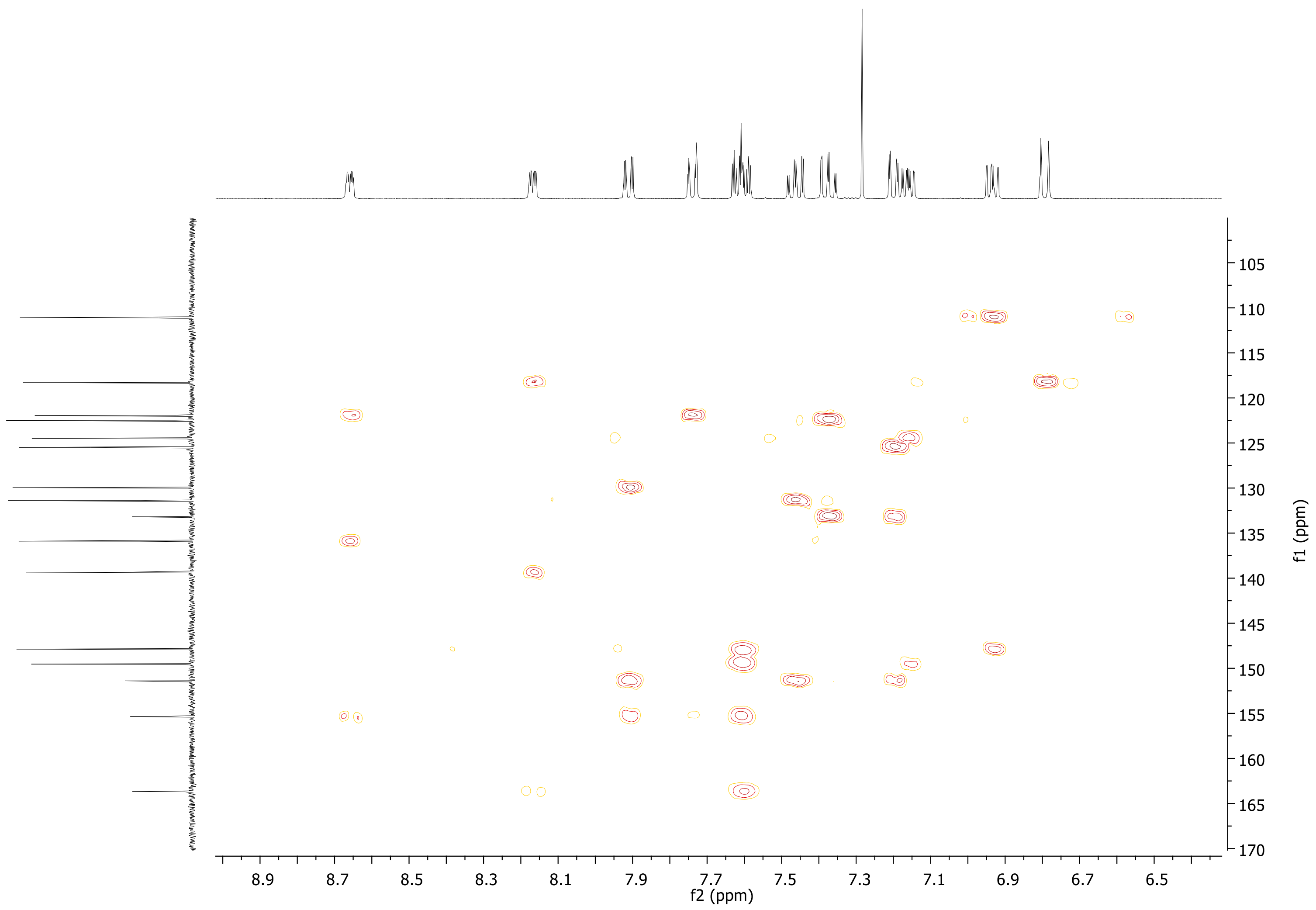
8.9
8.7
8.5
8.3
8.1
7.9
7.7
7.5
7.3
7.1
6.9
6.7
6.5
f2 (ppm)
105
110
115
120
125
130
135
140
145
150
155
160
165
170
f1 (ppm)

f)

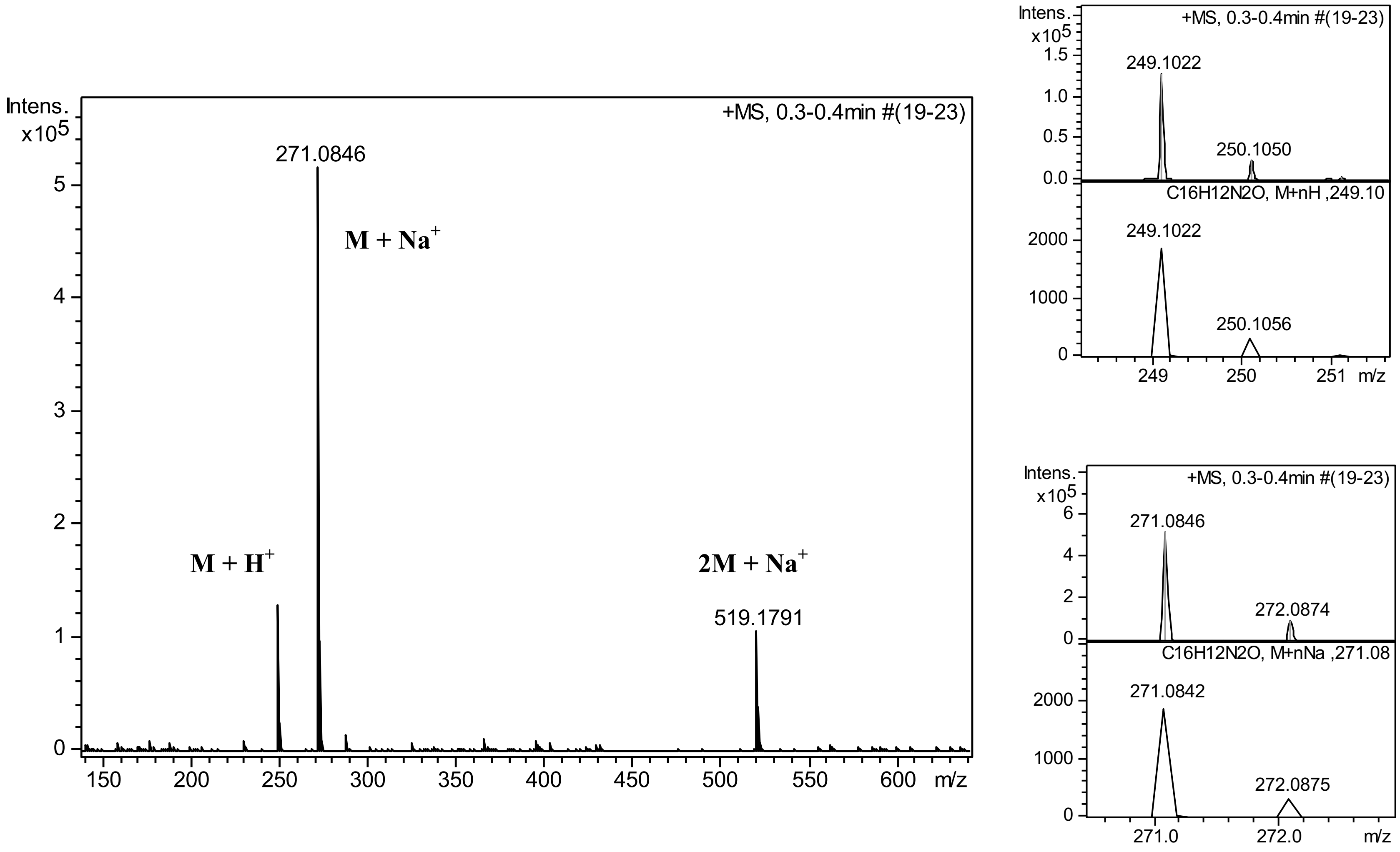
Intens.
x10^5
+MS, 0.3-0.4min #(19-23)
271.0846
M + Na+
M + H+
2M + Na+
519.1791
m/z
+MS, 0.3-0.4min #(19-23)
249.1022
250.1050
C16H12N2O, M+nH ,249.10
249.1022
250.1056
+MS, 0.3-0.4min #(19-23)
271.0846
272.0874
C16H12N2O, M+nNa ,271.08
271.0842
272.0875

Figure S16. a) ^{1}H-NMR spectrum of compound **1aj** in $CDCl_3$, 400 MHz, at 298 K; b) $^{13}C\{^1H\}$-NMR spectrum ($CDCl_3$, 100 MHz, 298 K); c) ^{1}H-^{1}H COSY spectrum ($CDCl_3$, 100 MHz, 298 K); d) ^{1}H-^{13}C HSQCed spectrum ($CDCl_3$, 400 MHz, 298 K); e) ^{1}H-^{13}C HMBC spectrum ($CDCl_3$, 400 MHz, 298 K); f) HRMS (ESI-MS) spectrum (*m/z*). Observed HRMS (left) with the theoretical isotope prediction (right).

a)

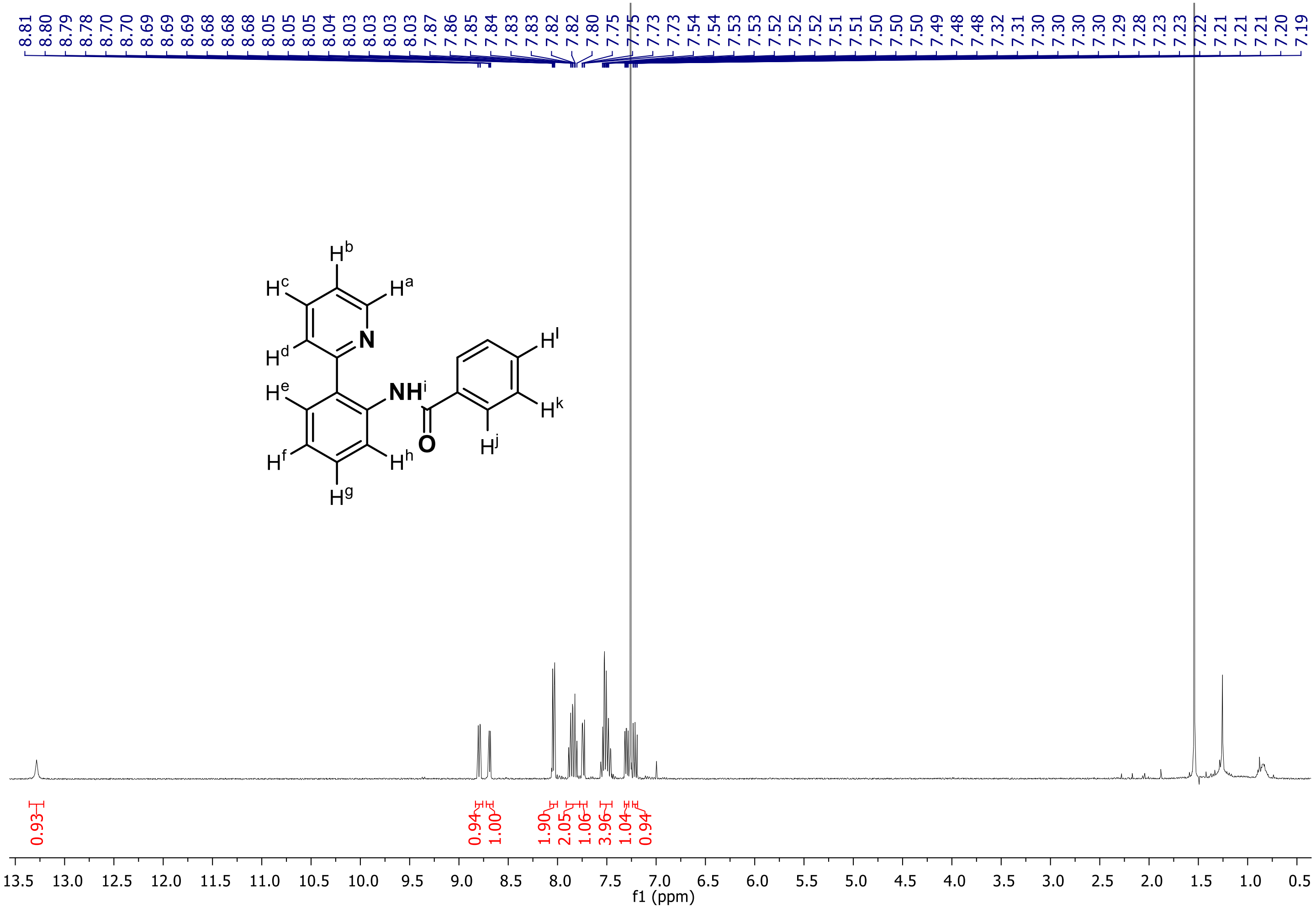

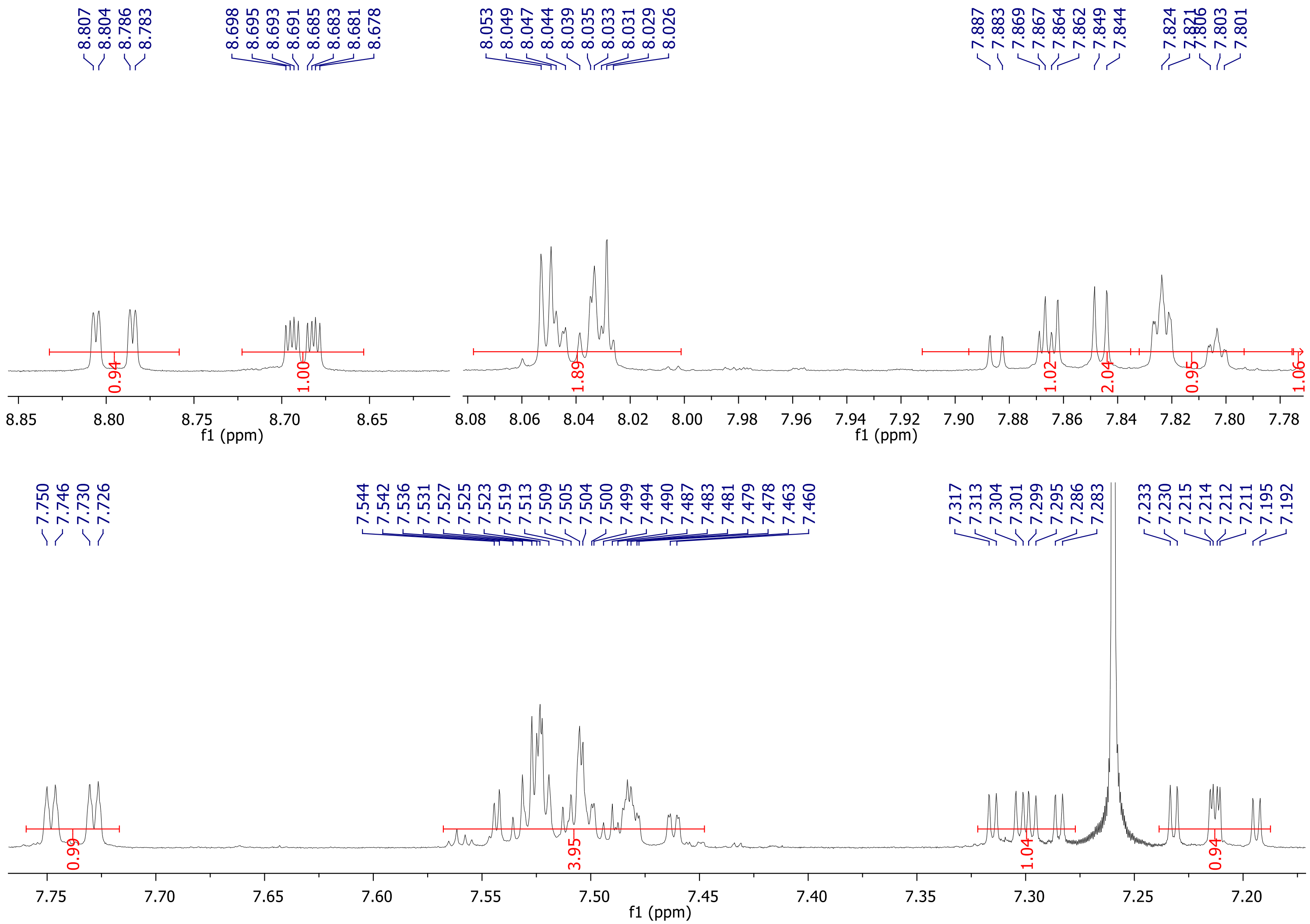

Selected aromatic region

b)

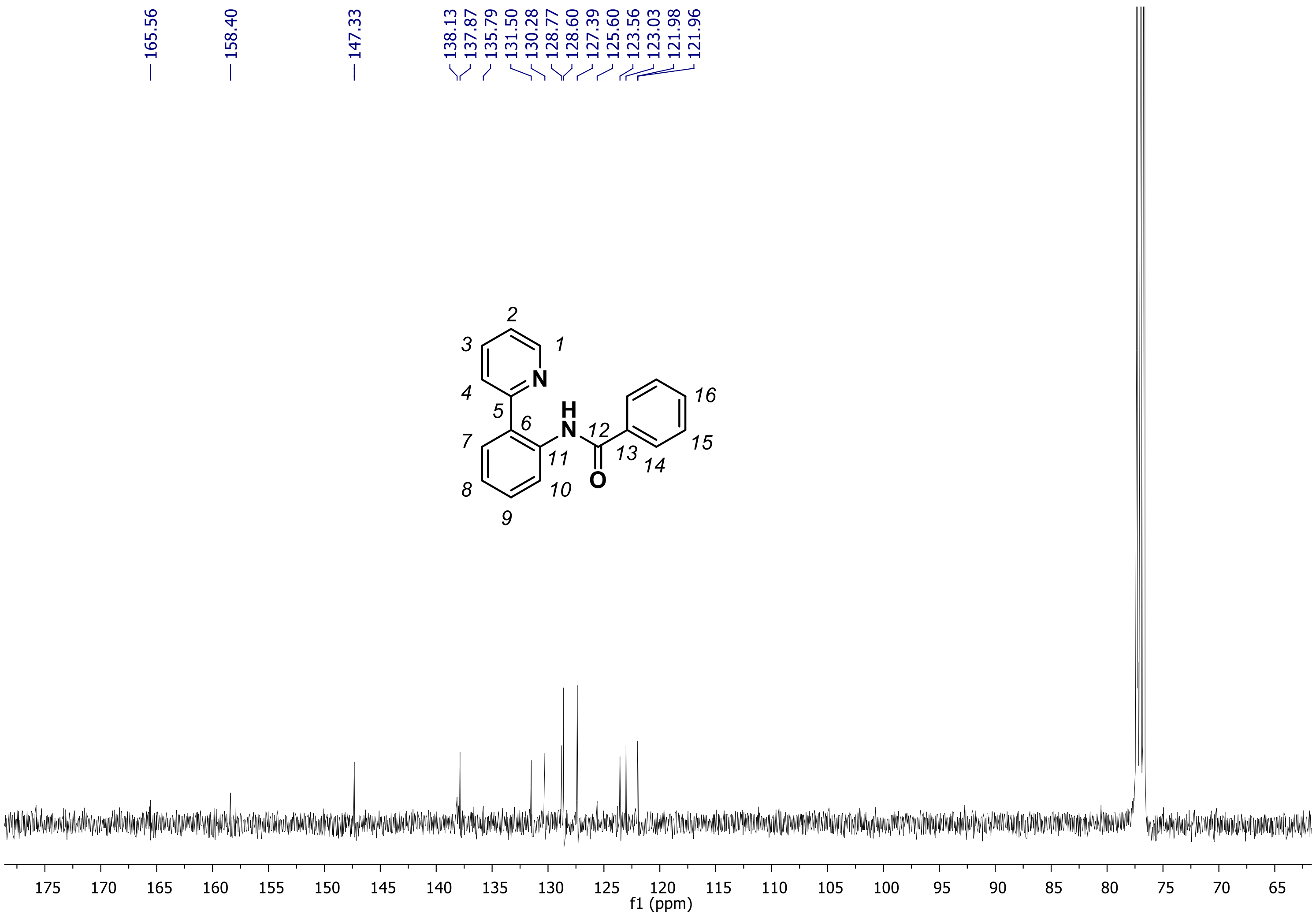

165.56
158.40
147.33
138.13
137.87
135.79
131.50
130.28
128.77
128.60
127.39
125.60
123.56
123.03
121.98
121.96
175 170 165 160 155 150 145 140 135 130 125 120 115 110 105 100 95 90 85 80 75 70 65
f1 (ppm)
1 2 3 4 5 6 7 8 9 10 11 12 13 14 15 16
N
H
N
O

c)

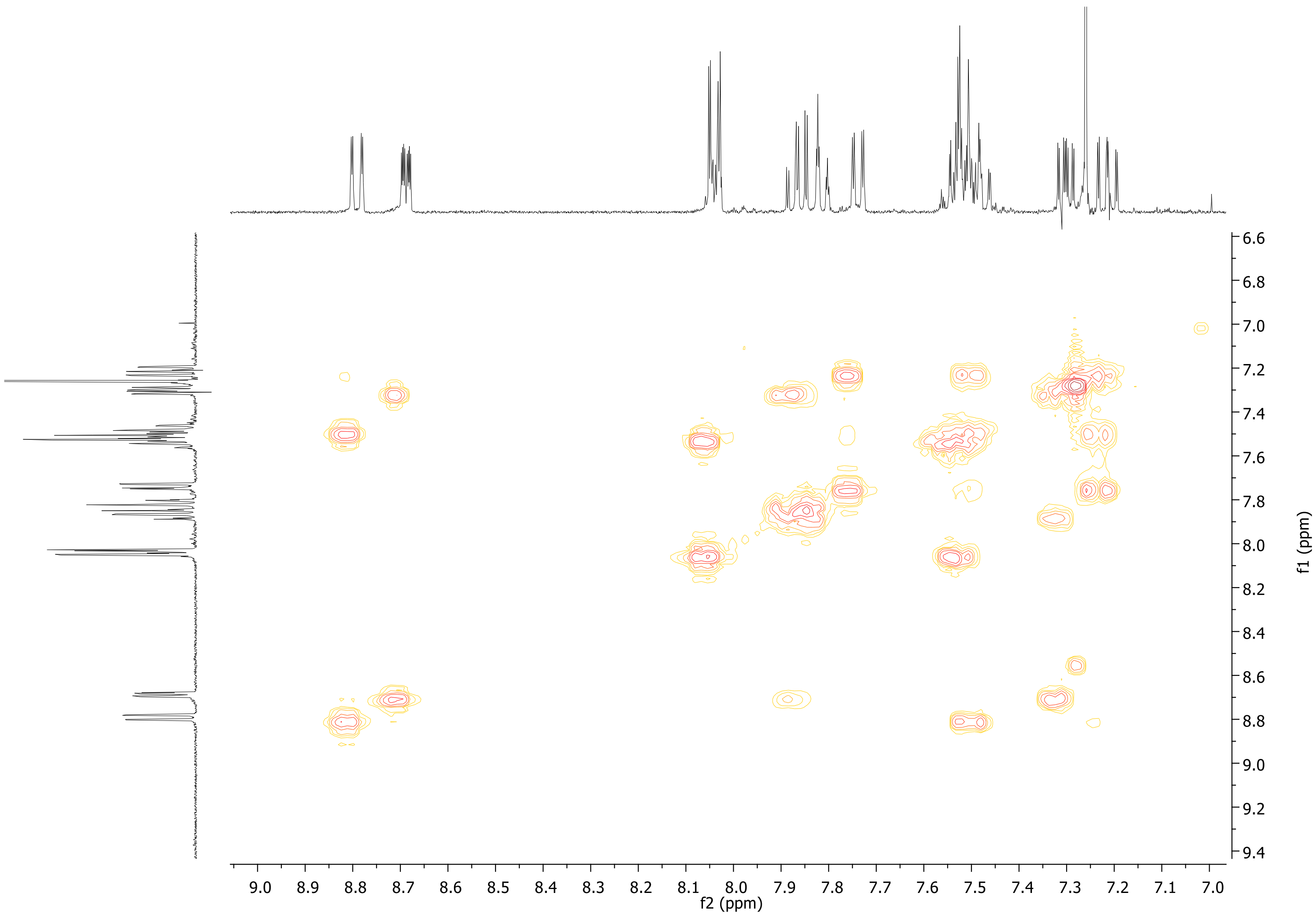

9.0
8.9
8.8
8.7
8.6
8.5
8.4
8.3
8.2
8.1
8.0
7.9
7.8
7.7
7.6
7.5
7.4
7.3
7.2
7.1
7.0
f2 (ppm)
6.6
6.8
7.0
7.2
7.4
7.6
7.8
8.0
8.2
8.4
8.6
8.8
9.0
9.2
9.4
f1 (ppm)

d)

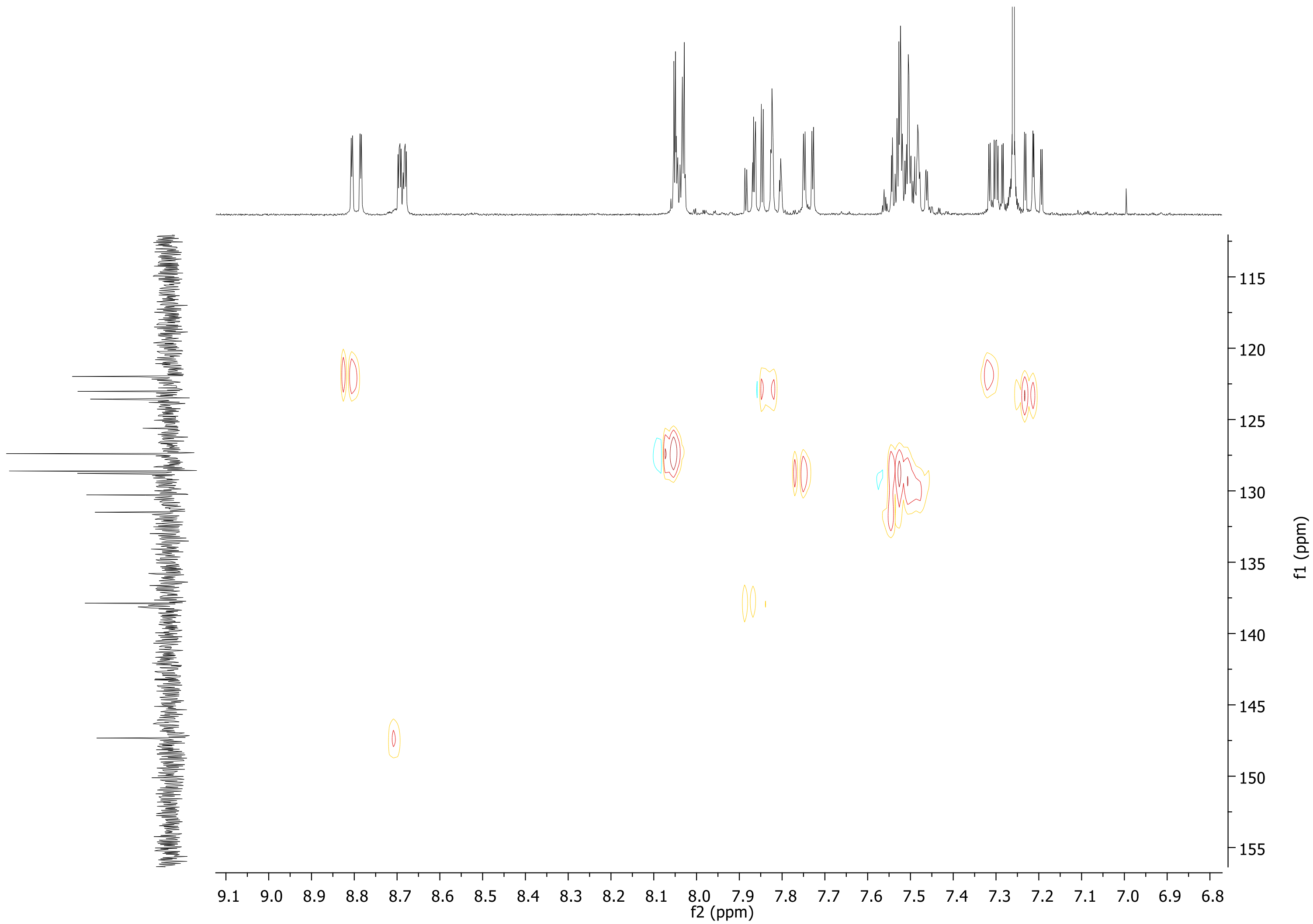

115
120
125
130
135
140
145
150
155
f1 (ppm)
9.1
9.0
8.9
8.8
8.7
8.6
8.5
8.4
8.3
8.2
8.1
8.0
7.9
7.8
7.7
7.6
7.5
7.4
7.3
7.2
7.1
7.0
6.9
6.8
f2 (ppm)

e)

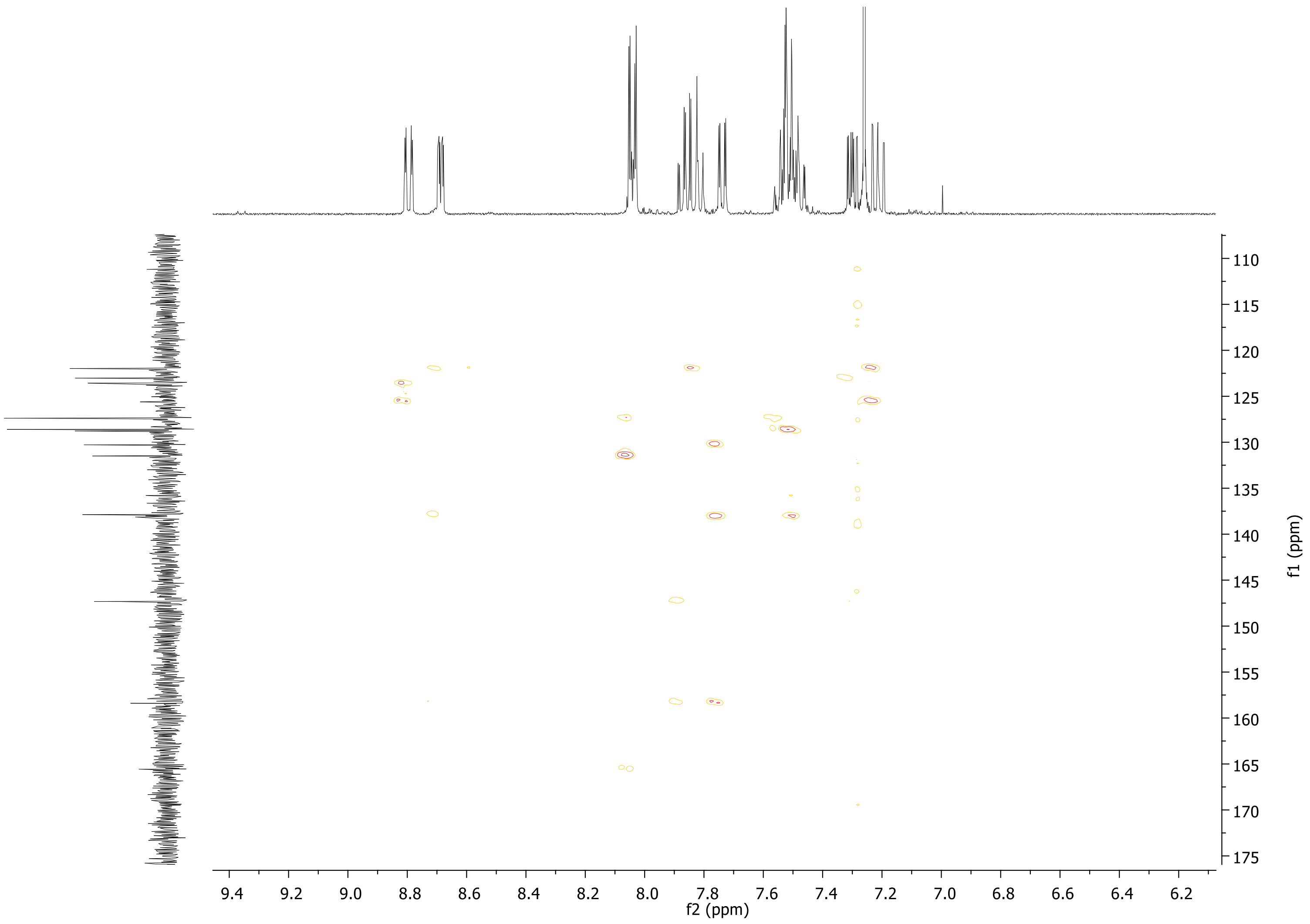

f)

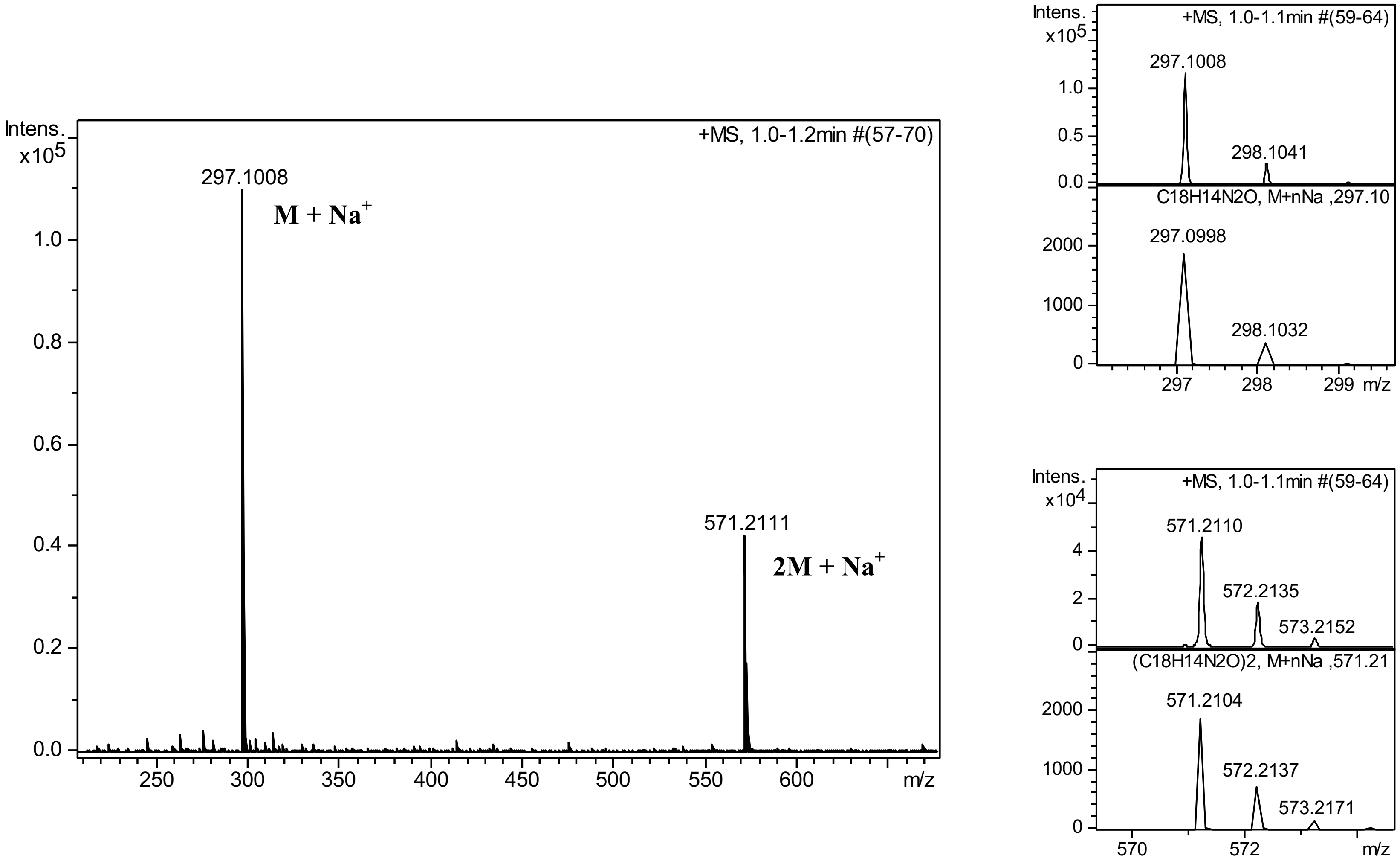
Intens.
x10^5
+MS, 1.0-1.2min #(57-70)
297.1008
M + Na+
571.2111
2M + Na+
1.0
0.8
0.6
0.4
0.2
0.0
250
300
350
400
450
500
550
600
m/z
+MS, 1.0-1.1min #(59-64)
297.1008
298.1041
C18H14N2O, M+nNa ,297.10
297.0998
298.1032
297
298
299
+MS, 1.0-1.1min #(59-64)
x10^4
571.2110
572.2135
573.2152
(C18H14N2O)2, M+nNa ,571.21
571.2104
572.2137
573.2171
570
572
2000
1000
0

Figure S17. a) ^{1}H-NMR spectrum of compound **1ak** in $CDCl_3$, 400 MHz, at 298 K; b) $^{13}C\{^{1}H\}$-NMR spectrum ($CDCl_3$, 100 MHz, 298 K); c) ^{1}H-^{1}H COSY spectrum ($CDCl_3$, 100 MHz, 298 K); d) ^{1}H-^{13}C HSQCed spectrum ($CDCl_3$, 400 MHz, 298 K); e) ^{1}H-^{13}C HMBC spectrum ($CDCl_3$, 400 MHz, 298 K); f) HRMS (ESI-MS) spectrum (*m/z*). Observed HRMS (left) with the theoretical isotope prediction (right).

a)

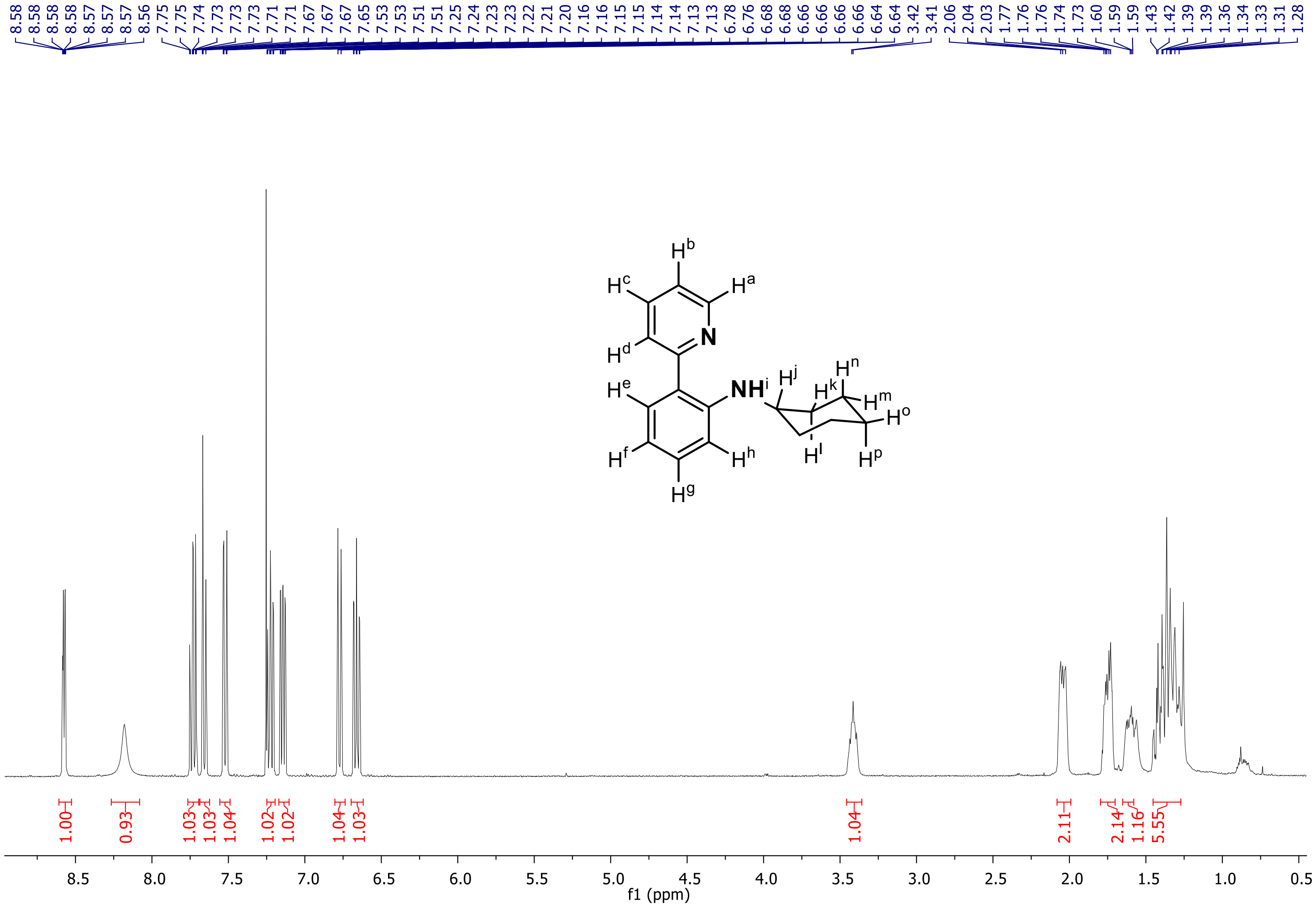

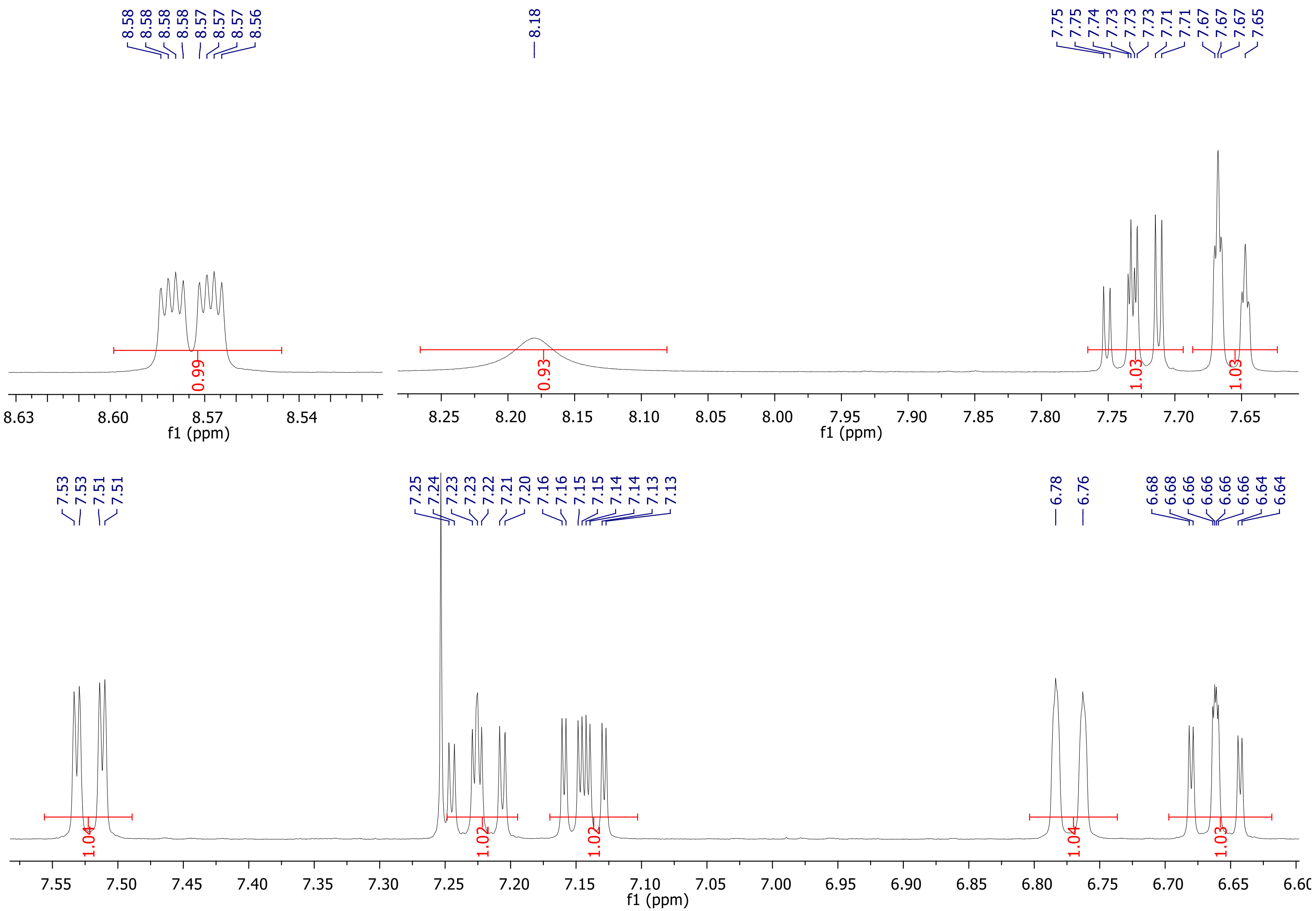

Selected aromatic region

b)

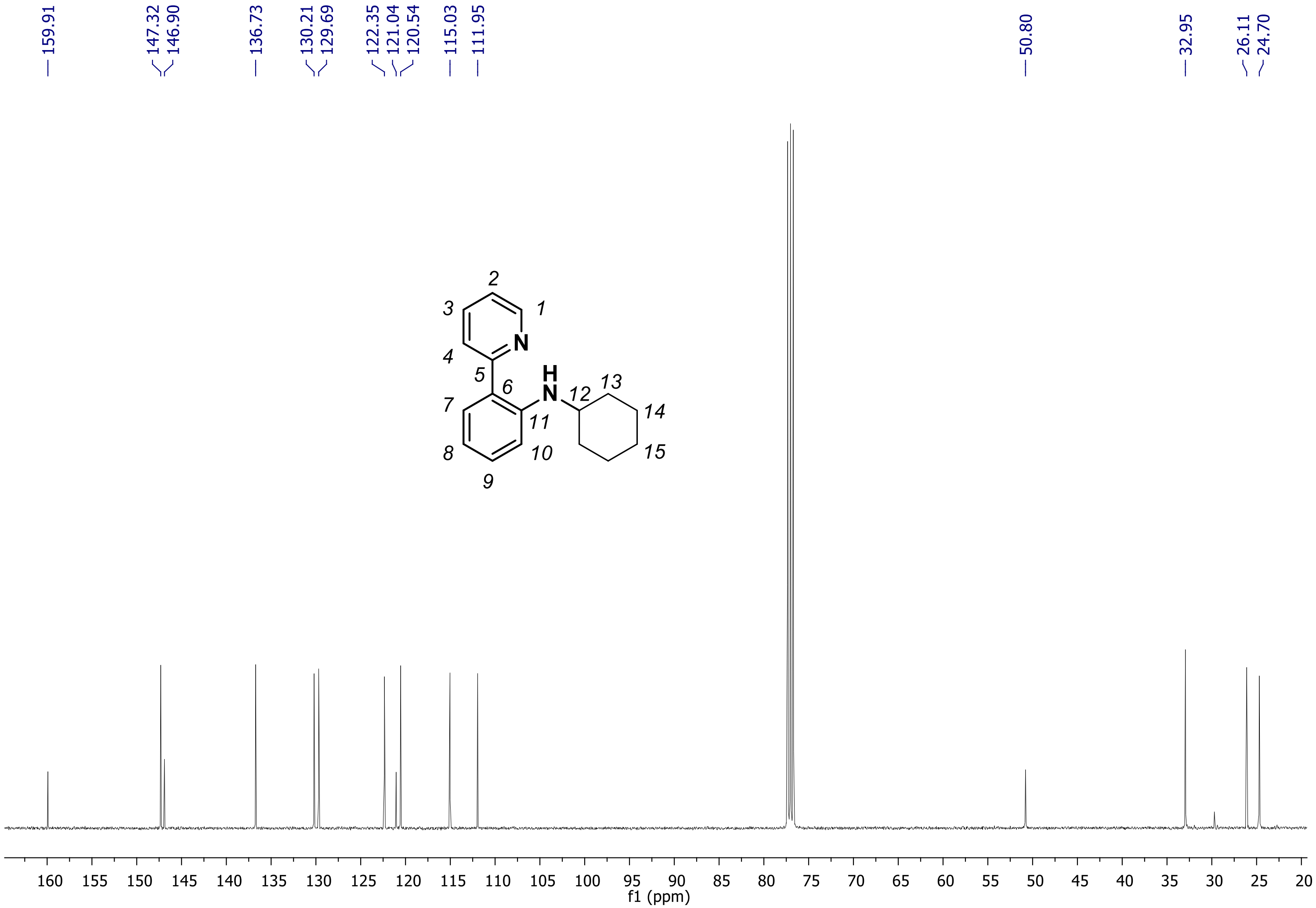
159.91
147.32
146.90
136.73
130.21
129.69
122.35
121.04
120.54
115.03
111.95
50.80
32.95
26.11
24.70
2
1
3
N
4
5
H
N
6
12
13
7
14
11
8
10
15
9
160 155 150 145 140 135 130 125 120 115 110 105 100 95 90 85 80 75 70 65 60 55 50 45 40 35 30 25 20
f1 (ppm)

c)

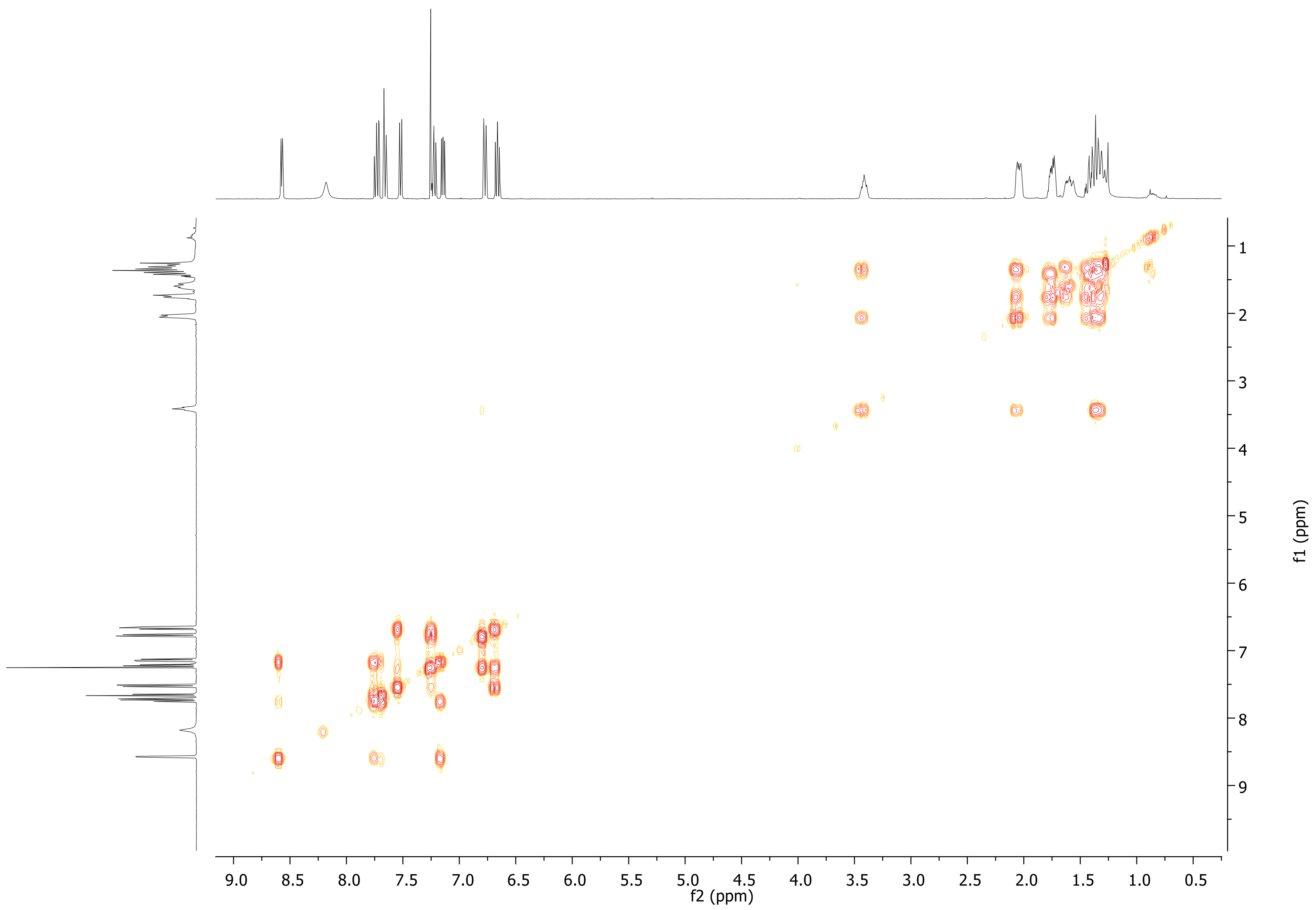

9.0
8.5
8.0
7.5
7.0
6.5
6.0
5.5
5.0
4.5
4.0
3.5
3.0
2.5
2.0
1.5
1.0
0.5
f2 (ppm)
1
2
3
4
5
6
7
8
9
f1 (ppm)

d)

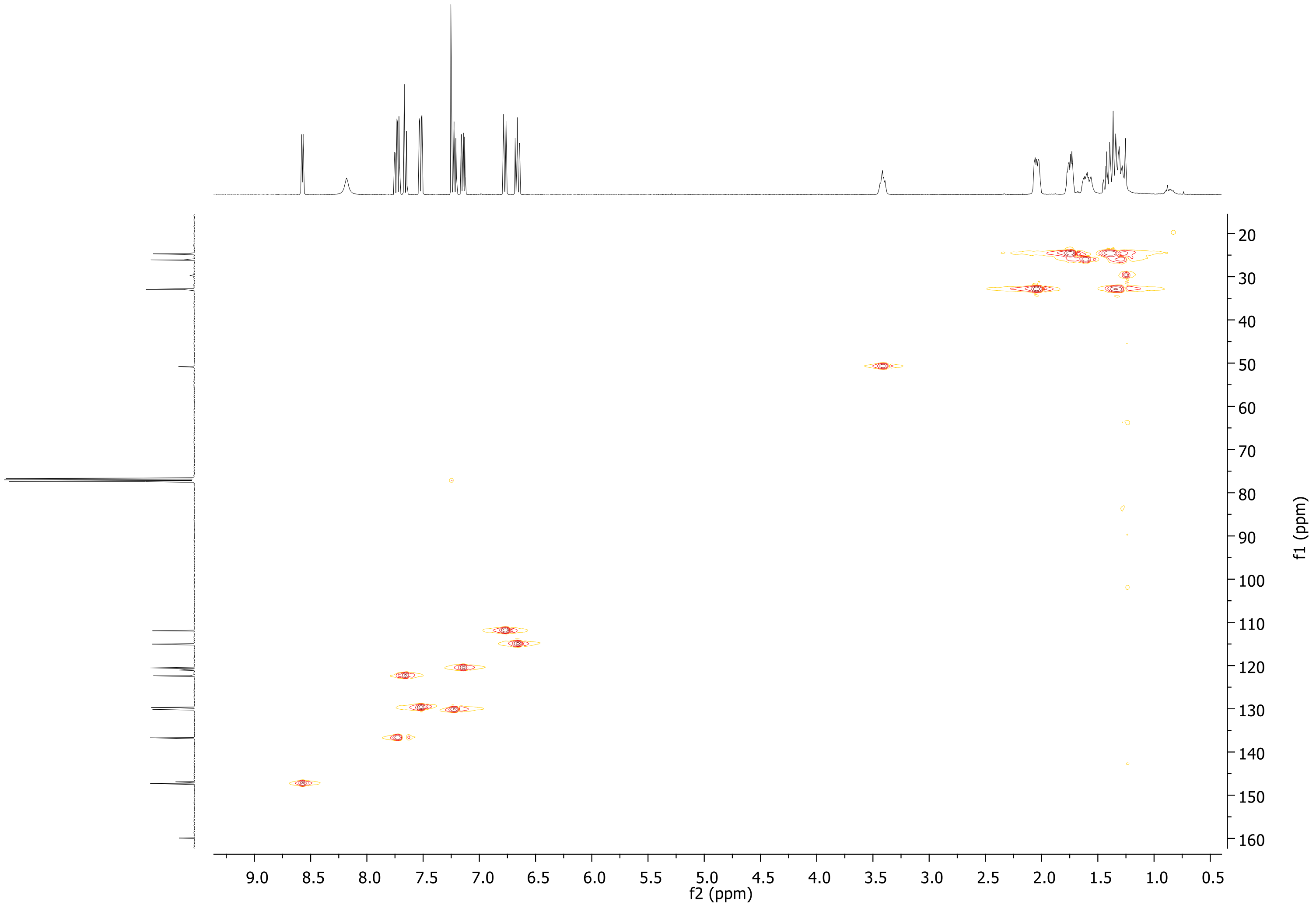
20
30
40
50
60
70
80
90
100
110
120
130
140
150
160
f1 (ppm)
9.0
8.5
8.0
7.5
7.0
6.5
6.0
5.5
5.0
4.5
4.0
3.5
3.0
2.5
2.0
1.5
1.0
0.5
f2 (ppm)

e)

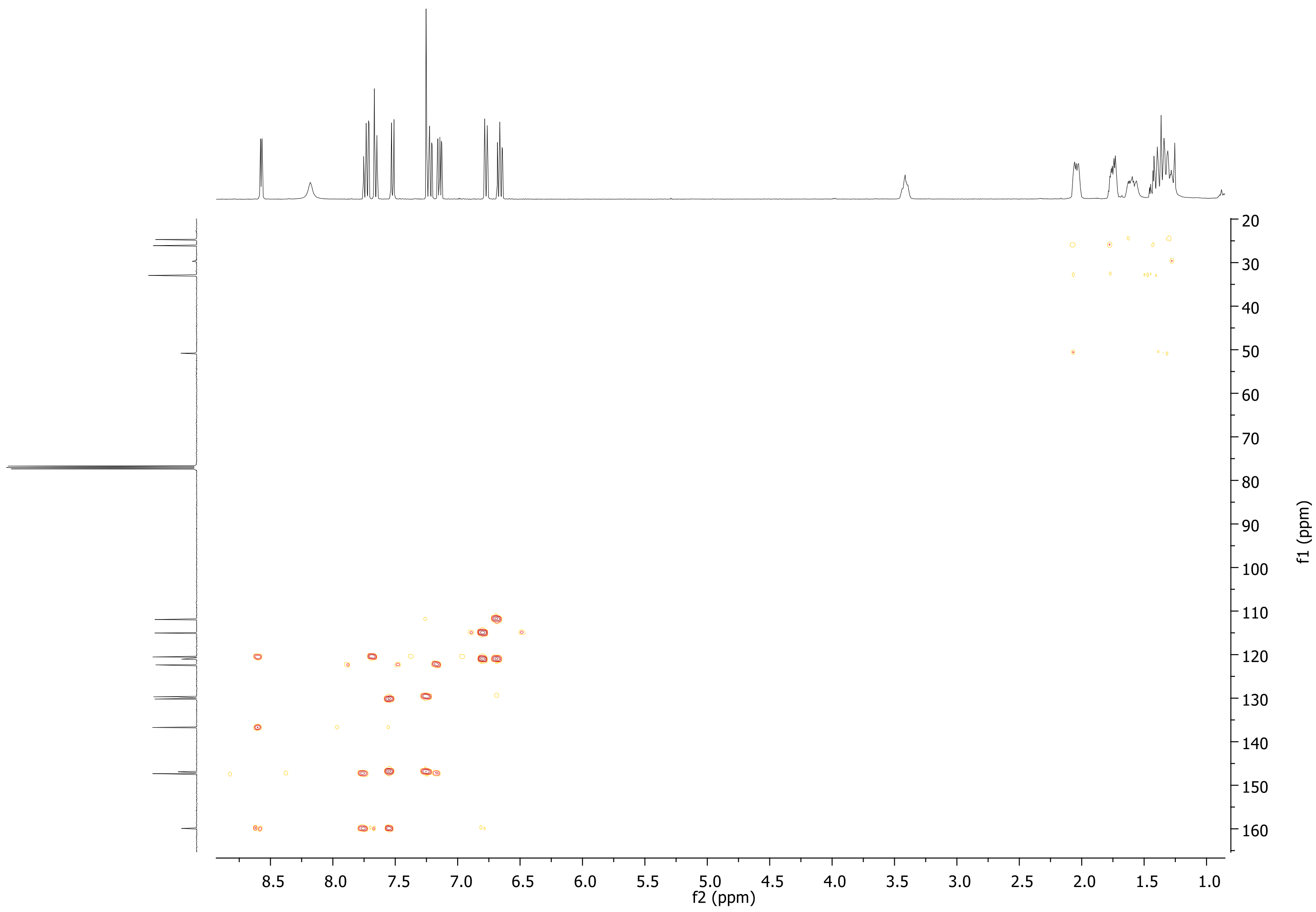

f)

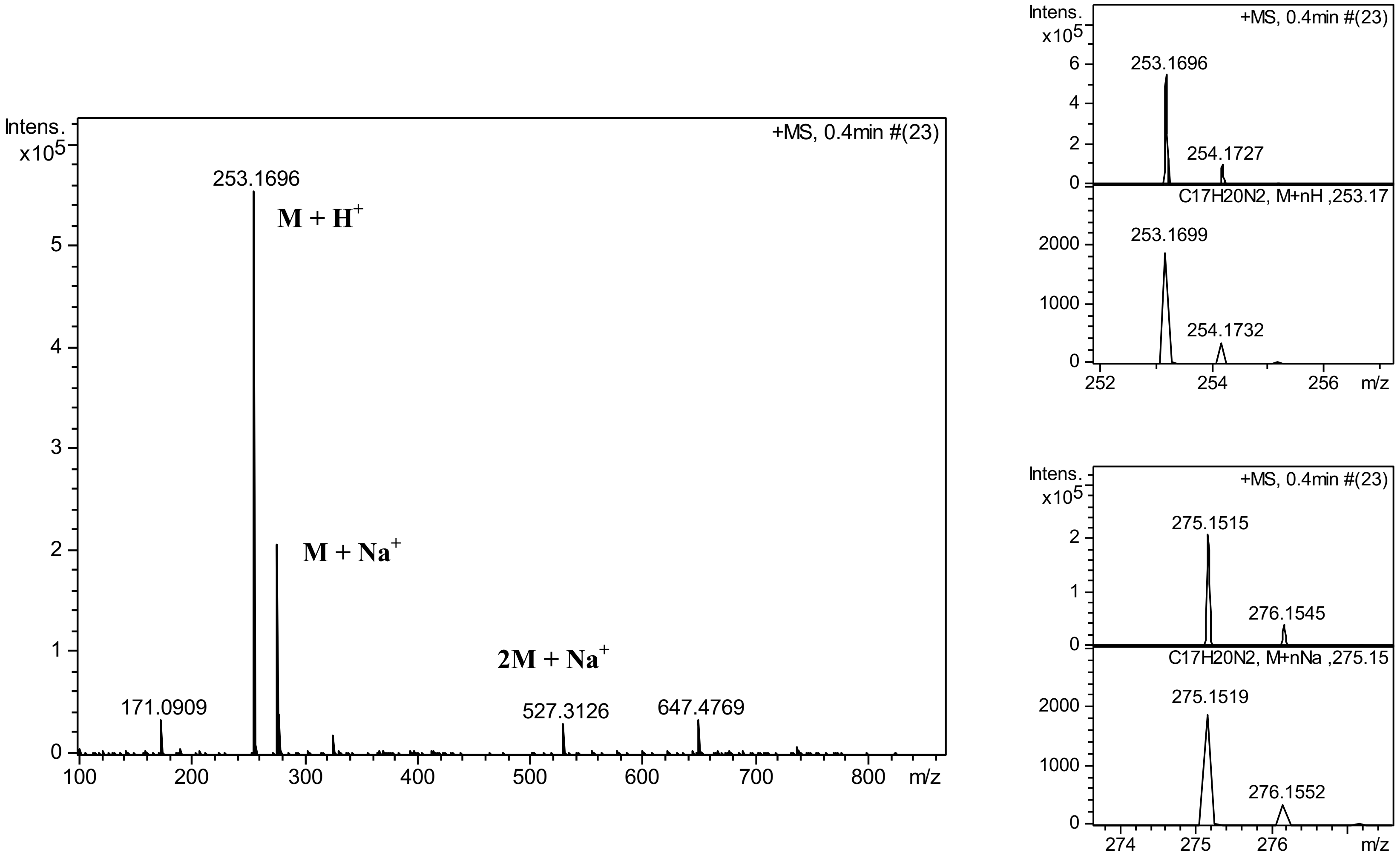

Intens.
x10^5
+MS, 0.4min #(23)
253.1696
M + H+
M + Na+
2M + Na+
171.0909
527.3126
647.4769
m/z
+MS, 0.4min #(23)
253.1696
254.1727
C17H20N2, M+nH ,253.17
253.1699
254.1732
+MS, 0.4min #(23)
275.1515
276.1545
C17H20N2, M+nNa ,275.15
275.1519
276.1552

Figure S18. a) ^{1}H-NMR spectrum of compound **1al** in $CDCl_3$, 400 MHz, at 298 K; b) $^{13}C\{^{1}H\}$-NMR spectrum ($CDCl_3$, 100 MHz, 298 K); c) ^{1}H-^{1}H COSY spectrum ($CDCl_3$, 100 MHz, 298 K); d) ^{1}H-^{13}C HSQCed spectrum ($CDCl_3$, 400 MHz, 298 K); e) ^{1}H-^{13}C HMBC spectrum ($CDCl_3$, 400 MHz, 298 K); f) HRMS (ESI-MS) spectrum (*m/z*). Observed HRMS (left) with the theoretical isotope prediction (right).

a)

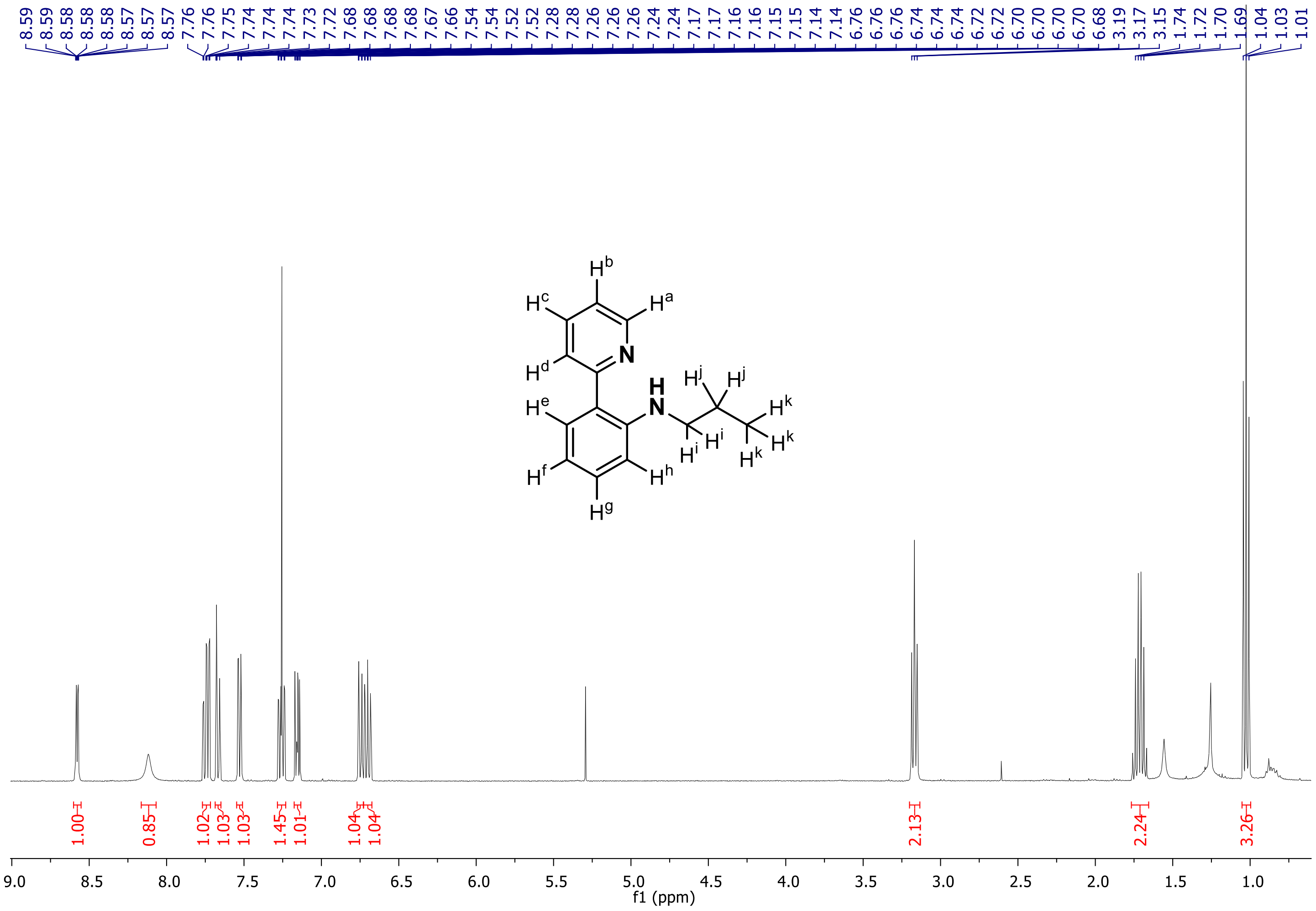

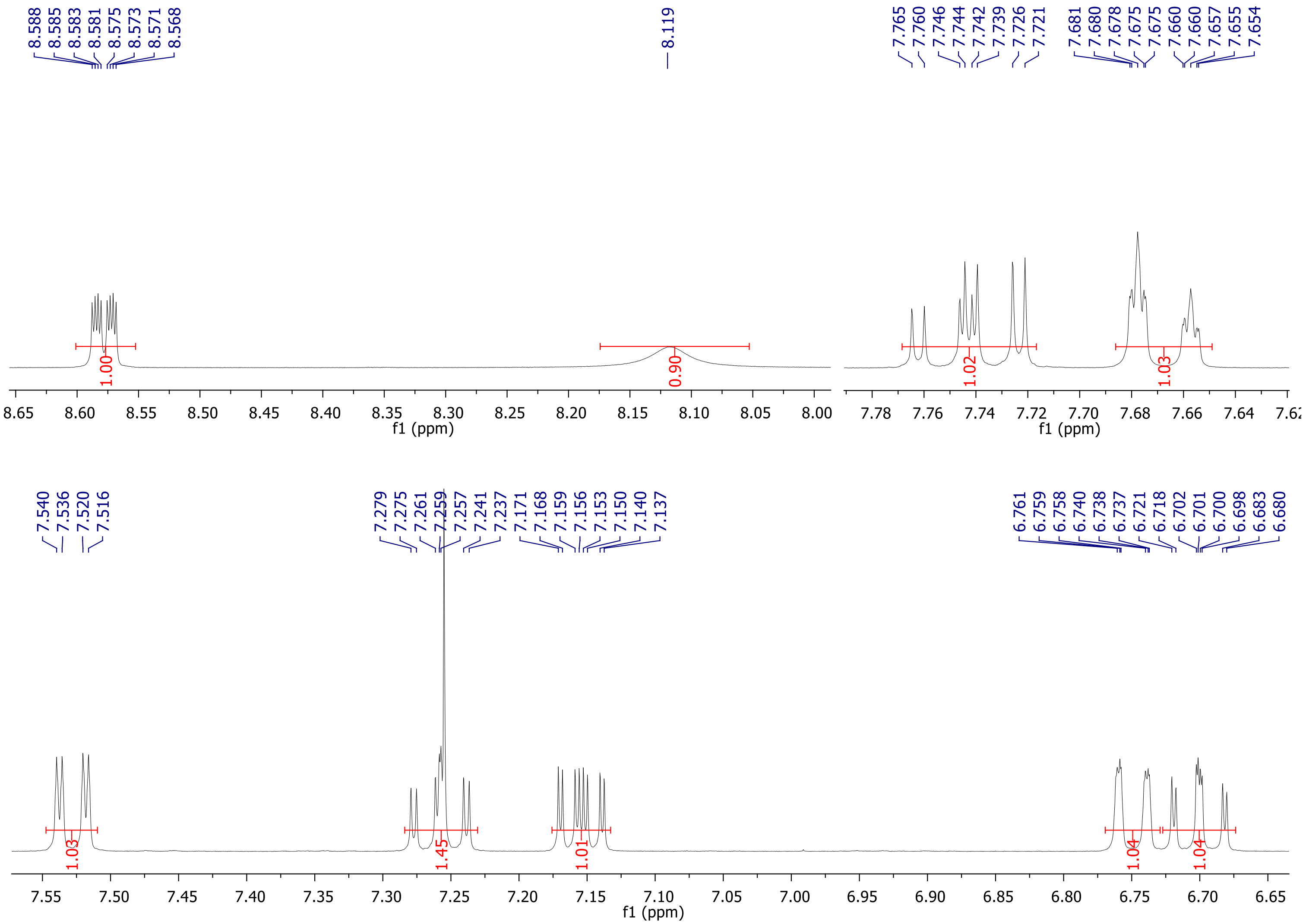

Selected aromatic region

b)

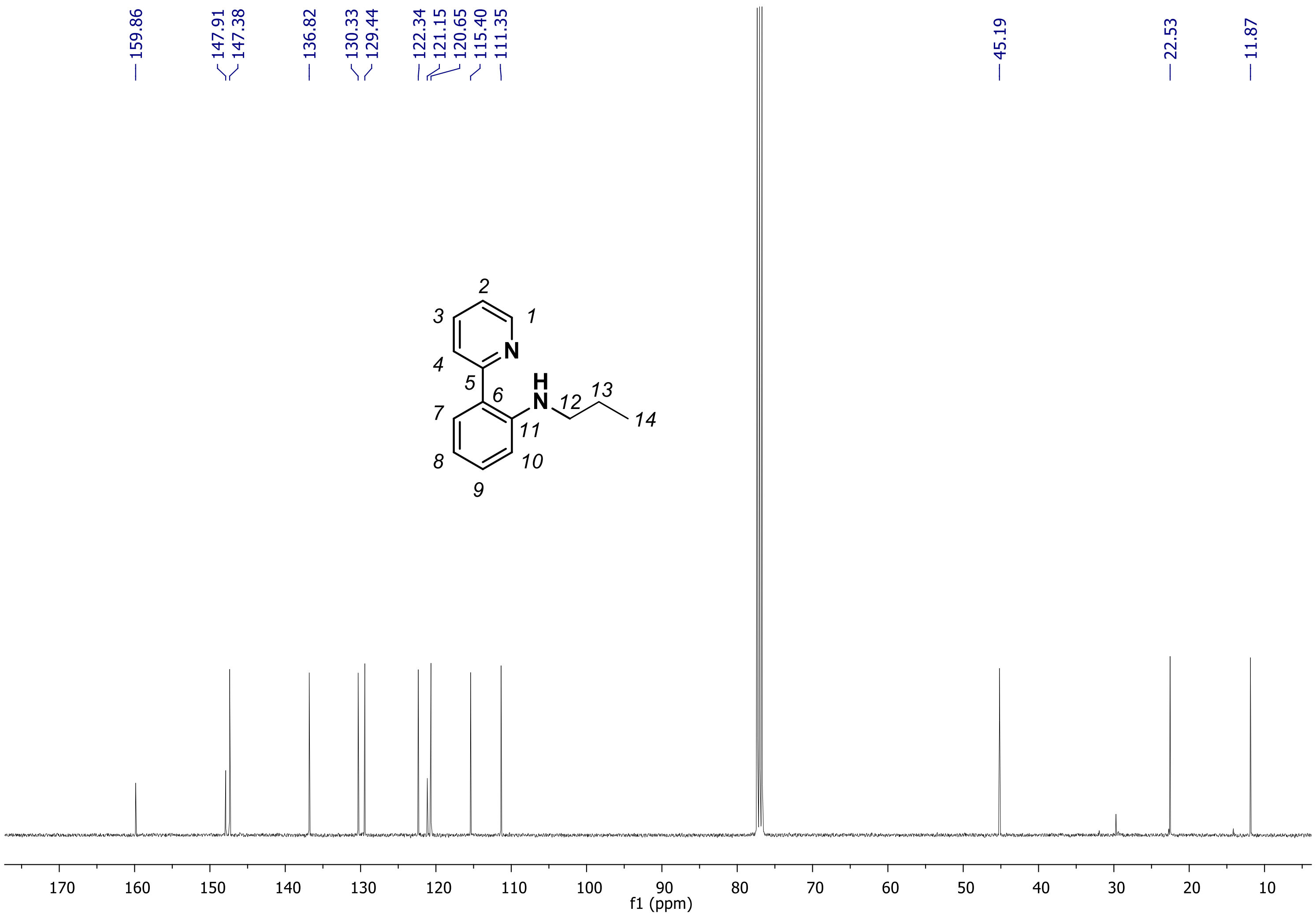
159.86
147.91
147.38
136.82
130.33
129.44
122.34
121.15
120.65
115.40
111.35
45.19
22.53
11.87
N
H
N
1
2
3
4
5
6
7
8
9
10
11
12
13
14
170
160
150
140
130
120
110
100
90
80
70
60
50
40
30
20
10
f1 (ppm)

c)

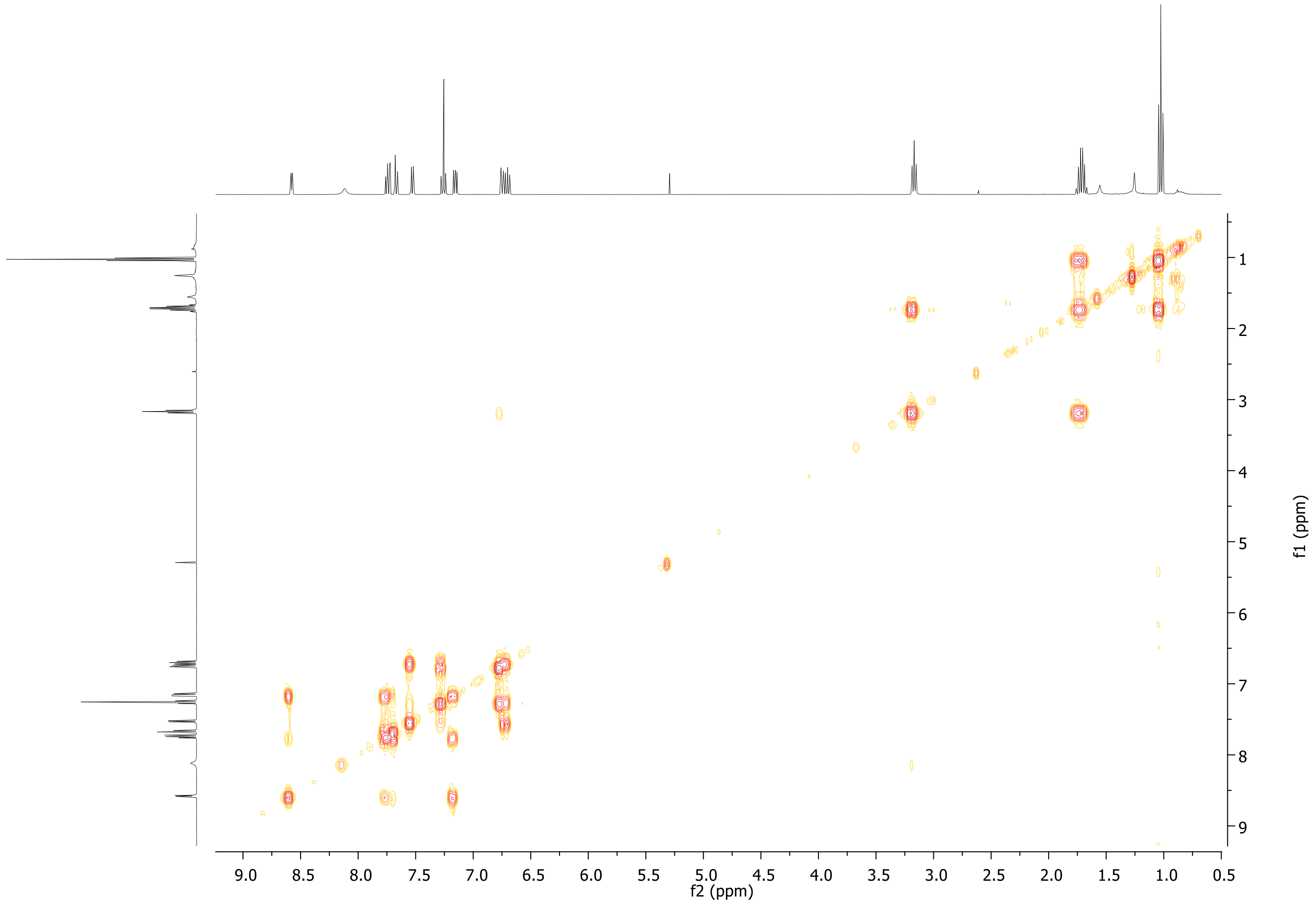

9.0 8.5 8.0 7.5 7.0 6.5 6.0 5.5 5.0 4.5 4.0 3.5 3.0 2.5 2.0 1.5 1.0 0.5
f2 (ppm)
1 2 3 4 5 6 7 8 9
f1 (ppm)

d)

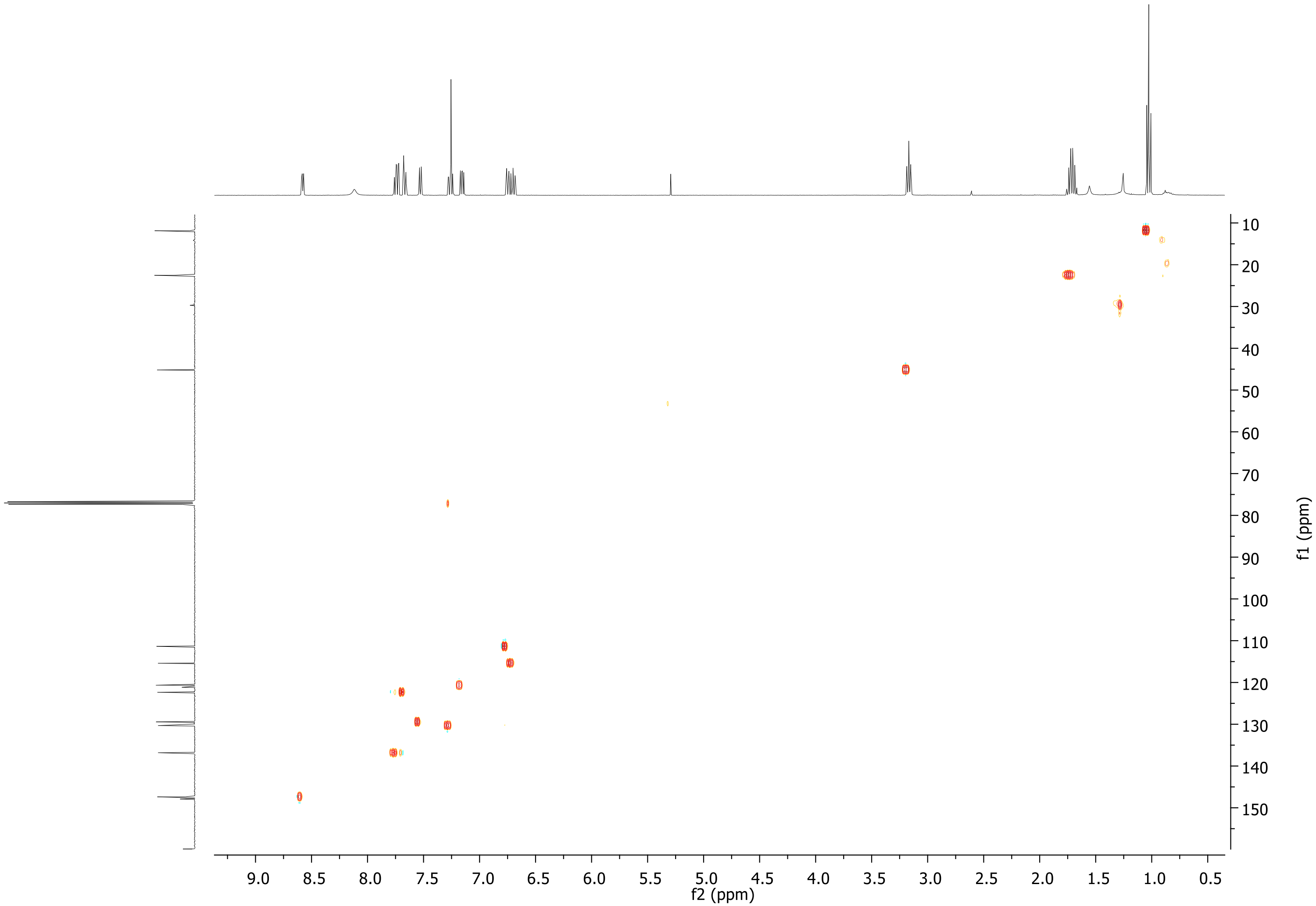

f2 (ppm)
f1 (ppm)
9.0 8.5 8.0 7.5 7.0 6.5 6.0 5.5 5.0 4.5 4.0 3.5 3.0 2.5 2.0 1.5 1.0 0.5
10 20 30 40 50 60 70 80 90 100 110 120 130 140 150

e)

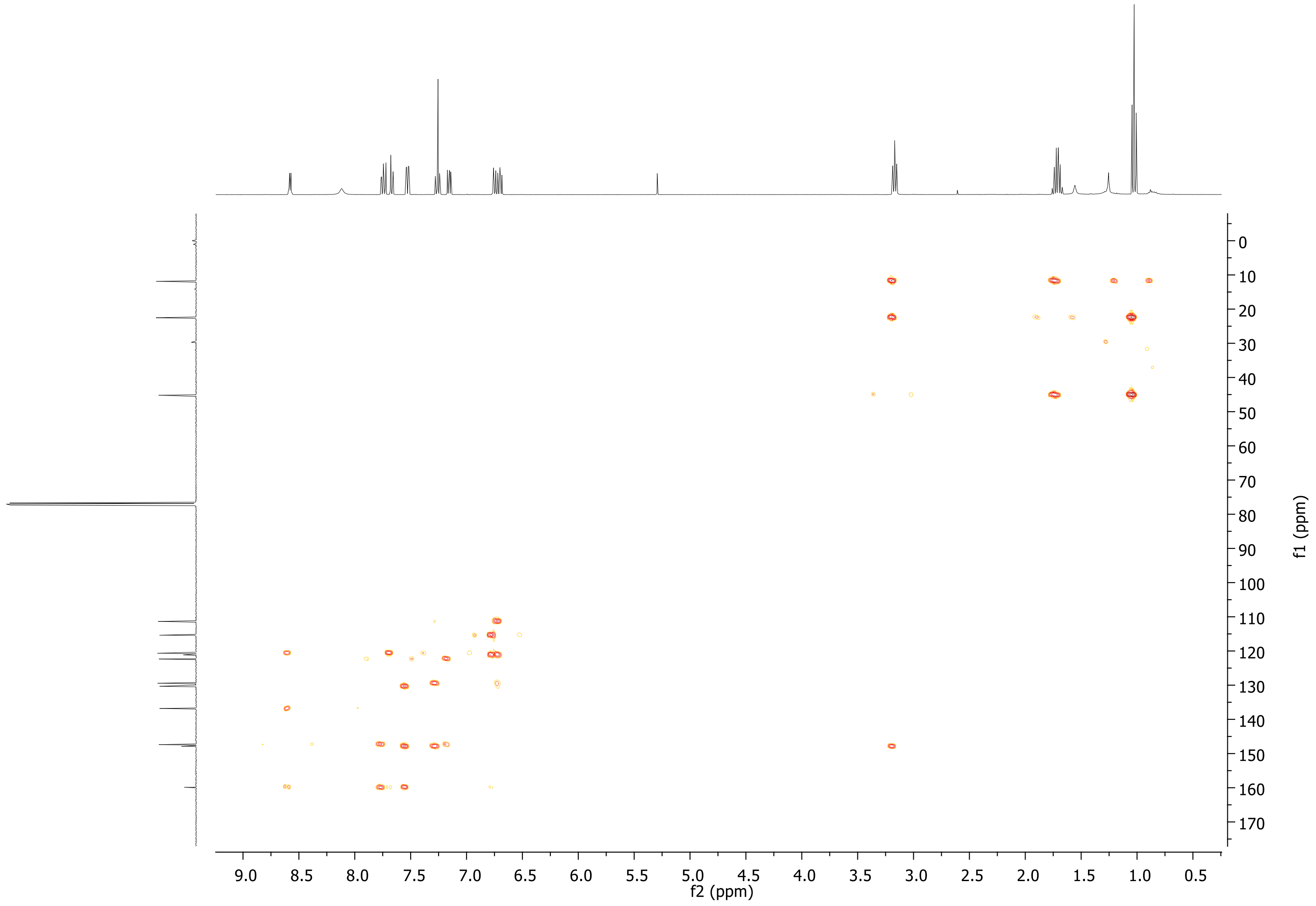

f)

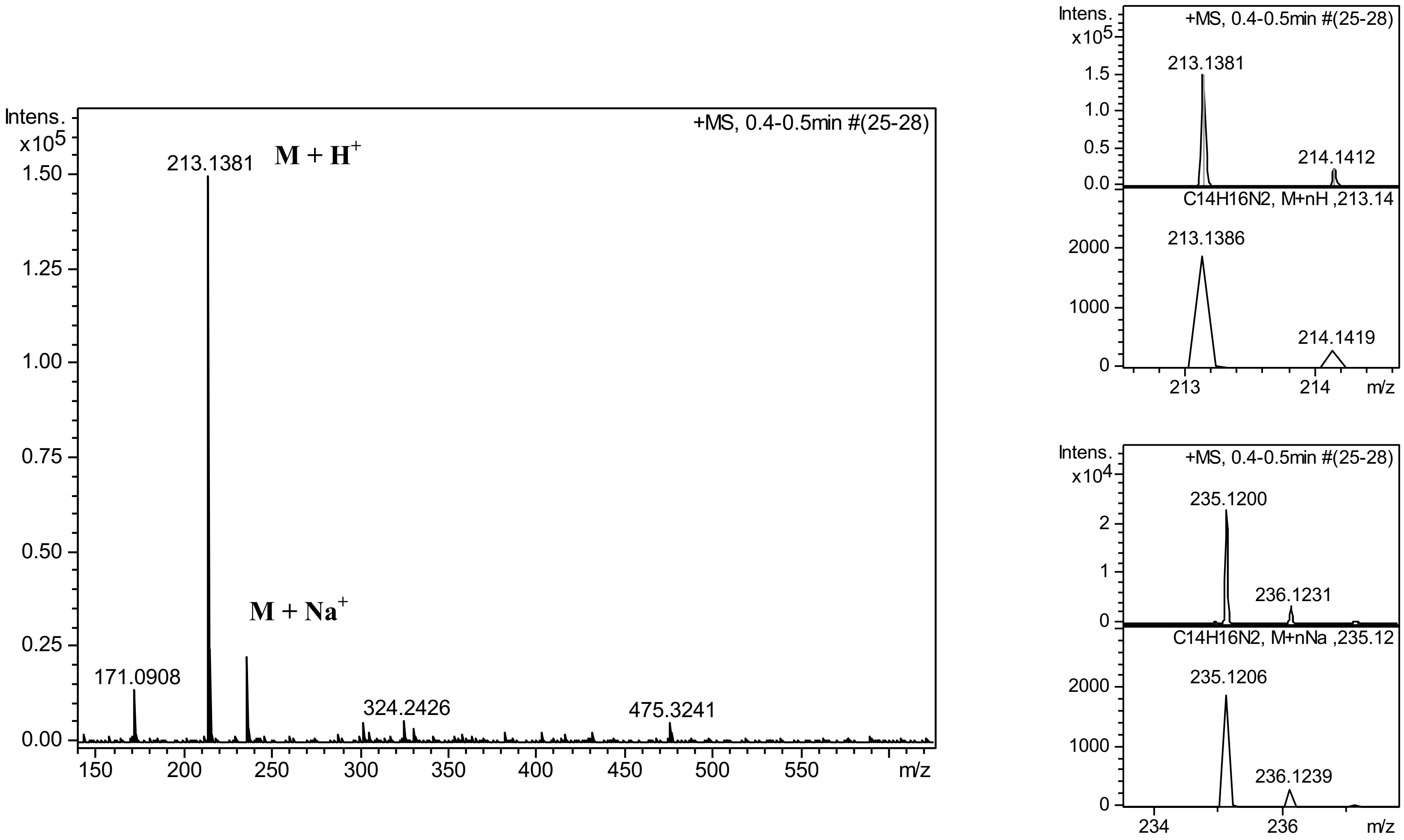
Intens.
x10^5
+MS, 0.4-0.5min #(25-28)
213.1381
M + H+
M + Na+
171.0908
324.2426
475.3241
1.50
1.25
1.00
0.75
0.50
0.25
0.00
150
200
250
300
350
400
450
500
550
m/z
Intens.
x10^5
+MS, 0.4-0.5min #(25-28)
213.1381
214.1412
C14H16N2, M+nH ,213.14
213.1386
214.1419
213
214
m/z
Intens.
x10^4
+MS, 0.4-0.5min #(25-28)
235.1200
236.1231
C14H16N2, M+nNa ,235.12
235.1206
236.1239
234
236
m/z

Figure S19. a) ^{1}H-NMR spectrum of compound **1am** in $CDCl_3$, 400 MHz, at 298 K; b) $^{13}C\{^{1}H\}$-NMR spectrum ($CDCl_3$, 100 MHz, 298 K); c) ^{1}H-^{1}H COSY spectrum ($CDCl_3$, 100 MHz, 298 K); d) ^{1}H-^{13}C HSQCed spectrum ($CDCl_3$, 400 MHz, 298 K); e) ^{1}H-^{13}C HMBC spectrum ($CDCl_3$, 400 MHz, 298 K); f) HRMS (ESI-MS) spectrum (*m/z*). Observed HRMS (left) with the theoretical isotope prediction (right).

a)

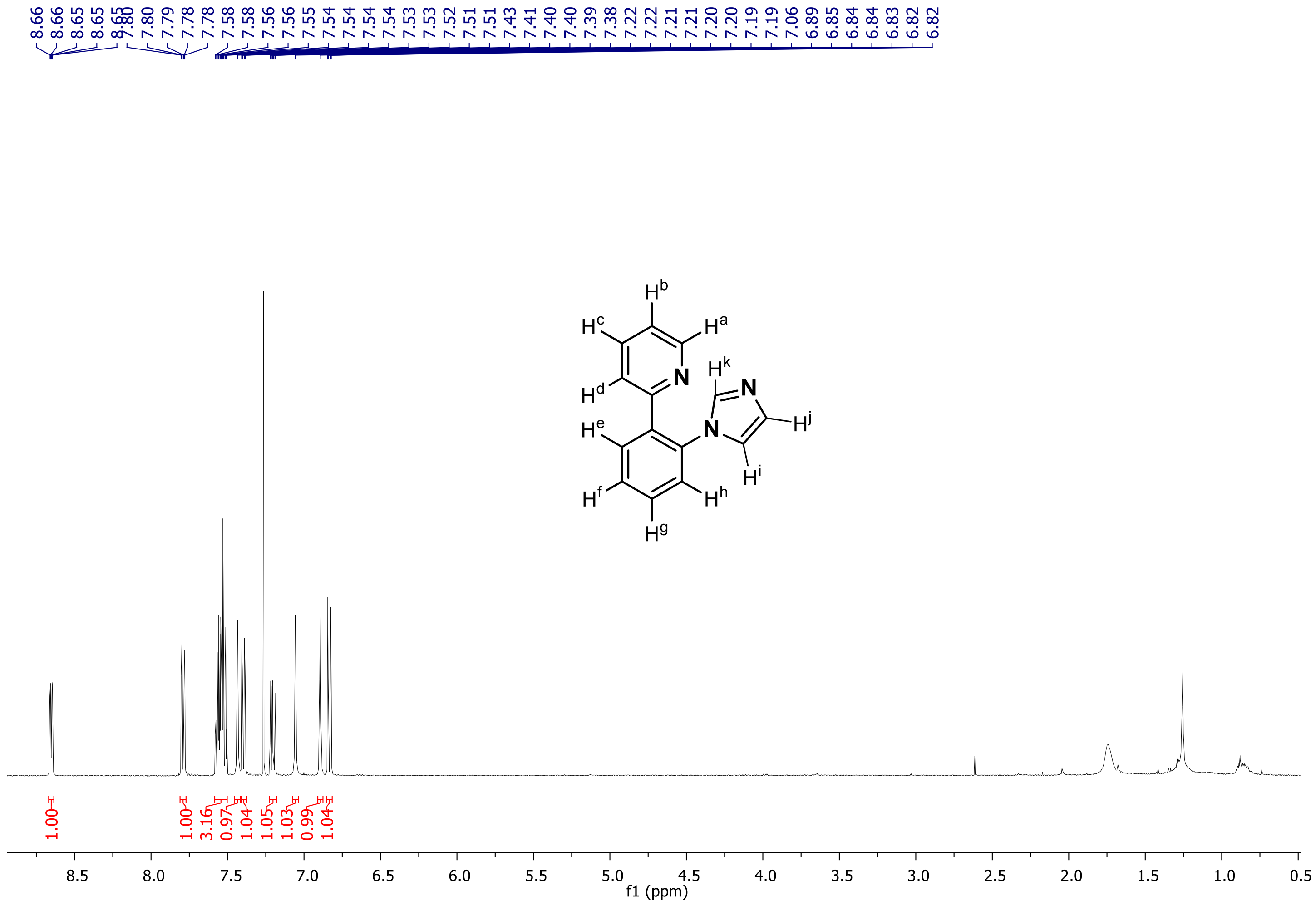

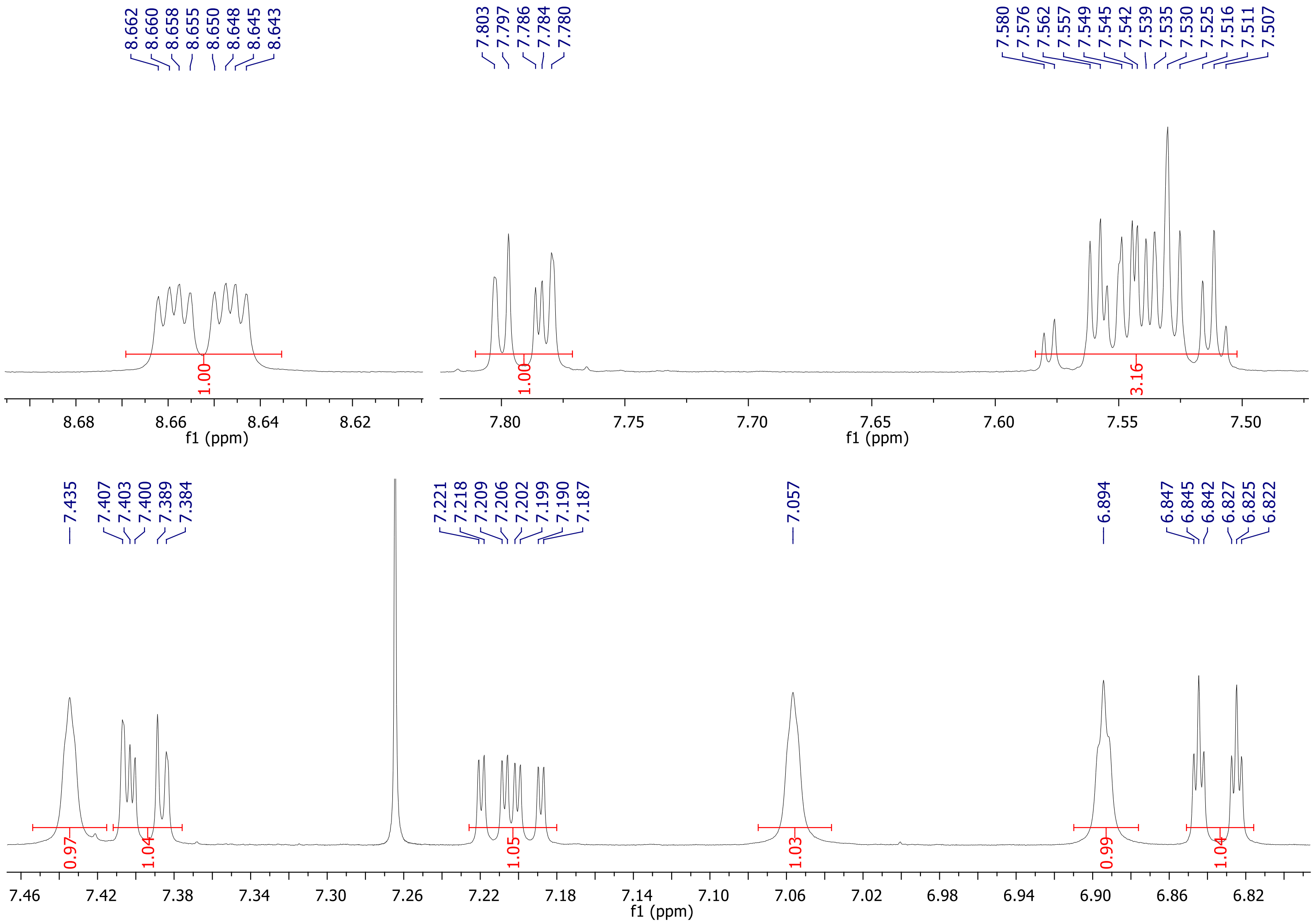

Selected aromatic region

b)

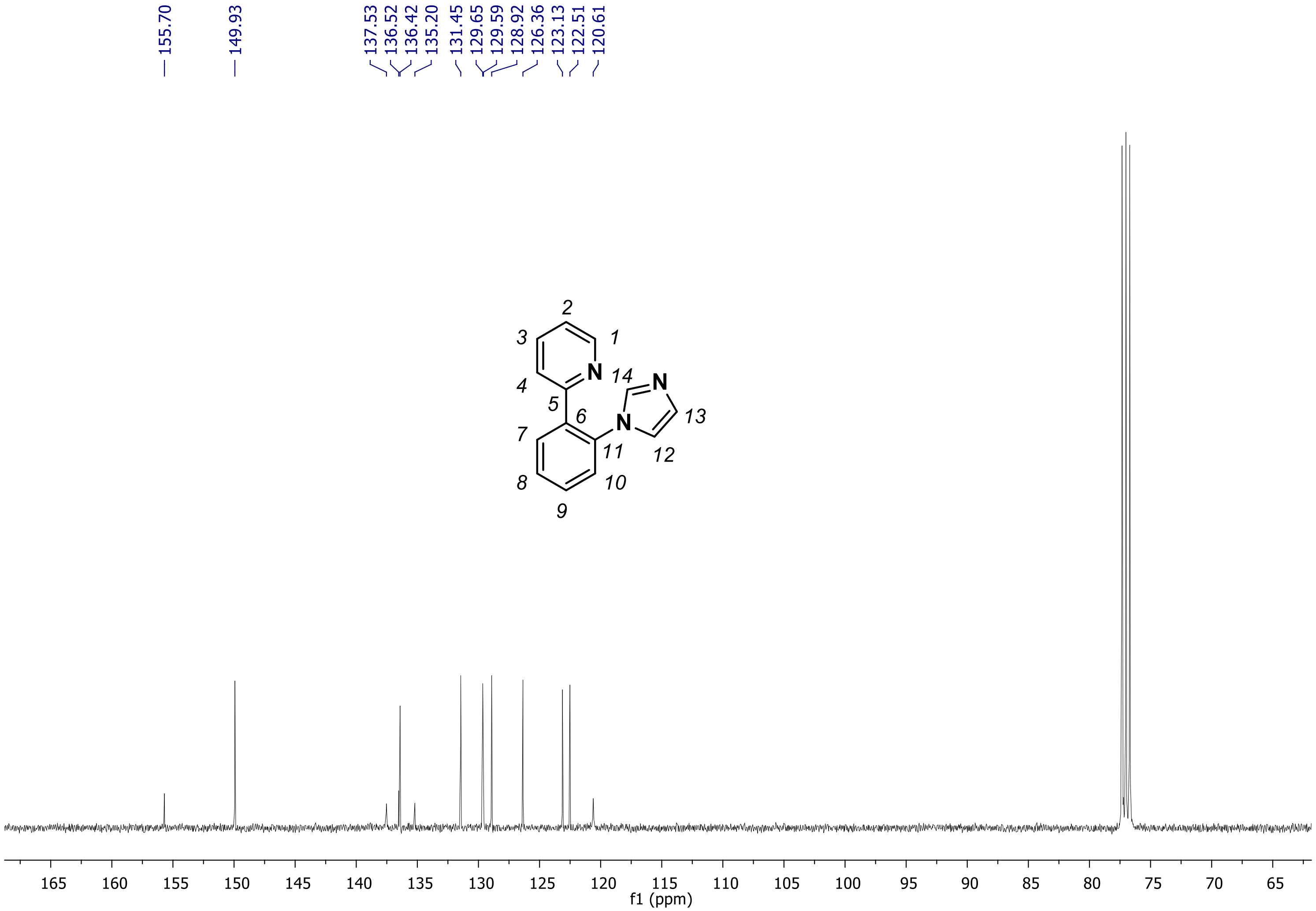
155.70
149.93
137.53
136.52
136.42
135.20
131.45
129.65
129.59
128.92
126.36
123.13
122.51
120.61
1
2
3
4
5
6
7
8
9
10
11
12
13
14
N
N
N
165
160
155
150
145
140
135
130
125
120
115
110
105
100
95
90
85
80
75
70
65
f1 (ppm)

c)

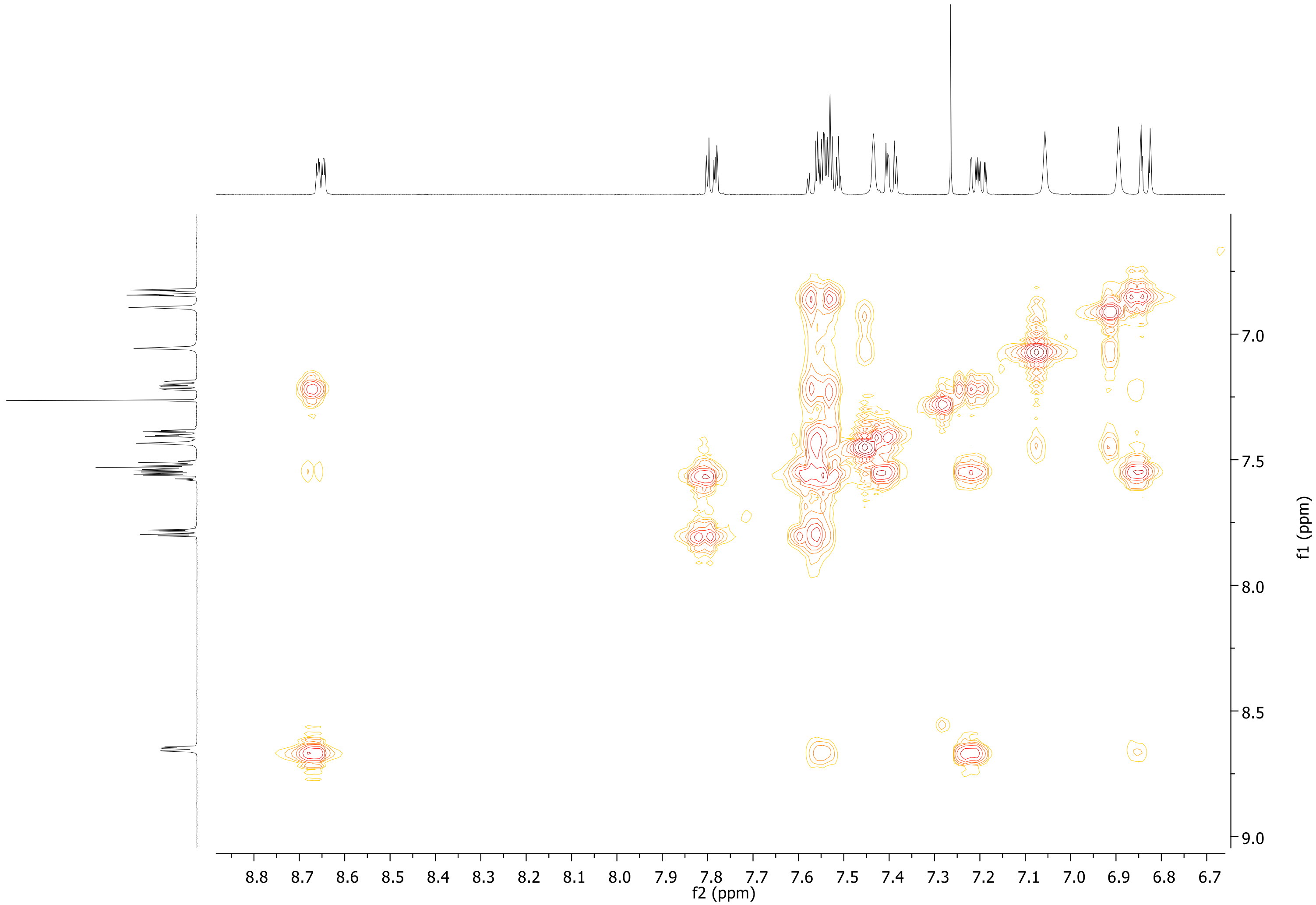

8.8
8.7
8.6
8.5
8.4
8.3
8.2
8.1
8.0
7.9
7.8
7.7
7.6
7.5
7.4
7.3
7.2
7.1
7.0
6.9
6.8
6.7
f2 (ppm)
7.0
7.5
8.0
8.5
9.0
f1 (ppm)

d)

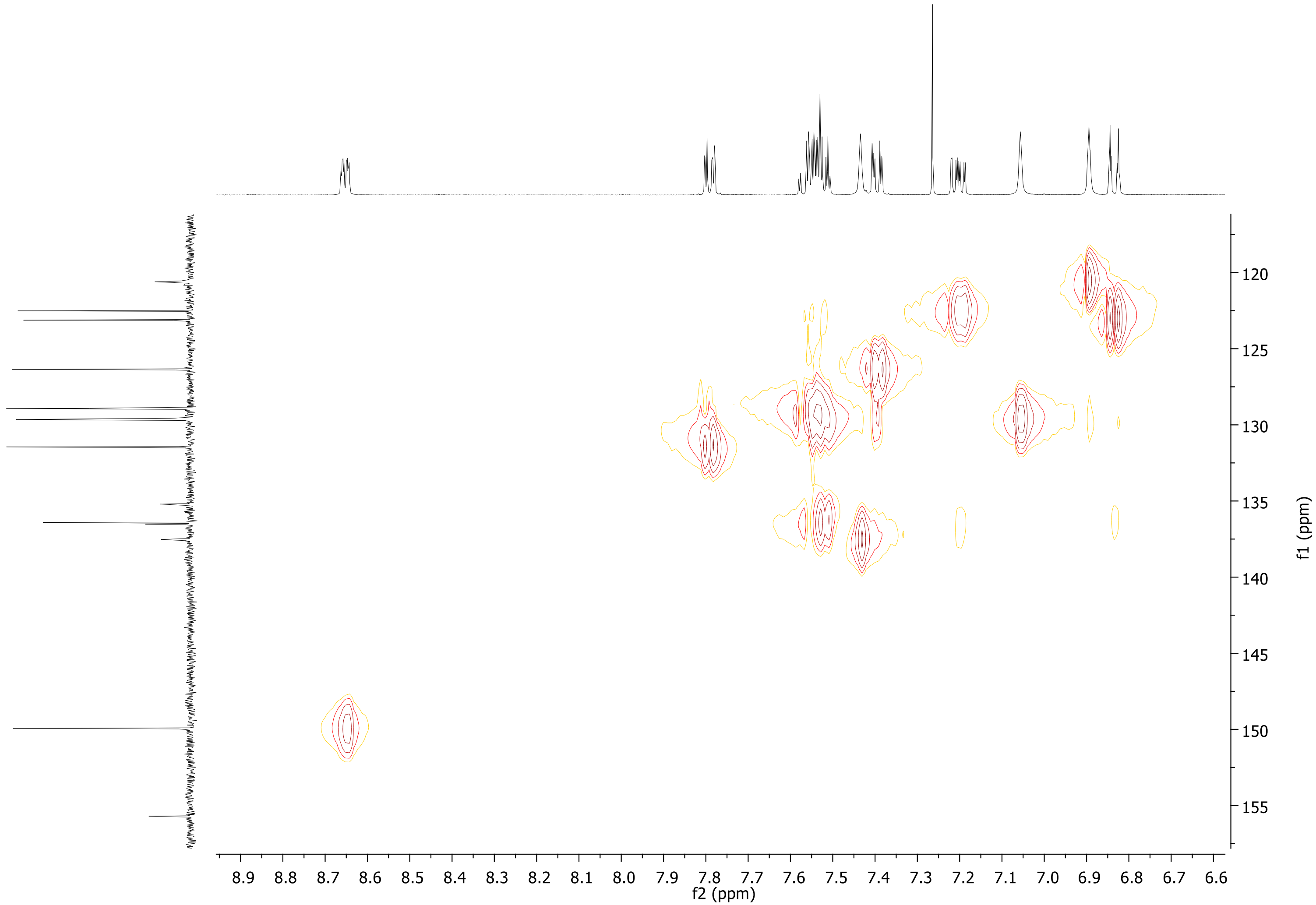

e)

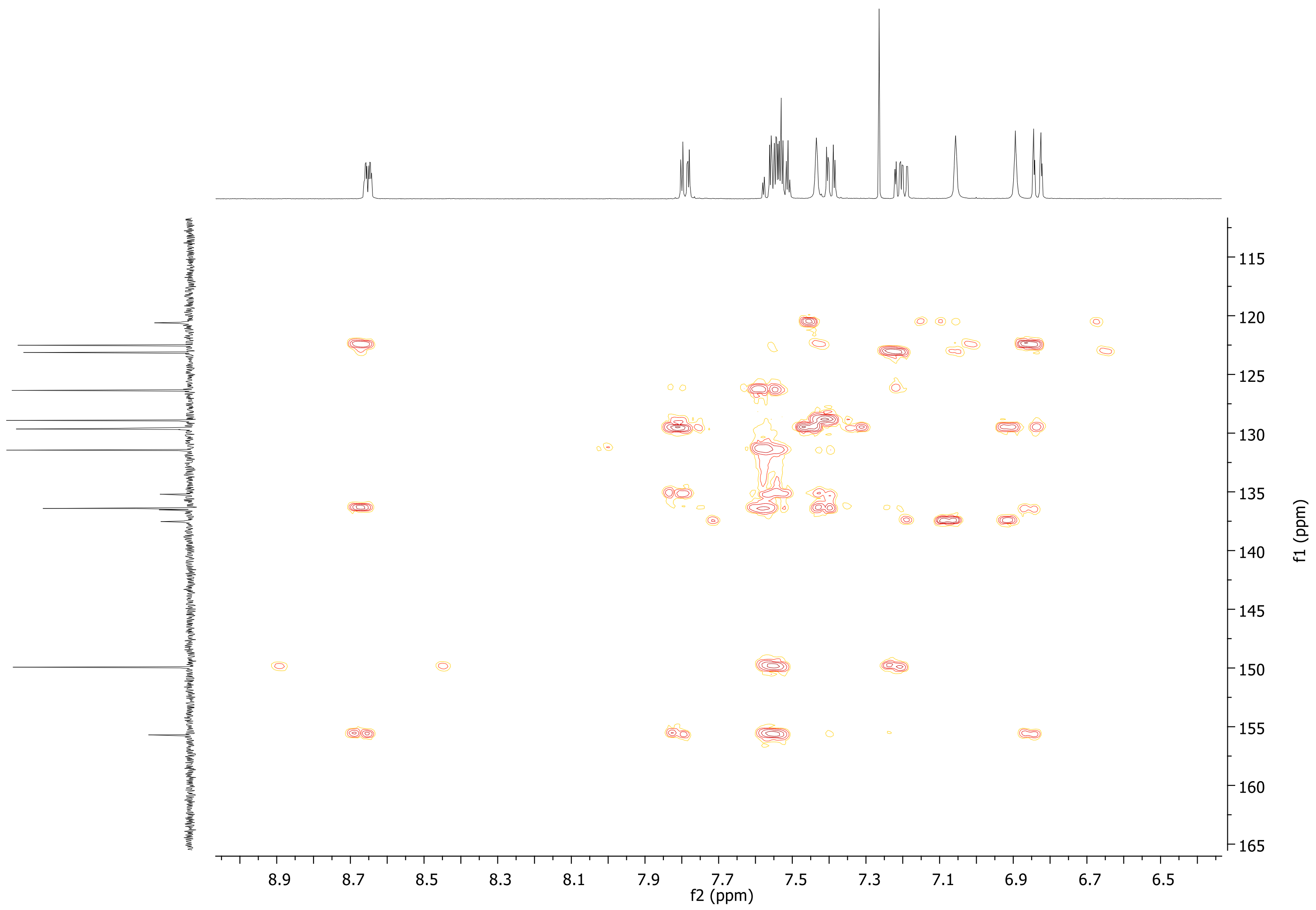

115
120
125
130
135
140
145
150
155
160
165
f1 (ppm)
8.9
8.7
8.5
8.3
8.1
7.9
7.7
7.5
7.3
7.1
6.9
6.7
6.5
f2 (ppm)

f)

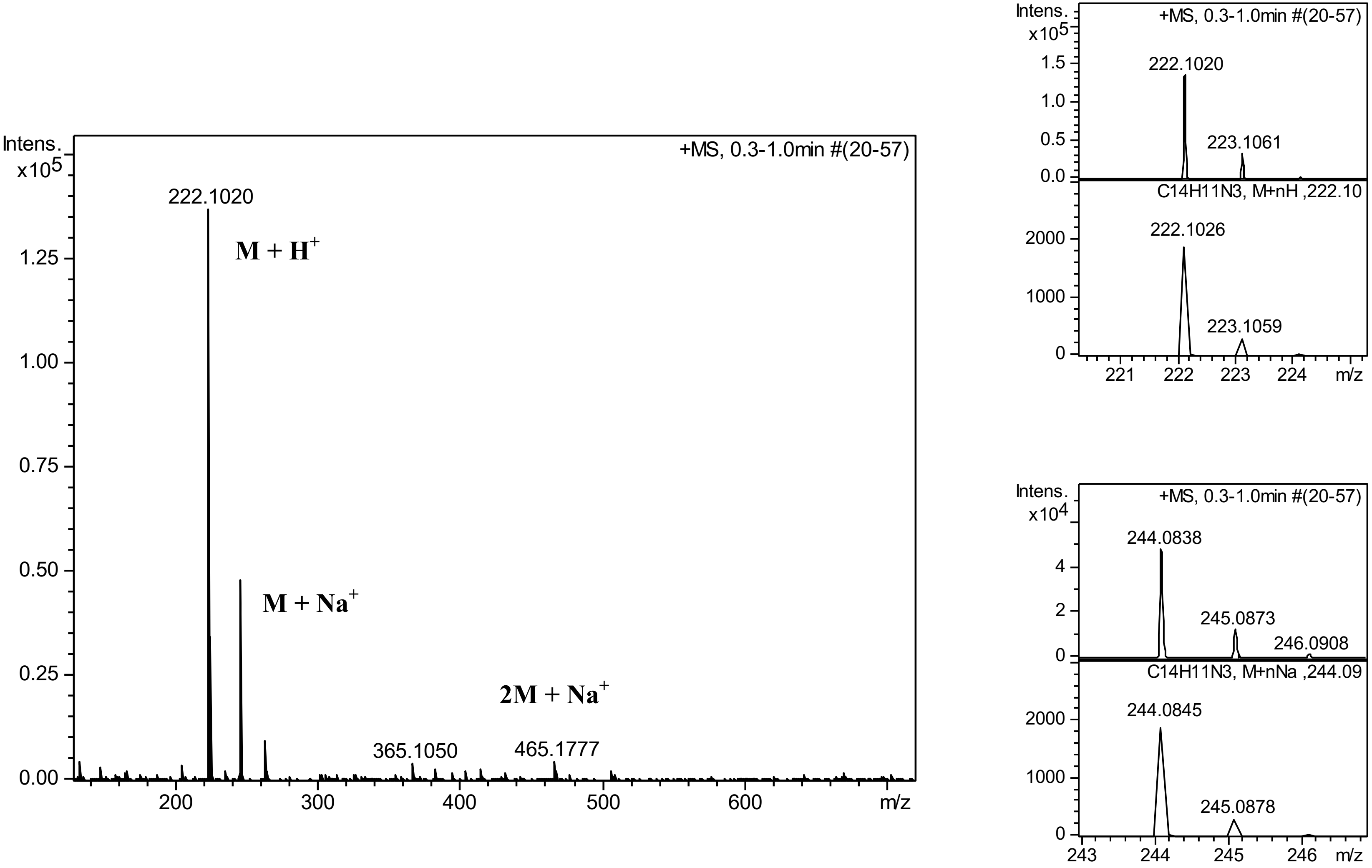
Intens.
x10^5
+MS, 0.3-1.0min #(20-57)
222.1020
M + H+
M + Na+
2M + Na+
365.1050
465.1777
1.25
1.00
0.75
0.50
0.25
0.00
200
300
400
500
600
m/z
Intens.
x10^5
+MS, 0.3-1.0min #(20-57)
1.5
1.0
0.5
0.0
222.1020
223.1061
C14H11N3, M+nH ,222.10
2000
1000
0
222.1026
223.1059
221
222
223
224
m/z
Intens.
x10^4
+MS, 0.3-1.0min #(20-57)
4
2
0
244.0838
245.0873
246.0908
C14H11N3, M+nNa ,244.09
2000
1000
0
244.0845
245.0878
243
244
245
246
m/z

Figure S20. a) ^{1}H-NMR spectrum of compound **1an** in $CDCl_3$, 400 MHz, at 298 K; b) HRMS (ESI-MS) spectrum (*m/z*). Observed HRMS (left) with the theoretical isotope prediction (right).

a)

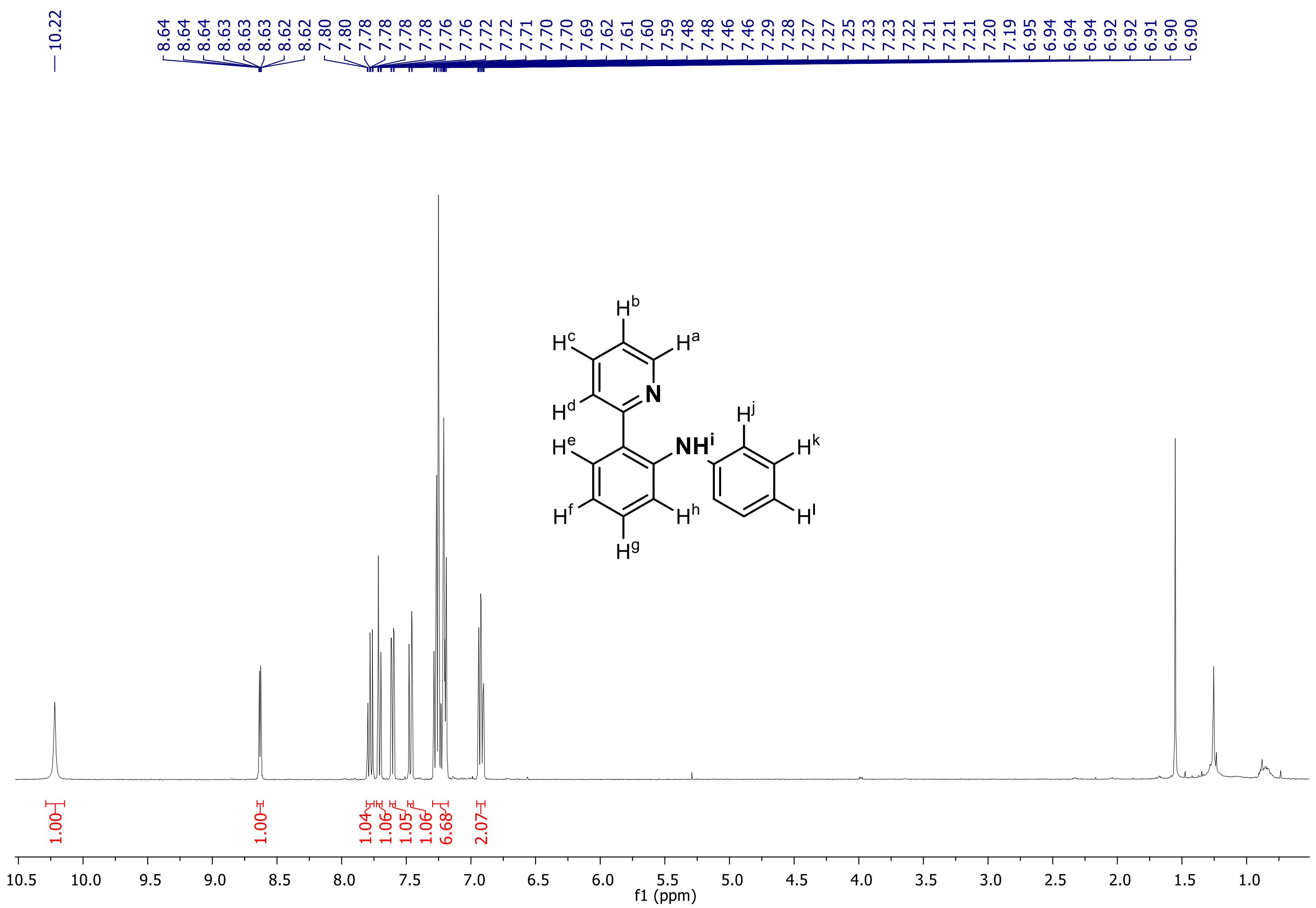

b)

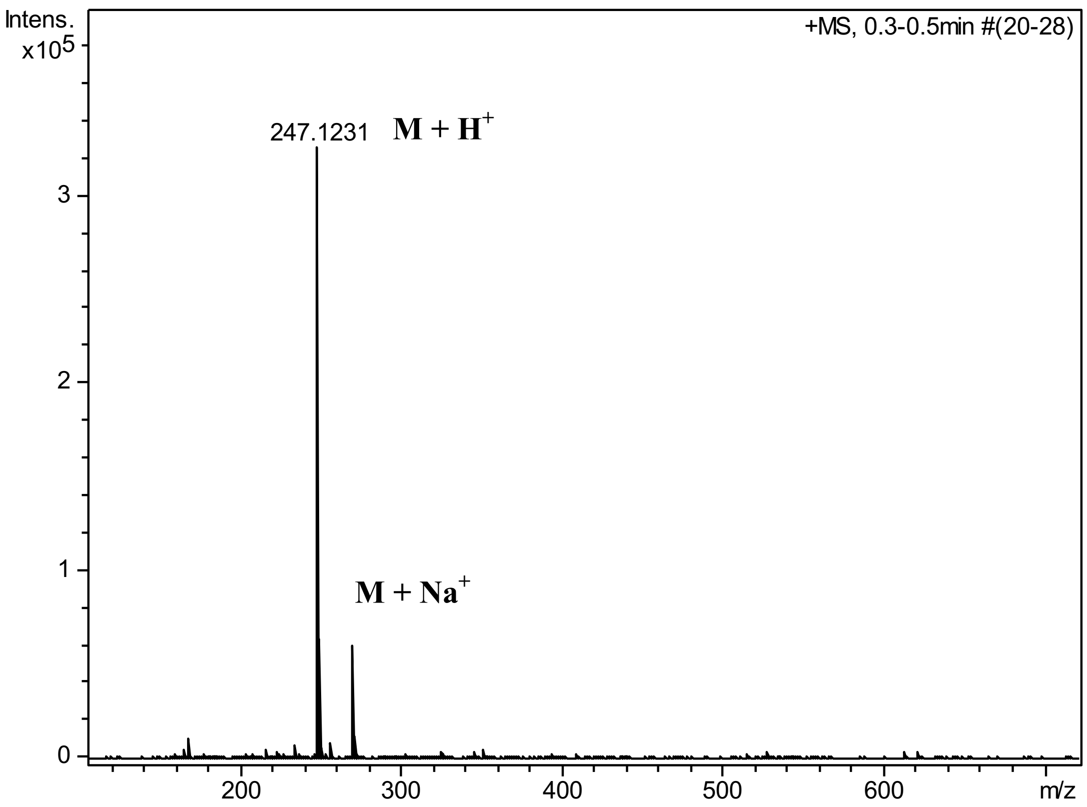

Intens.
x10^5
+MS, 0.3-0.5min #(20-28)
247.1231
M + H+
M + Na+
m/z

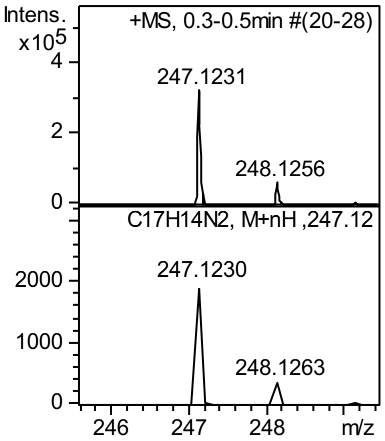

Intens.
x10^5
+MS, 0.3-0.5min #(20-28)
247.1231
248.1256
C17H14N2, M+nH ,247.12
247.1230
248.1263
m/z

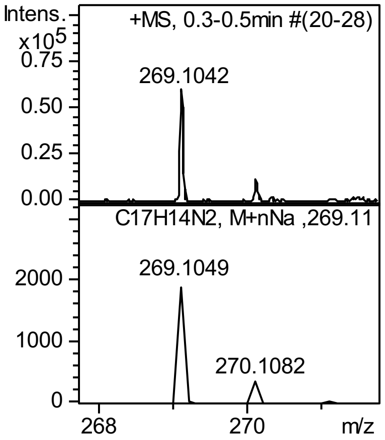

Intens.
x10^5
+MS, 0.3-0.5min #(20-28)
269.1042
C17H14N2, M+nNa ,269.11
269.1049
270.1082
m/z

Figure S21. a) ^{1}H-NMR spectrum of compound **1ap** in $CDCl_3$, 400 MHz, at 298 K; b) HRMS (ESI-MS) spectrum (*m/z*). Observed HRMS (left) with the theoretical isotope prediction (right).

a)

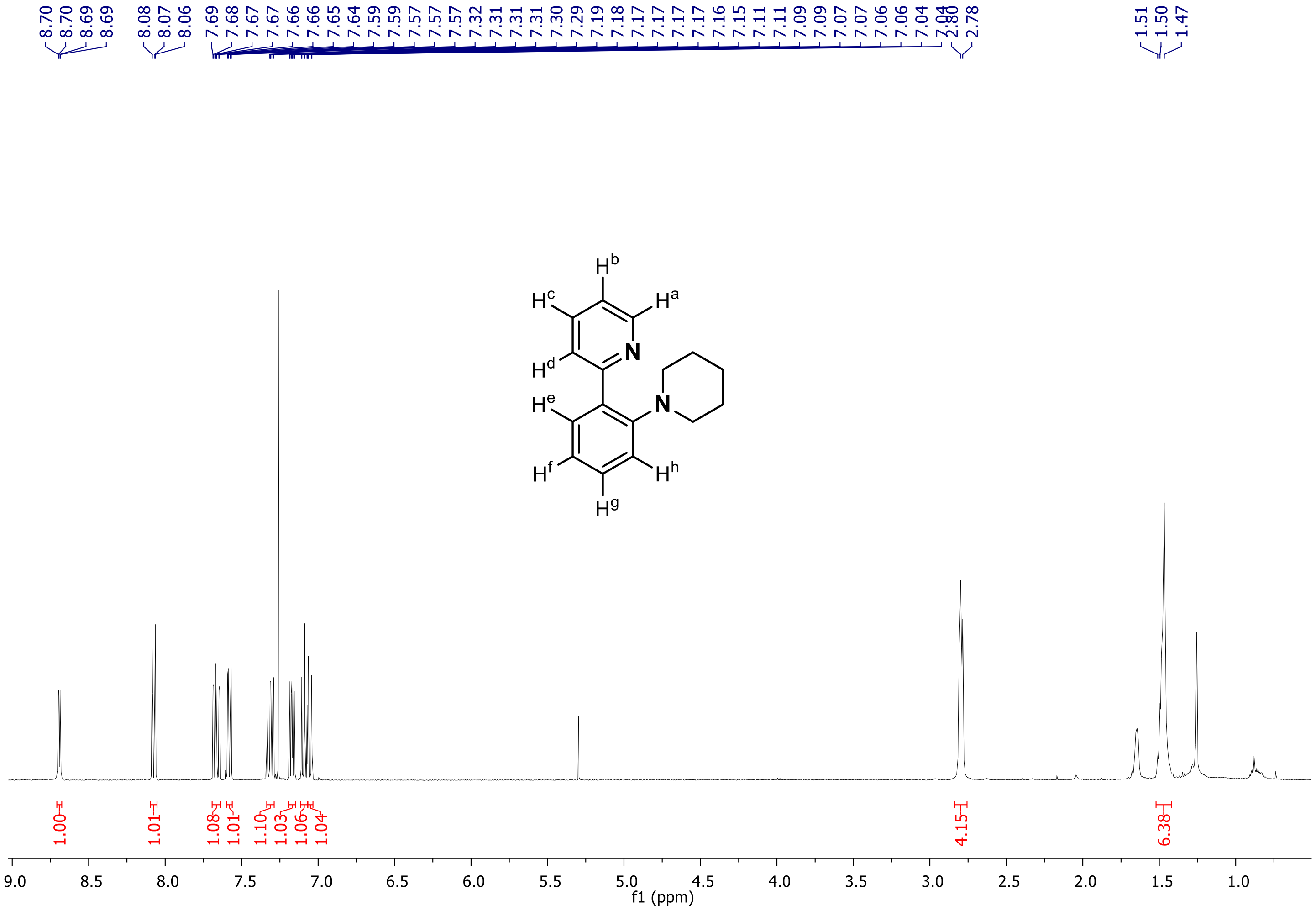

b)

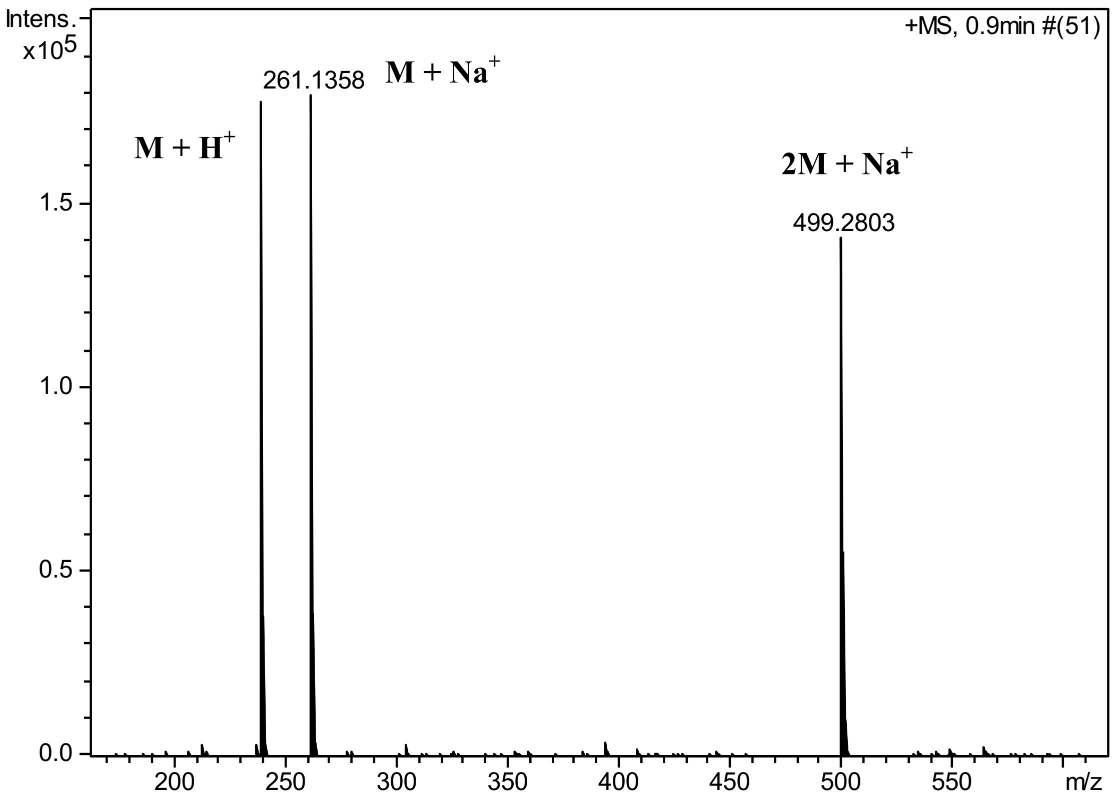

Intens.
x10^5
+MS, 0.9min #(51)
261.1358
M + Na+
M + H+
2M + Na+
499.2803
1.5
1.0
0.5
0.0
200
250
300
350
400
450
500
550
m/z

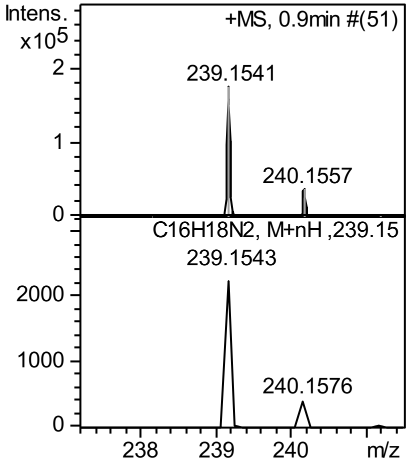

Intens.
x10^5
+MS, 0.9min #(51)
239.1541
240.1557
C16H18N2, M+nH ,239.15
239.1543
240.1576
2000
1000
238
239
240
m/z

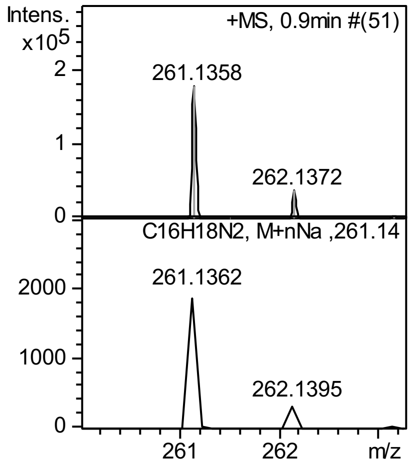

Intens.
x10^5
+MS, 0.9min #(51)
261.1358
262.1372
C16H18N2, M+nNa ,261.14
261.1362
262.1395
2000
1000
261
262
m/z

Figure S22. a) ^{1}H-NMR spectrum (CD_3CN, 298K) of the reaction crude of quantitative *p*-chlorophenolate insertion to **2a-Br** to form **2aa** (●) catalyzed by **3b**, followed by formation of [Au(Br)IPr] (▲) and liberation of the pyridine moiety (◆).

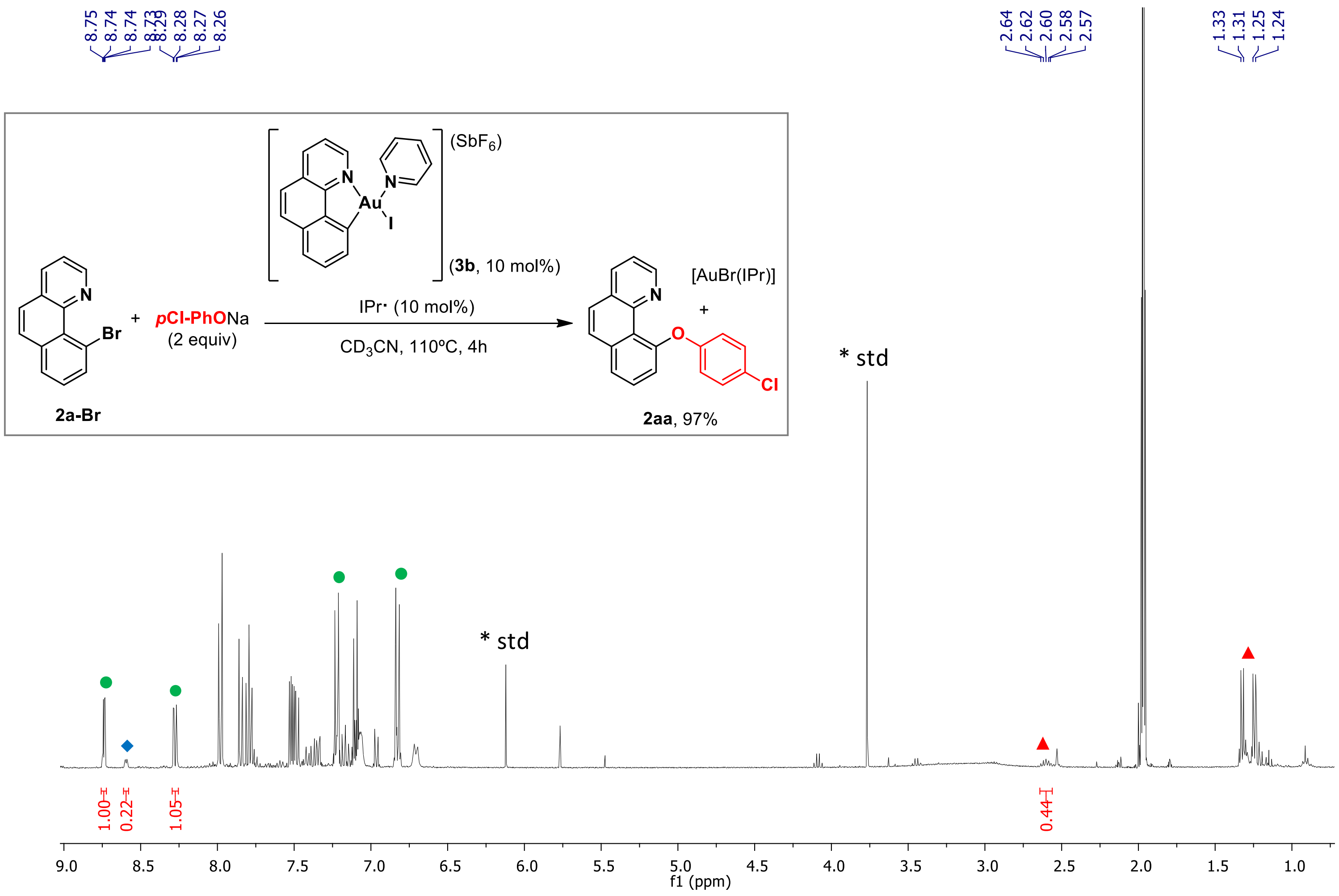

3. X-ray crystallography data

Crystallographic data for compound **1ah** (CCDC-1489676) and complexes **3a** (CCDC-1489681) and **3b** (CCDC-1489682) can be obtained free of charge from the Cambridge Crystallographic Data Centre (CCDC) *via* www.ccdc.cam.ac.uk/data_request/cif.

X-Ray structure of 1ah:

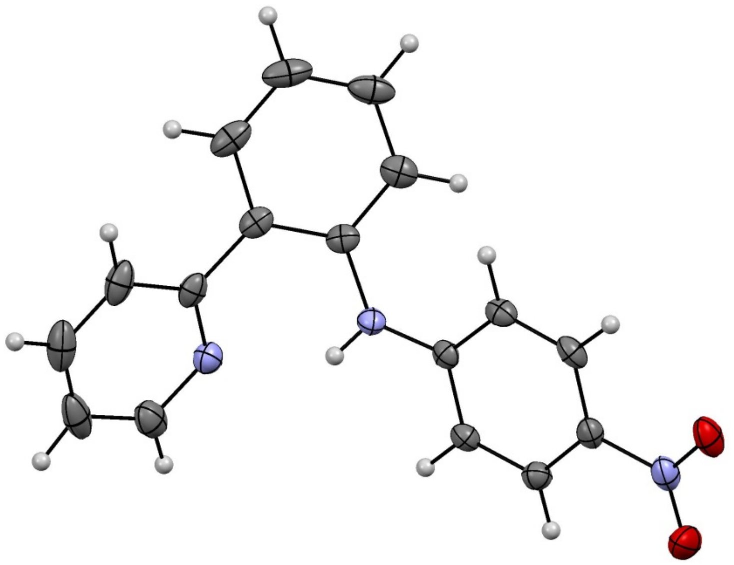

Figure S23. X-Ray structure of compound **1ah** obtained by crystallization from $CHCl_3$. Ellipsoids are set at 50% probability.

Yellow crystals were grown from slow evaporation of a $CHCl_3$ solution of the compound, and used for low temperature (100(2) K) X-ray structure determination. The measurement was carried out on a *BRUKER SMART APEX CCD* diffractometer using graphite-monochromated Mo $K\alpha$ radiation (λ = 0.71073 Å) from an x-Ray Tube. The measurements were made in the range 2.144 to 28.317° for θ. Full-sphere data collection was carried out with ω and φ scans. A total of 20282 reflections were collected of which 3428 [R(int) = 0.0312] were unique. Programs used: data collection, Smart[11]; data reduction, Saint+[12]; absorption correction, SADABS[13]. Structure solution and refinement was done using SHELXTL[14].

The structure was solved by direct methods and refined by full-matrix least-squares methods on F^2. The non-hydrogen atoms were refined anisotropically. The H-atoms were placed in geometrically optimized positions and refined without constrains or restrains.

**Chemical formula**	$C_{17}H_{13}N_3O_2$
**fw (g mol^{-1})**	291.30
**T (K)**	100(2)
**Space group**	Orthorhombic, P 21/c
a **(Å)**	13.283(2)
b **(Å)**	13.585(2)
c **(Å)**	7.6811(13)
**α (deg.)**	90
**β (deg.)**	90
**γ (deg.)**	90
V **(Å^3)**	1386.0(4)
**$\rho_{calcd.}$ (g cm^{-3})**	1.396
**λ (Å)**	0.71073
**R_1 [I>2sigma(I)]**	0.0380
**wR_2 [I>2sigma(I)]**	0.0873

X-Ray structure of 3a:

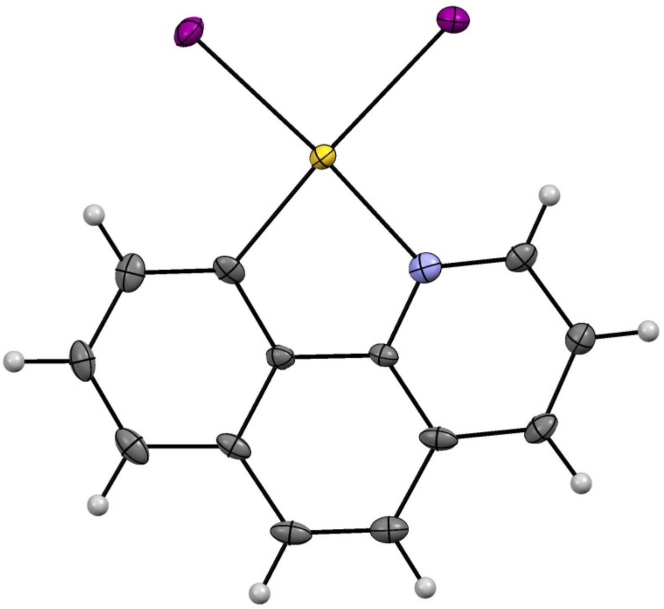

Figure S24. X-Ray structure of compound **3a** obtained by crystallization from CH_2Cl_2. Ellipsoids are set at 50% probability.

Red crystals were grown from CH_2Cl_2, and used for low temperature (100(2) K) X-ray structure determination. The measurement was carried out on a *BRUKER SMART APEX CCD* diffractometer using graphite-monochromated Mo *K*α radiation (λ = 0.71073 Å) from an x-Ray Tube. The measurements were made in the range 2.171 to 28.295° for θ. Full-sphere data collection was carried out with ω and φ scans. A total of 19253 reflections were collected of which 3202 [R(int) = 0.0647] were unique. Programs used: data collection, Smart[11]; data

reduction, Saint+[12]; absorption correction, SADABS[13]. Structure solution and refinement was done using SHELXTL[14].

The structure was solved by direct methods and refined by full-matrix least-squares methods on F^2. The non-hydrogen atoms were refined anisotropically. The H-atoms were placed in geometrically optimized positions and forced to ride on the atom to which they are attached.

**Chemical formula**	$C_{13}H_8AuI_2N$
**fw (g mol^{-1})**	628.97
**T (K)**	100(2)
**Space group**	Monoclinic, P 21/c
a **(Å)**	10.4599(18)
b **(Å)**	13.687(2)
c **(Å)**	10.1110(17)
**α (deg.)**	90
**β (deg.)**	116.270(3)
**γ (deg.)**	90
V **(Å^3)**	1298.1(4)
**$\rho_{calcd.}$ (g cm^{-3})**	3.218
**λ (Å)**	0.71073
**R_1 [I>2sigma(I)]**	0.0341
**wR_2 [I>2sigma(I)]**	0.0849

X-Ray structure of 3b:

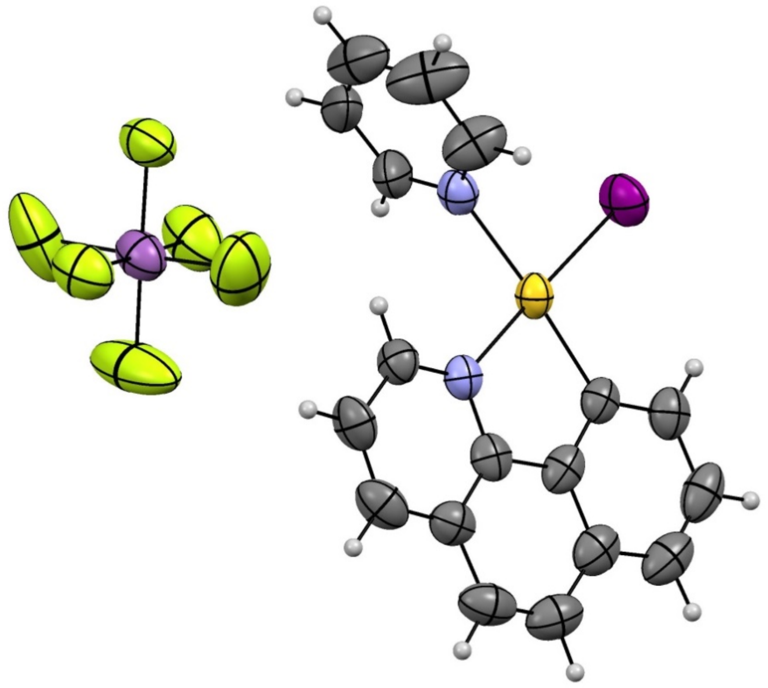

Figure S25. X-Ray structure of compound **3b** obtained by crystallization from CH_3CN and diethyl ether. Ellipsoids are set at 50% probability.

Orange crystals were grown from slow diffusion of diethyl ether in a CH_3CN solution of the compound, and used for low temperature (100(2) K) X-ray structure determination. The measurement was carried out on a *BRUKER SMART APEX CCD* diffractometer using graphite-monochromated Mo *K*α radiation (λ = 0.71073 Å) from an x-Ray Tube. The measurements were made in the range 2.07 to 28.58° for θ. Full-sphere data collection was carried out with ω and φ scans. A total of 62991 reflections were collected of which 10037 [R(int) = 0.0686] were unique. Programs used: data collection, Smart[11]; data reduction, Saint+[12]; absorption correction, SADABS[13]. Structure solution and refinement was done using SHELXTL[14].

The structure was solved by direct methods and refined by full-matrix least-squares methods on F^2. The non-hydrogen atoms were refined anisotropically. The H-atoms were placed in geometrically optimized positions and forced to ride on the atom to which they are attached.

**Chemical formula**	$C_{18}H_{13}AuIN_2F_6Sb$
**fw (g mol^{-1})**	816.92
**T (K)**	100(2)
**Space group**	Monoclinic, P 21/c
***a* (Å)**	14.260(9)
***b* (Å)**	20.667(13)
***c* (Å)**	14.033(9)
**α (deg.)**	90
**β (deg.)**	104.38(1)
**γ (deg.)**	90
***V* (Å^3)**	4006(4)
**$\rho_{calcd.}$ (g cm^{-3})**	2.709
**λ (Å)**	0.71073
**R_1 [I>2sigma(I)]**	0.0598
***w*R_2 [I>2sigma(I)]**	0.1587

4. References

[1] P. de Frémont, N. Marion, S. P. Nolan, *J. Organomet. Chem.*, **2009**, *694*, 551.

[2] X. Mu, H. Zhang, P. Chen, G. Liu, *Chem. Sci.*, **2014**, *5*, 275.

[3] A. Klapars, S. L. Buchwald, *J. Am. Chem. Soc.*, **2002**, *124*, 14844.

[4] J. Serra, C. Whiteoak, F. Acuña-Parés, M. Font, J. M. Luis, J. Lloret-Fillol, X. Ribas, *J. Am. Chem. Soc.,* **2015***, 137,* 13389.

[5] A. R. Dick, K. L. Hull, M. S. Sanford, *J. Am. Chem. Soc.*, **2004**, *126*, 2300.

[6] J. I. JuncosaJr., M. Hansen, L. A. Bonner, J. P. Cueva, R. Maglathlin, J. D. McCorvy, D. Marona-Lewicka, M. A. Lill, D. E. Nichols, *ACS Chem. Neurosci.*, **2013**, *4*, 96.

[7] T. Vogler, A. Studer, *Org. Lett.*, **2008**, *10*, 129.

[8] X. Chen, X.-S. Hao, C. E. Goodhue, J.-Q. Yu, *J. Am. Chem.*, **2006**, *128*, 6790.

[9] M. A. Ali, X. Yao, G. Li, H. Lu, *Org. Lett.*, **2016**, *18*, 1386.

[10] S. Thapa, A. Kafle, S. K. Gurung, A. Montoya, P. Riedel, R. Giri, *Angew. Chem. Int. Ed.*, **2015**, *54*, 8236.

[11] Bruker Advanced X-ray Solutions. SMART: Version 5.631, **1997-2002**.

[12] Bruker Advanced X-ray Solutions. SAINT +: Version 6.36A, **2001**.

[13] G. M. Sheldrick, *Emperical Absorption Correction Program*, Universität Göttingen, **1996**, Bruker Advanced X-ray Solutions. SADABS Version 2.10, **2001**.

[14] G. M. Sheldrick, *Program for Crystal Structure Refinement*, Universität Göttingen, **1997**, Bruker Advanced X-ray Solutions. SHELXTL Version 6.14, **2000-2003**. SHELXL-2013 (Sheldrick, **2013**).
